# Homo- and Cross-Coupling of Phenylacetylenes and α‑Hydroxyacetylenes Catalyzed by a Square-Planar Rhodium Monohydride

**DOI:** 10.1021/acscatal.4c00264

**Published:** 2024-05-14

**Authors:** Laura A. de las Heras, Miguel A. Esteruelas, Montserrat Oliván, Enrique Oñate

**Affiliations:** Departamento de Química Inorgánica—Instituto de Síntesis Química y Catálisis Homogénea (ISQCH)—Centro de Innovación en Química Avanzada (ORFEO-CINQA), 16765 Universidad de Zaragoza—CSIC, 50009 Zaragoza, Spain

**Keywords:** rhodium, hydride, alkyne, homocoupling, cross-coupling

## Abstract

The C–C triple bond of phenylacetylene undergoes the *anti*-Markovnikov addition of the Rh–H bond of RhH­{κ^3^-*P*,*O*,*P*-[xant­(P^i^Pr_2_)_2_]} (**1**; xant­(P^i^Pr_2_)_2_ = 9,9-dimethyl-4,5-bis­(diisopropylphosphino)­xanthene) to give Rh­{(*E*)–CH=CHPh}­{κ^3^-*P*,*O*,*P*-[xant­(P^i^Pr_2_)_2_]} (**2**), which reacts with a second alkyne molecule to produce Rh­(C≡CPh)­{κ^3^-*P,O,P*-[xant­(P^i^Pr_2_)_2_]} (**3**) and styrene before the transformation from **1** to **2** is complete. The metal center of **3** undergoes the oxidative addition of the C­(sp)–H bond of another alkyne molecule to produce RhH­(C≡CPh)_2_{κ^3^-*P*,*O*,*P*-[xant­(P^i^Pr_2_)_2_]} (**4**), which also reacts with more phenylacetylene before completing the transformation from **3** to **4**. The reaction leads to Rh­{(*E*)–CH=CHPh}­(C≡CPh)_2_{κ^3^-*P*,*O*,*P*-[xant­(P^i^Pr_2_)_2_]} (**5**), which reductively eliminates (*E*)-1,4-diphenyl-1-buten-3-yne to regenerate **3**. Complexes **3**, **4**, and **5** constitute a cycle for head-to-head dimerization of phenylacetylene. Consequently, complex **1** promotes the catalytic homocoupling of terminal alkynes to (*E*)-enynes, including the dimerization of α-hydroxyacetylenes to (*E*)-enyne-diols. The rate-determining step of the couplings depends on the nature of the alkyne, being the insertion of the C–C triple bond into the Rh–H bond of a bis­(acetylide)-rhodium­(III)-hydride intermediate for phenylacetylenes and the reductive elimination of the product (*E*)-enyne-diol for α-hydroxyacetylenes. In support of the latter, complex Rh­{(*E*)–CH=CHC­(OH)­Ph_2_}­{C≡CC­(OH)­Ph_2_}_2_{κ^3^-*P*,*O*,*P*-[xant­(P^i^Pr_2_)_2_]} (**6**) has been isolated and characterized by X-ray diffraction analysis. Complex **1** also effectively promotes the formation of compounds of the type (*E*)-5-phenyl-2-penten-4-yn-1-ol, by cross-coupling between phenylacetylenes and α-hydroxyacetylenes. These reactions take place through two cycles similar to the cycle that produces the homocouplings, the rate-determining step being the reductive elimination of (*E*)-enyn-ol for both. The catalytic performance of **1** provides good efficiency in homocoupling and cross-coupling reactions involving progestin-type compounds such as ethisterone.

## Graphical abstract

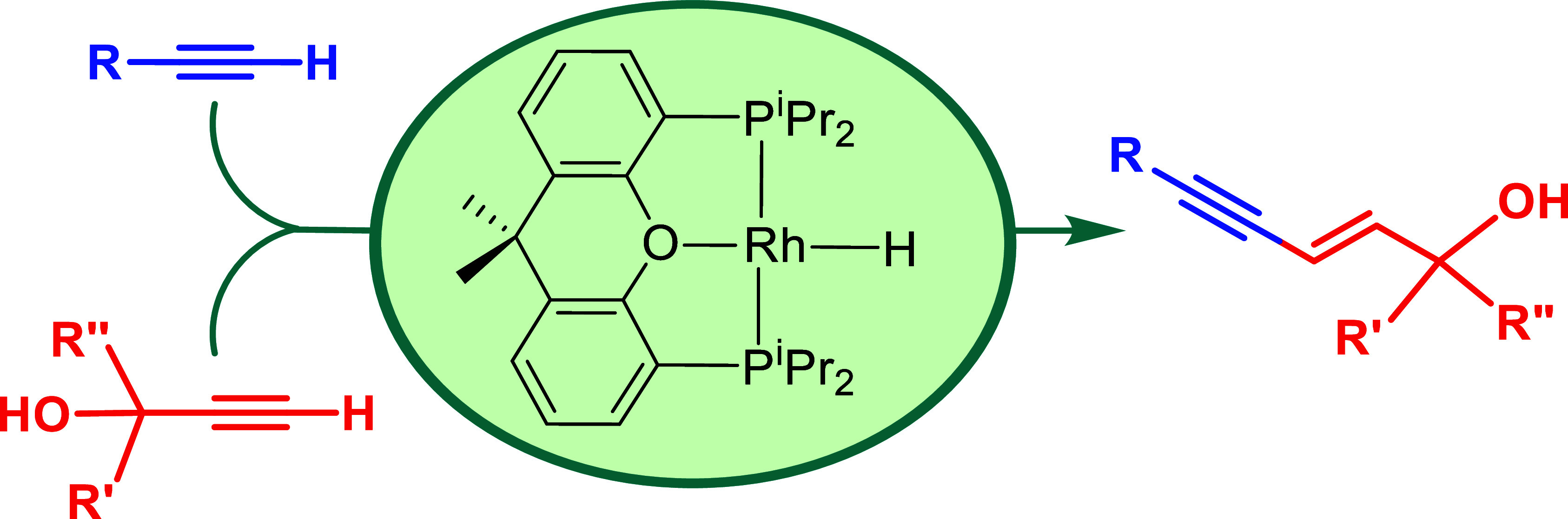

## Introduction

Diastereoselective coupling of terminal alkynes catalyzed by transition metal complexes is a direct and atom-economical procedure to form 1,3-enynes, which are important building blocks in organic synthesis.^1^ However, it is a great challenge; in the simplest case involving two molecules of the same alkyne, homocoupling, dimerization can be head-to-head or head-to-tail.^2^ The first type of coupling is able of generating (*E*)- and (*Z*)-1,2,3-butatrienes^3^ and (*E*)- and (*Z*)-1,4-disubstituted-1-buten-3-ynes, while 2,4-disubstituted-1-buten-3-ynes are formed via the second. Thus, the reactions can give two butatrienes in addition to three 1,3-enynes (Scheme 1), existing a limited number of catalysts capable of reaching high selectivity in the formation of only one of the 1,3-enynes.^2^

**Scheme 1:**
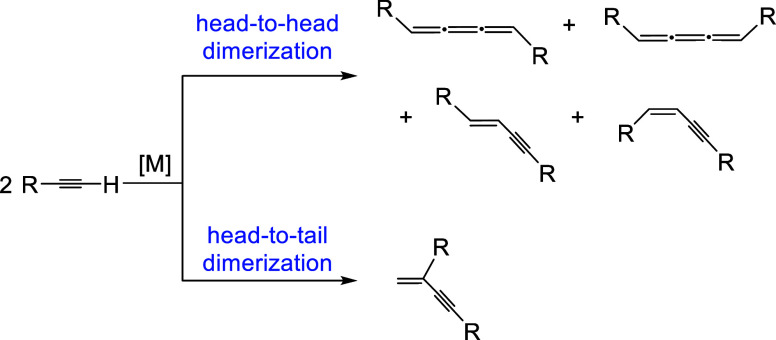
Homocoupling of Alkynes

The elementary steps for the dimerization to enynes are an insertion between two C­(sp)–H-bond activations. The insertion step decides the stereochemistry of the product, whereas the heterolytic or homolytic character of the C­(sp)–H bond rupture depends on the electronic nature of the catalyst precursor (Scheme 2). Electron-poor precursors promote heterolytic cleavage to produce an acetylide derivative. Subsequent insertion of the C–C triple bond of another alkyne molecule into the metal-acetylide bond leads to a η^3^-butenynyl intermediate, which then reacts with a new alkyne molecule to give the dimer and regenerate the active acetylide species (**a** in Scheme 2).^4^ In contrast, electron-rich precursors react by homolytic cleavage of the C­(sp)–H bond, to give a hydride–metal–acetylide complex. This species inserts the C–C triple bond of a new alkyne molecule, into the metal-hydride bond, to generate an alkenyl-metal-acetylide intermediate. In the presence of more alkyne, this intermediate undergoes reductive elimination of the dimer, regenerating the key hydride-metal-acetylide species (**b** in Scheme 2).^5^

**Scheme 2:**
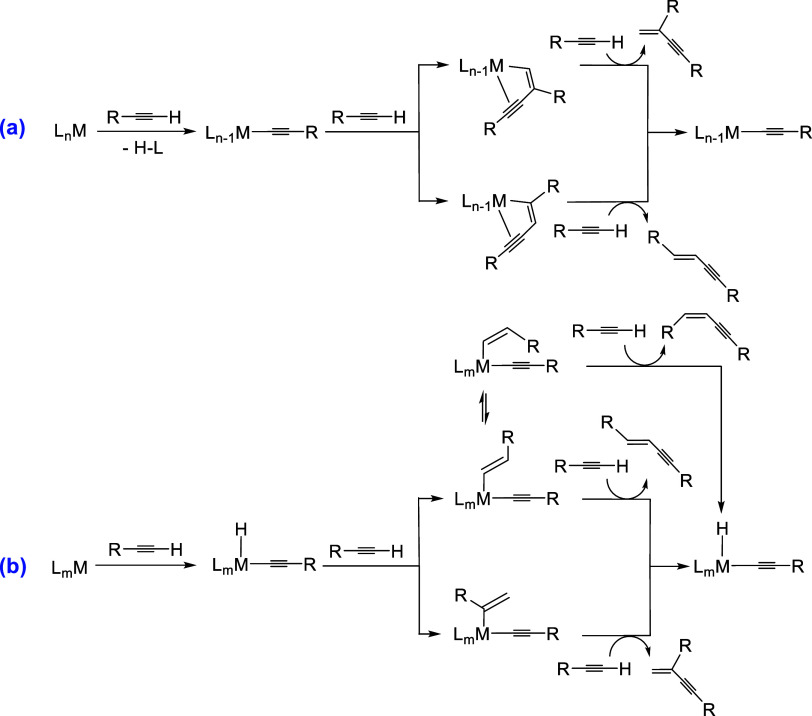
Reaction Pathways for the Dimerization of Terminal Alkynes to 1,3-Enynes

The α-hydroxyacetylenes occupy a prominent position among the alkyne substrates. They are present as an additional chemical function in several progestins, including norethindrone, norgestrel, ethisterone, and related steroid-containing molecules;^6^ relevant biologically active compounds, with interesting pharmaceutical applications.^7^ In addition, the dimerization products of these substrates, enyne-diols, are a direct pathway for the synthesis of enol ethers and a wide variety of oxygen-containing heterocycles.^8^ This class of substrates has surprisingly received very little attention, with reactions being limited to a few simple substrates. Rhodium catalysts promote the preferential formation of head-to-head (*E*)-products,^9^ whereas palladium precursors favor head-to-tail dimers.^10^ In contrast to the rhodium and palladium complexes, osmium­(IV)-polyhydride OsH_3_(SiEt_3_)­{κ^3^-*P*,*O*,*P*-[xant­(P^i^Pr_2_)_2_]} (xant­(P^i^Pr_2_)_2_ = 9,9-dimethyl-4,5-bis­(diisopropylphosphino)­xanthene) promotes the head-to-head dimerization of 1,1-diphenyl-2-propyn-1-ol to the *Z*-enyne-diol. Under the reaction conditions, the latter is not stable and evolves to a furanol, which results from the addition of the hydroxy group in 1-position to the C–C triple bond.^11^

Cross-coupling between different terminal alkynes represents a significant increase in the level of difficulty. Besides the problem of the formation of different stereoisomers, each alkyne molecule must play a specific role; one of them acting as a hydrogen donor and the other as a hydrogen acceptor. Furthermore, the cross-coupling is competitive with the homocoupling of both alkynes, all three couplings taking place through similar mechanisms. Thus, cross-couplings must have a lower activation energy than homocouplings to proceed successfully.^12^ To bypass these difficulties, one of the alkynes is often used in excess over the other.^4c,13^ α-Hydroxyacetylenes act as hydrogen acceptors in these reactions. Therefore, they are involved in the insertion step, which generally gives a M–CH=C­(C≡CR)­C­(OH)­R′_2_ intermediate (**a** in Scheme 2) or is of the Markovnikov-type (**b** in Scheme 2). Thus, the main reaction product is usually a *gem*-stereoisomer,^14^ with very few exceptions.^15^ Shaughnessy and co-workers reported a palladacycle-promoted cross-coupling between propargyl alcohols, with a sp^2^-hybridized substituent on the carbon atom at 1-position and phenylacetylene. In contrast to the common trend, the reactions produce (*E*)-5-phenyl-2-penten-4-yn-1-ol derivatives in 61–73% yield;^16^ compounds of particular interest, as they can be used as precursors of class NNC 61-4655 candidate drugs for type-2 diabetes.^17^

Pincer ligands have gained prominence in catalysis in the last two decades. The robustness provided to catalysts by the strong tridentate coordination of the pincer, which is highly desirable to withstand harsh reaction conditions, was initially argued as the main reason for its rapid implementation. Catalysts stabilized by pincer ligands are, however, much more, since they are demonstrating reactivity adapted to the requirements of certain transformations or behaviors in accordance with certain applications;^18^ abilities related to the properties of the pincer, including coordinative flexibility and hemilability. Diphosphine xant­(P^i^Pr_2_)_2_ ^19^ significantly improves the capabilities of classical *P*,*O*,*P*-ligands.^20^ It shows a greater tendency to coordinate as a pincer,^21^ providing robust catalysts.^22^ Despite this, its oxygen atom is hemilabile, which allows for bidentate coordination.^23^ In addition, it acts with a wide variety of coordination forms, including κ^3^-*P*,*O*,*P-mer* and -*fac*, and κ^2^ *-P*,*P-cis* and -*trans*.^24^ As a result, efficient ruthenium,^25^ osmium,^11,26^ rhodium,^27^ and iridium^28^ catalysts stabilized by this diphosphine have been reported for a variety of organic and inorganic reactions. Among them, the monohydride RhH­{κ^3^-*P*,*O*,*P*-[xant­(P^i^Pr_2_)_2_]} occupies a distinguished position; it catalyzes the borylation of arenes,^29^ the deuteration of boranes and hydrides of group 14 elements,^30^ the ammonia borane dehydrogenation,^31^ and the dehydropolymerization of amine-boranes.^32^

The versatility of this monohydride led us to explore its catalytic performance for the coupling of terminal alkynes. This paper demonstrates that monohydride RhH­{κ^3^-*P*,*O*,*P*-[xant­(P^i^Pr_2_)_2_]} is an efficient catalyst precursor for the homocoupling of a wide variety of terminal alkynes and α-hydroxyacetylenes, to selectively give (*E*)-enyne and (*E*)-enyne-diols, respectively. This complex also promotes cross-coupling between phenylacetylenes and α-hydroxyacetylenes with a secondary or tertiary alcohol function. The scope of cross-coupling includes ethisterone and leads to a broader range of (*E*)-5-phenyl-2-penten-4-yn-1-ol derivatives than that previously reported with the Shaughnessy’s palladacycle, with better selectivity and higher yields. In addition, the coupling mechanism is established through the characterization of the key catalytic intermediates and DFT calculations, and the reason for the observed selectivity is also analyzed.

## Results and Discussion

### Homocouplings

One of the most common reactions of unsaturated transition metal monohydride complexes is the addition of the M–H bond to the C–C triple bond of an alkyne to produce metal-alkenyl derivatives. The C–C double bond of the alkenyl ligand rarely prevents for the electron deficiency of the metal when it is a 4d element, in contrast to what is observed for the 5d derivatives of this class. Unsaturated complexes of platinum group metals also have a remarkable ability to activate σ-bonds; activation of the C­(sp)–H bond of terminal alkynes being particularly easy. The behavior of monohydride RhH­{κ^3^-*P*,*O*,*P*-[xant­(P^i^Pr_2_)_2_]} (**1**) toward phenylacetylene is consistent with this (Scheme 3).

**Scheme 3:**
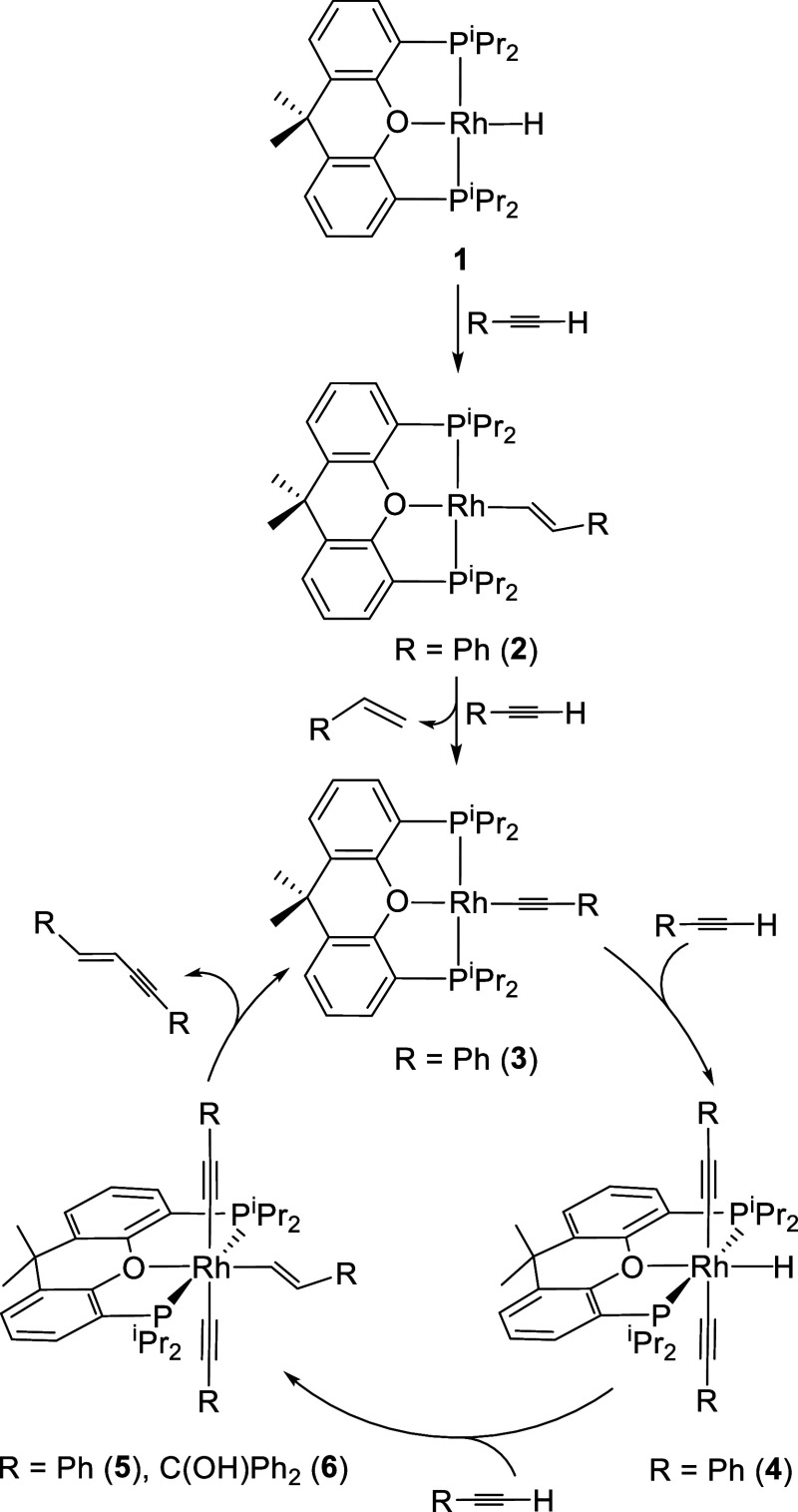
Reaction of **1** with Terminal Alkynes Showing the Spectroscopically Detected and Isolated Species

The C–C triple bond of phenylacetylene undergoes the *anti*-Markovnikov addition of the Rh–H bond of **1** to give the alkenyl derivative Rh­{(*E*)–CH=CHPh}­{κ^3^-*P,O,P*-[xant­(P^i^Pr_2_)_2_]} (**2**). However, the latter reacts with a second alkyne molecule before the transformation from **1** to **2** is completed. The reaction leads to the acetylide compound Rh­(C≡CPh)­{κ^3^-*P,O,P*-[xant­(P^i^Pr_2_)_2_]} (**3**) and styrene, as a result of the oxidative addition of the C­(sp)-H bond of phenylacetylene to the rhodium center of **2** and the subsequent reductive elimination of the olefin. Thus, the addition of less than 2.0 equiv of alkyne to solutions of **1**, in benzene-*d*_6_, at room temperature leads to mixtures of **2** and **3**, together with styrene in the same molar quantity as **3**. The presence of **2** in the mixtures is strongly supported by a doublet of triplets of doublets (^2^ *J*_H–Rh_ = 3 Hz, ^3^ *J*_H–P_ = 6 Hz, ^3^ *J*_H–H_ = 17 Hz) at 9.90 ppm in the ^1^H NMR spectrum, characteristic resonance for the RhCH-hydrogen atom (Figure S1), and a doublet (^1^ *J*_P–Rh_ = 176 Hz) at 38.6 ppm in the ^31^P­{^1^H} NMR spectrum (Figure S2). Complex **3** was isolated as a white solid in almost quantitative yield by addition of 2.5 equiv of alkyne to solutions of **1**, in pentane, at room temperature. The presence of the acetylide ligand is supported by the IR and the ^13^C­{^1^H} NMR spectrum, in benzene-*d*_6_, at room temperature. The first contains a characteristic ν­(C≡C) band at 2088 cm^–1^, whereas the second shows two doublets of triplets at 123.8 (^2^ *J*_C–Rh_ = 20.1 Hz, ^3^ *J*_C–P_ = 4.2 Hz) and 110.0 (^1^ *J*_C–Rh_ = 62.0 Hz, ^2^ *J*_C–P_ = 19.2 Hz) ppm due to the C_β_ and C_α_ atoms of the C-donor ligand, respectively. A doublet (^1^ *J*_P–Rh_ = 152 Hz) at 45.5 ppm in the ^31^P­{^1^H} NMR spectrum is another noticeable feature of this compound.

The metal center of **3** is coordinatively unsaturated, like that of **2**, and, in agreement with the latter, it undergoes oxidative addition of the C­(sp)–H bond of a new alkyne molecule to produce the monohydride-rhodium­(III) derivative RhH­(C≡CPh)_2_{κ^3^-*P,O,P*-[xant­(P^i^Pr_2_)_2_]} (**4**). Complex **4** is also unstable in the presence of the alkyne. In spite of its saturated character, it reacts with more phenylacetylene before the transformation from **3** to **4** is completed. The reaction leads to the alkenyl-rhodium­(III)-bis­(acetylide) species Rh­{(*E*)–CH=CHPh}­(C≡CPh)_2_{κ^3^-*P,O,P*-[xant­(P^i^Pr_2_)_2_]} (**5**), as a consequence of the *anti*-Markovnikov insertion of the C–C triple bond of the alkyne in the Rh–H bond. The hemilabile character of the diphosphine oxygen atom and the coordinative flexibility of this ligand allow coordination of the alkyne and their subsequent migratory insertion. As a result of both reactions, the addition of approximately 11 equiv of phenylacetylene to the solutions of **3**, in benzene-*d*_6_, at room temperature generates a mixture of **3**, **4**, and **5** in a molar ratio of about 25:67:8 after 10–20 min (Figure S6). Spectroscopic identification features of **4** are a doublet of triplets (^1^ *J*_H–Rh_ = 32.2 Hz, ^2^ *J*_H–P_ = 12.0 Hz) at −18.84 ppm, corresponding to the hydride ligand, in the ^1^H NMR spectrum (Figure S7) and a doublet (^1^ *J*_P–Rh_ = 100 Hz) at 54.4 ppm in the ^31^P­{^1^H} NMR spectrum (Figure S6). The presence of **5** in the mixture is supported by a doublet of triplets of doublets (^2^ *J*_H–Rh_ = 1.2 Hz, ^3^ *J*_H–P_ = 2.1 Hz, ^3^ *J*_H–H_ = 14.3 Hz) at 8.58 ppm, typical for a RhCH hydrogen atom (Figure S8), and a doublet with a characteristic P–Rh coupling constant for rhodium­(III) of 100 Hz at 32.9 ppm in the ^31^P­{^1^H} NMR spectrum (Figure S9). The mutually *trans* arrangement of the acetylide ligands in both compounds was inferred from the aliphatic resonances in the ^1^H NMR spectra, which for each compound show two signals for the methyl groups of the diisopropylphosphine substituents and one signal for the methyl substituents of the central heterocycle. This signal pattern is consistent with the existence of a molecular plane of symmetry, which makes equivalent the two acetylide ligands, the isopropyl substituents of each P^i^Pr_2_ group, and the methyl substituents of the central heterocycle.

Complex **5** reductively eliminates (*E*)-1,4-diphenyl-1-buten-3-yne to regenerate **3**. Therefore, complexes **3**, **4**, and **5** constitute a stoichiometric cycle for head-to-head (*E*)-dimerization of phenylacetylene. To obtain information on the catalytic performance of this cycle, we treated complex **3** with 20 equiv of alkyne, in benzene-*d*_6_, at 80 °C. As expected, the selective formation of (*E*)-1,4-diphenyl-1-buten-3-yne was observed, in almost quantitative yield (93%), after 10 h. Figure 1a shows the transformation profile, while Figure 1b provides the ^31^P­{^1^H} NMR spectra of the solution as a function of time. The spectra show that the cycle generated is, of course, catalytic; complexes **3** and **4** are present in all the spectra and some of them also contain traces of **5**. A qualitative analysis of the variation of the concentration of the species observed over time also indicates that (i) the rhodium­(I)-acetylide complex **3** is the organometallic species that initiates and recovers from catalysis. (ii) The monohydride-rhodium­(III) derivative **4** is a catalytic intermediate, which is in equilibrium with **3**. (iii) Insertion of the C–C triple bond of the alkyne into the Rh–H bond of **4** to give **5** is the rate-determining step of the catalysis, since complex **4** is the main metallic species for higher alkyne concentrations. (iv) The reductive elimination of the (*E*)-1,3-enyne is fast.

**Figure 1:**
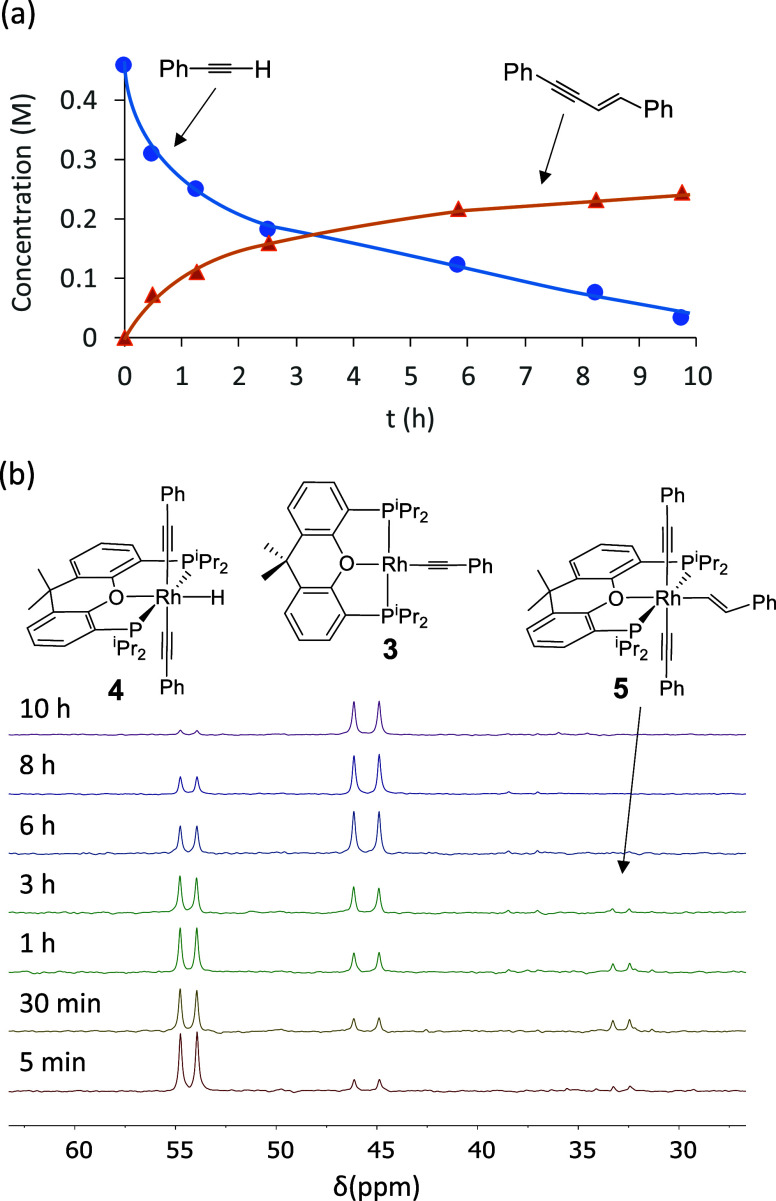
(a) Profile of the dimerization of phenylacetylene to (*E*)-1,4-diphenyl-1-buten-3-yne. (b) Stacked ^31^P­{^1^H} NMR spectra showing the reaction of complex **3** with 20 equiv of phenylacetylene (benzene-*d*_6_, 80 °C).

Having established the catalytic character of the cycle shown in Scheme 3, we decided to study the range of alkynes that can be coupled in a similar way. Scheme 3 indicates that each dimerization takes place through its own square-planar rhodium­(I) acetylide derivative. These species result from the reaction of monohydride **1** with 2.0 equiv of alkyne. Therefore, they can be generated in the reaction by adding an additional 2.0 equiv of substrate. The use of 2.4 mol % of acetylide complex with regard to the total amount of alkyne, benzene, and 80 °C are appropriate catalytic conditions to perform the dimerization. Under these conditions, complex **1** is an efficient catalyst precursor to promote head-to-head dimerization of a variety of substituted phenylacetylenes, showing good tolerance to functional groups (CF_3_, F, CN, OMe, and NMe_2_). The corresponding (*E*)-1,3-enynes (**a**_**4**_ **–f**_**4**_) are selectively obtained with a high yield (≈90%), after 22 h, in all cases. 2-Ethynyl-naphthalene, 2-methyl-1-buten-3-yne, 3-phenyl-1-propyne, and trimethylsilylacetylene also efficiently and selectively homocouple to produce the respective (*E*)-1,3-enynes (**g**_**4**_ **–j**_**4**_) (Scheme 4). In contrast, *tert*-butylacetylene does not give significant yields of dimer.

**Scheme 4:**
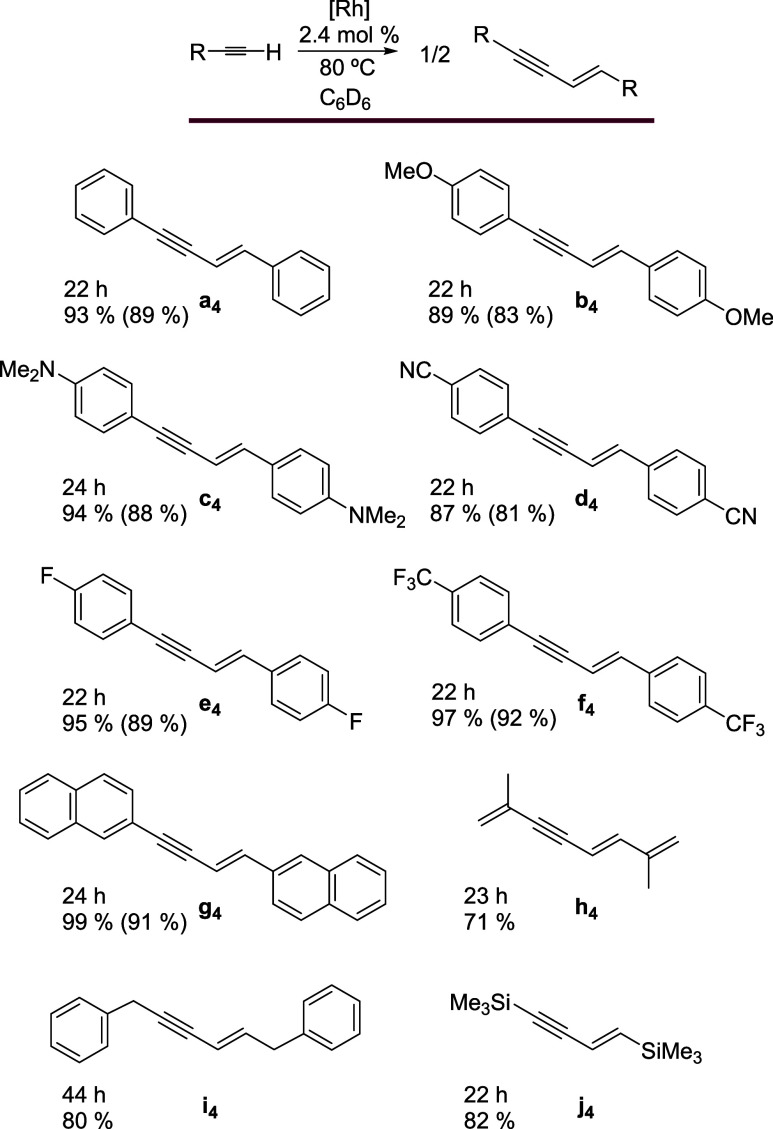
Homocoupling of Terminal Alkynes^a^

a Conditions: **1** (5 mg, 0.009 mmol), alkyne (0.38 mmol), and benzene-*d*_6_ (0.4 mL). Yields were determined by ^1^H NMR using dioxane or 1,1,2,2-tetrachloroethane as internal standards; isolated yields are given in parentheses.

Tolerance of **1** to functional groups includes alcohols. This monohydride derivative is also an efficient catalyst precursor for the homocoupling of α-hydroxyacetylenes having a secondary or tertiary alcohol function. Under the same conditions as those used in the dimerization of phenylacetylenes, it promotes head-to-head dimerization of a wide range of these substrates, including compounds with alkyl, phenyl, and vinyl substituents at the alcohol function. The coupling selectively generates (*E*)-enyne-diols **a**_**5**_ **-i**_**5**_ (Scheme 5), in very high yields (80–90%). Preparation of nonreported compounds (*E*)-2,9-dimethyl-4-decen-6-yne-3,8-diol (**b**_**5**_), (*E*)-2,11-dimethyl-5-decen-7-yne-4,9-diol (**c**_**5**_), and (*E*)-3,8-dimethyl-1,4,9-decatrien-6-yne-3,8-diol (**d**_**5**_) and their characterizations should be highlighted.

**Scheme 5:**
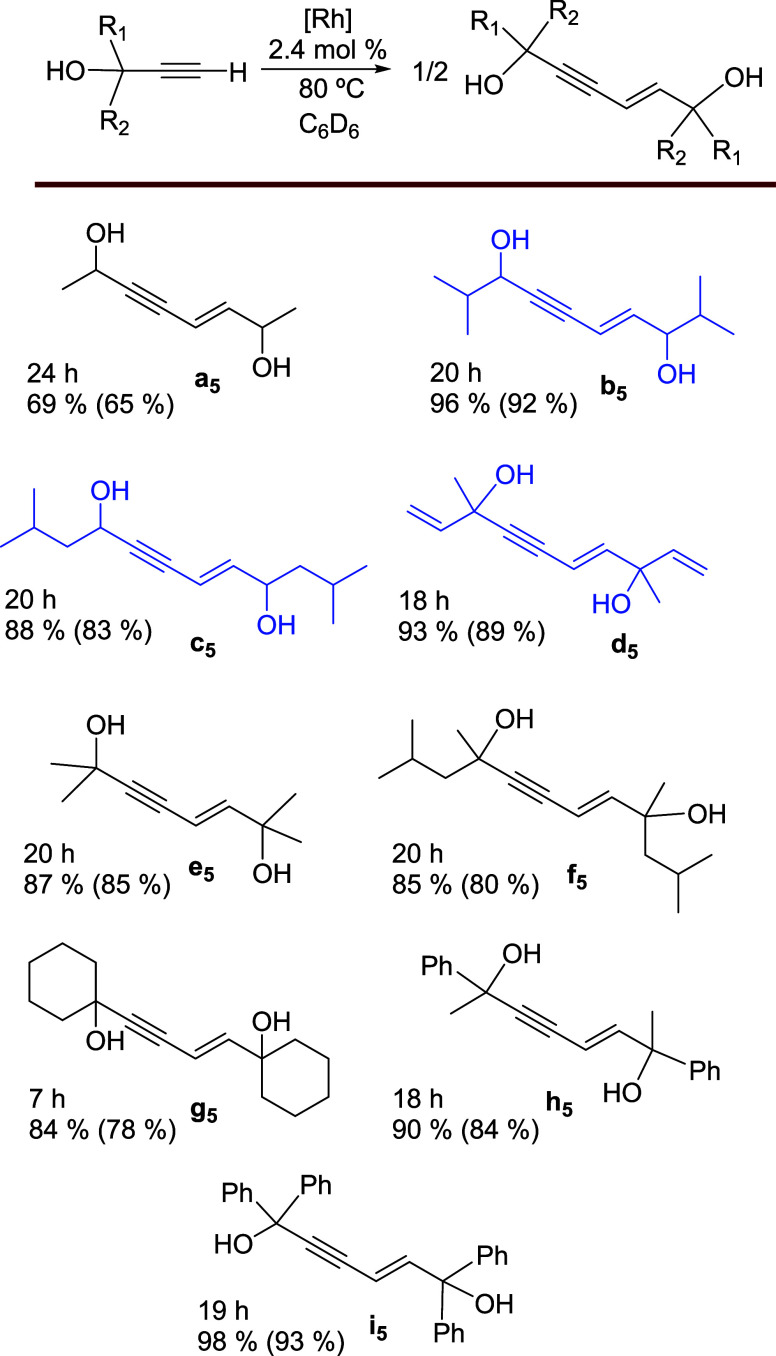
Homocoupling of α-Hydroxyacetylenes^a^

a Nonreported compounds in blue. Conditions: **1** (5 mg, 0.009 mmol), alkyne (0.38 mmol), and benzene-*d*_6_ (0.4 mL). Yields were determined by ^1^H NMR using dioxane as internal standard; isolated yields are given in parentheses.

The ^31^P­{^1^H} NMR spectra of the solution, in which the formation of (*E*)-1,1,6,6-tetraphenyl-2-hexen-4-yne-1,6-diol (**i**_**5**_) takes place, revealed that the (α-hydroxyalkenyl)-rhodium­(III)-bis­(α-hydroxyacetylide) complex Rh­{(*E*)–CH=CHC­(OH)­Ph_2_}­{C≡CC­(OH)­Ph_2_}_2_{κ^3^-*P,O,P*-[xant­(P^i^Pr_2_)_2_]} (**6**) is the main rhodium species in the reaction (Figure S10); the analogue of **5**. Its presence rests on a doublet (^1^ *J*_P–Rh_ = 101.5 Hz) at 34.6 ppm. Complex **6** was obtained on a preparative scale as a white solid, in 82% yield, by treating **1** with 4.0 equiv of 1,1-diphenyl-2-propyn-1-ol, in toluene, at room temperature. It was then characterized by X-ray diffraction analysis. The structure has two chemically equivalent but crystallographically independent molecules in the asymmetric unit; Figure 2 shows one of them. The coordination polyhedron around the rhodium­(III) center is an octahedron, as befits a d^6^ six-coordinate species. The ether-diphosphine coordinates *mer*, with angles P(1)–Rh(1)–P(2) of 163.18(11) and 161.65(12)°. In addition, the structure confirms the *trans* arrangement of the α-hydroxyacetylide ligands, which form C(16)–Rh(1)–C(31) angles of 178.5(5)° and 177.2(4)°, and the *trans* arrangement of the α-hydroxyalkenyl group to the etherdiphosphine oxygen atom (C(1)–Rh(1)–O(1) = 174.3(4) and 177.7(5)°). The α-hydroxyalkenyl group shows an *E* stereochemistry at the C–C double bond, as expected for a concerted *anti*-Markovnikov addition of a Rh–H bond to the C–C triple bond of a terminal alkyne. The ^13^C­{^1^H} NMR spectrum in benzene-*d*_6_ is consistent with the presence of the α-hydroxyacetylide and α-hydroxyalkenyl ligands in the complex. The C­(sp) carbon atoms of the equivalent α-hydroxyacetylide groups give rise to doublets of triplets at 113.4 (C_α_, ^1^ *J*_C–Rh_ = 38.9 Hz, ^2^ *J*_C–P_ = 15.5 Hz) and 109.8 (C_β_, ^1^ *J*_C–Rh_ = 3.0 Hz, ^2^ *J*_C–P_ = 0.8 Hz) ppm, while the C­(sp^2^) atoms of the vinylic moiety of the α-hydroxyalkenyl ligand generate a triplet (C_β_, ^3^ *J*_C–P_ = 3.7 Hz) at 139.0 and a doublet of triplets (C_α_,^1^ *J*_C–Rh_ = 34.1 Hz, ^2^ *J*_C–P_ = 9.9 Hz) at 129.7 ppm.

**Figure 2:**
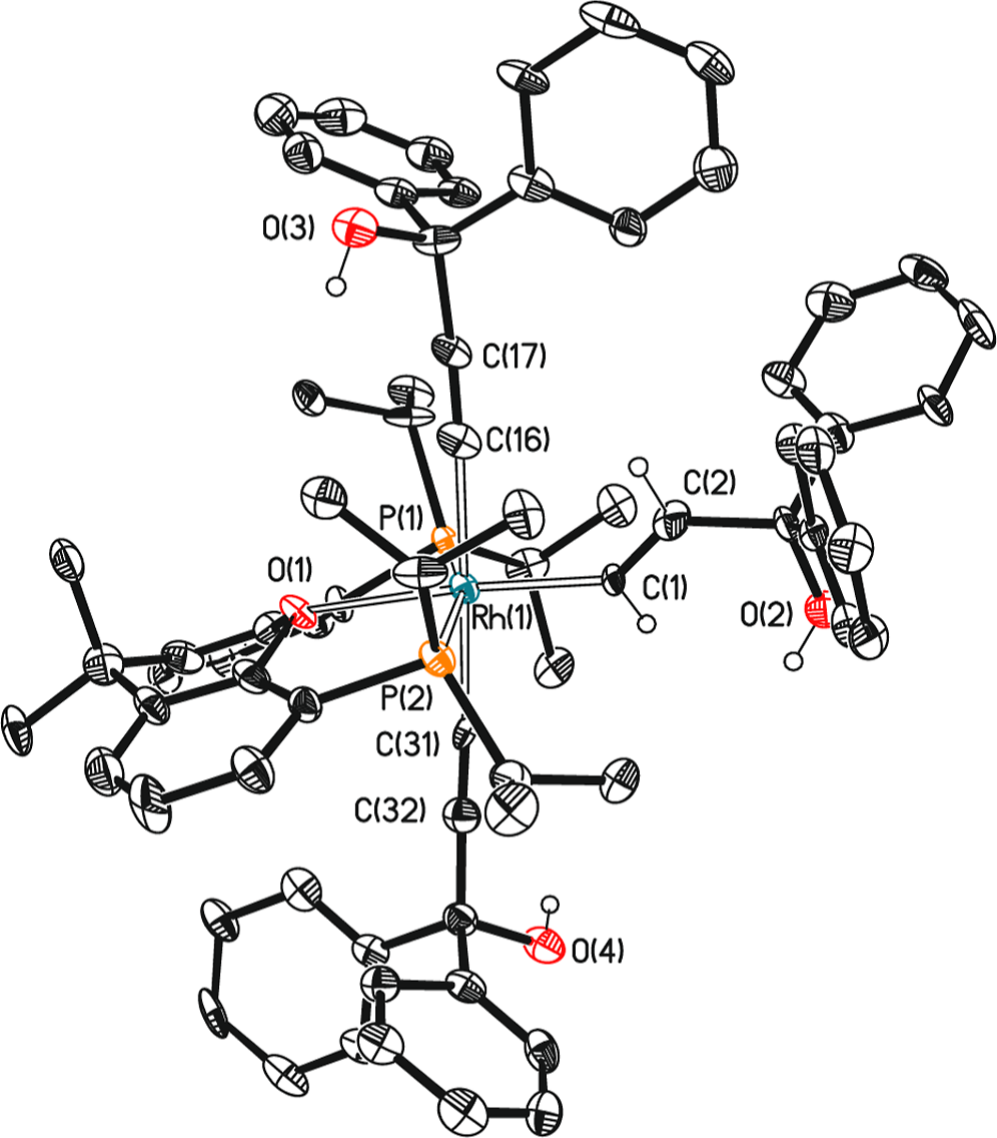
Molecular diagram of one of the two chemically equivalent but crystallographically independent molecules of complex **6** (displacement ellipsoids shown at 50% probability). All hydrogen atoms (except those of the vinyl and OH moieties) are omitted for clarity. Selected bond distances (Å) and angles (deg): Rh(1)–P(1) = 2.304(3), 2.320(3), Rh(1)–P(2) = 2.306(3), 2.304(3), Rh(1)–O(1) = 2.291(8), 2.278(8), Rh(1)–C(1) = 2.016(10), 2.014(11), Rh(1)–C(16) = 2.046(13), 2.012(11), Rh(1)–C(31) = 2.049(12), 2.050(12), C(1)–C(2) = 1.317(17), 1.310(17), C(16)–C(17) = 1.203(18), 1.207(17), C(31)–C(32) = 1.208(17), 1.215(17); P(1)–Rh(1)–P(2) = 163.18(11), 161.65(12), O(1)–Rh(1)–C(1) = 174.3(4), 177.7(4), C(16)–Rh(1)–C(31) = 178.5(5), 177.2(4).

Complex **6** reductively eliminates (*E*)-1,1,6,6-tetraphenyl-2-hexen-4-yne-1,6-diol, in benzene-*d*_6_, to produce the square planar derivative Rh­{C≡CC­(OH)­Ph_2_}­{κ^3^-*P,O,P*-[xant­(P^i^Pr_2_)_2_]} (**7**; δ_31P_, 45.9 ppm; ^1^ *J*_P–Rh_ = 151.5 Hz); the analogue of **3**. This stoichiometric reaction and the presence of **6** in the catalytic solution as the main organometallic species suggest that the sequence of events for the dimerization of 1,1-diphenyl-2-propyn-1-ol is the same as for the dimerization of phenylacetylene, although the steps that determine the rate of both reactions are different. Unlike the dimerization of phenylacetylene, the rate-determining step for the dimerization of 1,1-diphenyl-2-propyn-1-ol is the reductive elimination of (*E*)-1,1,6,6-tetraphenyl-2-hexen-4-yne-1,6-diol. Accordingly, the activation parameters for the stoichiometric reductive elimination and for the catalytic reaction are the same; Δ*H* ^≠^ = 19.7 ± 1.2 kcal mol^–1^, Δ*S* ^≠^ = −16.9 ± 3.5 cal^–1^ K^–1^ mol^–1^, and ^298^Δ*G* ^≠^ = 24.7 ± 2.2 kcal mol^–1^ (Figure 3).

**Figure 3:**
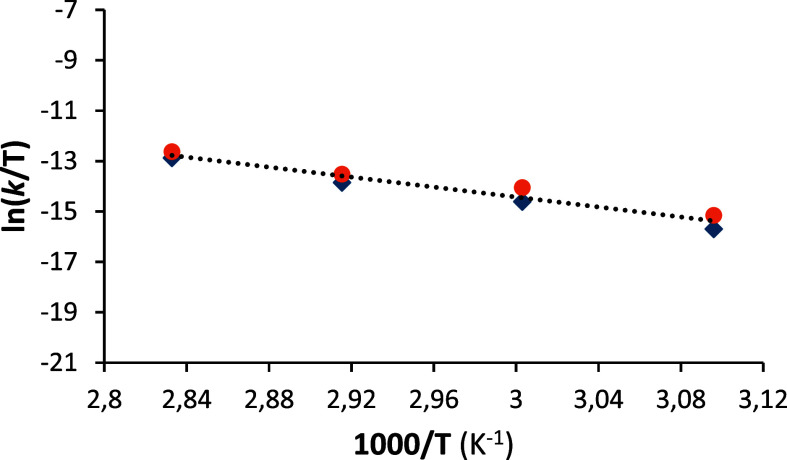
Eyring plot for the reductive elimination of (*E*)-1,1,6,6-tetraphenyl-2-hexen-4-yne-1,6-diol from **6** (blue squares) and for the catalytic dimerization of 1,1-diphenyl-2-propyn-1-ol (orange circles).

The DFT calculations (SMD-(toluene)-B3LYP-D3//SDD­(f)/6-31-G**) perfectly reproduce the cycle shown in Scheme 3 as well as the differences between phenylacetylenes and α-hydroxyacetylenes, for our delight. The changes in free energy (Δ*G*) were calculated at 298.15 K and 1 atm using phenylacetylene and 2-methyl-3-butyn-2-ol as alkyne models. Figure 4a shows the energy profile calculated for the homocoupling of the former, whereas Figure 4b shows that for the dimerization of the latter.

**Figure 4:**
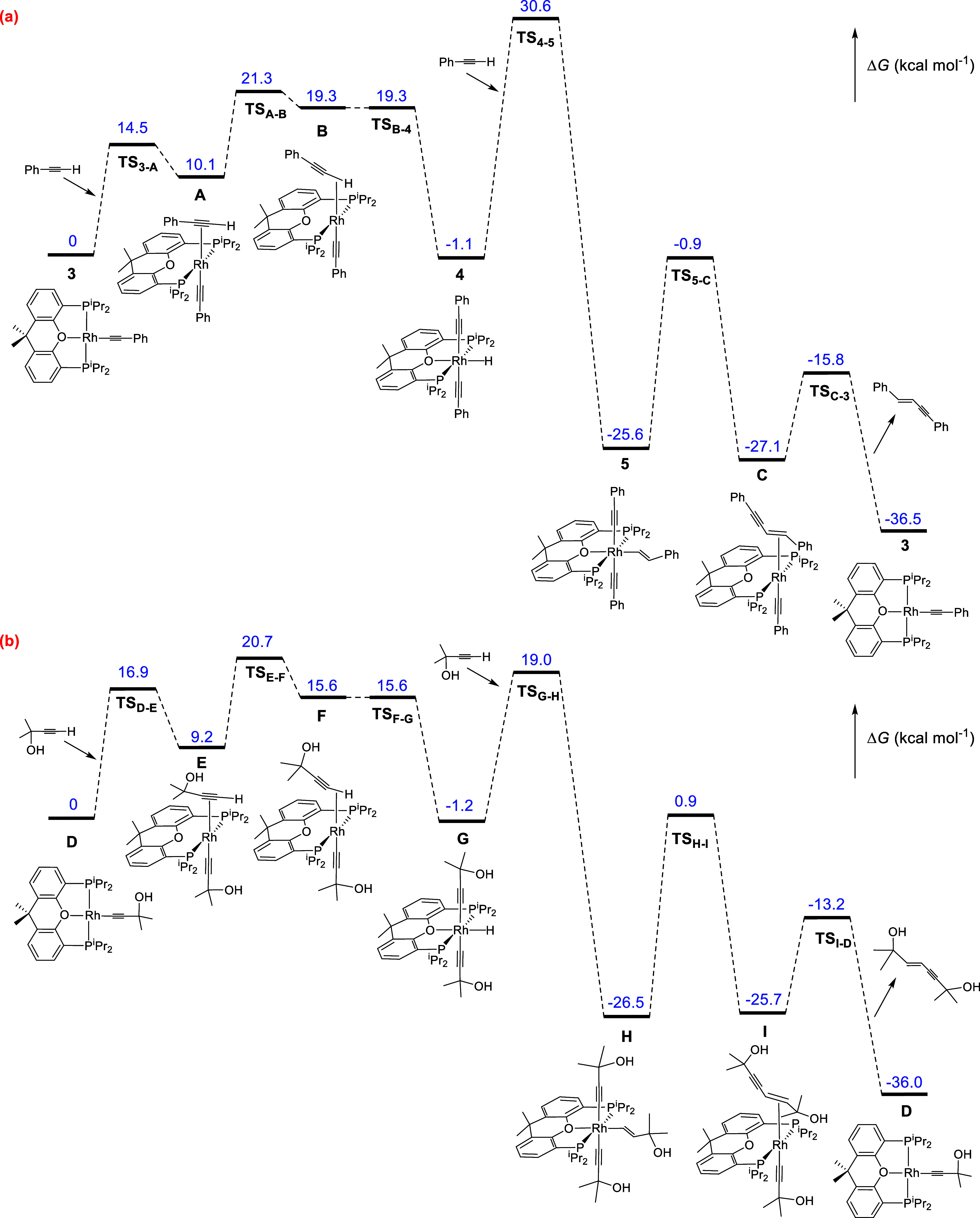
Computed energy profile (Δ*G*, in kcal mol^–1^) for the homocoupling of phenylacetylene (a) and 2-methyl-3-butyn-2-ol (b).

The oxidative addition of the C­(sp)–H bond of the substrates to the respective rhodium­(I)-acetylide catalysts **3** (Figure 4a) and **D** (Figure 4b) takes place in three steps. The first step involves the coordination of the C–C triple bond of the alkynes with the unsaturated metal center of the catalysts, initially leading to the π-alkyne intermediates **A** and **E**. In the second step, the metal center slides toward the C­(sp)–H bond, to give **B** and **F**. In the third step, the σ-intermediates **B** and **F** undergo homolytic cleavage of the coordinated σ-bond. The generated rhodium­(III) species **4** and **G** are approximately 1 kcal mol^–1^ more stable than the respective rhodium­(I)-acetylide compounds. This small difference in stability between the hydride-rhodium­(III)-bis­(acetylide) intermediates and the rhodium­(I)-acetylide species is consistent with the coexistence of both in equilibrium, as revealed by the ^31^P­{^1^H} NMR spectra shown in Figure 1b. The oxidative additions must overcome a maximum barrier of approximately 21 kcal mol^–1^, which corresponds to the sliding of the metal center from the C–C triple bond to the σ–C–H bond.

The insertions of the C–C triple bond of alkynes into the Rh–H bond of the hydride-rhodium­(III)-bis­(acetylide) intermediates **4** and **G** to produce **5** and **H**, respectively, are concerted and occur throughout the typical transition states of four centers. Just as the C–C triple bond of the alkyne approaches the saturated metal center, the oxygen atom of diphosphine separates from the rhodium atom. In the transition states (Figure 5), the pincer ether-diphosphine becomes bidentate, acting with P–Rh–P angles of 117.1° in **TS**_**4–5**_ and 114.2° in **TS**_**G‑H**_. Although **TS**_**4–5**_ and **TS**_**G‑H**_ may superficially appear similar, there is a major difference in the barriers of the insertion process; while **TS**_**4–5**_ lies at 31.7 kcal mol^–1^ over **4**, **TS**_**G‑H**_ is only 20.2 kcal mol^–1^ over **G**. The reason for such a surprising difference seems to be an electrostatic interaction between the oxygen atom of α-hydroxyacetylene and the weakly acidic hydride, which stabilizes **TS**_**G‑H**_ with respect to **TS**_**4–5**_. The electrostatic character of the interaction in **TS**_**G‑H**_ is supported by the hydride-oxygen separation of 2.311 Å, which is shorter than the sum of the van der Waals radii of hydrogen and oxygen (*r*_vdw_(H) = 1.20 Å, *r*_vdw_(O) = 1.52 Å),^33^ and the Mulliken and NBO charges of the hydride (+0.067 and +0.096) and the oxygen atom of the α-hydroxyacetylene (−0.514 and −0.760).

**Figure 5:**
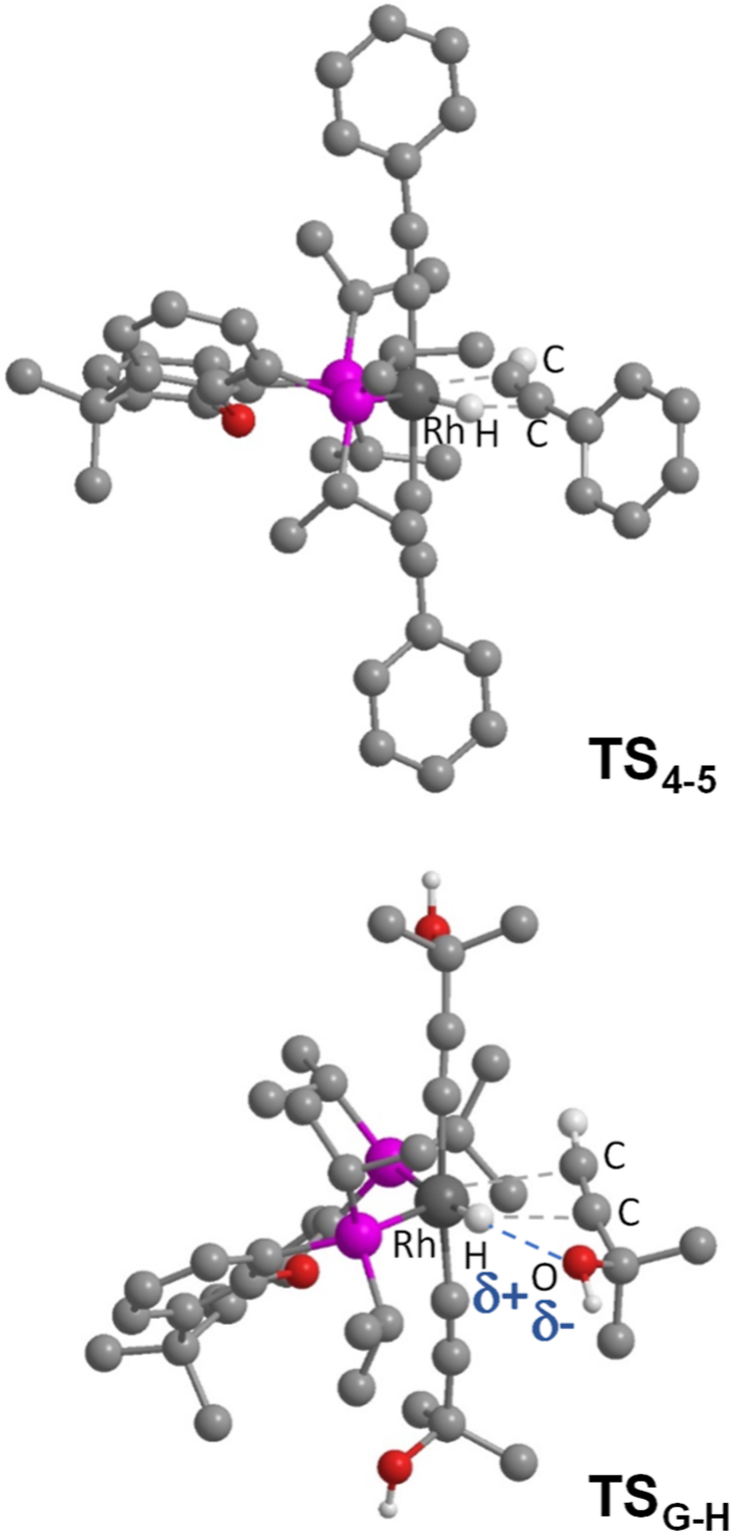
Transition states for the insertion of phenylacetylene (top) and 2-methyl-3-butyn-2-ol (bottom) into the Rh–H bond of the hydride-rhodium­(III)-bis­(acetylide) intermediates **4** and **G**. Hydrogens (except the hydrides, the OH and the C­(sp)–H protons of the alkyne) are omitted for clarity.

Intermediates **5** and **H** evolve by reductive coupling of the alkenyl group and one of the acetylide ligands to give the enyne derivatives **C** and **I**, which release the coupled fragments to close the cycle. Reductive eliminations present similar barriers; 24.7 kcal mol^–1^ for the dimerization of phenylacetylene and 27.4 kcal mol^–1^ for the formation of enyne-diol. The contextualization of these values in the respective catalytic sequences indicates that the rate-determining dimerization step depends on the nature of the alkyne, although the steps required for both catalysis are similar, as expected based on the experimental results. The insertion of the C–C bond of the alkyne into the Rh–H bond of **4** is the rate-determining step for the dimerization of phenylacetylene. However, the reductive elimination of enyne-diol from the intermediate (α-hydroxyalkenyl)-rhodium­(III)-bis­(α-hydroxyacetylide) **H** is the rate-determining step for the homocoupling of α-hydroxyacetylene. Because the barrier for insertion of the C–C bond of phenylacetylene into the Rh–H bond of **4** is higher than that for the reductive elimination of enyne-diol from the (α-hydroxyalkenyl)-rhodium­(III)-bis­(α-hydroxyacetylide) **H** intermediate, 2-methyl-3-butyn-2-ol homocoupling is faster than phenylacetylene homocoupling (Figure S115).

### Cross-Coupling of Phenylacetylenes and α-Hydroxyacetylenes

Complex **1** is also an efficient catalyst precursor for the formation of a variety of enyn-ols of the (*E*)-5-phenyl-2-penten-4-yn-1-ol type, by cross-coupling between phenylacetylenes and α-hydroxyacetylenes and trimethylsilyl-related derivatives resulting from the use of trimethylsilylacetylene instead of arylacetylenes. Phenylacetylenes include substrates with MeO, CN, and CF_3_ substituents on the phenyl group, while α-hydroxyacetylenes are substituted terminal alkynes with secondary and tertiary alcohol functions that result from the presence of alkyl, phenyl, or vinyl substituents. The reactions were carried out in benzene, at 80 °C, with equimolar amounts of substrates, using 5 mol % of precursor with respect to both alkynes. Products **a**_**6**_ **–m**_**6**_ were formed with yields of 80–90%, after 24 h. The yields are independent of the substituent of the C–C triple bonds, including the secondary or tertiary character of the alcohol, and the alkyl, phenyl, or vinyl nature of the alcohol substituents (Scheme 6). The efficiency of **1** is higher than that reported for Shaughnessy’s palladacycle in the cross-coupling between phenylacetylenes and α-hydroxyacetylenes, with primary and secondary alcohol functions, since our yields are higher. Furthermore, it should be noted that the Shaughnessy reactions were performed using an excess of α-hydroxyacetylene, between 2.5 and 5 equiv,^16^ unlike our reactions.

**Scheme 6:**
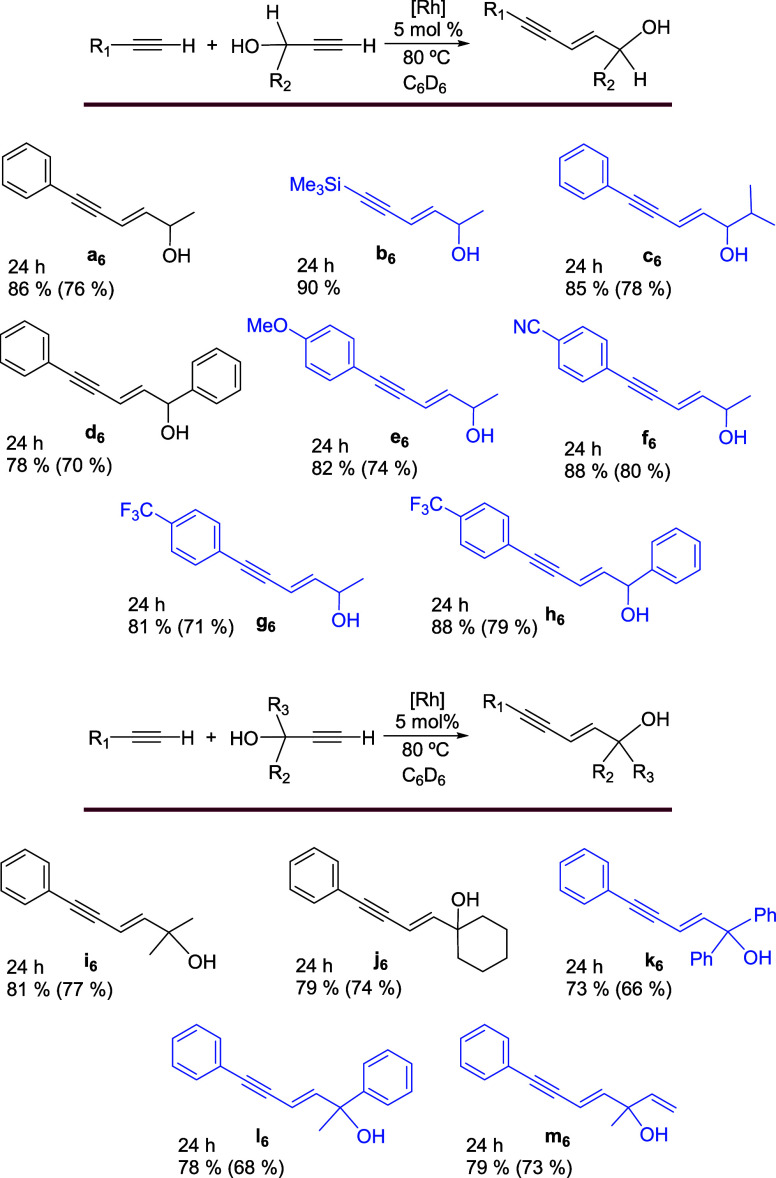
Cross-Coupling of Phenylacetylenes and α-Hydroxyacetylenes^a^

a Nonreported compounds in blue. Conditions: **1** (5 mg, 0.009 mmol), 0.19 mmol of each alkyne, and benzene-*d*_6_ (0.4 mL). Yields were determined by ^1^H NMR using dioxane as internal standard; isolated yields are given in parentheses.

Reaction crudes contain small amounts of homocoupling dimers. However, the (*E*)-5-phenyl-2-penten-4-yn-1-ols products were easily separated from these byproducts, by preparative thin-layer chromatography on silica gel, since their significantly different polarities. Thus, this catalysis is an efficient procedure of organic synthesis that allowed us to characterize five new (*E*)-5-phenyl-2-penten-4-yn-1-ols with a secondary alcohol function, three with a tertiary one, and **b**_**6**._ The new (*E*)-5-phenyl-2-penten-4-yn-1-ol derivatives include **c**_**6**_, **e**_**6**_ **–h**_**6**_, and **k**_**6**_ **–m**_**6**_. Compounds (*E*)-1,5-diphenyl-2-penten-4-yn-1-ol (**d**_**6**_) and (*E*)-6-phenyl-3-hexen-5-yn-2-ol (**a**_**6**_), which carry a secondary alcohol function, are known. The first of these is often used as a synthetic intermediate; it is generated by treatment of 5-phenyl-2-penten-4-ynoic acid ethyl ester with diisobutylaluminum hydride and is used immediately for subsequent reactions.^34^ The second, the methyl counterpart, was prepared as a mixture of *E* and *Z* stereoisomers, by acid-catalyzed rearrangement of 1-phenyl-4-hexen-1-yn-3-ol. The latter is obtained from the reaction of 2-buten-1-al with lithium phenylacetylide.^35^ However, a more appropriate procedure is the selective catalytic reduction of the carbonyl group of 6-phenyl-3-hexen-5-yn-2-one.^36^ Also known are the compounds (*E*)-2-methyl-6-phenyl-3-hexen-5-yn-2-ol (**i**_**6**_) and (*E*)-1-(4-phenyl-1-buten-3-yn-1-yl)-1-cyclohexanol (**j**_**6**_), which have a tertiary alcohol function. They were prepared as now, by rhodium-catalyzed cross-coupling between phenylacetylene and the corresponding alkynol, but in much lower yields, >40%.^9a,14b^

Scheme 7 rationalizes the formation of products of the type (*E*)-5-phenyl-2-penten-4-yn-1-ol, by cross-coupling between a phenylacetylene and a α-hydroxyacetylene, based on the elementary steps of Scheme 3. Because there are two different alkynes involved in the reaction, two different rhodium­(I)-acetylide species can be formed; **3** and an analogous α-hydroxyacetylide **D**. Oxidative addition of the C­(sp)–H bond of both alkynes to the metal center of these species should lead to the bis­(acetylide)-rhodium­(III)-hydride derivatives **4**, **G**, and **J**. The roles of the alkynes in the cross-coupling process are predetermined; α-hydroxyacetylene acts as a hydrogen acceptor, while phenylacetylene is the hydrogen donor. Therefore, the insertions of the C–C triple bond of α-hydroxyacetylene into the Rh–H bond of **4** and **J** are the only productive additions among the possible ones and define the C_1_ and C_2_ cycles responsible for the formation of the heterocoupling products. Such insertions provide intermediates **K** and **L**, which are related compounds to **5** and **6** but heteroleptic in nature. The addition of the Rh–H bond of **4** and **J** to the C–C triple bond of the α-hydroxyacetylene must be faster than the insertion of the C–C triple bond of phenylacetylene into the Rh–H bond of **4**, since the latter is the rate-determining step of homocoupling of phenylacetylene. Once **K** and **L** are formed, they should undergo reductive elimination of the cross-coupling product. Elimination at **K** would regenerate **3**, while elimination at **L** would produce **D**. They are the rate-determining step of the cycles C_1_ and C_2_ and, as previously mentioned, must also be faster than the rate-determining steps of both homocouplings; the above-mentioned insertion of the C–C triple bond of phenylacetylene in the Rh–H bond of **4** and the reductive elimination of (*E*)-enyne-diol from **H**. The ^31^P­{^1^H} NMR spectra of the solutions, in which the cross-coupling products are formed, recorded during the reactions are consistent with this. They show three doublets between 36 and 31 ppm (Figure S14); the region of the spectrum where **5** and **6** are observed. One of them corresponds to **H**,^37^ which is responsible for the formation of the observed small amounts of dimer from α-hydroxyacetylene homocoupling, whereas the other two are due to **K** and **L**. Thus, the homocoupling byproducts are generated in two different phases of the cross-coupling reaction. α-Hydroxyacetylene homocoupling is a competitive reaction with cross-coupling, while phenylacetylene homocoupling only occurs when cross-coupling is complete. As a consequence of the loss of a small amount of α-hydroxyacetylene in competitive homocoupling, a small amount of phenylacetylene remains, which undergoes dimerization. Consistently, the ^31^P­{^1^H} NMR spectra of solutions corresponding to reactions involving unsubstituted phenylacetylene show the presence of **3**, at the end of cross-coupling (Figure S14). DFT calculations on cycles C_1_ and C_2_ reproduce everything mentioned above.

**Scheme 7:**
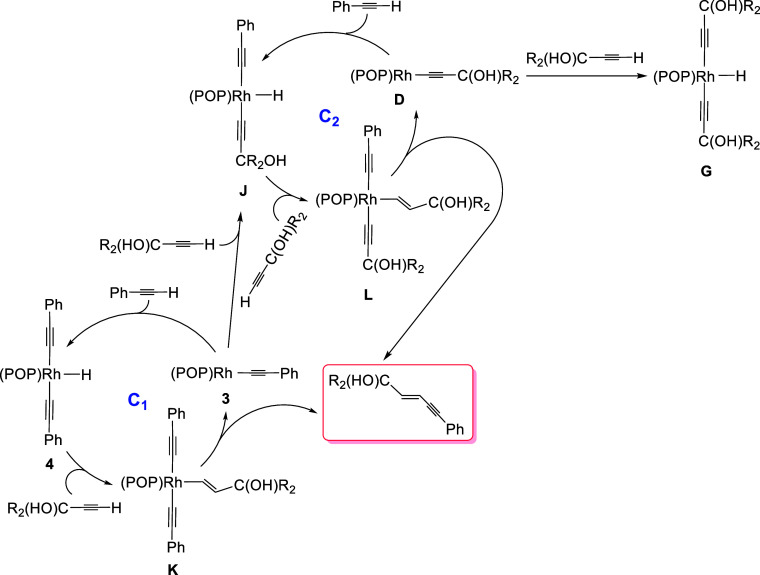
Plausible Mechanisms for the Formation of Cross-Coupling Products between a Phenylacetylene and an α-Hydroxyacetylene

Figure 6 shows the energy profile for the formation of **K**, by insertion of the C–C triple bond of the α-hydroxyacetylene into the R–H bond of **4**, and the release of the heterocoupling product, according to the C_1_ cycle. The insertion of the C–C triple bond of α-hydroxyacetylene in the Rh–H bond of **4** must overcome an activation energy of 21.0 kcal mol^–1^, similar to that observed for the same insertion in the Rh–H bond of the species bis­(α-hydroxyacetylide) **G**. The transition states of both insertions are also similar; the ether-diphosphine coordinates in a bidentate manner, with a P–Rh–P bite angle of 114.0°, whereas the hydride and the oxygen atom of α-hydroxyacetylene interact to be located 2.324 Å from each other. Reductive heterocoupling at **K** leads to **M**, which resembles **C** and **I**, but contains a coordinated (*E*)-phenylpentenynol. The activation energy of the coupling, 24.7 kcal mol^–1^, is higher than those calculated for the insertion and oxidative addition steps. Therefore, C–C bond formation is the rate-determining step of heterocoupling, as observed experimentally. Intermediate **M** dissociates the catalysis product to close the cycle with a low activation energy of 12.1 kcal mol^–1^.

**Figure 6:**
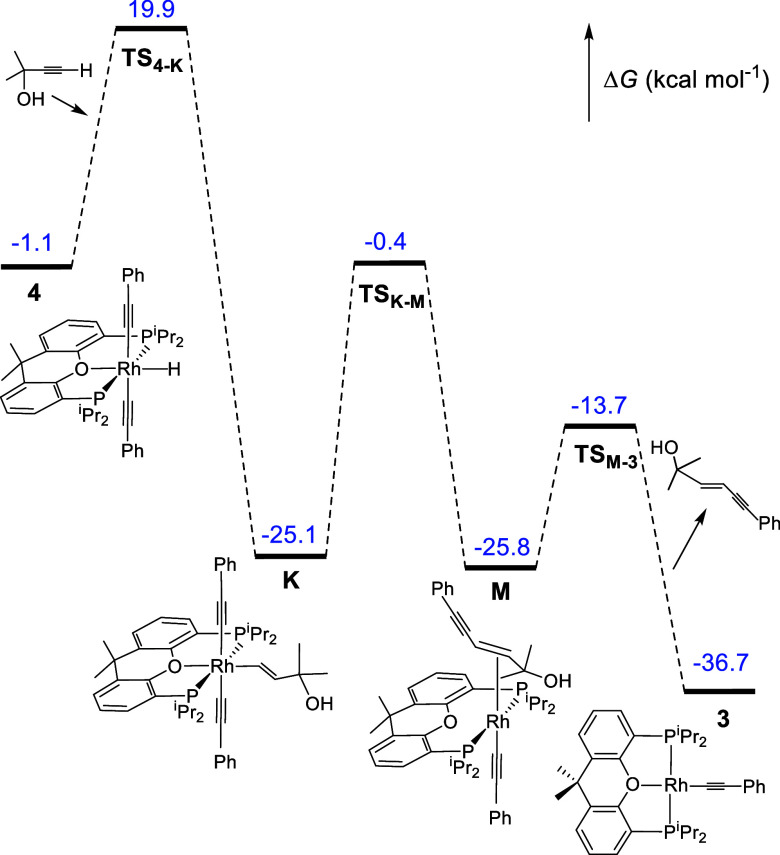
Computed energy profile (Δ*G*, in kcal mol^–1^) for the formation of **K**, by insertion of the C–C triple bond of the α-hydroxyacetylene into the R–H bond of **4**, and the release of the heterocoupling product, according to the C_1_ cycle.

The oxidative additions of the C­(sp)–H bond of the alkynes to **3** and **D** that afford the intermediate **J** of the C_2_ cycle (Figure S114) occur by the same three steps as the oxidative additions schematized in Figure 4. As before, the maximum barriers show values around 21 kcal mol^–1^, which correspond to the sliding of the metal center from the C–C triple bond toward the σ–C–H bond of the added alkyne. Figure 7 shows the energy profile for the formation of **L**, by insertion of the C–C triple bond of α-hydroxyacetylene into the Rh–H bond of **J**, and the release of the heterocoupling product from the latter. The profile for enyne-diol removal by homocoupling is also shown for comparison purposes. The insertion of the C–C triple bond of the α-hydroxyacetylene into the Rh–H bond of **J** has an activation energy of 20.4 kcal mol^–1^. This barrier is similar to the barriers of the functionalized alkyne insertions in the other hydride-rhodium­(III) intermediates. The reason is that all insertions have similar transition states. These barriers are also similar to those calculated for oxidative additions of the C­(sp)–H bond of alkynes to Rh­(I)-acetylide species. However, the reductive C–C bond formation in **L** shows an activation energy of 25.0 kcal mol^–1^, which is higher than the barriers of the oxidative addition and insertion reactions of the cycle. This value is similar to that calculated for the same reductive elimination in **K** but slightly lower than that calculated for the reductive homocoupling in the same intermediate, 26.6 kcal mol^–1^. Therefore, in a consistent manner with the experimental findings, we can say from the DFT calculations: (i) the reductive C–C elimination in **L** is the rate-determining step of the C_2_ cycle; (ii) the rate-determining step of cycles C_1_ and C_2_ is the same; (iii) it also has the same value; and (iv) this value is lower than the activation energy for the C–C reductive elimination of the enyne-diol homocoupling product. The reductive C–C couplings in **L** lead to intermediates **N** and **O**, which dissociate the catalysis products, overcoming low barriers.

**Figure 7:**
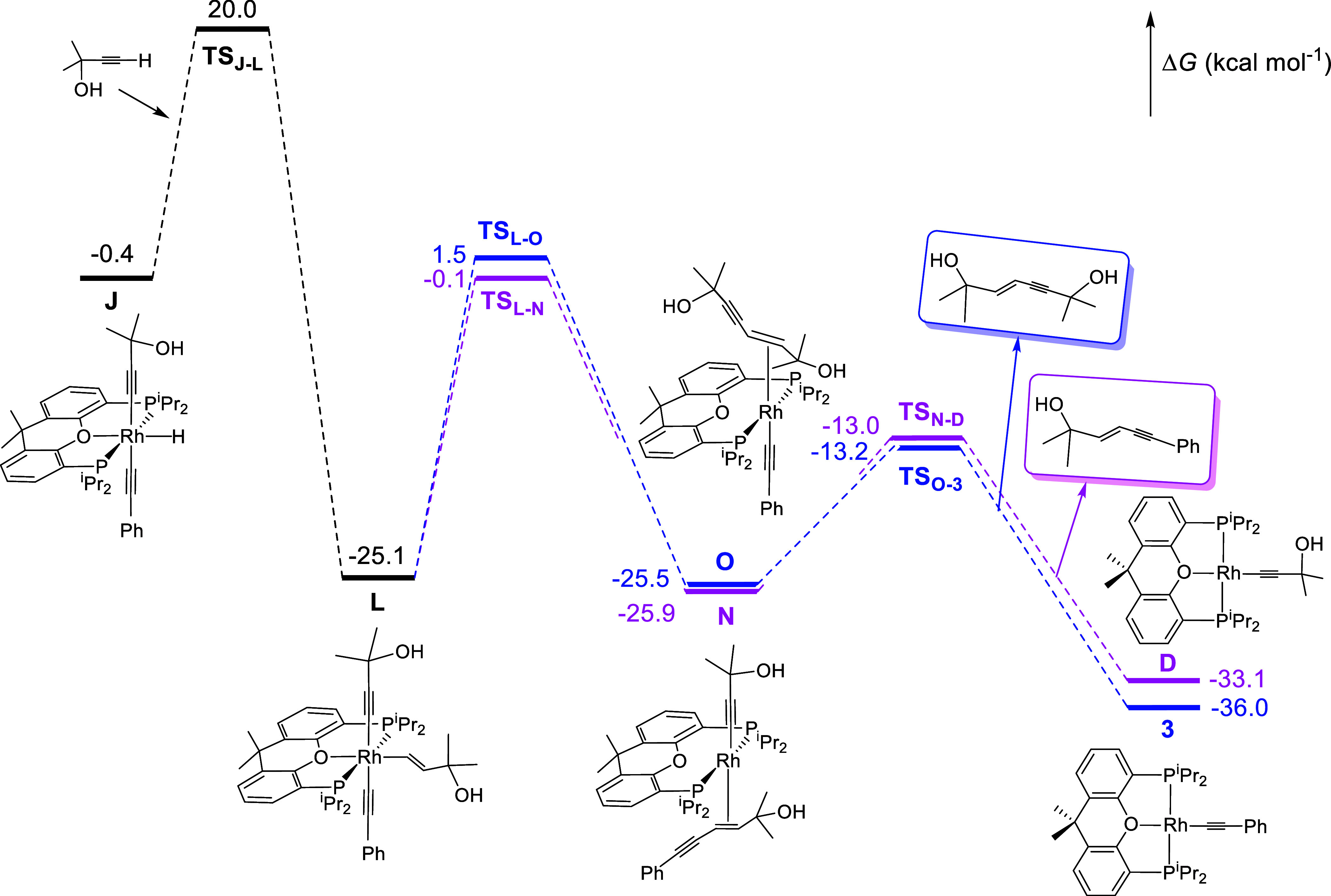
Energy profile (Δ*G*, in kcal mol^–1^) for the formation of **L** and the release of the heterocoupling product (in purple) or the enyne-diol (in blue) from it.

### Ethisterone Reactions

Ethisterone is a good example of a highly functionalized aliphatic α-hydroxyacetylene with a tertiary alcohol character. Its structural modification is of interest, given the important pharmaceutical uses of progestin.^38^ It undergoes some organic transformations typical of α-hydroxyacetylenes^39^ and has been shown to bind to transition metals, forming metal–carbon bonds.^40^ Zhou, Yin, and co-workers reported a Cu-catalyzed dehydrogenative ethisterone-phenylacetylene cross-coupling, giving 1,3-diyne.^41^ To demonstrate the remarkable potential of complex **1** as a precursor to homo- and cross-coupling reactions involving α-hydroxyacetylenes, we have also carried out ethisterone homocoupling and ethisterone-phenylacetylene cross-coupling (Scheme 8).

**Scheme 8:**
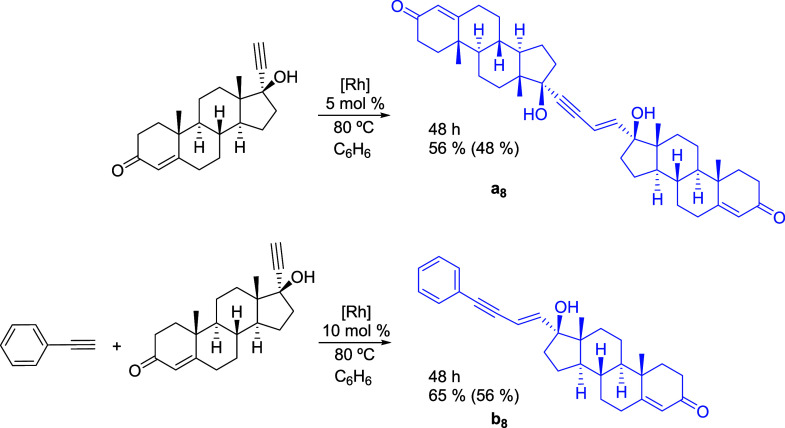
Ethisterone Homocoupling and Ethisterone-Phenylacetylene Cross-Coupling^a^

a Conditions homocoupling: **1** (5 mg, 0.009 mmol), 0.20 mmol of ethisterone, and benzene (2 mL). Heterocoupling: **1** (5 mg, 0.009 mmol), 0.10 mmol of ethisterone, 0.10 mmol phenylacetylene, and benzene (2 mL). Yields were determined by ^1^H NMR with respect to starting alkyne; isolated yields are given in parentheses.

Complex **1** catalyzes the head-to-head dimerization of ethisterone to selectively give the product (*E*)-enyne-diol **a**_**8**_. This dimer was formed in 56% yield, after 48 h, using 5 mol % of precursor with regard to ethisterone, in benzene, at 80 °C. The cross-coupling reaction was carried out with equimolar concentrations of phenylacetylene and ethisterone, using 10 mol % of complex **1** with respect to each alkyne. In benzene, at 80 °C, the asymmetrical (*E*)-enyn-ol **b**_**8**_ was formed with a yield of 65%, after 48 h. The products of both reactions were separated from the crude by column chromatography, isolated in 48% and 56% yield, and characterized by ^1^H and ^13^C­{^1^H} NMR spectroscopy and high-resolution mass spectrometry.

## Concluding Remarks

This study has revealed that the square-planar rhodium­(I) monohydride complex RhH­{κ^3^-*P*,*O*,*P*-[xant­(P^i^Pr_2_)_2_]} is an efficient catalyst precursor for the head-to-head homocoupling of terminal alkynes and the head-to-head cross-coupling between phenylacetylenes and α-hydroxyacetylenes. Homocoupling reactions give (*E*)-enyne products, while cross-coupling reactions lead to (*E*)-enyn-ols of the type (*E*)-5-phenyl-2-penten-4-yn-1-ol.

Homocoupling reactions occur as follows: the monohydride complex generates rhodium­(I)-acetylide species, which are responsible for catalysis. These square planar derivatives, a different one for each alkyne, oxidatively add the C­(sp)-H bond of the substrates to produce bis­(acetylide)-rhodium­(III)-hydride intermediates, which undergo *anti*-Markovnikov insertion of the C–C triple bond of a new alkyne molecule in the Rh–H bond. The resulting {(*E*)-alkenyl}-rhodium­(III)-bis­(acetylide) complexes reductively remove the (*E*)-enyne products, regenerating the rhodium­(I)-acetylide catalysts. The rate-determining step of the coupling depends on the nature of the alkyne, being the insertion of the C–C triple bond at the Rh–H bond for phenylacetylenes and the reductive elimination of the (*E*)-enyne-diol product for α-hydroxyacetylenes.

Cross-coupling reactions take place through two cycles similar to the cycle that produces homocoupling. They start from the rhodium­(I)-acetylide species that generate the two different alkynes involved in each reaction. For both cycles, the rate-determining step is the reductive elimination of the (*E*)-5-phenyl-2-penten-4-yn-1-ol-type products.

The observation of metallic intermediates in organic reactions catalyzed by metals allows for establishing the catalytic cycle and understanding the origin of selectivity. Starting from this premise, we have discovered the mechanism of the homocoupling of phenylacetylenes and α-hydroxyacetylenes, promoted by a highly efficient catalyst precursor, including the rate-determining steps of both dimerizations. DFT calculations reproduce the experimental findings and corroborate the conclusions provided by them. The information obtained in this way has led us to develop cross-coupling reactions between phenylacetylenes and one of the widest ranges of α-hydroxyacetylenes used to date. We can say that there is now a rhodium catalyst precursor for preparing in almost quantitative yield reported and unreported compounds of the type (*E*)-5-phenyl-2-penten-4-yn-1-ol, by direct cross-coupling between phenylacetylenes and α-hydroxyacetylenes.

## Experimental Section

### General Information

All reactions were carried out with the exclusion of air using Schlenk-tube techniques or in a glovebox. Instrumental methods and X-ray details are given in the Supporting Information. In the NMR spectra (Figures S1–S113), the chemical shifts (in ppm) were referenced to residual solvent peaks (^1^H, ^13^C­{^1^H}) or external 85% H_3_PO_4_ (^31^P­{^1^H}), while *J* and *N* (*N* = *J*_P–H_ + *J*_P′–H_ for ^1^H and *N* = *J*_P–C_ + *J*_P′–C_ for ^13^C­{^1^H}) were given in hertz. RhH­{κ^3^-*P*,*O*,*P*-[xant­(P^i^Pr_2_)_2_]} (**1**)^30^ was prepared according to the reported procedure.

### Spectroscopic Detection of Rh­{(*E*)–CH=CHPh}­{κ^3^-*P*,*O*,*P*-[xant­(P^i^Pr_2_)_2_]} (2)

To a NMR tube containing **1** (10 mg, 0.018 mmol), a solution of phenylacetylene in benzene-*d*_6_ (0.4 mL, 64 mM; 1.4 equiv) was added. The ^1^H and ^31^P­{^1^H} NMR spectra recorded after 5 min at room temperature show the presence of complexes **2**, **3**, and styrene in a 1:0.2:0.2 ratio. Distinctive spectroscopic data for complex **2**: ^1^H NMR (300 MHz, benzene-*d*_6_, 298 K): δ 9.90 (ddt, ^3^ *J*_H–H_ = 17, ^2^ *J*_H–Rh_ = 3, ^3^ *J*_H–P_ = 6, Rh–CH=). ^31^P­{^1^H} NMR (121.49 MHz, benzene-*d*_6_, 298 K): δ 38.6 (d, ^1^ *J*_P–Rh_ = 176 Hz).

### Preparation of Rh­(C≡CPh)­{κ^3^-*P*,*O*,*P*-[xant­(P^i^Pr_2_)_2_]} (**3**)

A solution of **1** (100 mg, 0.18 mmol) in pentane (3 mL) was treated with phenylacetylene (50 μL, 0.46 mmol) and then stirred overnight at room temperature. After that time, it was evaporated to dryness, getting a brown residue. The addition of pentane (4 mL) afforded a white solid that was washed with pentane (2 × 2 mL) and dried in vacuo. Yield: 109 mg (92%). Anal. Calcd for C_35_H_45_OP_2_Rh (%): C, 65.01; H, 7.01. Found: C, 64.69; H, 7.18. HRMS (electrospray, *m*/*z*): calcd for C_35_H_46_OP_2_Rh [M + H]^+^, 647.2073; found, 647.2061. IR (cm^–1^): ν­(C≡C) 2088 (m), ν­(C–O–C) 1208 (m). ^1^H NMR (300.13 MHz, benzene-*d*_6_, 298 K): δ 7.72 (dd, ^3^ *J*_H–H_ = 8.2, ^4^ *J*_H–H_ = 1.2, 2H, Ph), 7.28–7.19 (4H, 2H CH-arom POP + 2H Ph), 7.04–6.97 (3H, 2H CH-arom POP + 1H Ph), 6.85 (t, ^3^ *J*_H–H_ = 7.5, 2H, CH-arom POP), 2.43 (m, 4H, PC*H*(CH_3_), 1.64 (dvt, ^3^ *J*_H–H_ = 7.2, *N* = 16.5, 12H, PCH­(C*H*_3_)_2_), 1.20 (s, 6H, CH_3_), 1.18 (dvt, ^3^ *J*_H–H_ = 7.2, *N* = 14.8, 12H, PCH­(C*H*_3_)_2_). ^13^C­{^1^H}-apt NMR (75.48 MHz, benzene-*d*_6_, 298 K): δ 157.1 (vt, *N* = 16.4, C-arom POP), 132.4 (s, C-arom POP), 131.2 (s, CH-arom POP), 131.1 (m, C Ph), 130.9 (s, CH Ph), 128.2 (s, CH Ph), 127.9 (s, CH-arom POP), 126.0 (vt, *N* = 15.9, C-arom POP), 124.8 (s, CH-arom POP), 123.8 (dt, ^2^ *J*_C–Rh_ = 20.1, ^3^ *J*_C–P_ = 4.2, Rh–C≡*C*Ph), 122.9 (s, CH Ph), 110.0 (dt, ^1^ *J*_C–Rh_ = 62.0, ^2^ *J*_C–P_ = 19.2, Rh–*C*≡CPh), 34.1 (s, *C*(CH_3_)_2_), 32.5 (s, C­(*C*H_3_)_2_), 27.2 (dvt, ^3^ *J*_C–Rh_ = 2.3, N = 10.0, P*C*H­(CH_3_)_2_), 20.0 (vt, *N* = 8.0, PCH­(*C*H_3_)_2_), 19.6 (s, PCH­(*C*H_3_)_2_). ^31^P­{^1^H} NMR (121.49 MHz, benzene-*d*_6_, 298 K): δ 45.5 (d, ^1^ *J*_Rh–P_ = 152).

### Reaction of Rh­(C≡CPh)­{κ^3^-*P,O,P*-[xant­(P^i^Pr_2_)_2_]} (**3**) with Phenylacetylene. Spectroscopic Detection of RhH­(C≡CPh)_2_{κ^3^-*P*,*O*,*P*-[xant­(P^i^Pr_2_)_2_]} (**4**) and Rh­{(*E*)–CH=CHPh}­(C≡CPh)_2_{κ^3^-*P*,*O*,*P*-[xant­(P^i^Pr_2_)_2_]} (**5**)

A NMR tube containing a solution of **3** (15 mg, 0.02 mmol) in benzene-*d*_6_ (0.4 mL) was treated with phenylacetylene (25 μL, 0.23 mmol). The ^1^H and ^31^P­{^1^H} NMR spectra recorded after 10–20 min at room temperature, show the presence of complexes **3**, **4**, and **5** in an approximate 25:67:8 molar ratio. Distinctive spectroscopic data for complex **4**: ^1^H NMR (300 MHz, benzene-*d*_6_, 298 K): δ −18.84 (dt, ^1^ *J*_H–Rh_ = 32.2, ^2^ *J*_H–P_ = 12.0, RhH). ^31^P­{^1^H} NMR (121.49 MHz, benzene-*d*_6_, 298 K): δ 54.4 (d, ^1^ *J*_P–Rh_ = 100 Hz). Distinctive spectroscopic data for complex **5**: ^1^H NMR (300 MHz, benzene-*d*_6_, 298 K): δ 8.58 (ddt, ^3^ *J*_H–H_ = 14.3, ^3^ *J*_H–P_ = 2.1, ^2^ *J*_H–Rh_ = 1.2, Rh–CH=). ^31^P­{^1^H} NMR (121.49 MHz, benzene-*d*_6_, 298 K): δ 32.9 (d, ^1^ *J*_P–Rh_ = 100 Hz). Complex **5** was generated as an almost exclusive organometallic species under the following conditions: **3** (15 mg, 0.02 mmol), phenylacetylene (38 μL, 0.35 mmol), benzene-*d*_6_ (0.4 mL), 2 h, and room temperature.

### Preparation of Rh­{(*E*)–CH=CHC­(OH)­Ph_2_}­{C≡CC­(OH)­Ph_2_}_2_{κ^3^-*P,O,P*-[xant­(P^i^Pr_2_)_2_]} (**6**)

A solution of **1** (100 mg, 0.18 mmol) in benzene (3 mL) was treated with 1,1-diphenyl-2-propyn-1-ol (152 mg, 0.73 mmol) and then stirred at room temperature for 1 h. After that time, the solution was evaporated to dryness, to give a brown residue. The addition of pentane (4 mL) afforded a white solid that was washed with pentane (2 × 2 mL) and dried in vacuo. Yield: 175 mg (82%). Anal. Calcd for C_72_H_75_O_4_P_2_Rh (%): C, 73.96; H, 6.47. Found: C, 73.94; H, 6.50. HRMS (electrospray, *m*/*z*): calcd for C_72_H_75_NaO_4_P_2_Rh [M + Na]^+^, 1191.4088; found, 1191.4127. IR (cm^–1^): ν­(O–H) 3607 (w), ν­(C≡C) 2094 (w), ν­(C–O–C) 1244 (m), ν­(C–O) 1183 and 1150 (m). ^1^H NMR (500.13 MHz, benzene-*d*_6_, 298 K): δ 7.78 (d, ^3^ *J*_H–H_ = 8.4, 4H, Ph) δ 7.52 (d, ^3^ *J*_H–H_ = 8.4, 8H, Ph), 7.35 (d, ^3^ *J*_H–H_ = 14.0, 1H, CH=CH), 7.27 (t, ^3^ *J*_H–H_ = 7.7, 4H, Ph), 7.18–6.92 (19H, 4H CH-arom POP, 1H CH = CH, 14H Ph), 6.86 (t, ^3^ *J*_H–H_ = 7.8, 2H, CH-arom POP), 2.72 (m, 4H, PC*H*(CH_3_)_2_), 2.26 (s, 1H, OH), 2.13 (s, 2H, OH), 1.20 (s, 6H, CH_3_), 1.07 (dvt, ^3^ *J*_H–H_ = 7.6, *N* = 12.3, 12H, PCH­(C*H*_3_)_2_), 1.05 (dvt, ^3^ *J*_H–H_ = 7.6, *N* = 12.3, 12H, PCH­(C*H*_3_)_2_). ^13^C­{^1^H}-apt NMR (75.48 MHz, benzene-*d*_6_, 298 K): δ 156.7 (vt, *N* = 11.0, *C*-arom POP), 148.9, 148.6 (s, C Ph), 139.0 (t, ^3^ *J*_C–P_ = 3.7, Rh–CH=*C*H), 133.5 (vt, *N* = 5.1, C-arom POP), 130.2 (s, CH-arom POP), 129.7 (dt, ^1^ *J*_C–Rh_ = 34.1, ^2^ *J*_C–P_ = 9.9, Rh–*C*H=CH), 128.0, 127.8, 127.8 (s, CH Ph), 127.1, 126.7, 126.4 (s, CH-arom POP), 124.6 (vt, *N* = 5.3, CH-arom POP), 123.3 (vt, *N* = 14.2, C-arom POP), 113.4 (dt, ^1^ *J*_C–Rh_ = 38.9, ^2^ *J*_C–P_ = 15.5, Rh–*C* ≡ CC­(OH)­Ph_2_), 109.8 (dt, ^2^ *J*_C–Rh_ = 3.0, ^3^ *J*_C–P_ = 0.8, Rh–C≡*C*C­(OH)­Ph_2_), 80.4 (dt, ^3^ *J*_C–Rh_ = 3.0, ^4^ *J*_C–P_ = 0.8, *C*(OH)­Ph_2_), 75.5 (s, *C*(OH)­Ph_2_), 35.0 (s, C­(*C*H_3_)_2_), 29.0 (s, *C*(CH_3_)_3_), 25.8 (vt, *N* = 24.9, P*C*H­(CH_3_)_2_), 19.0, 18.9 (both s, PCH­(*C*H_3_)_2_). ^31^P­{^1^H} NMR (202.46 MHz, benzene-*d*_6_, 298 K): δ 34.6 (d, ^1^ *J*_Rh–P_ = 101.5).

### Kinetic Analysis of the Reductive Elimination of (*E*)-1,1,6,6-Tetraphenyl-2-hexen-4-yne-1,6-diol from Rh­{(*E*)–CH=CHC­(OH)­Ph_2_}­{C≡CC­(OH)­Ph_2_}_2_{κ^3^-*P,O,P*-[xant­(P^i^Pr_2_)_2_]} (**6**)

The experimental procedure is described for a particular case, but the same method was used in all experiments. In the glovebox, a NMR tube was charged with a solution of **6** (10 mg, 0.008 mmol) in benzene-*d*_6_ (0.4 mL), and a sealed capillary tube filled with a solution of the internal standard (PPh_3_) in benzene-*d*_6_ was placed in the NMR tube. The tube was immediately introduced into a NMR probe preheated at the desired temperature (353, 343, 333, and 323 K), and the reaction was monitored by ^31^P­{^1^H} NMR at different intervals of time. With these experiments and using eq 1 the rate constants *k* were calculated

1 $$\ln\frac{\left[6\right]}{{\left[6\right]}_{0}}=-kt$$

### Kinetic Analysis of the Dimerization of 1,1-Diphenyl-2-propyn-1-ol Catalyzed by Rh­{(*E*)–CH=CHC­(OH)­Ph_2_}­{C≡CC­(OH)­Ph_2_}_2_{κ^3^-*P*,*O*,*P*-[xant­(P^i^Pr_2_)_2_]} (**6**)

The experimental procedure is described for a particular case, but the same method was used in all experiments. In the glovebox, a screw-cap NMR tube was charged with **6** (10 mg, 0.008 mmol), 1,1-diphenyl-2-propyn-1-ol (71 mg, 0.34 mmol), and a sealed capillary tube filled with a solution of the internal standard (dioxane) in benzene-*d*_6_ was placed in the NMR tube. In the NMR room, 0.4 mL of benzene-*d*_6_ was added to that tube through the septum of the screw-cap, the tube was immediately introduced into a NMR probe preheated at the desired temperature (353, 343, 333, and 323 K), and the reaction was monitored by ^1^H NMR at different intervals of time. With these experiments and using eq 2 the rate constants *k* were calculated

2 $$\frac{1}{{\left[alkyne\right]}_{t}}=\frac{1}{{\left[alkyne\right]}_{0}}+2k_{obs}t$$

where

$$k_{obs}=k\left[catalyst\right]$$

### General Procedure for Homocoupling Reactions

All experiments were performed in duplicate, monitored by ^1^H NMR spectroscopy, and prepared in a glovebox. The procedure used in all cases was as follows: a NMR tube was loaded with a solution of **1** (5 mg, 0.009 mmol) in benzene-*d*_6_ (0.4 mL). Subsequently, a sealed capillary tube containing a solution of the internal standard (dioxane or 1,1,2,2-tetrachloroethane) in benzene-*d*_6_ was introduced, and the corresponding alkyne (0.38 mmol) was added. The tube was then closed with a cap, removed from the glovebox, and placed in a thermostatic bath at 80 °C. After completion of the reaction, the solvent was removed, and diethyl ether (phenylacetylenes) or ethyl acetate (alkynols) was added to the crude product. The solution was passed through a silica pad and dried under a vacuum. The products were characterized by ^1^H and ^13^C­{^1^H} NMR spectroscopies and, in addition, those nonreported by HR-MS.

### General Procedure for the Cross-Coupling Reactions

The experimental procedure was similar to that described for the homocoupling reactions, but using 0.19 mmol of each alkyne. After completion of the reaction, the crude products were purified by preparative thin layer chromatography eluting with pentane/ethyl acetate 9:1 (2:1 for **e**_**6**_ and **f**_**6**_). The products were characterized by ^1^H and ^13^C­{^1^H} NMR spectroscopies and, in addition, those nonreported by HR-MS.

### Homocoupling of Ethisterone

Inside the glovebox, ethisterone (63 mg, 0.20 mmol) was added to a solution of **1** (5 mg, 0.009 mmol) in benzene (2 mL) and placed in an Ace pressure tube. The tube was taken out of the glovebox and heated at 80 °C for 48 h. After this time, the solvent was evaporated under reduced pressure to afford a crude reaction mixture, which was dissolved in a minimal amount of ethyl acetate and purified by column chromatography on silica eluting with ethyl acetate/hexane (1:9 to 9:1). The product (**a**_**8**_) was characterized by ^1^H and ^13^C­{^1^H} NMR spectroscopies and by HR-MS.

### Cross-Coupling of Ethisterone

Inside the glovebox, ethisterone (34 mg, 0.11 mmol) and phenylacetylene (12, μL, 0.11 mmol) were added to a stirred solution of **1** (5 mg, 0.009 mmol) in benzene (2 mL) and placed in an Ace pressure tube. The tube was taken out of the glovebox and heated at 80 °C for 48 h. After this time, the solvent was evaporated under reduced pressure to afford a crude reaction mixture, which was dissolved in a minimal amount of ethyl acetate and purified by column chromatography on silica eluting with ethyl acetate/hexane (1:9 to 9:1). The product (**b**_**8**_) was characterized by ^1^H and ^13^C­{^1^H} NMR spectroscopies and by HR-MS.

## Supplementary Material







## Notes

General information for the Experimental Section, characterization data of the coupling products, structural analysis, computational details, and NMR spectra (PDF)
Cartesian coordinates of calculated structures (XYZ)
CIF file of complex **6** (CIF)
