# Supplementary material for: Homo- and Cross-Coupling of Phenylacetylenes and α‑Hydroxyacetylenes Catalyzed by a Square-Planar Rhodium Monohydride
Source: ACS Catal. 2024 May 14;14(11):8389–404. doi: 10.1021/acscatal.4c00264 (PMC12582320; doi:10.1021/acscatal.4c00264)
Supplement: Supplementary file 1 [file cs4c00264_si_001.pdf]

SUPPORTING INFORMATION

Homo- and Cross-Coupling of Phenylacetylenes and α-Hydroxyacetylenes Catalyzed by a Square-Planar Rhodium Monohydride

Laura A. de las Heras, Miguel A. Esteruelas,* Montserrat Oliván, and Enrique Oñate

Departamento de Química Inorgánica – Instituto de Síntesis Química y Catálisis Homogénea (ISQCH) – Centro de Innovación en Química Avanzada (ORFEO-CINQA), Universidad de Zaragoza – CSIC, 50009 Zaragoza, Spain

* e-mail: maester@unizar.es

Contents:

- General Information	S2
- Characterization Data of Homo-Coupling Products	S2
- Characterization Data of Cross-Coupling Products	S7
- Ethisterone Homo-Coupling and Ethisterone-Phenylacetylene Cross-Coupling	S10
- NMR spectra	S12
- Structural Analysis of Complex **6**	S69
- Computational Details	S70
- References	S74

• General Information

All reactions were carried out with exclusion of air using Schlenk-tube techniques or in a drybox. Pentane and toluene were obtained oxygen- and water-free from an MBraun solvent purification apparatus. Alkynes (except the solid ones, that were used as received) were distillated in a Kugelrohr glass over prior to use. ^{1}H, ^{13}C{^{1}H}, and ^{31}P{^{1}H} NMR spectra were recorded on Bruker 300 ARX, Bruker Avance 300 MHz, Bruker Avance 400 MHz, or Bruker Avance 500 MHz instruments. Chemical shifts (expressed in ppm) are referenced to residual solvent peaks (^{1}H, ^{13}C{^{1}H}) or external 85% H_3PO_4 (^{31}P{^{1}H}). Coupling constant J and N ($N = J_{P\text{-}H} + J_{P'\text{-}H}$ for ^{1}H and $N = J_{P\text{-}C} + J_{P'\text{-}C}$ for ^{13}C{^{1}H}) are given in hertz. Attenuated total reflection infrared spectra (ATR-IR) of solid samples were run on a PerkinElmer Spectrum 100 FT-IR spectrometer. C, H, and N analyses were carried out in a PerkinElmer 2400 CHNS/O analyzer. High-resolution electrospray or atmospheric-pressure chemical ionization (APCI) mass spectra were acquired using a MicroTOF-Q hybrid quadrupole time-of-flight spectrometer (Bruker Daltonics, Bremen, Germany). RhH{κ^3-P,O,P-[xant(P^{i}Pr$_2$)$_2$]} (**1**)[1] was prepared by the published method.

• Characterization Data of Homo-Coupling Products

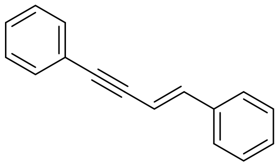

a4.[2] ^{1}H NMR (300.13 MHz, $CDCl_3$, 298 K): δ 7.51-7.23 (10H, Ph), 7.04 (d, $^{3}J_{H\text{-}H}$ = 16.2, 1H, =CH), 6.38 (d, $^{3}J_{H\text{-}H}$ = 16.2, 1H, =CH). ^{13}C{^{1}H}-apt NMR (75.48 MHz, $CDCl_3$, 298 K): δ 141.4 (s, =CH), 136.5 (s, C Ph), 131.7, 128.9, 128.8, 128.5, 128.3, 126.47 (all s, CH Ph), 123.6 (s, C Ph), 108.3 (s, =CH), 91.9, 89.1 (both s, C≡C).

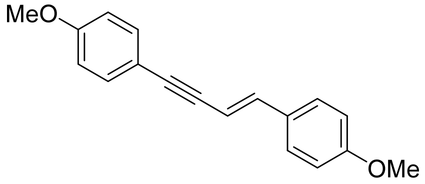

b4.[2] ^{1}H NMR (300.13 MHz, $CDCl_3$, 298 K): δ 7.45-7.32 (4H, Ph), 6.96 (d, $^{3}J_{H\text{-}H}$ = 16.2, 1H, =CH), 7.91-6.81 (4H, Ph), 6.24 (d, $^{3}J_{H\text{-}H}$ = 16.2, 1H, =CH), 3.83, 3.82 (both s, 3H, CH_3). ^{13}C{^{1}H}-apt NMR (75.48 MHz, $CDCl_3$, 298 K): δ 160.1, 159.6 (both s, C, Ph), 140.2 (s, =CH), 133.0 (s, CH Ph), 129.5 (s, C Ph), 127.7 (s, CH Ph), 115.9 (s, C Ph), 114.3, 114.1 (both s, CH Ph), 106.1 (s, =CH), 91.2 (s, C≡C), 88.1 (s, C≡C), 55.5, 55.4 (both s, CH_3).

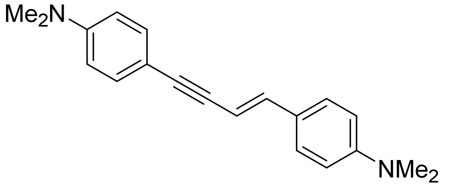

c4.[2] ^{1}H NMR (300.13 MHz, C_6D_6, 298 K): δ 7.36-7.25 (4H, CH Ph), 6.88 (d, $^3J_{H\text{-}H}$ = 16.1, 1H, =CH), 6.64 (t, $^3J_{H\text{-}H}$ = 9.6, 4H, CH Ph), 6.16 (d, $^3J_{H\text{-}H}$ = 16.1, 1H, =CH), 2.96 (s, 12H, CH_3). ^{13}C{^{1}H}-apt NMR (75.48 MHz, C_6D_6, 298 K): δ 150.6, 149.9 (both s, C Ph), 139.9 (s, =CH), 132.6, 127.4 (both s, CH Ph), 125.3 (s, CH Ph), 112.4 (s, =CH), 112.1 (s, CH Ph), 111.0 (s, CH Ph), 91.7, 88.0 (both s, C≡C), 40.5. 40.4 (both s, CH_3).

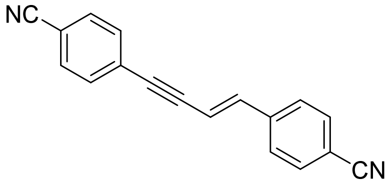

d4.[2] ^{1}H NMR (300.13 MHz, $CDCl_3$, 298 K): δ 7.69-7.46 (8H, Ph), 7.08 (d, $^3J_{H\text{-}H}$ = 16.3, 1H, =CH), 6.48 (d, $^3J_{H\text{-}H}$ = 16.2, 1H, =CH). ^{13}C{^{1}H}-apt NMR (75.48 MHz, $CDCl_3$, 298 K): δ 140.8 (s, =CH), 140.2 (s, C Ph), 132.7, 132.2 (both s, CH Ph), 127. 9 (s, C Ph), 127.0 (s, CH Ph), 118.7, 118.5 (both s, CN), 112.3, 112.0 (both s, C Ph), 111.3 (s, =CH), 92.3, 92.1 (both s, C≡C).

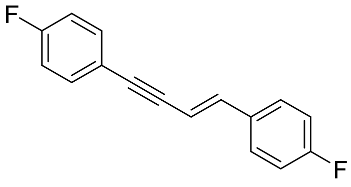

e4.[3] ^{1}H NMR (300.13 MHz, $CDCl_3$, 298 K): δ 7.54-7.32 (4H, Ph), 7.12-6.95 (5H, 1 =CH + 4 Ph), 6.28 (d, $^3J_{H\text{-}H}$ = 16.2, 1H, =CH). ^{13}C{^{1}H}-apt NMR (75.48 MHz, $CDCl_3$, 298 K): δ 163.1 (d, $^1J_{C\text{-}F}$ = 249.0, C-F), 162.6 (d, $^1J_{C\text{-}F}$ = 249.8, C-F), 140.2 (s, =CH), 133.5 (d, $^3J_{C\text{-}F}$ = 8.3, CH Ph), 132.6 (d, $^4J_{C\text{-}F}$ = 3.4, C Ph), 128.1 (d, $^3J_{C\text{-}F}$ = 8.1, CH Ph), 119.6 (d, $^4J_{C\text{-}F}$ = 3.4, C Ph), 115.9 (d, $^2J_{C\text{-}F}$ = 21.8, CH Ph), 115.8 (d, $^2J_{C\text{-}F}$ = 22.1, CH Ph), 107.9 (s, =CH), 90.7 (s, C≡C), 88.5 (s, C≡C).

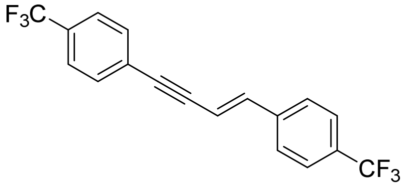

f4.[2] ^{1}H NMR (300.13 MHz, $CDCl_3$, 298 K): δ 7.57 (m, 8H, Ph), 7.10 (d, $^3J_{H\text{-}H}$ = 16.3, 1H, =CH), 6.47 (d, $^3J_{H\text{-}H}$ = 16.3, 1H, =CH). ^{13}C{^{1}H}-apt NMR (75.48 MHz, $CDCl_3$, 298 K): δ 140.8 (s, =CH), 139.5 (q, $^5J_{C\text{-}F}$ = 1.3, C Ph), 131.9 (s, CH Ph), 130.7 (q, $^2J_{C\text{-}F}$ = 32.4, C Ph), 130.3 (q, $^2J_{C\text{-}F}$ = 32.7, C Ph), 127.0 (q, $^5J_{C\text{-}F}$ = 1.3, C Ph), 126.7 (s, CH Ph), 125.9 (q, $^3J_{C\text{-}F}$ = 3.8, CH Ph), 125.5 (q, $^3J_{C\text{-}F}$ = 3.8, CH Ph), 124.1 (q, $^1J_{C\text{-}F}$ = 272.1, C CF_3), 124.0 (q, $^1J_{C\text{-}F}$ = 272.3, C CF_3), 110.4 (s, =CH), 91.7 (s, C≡C), 90.7 (s, C≡C).

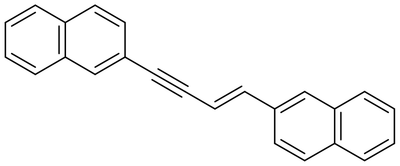

g$_4$.[4] ^{1}H NMR (300.13 MHz, $CDCl_3$, 298 K): δ 7.90-7.77 (7H, CH naph), 7.60-7.42 (7H, CH naph), 7.26 (d, $^3J_{H\text{-}H}$ = 16.2, 1H, =CH), 6.57 (d, $^3J_{H\text{-}H}$ = 16.2, 1H, =CH).$^{13}C\{^1H\}$-apt NMR (75.48 MHz, $CDCl_3$, 298 K): δ 141.6 (s, =CH), 134.0, 133.7, 133.6, 133.2, 133.0 (s, C, naph), 131.5, 128.6, 128.5, 128.4, 128.2, 128.0, 127.9, 127.8, 127.1, 126.8, 126.7, 126.7, 126.6, 122.9 (all s, CH naph), 120.9 (s, C, naph), 108.6 (s, =CH), 92.8 (s, C≡C), 89.7 (s, C≡C).

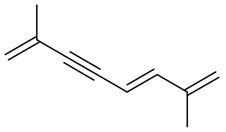

h$_4$.[5] ^{1}H NMR (300.13 MHz, C_6D_6, 298 K): δ 6.70 (d, $^3J_{H\text{-}H}$ = 16.0, 1H, =CH), 5.65 (d, $^3J_{H\text{-}H}$ = 16.0, 1H, =CH), 5.39 (m, 1H, CH_2), 5.07 (m, 1H, CH_2) 4.86 - 4.80 (m, 2H, CH_2), 1.82 (m, 3H, CH_3), 1.53 (m, 3H, CH_3). $^{13}C\{^1H\}$-apt NMR (75.48 MHz, C_6D_6, 298 K): δ 144.2 (s, =CH), 141.7 (s, C), 127.5 (s, C), 121.7, 118.9 (both s, =CH_2), 109.5 (s, =CH), 93.6, 76.5 (both s, C≡C), 23.1, 17.3 (both s, CH_3).

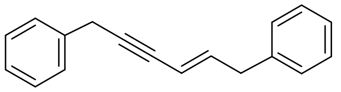

i$_4$.[6] ^{1}H NMR (300.13 MHz, $CDCl_3$, 298 K): δ 7.55-7.35 (10H, Ph), 7.04 (dt, $^3J_{H\text{-}H}$ = 15.8, $^4J_{H\text{-}H}$ = 6.9, 1H, =CH), 7.04 (dt, $^3J_{H\text{-}H}$ = 15.8, $^5J_{H\text{-}H}$ = 2.0, 1H, =CH), 3.91 (d, $^4J_{H\text{-}H}$ = 2.1, 1H, CH_2), 3.64 (d, $^3J_{H\text{-}H}$ = 7.4, 1H, CH_2). $^{13}C\{^1H\}$-apt NMR (75.48 MHz, $CDCl_3$, 298 K): δ 142.1 (s, =CH), 139.2, 137.0 (both s, C Ph), 128.8, 128.7, 128.6, 128.0, 126.7 126.5 (all s, CH Ph), 111.2 (s, =CH), 86.9, 81.1 (both s, C≡C), 39.4, 25.9 (both s, CH_2).

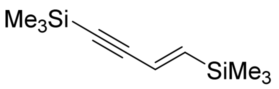

j$_4$.[7] ^{1}H NMR (500.13 MHz, C_6D_6, 298 K): δ 6.52 (d, $^3J_{H\text{-}H}$ = 19.3, 1H, =CH), 6.00 (d, $^3J_{H\text{-}H}$ = 19.3, 1H, =CH), 0.21, 0.08 (both s, 9H, $SiMe_3$). $^{13}C\{^1H\}$-apt NMR (125.77 MHz, C_6D_6, 298 K): δ 146.5 (s, =CH), 124.4 (s, =CH), 106.2 (s, C≡C), 0.05, -1.79 (both s, $SiMe_3$).

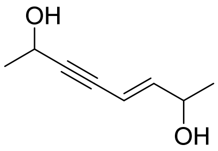

a$_5$.[8] ^{1}H NMR (300.13 MHz, $CDCl_3$, 298 K): δ 6.16 (dd, $^3J_{H\text{-}H}$ = 15.9, $^3J_{H\text{-}H}$ = 5.7, 1H, =CH), 5.71 (ddd, $^3J_{H\text{-}H}$ = 15.9, $^3J_{H\text{-}H}$ = 1.6, $^4J_{H\text{-}H}$ = 1.6, 1H, =CH), 4.63 (dq, $^3J_{H\text{-}H}$ = 6.6, $^4J_{H\text{-}H}$ = 1.4, 1H, C*H*-OH), 4.14 (ddq, $^3J_{H\text{-}H}$ = 6.5, $^3J_{H\text{-}H}$ = 6.5, $^3J_{H\text{-}H}$ = 1.1, H, C*H*-OH), 2.11 (br, 1H, OH), 1.80 (br, 1H, OH), 1.46 (d, $^3J_{H\text{-}H}$ = 6.5, 3H, CH_3), 1.28 (d, $^3J_{H\text{-}H}$ = 6.5, 3H, CH_3). $^{13}C\{^1H\}$-apt NMR (75.48 MHz, $CDCl_3$, 298 K): δ 147.1, 108.6 (both s, =CH), 91.9, 82.0 (both s, C≡C), 68.2, 58.9 (both s, *C*H-OH), 24.5, 23.1 (both s, CH_3).

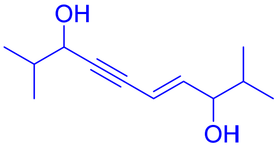

b$_5$. ^{1}H NMR (300.13 MHz, $CDCl_3$, 298 K): δ 6.14 (dd, $^3J_{H\text{-}H}$ = 15.9, $^3J_{H\text{-}H}$ = 6.3, 1H, =CH), 5.73 (dt, $^3J_{H\text{-}H}$ = 15.9, $^4J_{H\text{-}H}$ = 1.6, 1H, =CH), 4.28 (dd, $^3J_{H\text{-}H}$ = 5.7, $^3J_{H\text{-}H}$ = 1.7, 1H, C*H*-OH), 3.92 (td, $^3J_{H\text{-}H}$ = 6.1, $^3J_{H\text{-}H}$ = 1.3, 1H, C*H*-OH), 1.94-1.69 (4H, 2 CH + 2 OH), 1.01 (d, $^3J_{H\text{-}H}$ = 6.7, 3H, CH_3), 0.99 (d, $^3J_{H\text{-}H}$ = 6.7, 3H, CH_3), 0.93 (d, $^3J_{H\text{-}H}$ = 6.4, 3H, CH_3), 0.91 (d, $^3J_{H\text{-}H}$ = 6.6, 3H, CH_3). ^{13}C{^{1}H}-apt NMR (75.48 MHz, $CDCl_3$, 298 K): δ 144.5 (s, =CH), 110.3 (s, =CH), 89.6 (s, C≡C), 83.7 (s, C≡C), 77.3 (s, *C*H-OH), 68.5 (s, *C*H-OH), 40.0 (s, CH_2), 34.8 (s, CH), 33.9 (s, CH), 18.3, 18.2, 17.9, 17.7 (all s, CH_3). HRMS (electrospray, *m*/*z*) calcd. for $C_{12}H_{20}NaO_2$ [M + Na]$^+$: 219.1356; found: 219.1360.

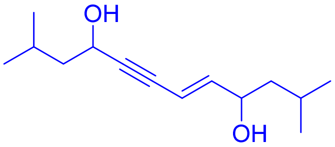

c$_5$. ^{1}H NMR (300.13 MHz, $CDCl_3$, 298 K): δ 6.13 (dd, $^3J_{H\text{-}H}$ = 15.9, $^3J_{H\text{-}H}$ = 6.2, 1H, =CH), 5.73 (dt, $^3J_{H\text{-}H}$ = 15.9, $^4J_{H\text{-}H}$ = 1.6, 1H, =CH), 4.53 (t, $^3J_{H\text{-}H}$ = 7.1, 1H, C*H*-OH), 4.22 (dd, $^3J_{H\text{-}H}$ = 6.8, $^3J_{H\text{-}H}$ = 6.5, 1H, C*H*-OH), 1.98-1.23 (8H, 2 CH_2 + 2 CH + 2 OH), 0.97-0.90 (12H, CH_3). ^{13}C{^{1}H}-apt NMR (75.48 MHz, $CDCl_3$, 298 K): δ 146.5 (s, =CH), 109.2 (s, =CH), 91.3 (s, C≡C), 82.8 (s, C≡C), 70.7 (s, *C*H-OH), 61.6 (s, *C*H-OH), 47.0 (s, CH_2), 46.2 (s, CH_2), 24.9 (s, CH), 24.7 (s, CH), 23.1, 22.7, 22.6, 22.4 (all s, CH_3). HRMS (electrospray, *m*/*z*) calcd. for $C_{14}H_{24}NaO_2$ [M + Na]$^+$: 247.1669; found: 247.1670.

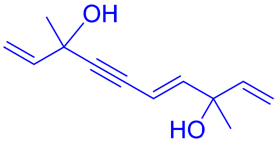

d$_5$. ^{1}H NMR (300.13 MHz, $CDCl_3$, 298 K): δ 6.14 (d, $^3J_{H\text{-}H}$ = 16.0, 1H, =CH), 6.03-5.86 (2H, =CH), 5.79 (d, $^3J_{H\text{-}H}$ = 16.0, 1H, =CH), 5.48 (dd, $^3J_{H\text{-}H}$ = 17.0, $^2J_{H\text{-}H}$ = 1.0, 1H, =CH_2), 5.25 (dd, $^3J_{H\text{-}H}$ = 17.0, $^2J_{H\text{-}H}$ = 1.0, 1H, =CH_2), 5.12 (dd, $^3J_{H\text{-}H}$ = 10.2, $^2J_{H\text{-}H}$ = 1.0, 1H, =CH_2), 5.10 (dd, $^3J_{H\text{-}H}$ = 10.6, $^2J_{H\text{-}H}$ = 0.9, 1H, =CH_2), 2.27 (br, 1H, OH), 1.85 (br, 1H, OH), 1.56 (s, 3H, CH_3), 1.38 (s, 3H, CH_3). ^{13}C{^{1}H}-apt NMR (75.48 MHz, $CDCl_3$, 298 K): δ 148.4 (s, =CH), 142.8 (s, *C*H=CH_2), 142.1 (s, *C*H=CH_2), 113.7 (s, =CH_2), 113.3 (s, =CH_2), 107.6 (s, =CH), 92.0 (s, C≡C), 82.8 (s, C≡C), 73.5 (s, C-OH), 68.7 (s, C-OH), 30.1, 27.9 (both s, CH_3). HRMS (electrospray, *m*/*z*) calcd. for $C_{12}H_{16}NaO_2$ [M + Na]$^+$: 215.1043; found: 215.1037.

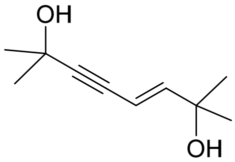

e$_5$.[9] ^{1}H NMR (300.13 MHz, $CDCl_3$, 298 K): δ 6.23 (d, $^3J_{H\text{-}H}$ = 16.0, 1H, =CH), 5.72 (d, $^3J_{H\text{-}H}$ = 16.0, 1H, =CH), 2.16, 1.64 (both br, 1H, OH), 1.52, 1.31 (both s, 6H, CH_3). ^{13}C{^{1}H}-apt NMR (75.48 MHz,

$CDCl_3$, 298 K): δ 150.7 (s, =CH), 106.5 (s, =CH), 94.6, 80.3 (both s, C≡C), 71.1, 65.7 (both s, *C*-OH), 31.6, 29.6 (both s, CH_3).

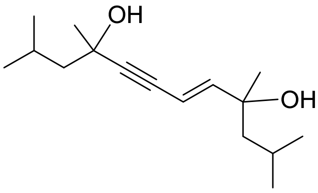

f5.[10] ^{1}H NMR (300.13 MHz, $CDCl_3$, 298 K): δ 6.15 (d, $^{3}J_{H-H}$ = 16.0, 1H, =CH), 5.73 (d, $^{3}J_{H-H}$ = 16.0, 1H, =CH), 1.97 (br, 1H, OH), 1.96-1.86 (m, 1H, CH), 1.78-1.68 (m, 1H, CH), 1.61 (d, $^{3}J_{H-H}$ = 6.1, 2H, CH_2), 1.50 (s, 3H, CH_3), 1.46 (d, $^{3}J_{H-H}$ = 6.3, 2H, CH_2), 1.41 (br, 1H, OH), 1.28 (s, 3H, CH_3), 1.01 (d, $^{3}J_{H-H}$ = 6.7, 3H, CH_3), 0.99 (d, $^{3}J_{H-H}$ = 6.7, 3H, CH_3), 0.93 (d, $^{3}J_{H-H}$ = 6.6, 6H, CH_3), 0.91 (d, $^{3}J_{H-H}$ = 6.6, 3H, CH_3). ^{13}C{^{1}H}-apt NMR (75.48 MHz, $CDCl_3$, 298 K): δ 150.2 (s, =CH), 106.8 (s, =CH), 93.8 (s, C≡C), 81.9 (s, C≡C), 73.9 (s, C-OH), 68.6 (s, C-OH), 52.1 (s, CH_2), 51.1 (s, CH_2), 31.0 (s, CH), 29.0 (s, CH), 25.3, 24.6, 24.6, 24.5, 24.4, 24.2 (all s, CH_3).

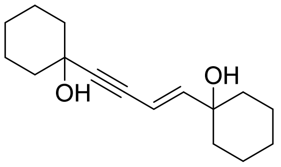

g5.[11] ^{1}H NMR (300.13 MHz, $CDCl_3$, 298 K): δ 6.23 (d, $^{3}J_{H-H}$ = 16.1, 1H, =CH), 5.79 (d, $^{3}J_{H-H}$ = 16.1, 1H, =CH), 2.18 (br, 1H, OH), 1.93-1.18 (21H, 1 OH + 20 CH_2). ^{13}C{^{1}H}-apt NMR (75.48 MHz, $CDCl_3$, 298 K): δ 150.5, 107.2 (both s, =CH), 93.6, 82.6 (both s, C≡C), 71.9, 69.1 (both s, *C*-OH), 40.1, 37.7, 25.5, 25.3, 23.4, 22.0 (all s, CH_2).

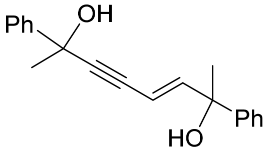

h5.[8] ^{1}H NMR (300.13 MHz, $CDCl_3$, 298 K): δ 7.66-7.59 (2H, Ph), 7.48-7.40 (2H, Ph), 7.38-20 (8H, Ph), 6.44 (d, $^{3}J_{H-H}$ = 15.9, 1H, =CH), 5.84 (d, $^{3}J_{H-H}$ = 15.9, 1H, =CH), 2.50 (br, 1H, OH), 2.03 (br, 1H, OH), 1.76 (s, 3H, CH_3), 1.66 (s, 3H, CH_3). ^{13}C{^{1}H}-apt NMR (75.48 MHz, $CDCl_3$, 298 K): δ 149.7 (s, =CH), 145.7, 145.6 (both s, C Ph), 128.6, 128.4, 127.8, 127.5, 125.3, 125.1 (s, CH Ph), 107.6 (s, =CH), 93.8 (s, C≡C), 83.0 (s, C≡C), 74.7 (s, C-OH), 70.4 (s, C-OH), 33.3, 29.4 (both s, CH_3).

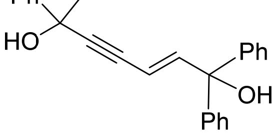

i5.[11] ^{1}H NMR (300.13 MHz, $CDCl_3$, 298 K): δ 7.58-7.52 (m, 4H, Ph), 7.30-7.20 (16H, Ph), 6.75 (d, $^{3}J_{H-H}$ = 15.8, 1H, =CH), 5.91 (d, $^{3}J_{H-H}$ = 15.8, 1H, =CH), 2.87 (br, 1H, OH), 2.35 (br, 1H, OH). ^{13}C{^{1}H}-apt NMR (75.48 MHz, $CDCl_3$, 298 K): δ 148.5 (s, =CH), 145.1, 145.0 (both s, C Ph), 132.6, 130.2, 128.5, 128.4, 127.8, 127.8, 127.0, 126.2 (s, CH Ph), 109.0 (s, =CH), 93.3 (s, C≡C), 85.3 (s, C≡C), 79.4 (s, C-OH), 75.0 (s, C-OH).

• Characterization Data of Cross-Coupling Products

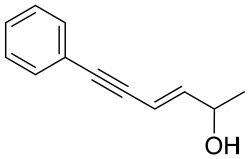

a$_6$.[12] ^{1}H NMR (300.13 MHz, $CDCl_3$, 298 K): δ 7.46-7.40 (m, 2H, Ph), 7.34-7.28 (3H, Ph), 6.27 (dd, $^3J_{H-H}$ = 15.9, $^3J_{H-H}$ = 5.8, 1H, =CH), 5.93 (dd, $^3J_{H-H}$ = 15.9, $^4J_{H-H}$ = 1.4, 1H, =CH), 4.42 (m, 1H, C*H*-OH), 1.57 (br, 1H, OH), 1.33 (d, $^3J_{H-H}$ = 6.5, 3H, CH_3). ^{13}C{^{1}H}-apt NMR (75.48 MHz, $CDCl_3$, 298 K): δ 146.8 (s, =CH), 131.7, 128.4, 128.3 (all s, CH Ph), 123.4 (s, C Ph), 109.4 (s, =CH), 90.3 (s, C≡C), 87.4 (s, C≡C), 68.5 (s, CH-OH), 23.2 (s, CH_3).

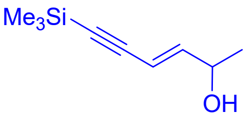

b$_6$. ^{1}H NMR (300.13 MHz, C_6D_6, 298 K): δ 6.09 (dd, $^3J_{H-H}$ = 15.9, $^3J_{H-H}$ = 5.3, 1H, =CH), 5.70 (d, $^3J_{H-H}$ = 15.9, $^3J_{H-H}$ = 1.6, 1H, =CH), 3.77 (m, 1H, C*H*-OH), 0.86 (d, $^3J_{H-H}$ = 6.5, 3H, CH_3), 0.75 (br d, $^3J_{H-H}$ = 4.3, 1H, OH), 0.21 (s, 9H, $SiMe_3$). ^{13}C{^{1}H}-apt NMR (75.48 MHz, $CDCl_3$, 298 K): δ 148.8 (s, =CH), 109.0 (s, =CH), 104.4 (s, C≡C), 94.9 (s, C≡C), 67.7 (s, CH), 23.0 (s, CH_3), 0.08 (s, $SiMe_3$). HR-APCI-MS (*m*/*z*) calcd. for $C_9H_{17}OSi$ $[M + H]^+$: 169.1043; found: 169.1023.

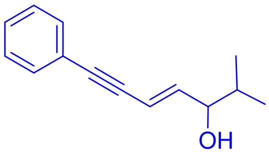

c$_6$. ^{1}H NMR (300.13 MHz, $CDCl_3$, 298 K): δ 7.46-7.41 (m, 2H, Ph), 7.33-7.28 (3H, Ph), 6.25 (dd, $^3J_{H-H}$ = 15.9, $^3J_{H-H}$ = 6.3, 1H, =CH), 5.84 (dd, $^3J_{H-H}$ = 15.9, $^4J_{H-H}$ = 1.4, 1H, =CH), 3.99 (ddd, $^3J_{H-H}$ = 6.0, $^3J_{H-H}$ = 6.0, $^4J_{H-H}$ = 1.3, 1H, C*H*-OH), 1.86-1.74 (m, 1H, CH), 1.57 (d, $^3J_{H-H}$ = 3.8, 1H, OH), 0.97 (d, $^3J_{H-H}$ = 6.8, 3H, CH_3), 0.95 (d, $^3J_{H-H}$ = 6.8, 3H, CH_3). ^{13}C{^{1}H}-apt NMR (75.48 MHz, $CDCl_3$, 298 K): δ 144.3 (s, =CH), 131.7, 128.5, 128.3 (all s, CH Ph), 123.4 (s, C Ph), 110.9 (s, =CH), 90.1 (s, C≡C), 87.6 (s, C≡C), 77.5 (s, OHCH), 34.0 (s, CH), 18.3, 17.9 (both s, CH_3). HRMS (electrospray, *m*/*z*) calcd. for $C_{14}H_{17}O$ $[M + H]^+$: 201.1279; found: 201.1272.

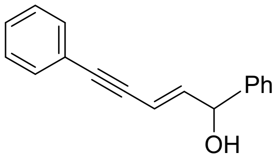

d$_6$.[13] ^{1}H NMR (300.13 MHz, $CDCl_3$, 298 K): δ 7.48-7.28 (10H, Ph), 6.37 (dd, $^3J_{H-H}$ = 15.7, $^3J_{H-H}$ = 5.8, 1H, =CH), 6.02 (dd, $^3J_{H-H}$ = 15.8, $^4J_{H-H}$ = 1.5, 1H, =CH), 5.33 (m, 1H, C*H*-OH), 2.04 (d, $^3J_{H-H}$ = 3.6, 1H, OH). ^{13}C{^{1}H}-apt NMR (75.48 MHz, $CDCl_3$, 298 K): δ 144.4 (s, =CH), 142.0 (s, C Ph), 131.7, 128.9, 128.5, 128.4, 128.3, 126.6 (all s, CH Ph), 123.3 (s, C Ph), 110.4 (s, =CH), 90.8 (s, C≡C), 87.4 (s, C≡C), 74.7 (s, CH-OH).

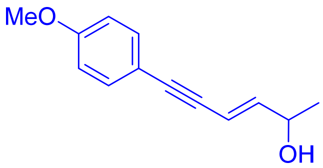

e6. ^{1}H NMR (300.13 MHz, $CDCl_3$, 298 K): δ 7.41-7.32 (m, 2H, Ph), 6.89-6.78 (m, 2H, Ph), 6.23 (dd, $^{3}J_{H\text{-}H}$ = 15.9, $^{3}J_{H\text{-}H}$ = 5.9, 1H, =CH), 5.91 (dd, $^{3}J_{H\text{-}H}$ = 15.9, $^{4}J_{H\text{-}H}$ = 1.3, 1H, =CH), 4.40 (m, 1H, C*H*-OH), 3.81 (s, OCH_3), 1.57 (d, $^{3}J_{H\text{-}H}$ = 4.6, 1H, OH), 1.32 (d, $^{3}J_{H\text{-}H}$ = 6.4, 3H, CH_3). $^{13}C\{^{1}H\}$-apt NMR (75.48 MHz, $CDCl_3$, 298 K): δ 159.7 (s, C Ph), 145.9 (s, =CH), 133.1, 114.1 (both s, CH Ph), 115.5 (s, C Ph), 109.7 (s, =CH), 90.3 (s, C≡C), 86.1 (s, C≡C), 68.5 (s, CH-OH), 55.4 (s, OCH_3), 23.2 (s, CH_3). HR-APCI-MS (*m*/*z*) calcd. for $C_{13}H_{15}O_2$ $[M+H]^+$: 203.1067; found: 203.1076.

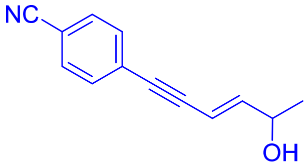

f6. ^{1}H NMR (300.13 MHz, $CDCl_3$, 298 K): δ 7.65-7.55 (m, 2H, Ph), 7.54-7.44 (m, 2H, Ph), 6.34 (dd, $^{3}J_{H\text{-}H}$ = 15.9, $^{3}J_{H\text{-}H}$ = 5.4, 1H, =CH), 5.94 (dd, $^{3}J_{H\text{-}H}$ = 15.9, $^{4}J_{H\text{-}H}$ = 1.4, 1H, =CH), 4.44 (m, 1H, C*H*-OH), 1.60 (d, $^{3}J_{H\text{-}H}$ = 4.5, 1H, OH), 1.34 (d, $^{3}J_{H\text{-}H}$ = 6.5, 3H, CH_3). $^{13}C\{^{1}H\}$-apt NMR (75.48 MHz, $CDCl_3$, 298 K): δ 148.7 (s, =CH), 132.2, 132.1 (both s, CH Ph), 128.4 (s, C Ph), 118.6 (s, CN), 111.6 (s, C Ph), 108.5 (s, =CH), 91.9 (s, C≡C), 88.5 (s, C≡C), 68.2 (s, CH-OH), 23.2 (s, CH_3). HR-APCI-MS (*m*/*z*) calcd. for $C_{13}H_{12}NO$ $[M+H]^+$: 198.0913; found: 198.0924.

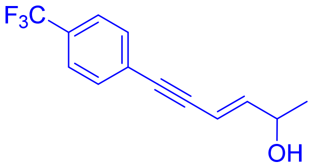

g6. ^{1}H NMR (300.13 MHz, $CDCl_3$, 298 K): δ 7.54 (m, 4H, Ph), 6.33 (dd, $^{3}J_{H\text{-}H}$ = 15.9, $^{3}J_{H\text{-}H}$ = 5.6, 1H, =CH), 5.94 (dd, $^{3}J_{H\text{-}H}$ = 15.9, $^{4}J_{H\text{-}H}$ = 1.5, 1H, =CH), 4.43 (m, 1H, C*H*-OH), 1.55, (br, 1H, OH), 1.33 (d, $^{3}J_{H\text{-}H}$ = 6.5, 3H, CH_3). $^{13}C\{^{1}H\}$-apt NMR (75.48 MHz, $CDCl_3$, 298 K): δ 148.0 (s, =CH), 131.9 (s, CH Ph), 130.0 (q, $^{2}J_{C\text{-}F}$ = 32.0, C Ph), 127.3 (s, C Ph), 125.4 (q, $^{3}J_{C\text{-}F}$ = 3.8, CH Ph), 124.1 (q, $^{1}J_{C\text{-}F}$ = 272.1, CF_3), 108.8 (s, =CH), 89.8 (s, C≡C), 88.8 (s, C≡C), 68.3 (s, CH-OH), 23.2 (s, CH_3). HRMS (electrospray, *m*/*z*) calcd. for $C_{13}H_{10}F_3O$ $[M-H]^-$: 239.0689; found: 239.0683.

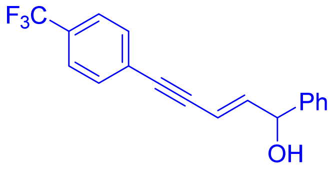

h6. ^{1}H NMR (300.13 MHz, $CDCl_3$, 298 K): δ 7.61-7.46 (m, 5H, Ph), 7.41-.7.36 (4H, Ph), 6.45 (dd, $^{3}J_{H\text{-}H}$ = 15.9, $^{3}J_{H\text{-}H}$ = 5.6, 1H, =CH), 6.07 (dd, $^{3}J_{H\text{-}H}$ = 15.8, $^{4}J_{H\text{-}H}$ = 1.6, 1H, =CH), 5.33 (m, 1H, C*H*-OH), 2.00 (d, $^{3}J_{H\text{-}H}$ = 2.0, 1H, OH). $^{13}C\{^{1}H\}$-apt NMR (75.48 MHz, $CDCl_3$, 298 K): δ 145.7 (s, =CH), 141.8 (s, C Ph),

131.9 (s, CH Ph), 130.0 (q, $^{2}J_{C\text{-}F}$ = 32.7 32.4, C Ph), 129.0, 128.4 (both s, CH Ph), 127.2 (q, $^{3}J_{C\text{-}F}$ = 1.6, C Ph), 126.6 (s, CH Ph), 125.4 (q, $^{3}J_{C\text{-}F}$ = 3.8, CH Ph), 124.1 (q, $^{3}J_{C\text{-}F}$ = 272.3, CF_3), 109.7 (s, =CH), 89.8 (s, C≡C), 89.3 (s, C≡C), 75.6 (s, CH-OH). HRMS (electrospray, *m/z*) calcd. for $C_{18}H_{12}F_3O$ $[M\text{-}H]^-$: 301.0846; found: 301.0837.

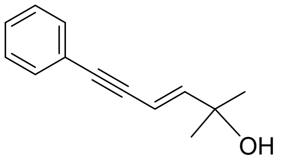

i_6.[14] ^{1}H NMR (300.13 MHz, $CDCl_3$, 298 K): δ 7.47-7.37 (m, 2H, Ph), 7.35-7.27 (3H, Ph), 6.36 (d, $^{3}J_{H\text{-}H}$ = 16.0, 1H, =CH), 5.96 (d, $^{3}J_{H\text{-}H}$ = 16.0, 1H, =CH), 1.49 (br, 1H, OH), 1.37 (s, 6H, CH_3). $^{13}C\{^{1}H\}$-apt NMR (75.48 MHz, $CDCl_3$, 298 K): δ 150.7 (s, =CH), 131.6, 128.4, 128.2 (all s, CH Ph), 123.5 (s, C Ph), 107.1 (s, =CH), 90.1 (s, C≡C), 87.7 (s, C≡C), 71.2 (s, C-OH), 29.7 (s, CH_3).

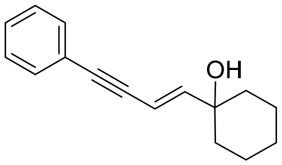

j_6.[15] ^{1}H NMR (300.13 MHz, $CDCl_3$, 298 K): δ 7.51-7.37 (m, 2H, Ph), 7.36-7.27 (3H, Ph), 6.37 (d, $^{3}J_{H\text{-}H}$ = 16.0, 1H, =CH), 6.00 (d, $^{3}J_{H\text{-}H}$ = 16.0, 1H, =CH), 1.71-1.50 (9H, CH_2), 1.37-1.24 (2H, OH + 1H CH_2). $^{13}C\{^{1}H\}$-apt NMR (75.48 MHz, $CDCl_3$, 298 K): δ 150.7 (s, =CH), 131.6, 128.4, 128.2 (all s, CH Ph), 123.5 (s, C Ph), 107.5 (s, =CH), 90.1 (s, C≡C), 88.0 (s, C≡C), 72.1 (s, C-OH), 37.8, 25.5, 22.0 (all s, CH_2).

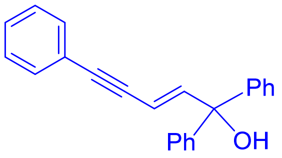

k_6. ^{1}H NMR (300.13 MHz, $CDCl_3$, 298 K): δ 7.50-7.27 (15H, Ph), 6.84 (d, $^{3}J_{H\text{-}H}$ = 15.8, 1H, =CH), 6.07 (d, $^{3}J_{H\text{-}H}$ = 15.8, 1H, =CH), 2.32 (s, 1H, OH). $^{13}C\{^{1}H\}$-apt NMR (75.48 MHz, $CDCl_3$, 298 K): δ 147.8 (s, =CH), 145.3 (s, C Ph), 131.6, 128.5, 127.7, 127.1 (all s, CH Ph), 123.4 (s, C Ph), 109.7 (s, =CH), 91.2 (s, C≡C), 87.7 (s, C≡C), 79.5 (s, C-OH). HRMS (electrospray, *m/z*) calcd. for $C_{23}H_{17}O$ $[M\text{-}H]^-$: 309.1285; found: 309.1290.

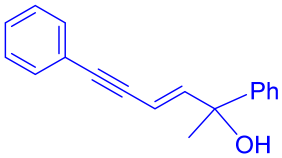

l_6. ^{1}H NMR (300.13 MHz, $CDCl_3$, 298 K): δ 7.55-7.27 (10H, Ph), 6.52 (d, $^{3}J_{H\text{-}H}$ = 15.9, 1H, =CH), 6.02 (d, $^{3}J_{H\text{-}H}$ = 15.9, 1H, =CH), 1.95 (s, 1H, OH), 1.72 (s, 3H, CH_3). $^{13}C\{^{1}H\}$-apt NMR (75.48 MHz, $CDCl_3$, 298 K): δ 149.1 (s, =CH), 145.6 (s, C Ph), 131.5, 128.5, 128.3, 128.2, 127.4, 125.2 (all s, CH Ph), 123.3 (s, C Ph), 108.1 (s, =CH), 90.6 (s, C≡C), 87.4 (s, C≡C), 74.6 (s, C-OH), 29.4 (s, CH_3). HRMS (electrospray, *m/z*) calcd. for $C_{18}H_{16}NaO$ $[M+Na]^+$: 271.1093; found: 271.1096.

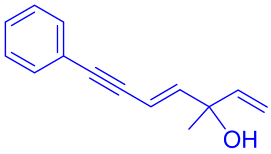

m6. ^{1}H NMR (300.13 MHz, $CDCl_3$, 298 K): δ 7.49-7.38 (m, 2H, Ph), 7.36-7.27 (3H, Ph), 6.32 (d, $^3J_{H\text{-}H}$ = 16.0, 1H, =CH), 5.98 (d, $^3J_{H\text{-}H}$ = 16.0, 1H, =CH), 5.97 (dd, $^3J_{H\text{-}H}$ = 17.3, $^3J_{H\text{-}H}$ = 10.6, 1H, =CH), 5.30 (dd, $^3J_{H\text{-}H}$ = 17.3, $^2J_{H\text{-}H}$ = 0.9, 1H, $=CH_2$), 5.14 (dd, $^3J_{H\text{-}H}$ = 10.6, $^2J_{H\text{-}H}$ = 0.9, 1H, $=CH_2$), 1.66 (s, 1H, OH), 1.43 (s, 3H, CH_3). $^{13}C\{^1H\}$-apt NMR (75.48 MHz, $CDCl_3$, 298 K): δ 148.0 (s, =CH), 142.9 (s, *C*H=CH_2), 131.6, 128.4, 128.3 (all s, CH Ph), 123.4 (s, C Ph), 113.4 (s, $=CH_2$), 108.4 (s, =CH), 90.5 (s, C≡C), 87.5 (s, C≡C), 73.6 (s, C-OH), 28.0 (s, CH_3). HRMS (electrospray, *m*/*z*) calcd. for $C_{14}H_{14}NaO$ $[M+Na]^+$: 221.0937; found: 221.0939.

• Ethisterone Homo-Coupling and Ethisterone-Phenylacetylene Cross-Coupling

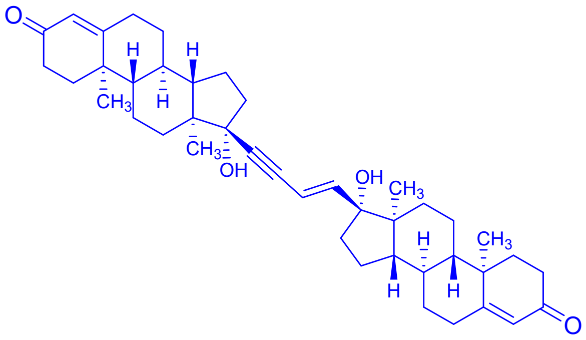

a8. ^{1}H NMR (300.13 MHz, $CDCl_3$, 298 K): δ 6.29 (d, $^3J_{H\text{-}H}$ = 16.0, 1H, =CH), 5.73 (d, $^3J_{H\text{-}H}$ = 16.0, 1H, =CH, overlapping with a singlet assigned to the 2 C*H*-CO protons) 2.52-0.84 (40H, 2H OH + 32H CH_2 + 6H CH), 1.20 (s, 3H, CH_3), 1.19 (s, 3H, CH_3), 0.95 (s, 3H, CH_3), 0.90 (s, 3H, CH_3). $^{13}C\{^1H\}$-apt NMR (75.48 MHz, $CDCl_3$, 298 K): δ 199.6 (s, C=O), 196.5 (s, C=O), 171.2 (s, *C*=CH), 171.0 (s, *C*=CH), 147.8 (s, =CH), 124.1 (s, =*C*H-CO), 124.0 (s, =*C*H-CO), 107.6 (s, =CH), 93.0 (s, C≡C), 84.5 (s, C≡C), 84.1 (s, C-OH), 80.4 (s, C-OH), 53.6, 53.5, 50.2, 50.0 (all s, CH), 47.2, 47.1 (both s, C), 39.1, 38.9 (both s, CH_2), 38.8, 37.0 (both s, C), 36.5, 36.4 (both s, CH), 35.9, 34.1, 34.0, 33.0, 32.9, 32.4, 31.8, 31.7 (all s, CH_2), 17.6, 17.5, 14.2, 13.0 (all s, CH_3). HRMS (electrospray, *m*/*z*) calcd. for $C_{42}H_{56}NaO_4$ $[M + Na]^+$: 647.4071; found: 647.4062.

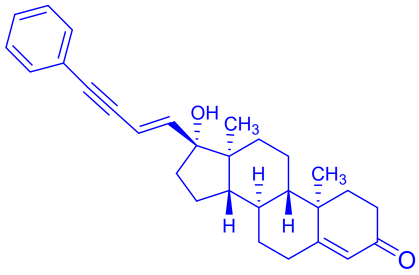

b8. ^{1}H NMR (300.13 MHz, $CDCl_3$, 298 K): δ 7.46-7.39 (2H, CH Ph), 7.32-7.27 (3H, CH Ph), 6.42 (d, $^3J_{H\text{-}H}$ = 15.9, 1H, =CH), 5.92 (d, $^3J_{H\text{-}H}$ = 15.9, 1H, =CH), 5.72 (s, 1H, CH-CO), 2.47-1.22 (20H, 1 OH + 16 CH_2 + 3H), 1.19 (s, 3H, CH_3), 0.97 (s, 3H, CH_3). $^{13}C\{^1H\}$-apt NMR (75.48 MHz, $CDCl_3$, 298 K): δ 199.5 (s, C=O), 171.1 (s, *C*=CH), 148.1 (s, =CH), 131.6, 128.4, 128.2 (all s, CH Ph),

124.1 (s, =*C*H-CO), 123.5 (s, C Ph), 107.9 (s, =CH), 89.9 (s, C≡C), 88.0 (s, C≡C), 84.2 (s, C-OH), 53.6, 50.1 (both s, CH), 47.2, 38.8 (both s, C), 37.1 (s, CH_2), 36.5 (s, CH), 35.8, 34.1, 32.9, 32.4, 31.8, 23.7, 20.8 (all s, CH_2), 17.6, 14.2 (both s, CH_3). HRMS (electrospray, *m*/*z*) calcd. for $C_{42}H_{56}NaO_4$ $[M + Na]^+$: 647.4071; found: 647.4062.

• **NMR spectra**

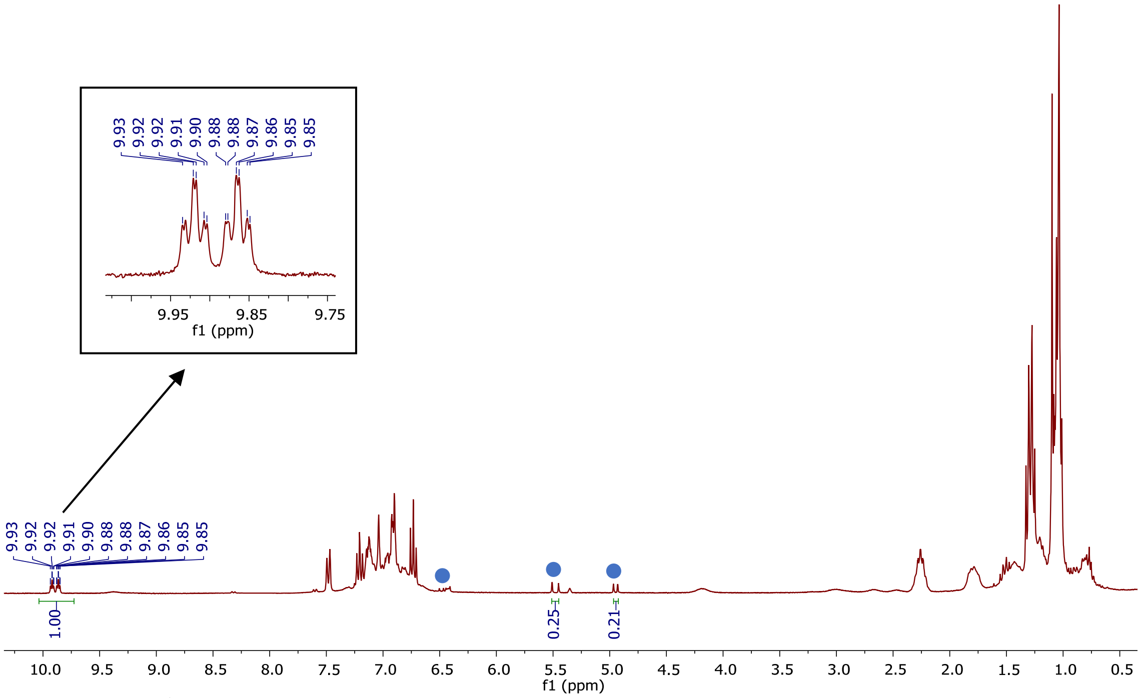

Figure S1. ^{1}H NMR spectrum (300.13 MHz, benzene-d_6, 298 K) of the reaction of RhH{κ^3-*P,O,P*-[xant(P^{i}Pr$_2$)$_2$]} (**1**) with phenylacetylene (1.4 eq) at room temperature (spectrum registered after 5 min). The formation of complexes Rh{(*E*)-CH=CHPh}{κ^3-*P,O,P*-[xant(P^{i}Pr$_2$)$_2$]} (**2**) and Rh(C≡CPh){κ^3-*P,O,P*-[xant(P^{i}Pr$_2$)$_2$]} (**3**), along with styrene (olefinic protons denoted with a blue spot), was observed (^{1}H and $^{31}P\{^{1}H\}$ NMR spectroscopies). The inset shows the resonance of one of the vinylic protons of complex **2**.

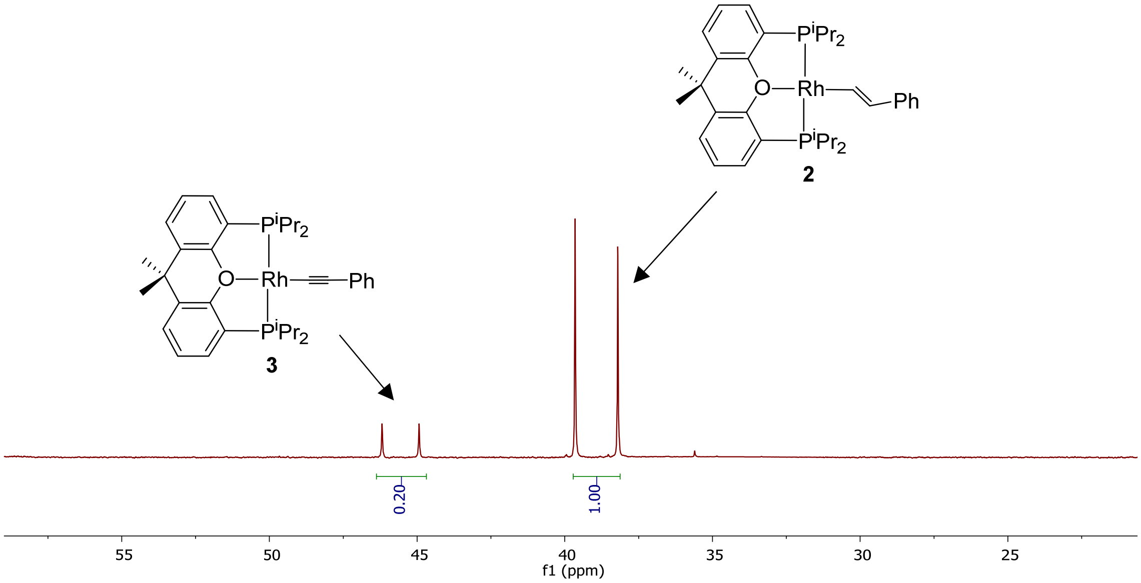

Figure S2. $^{31}P\{^{1}H\}$ NMR spectrum (121.49 MHz, benzene-d_6, 298 K) of the reaction of RhH{κ^3-*P,O,P*-[xant(P^{i}Pr$_2$)$_2$]} (**1**) with phenylacetylene (1.4 eq) showing the formation of complexes Rh{(*E*)-CH=CHPh}{κ^3-*P,O,P*-[xant(P^{i}Pr$_2$)$_2$]} (**2**) and Rh(C≡CPh){κ^3-*P,O,P*-[xant(P^{i}Pr$_2$)$_2$]} (**3**). Spectrum registered after 5 min of reaction at room temperature.

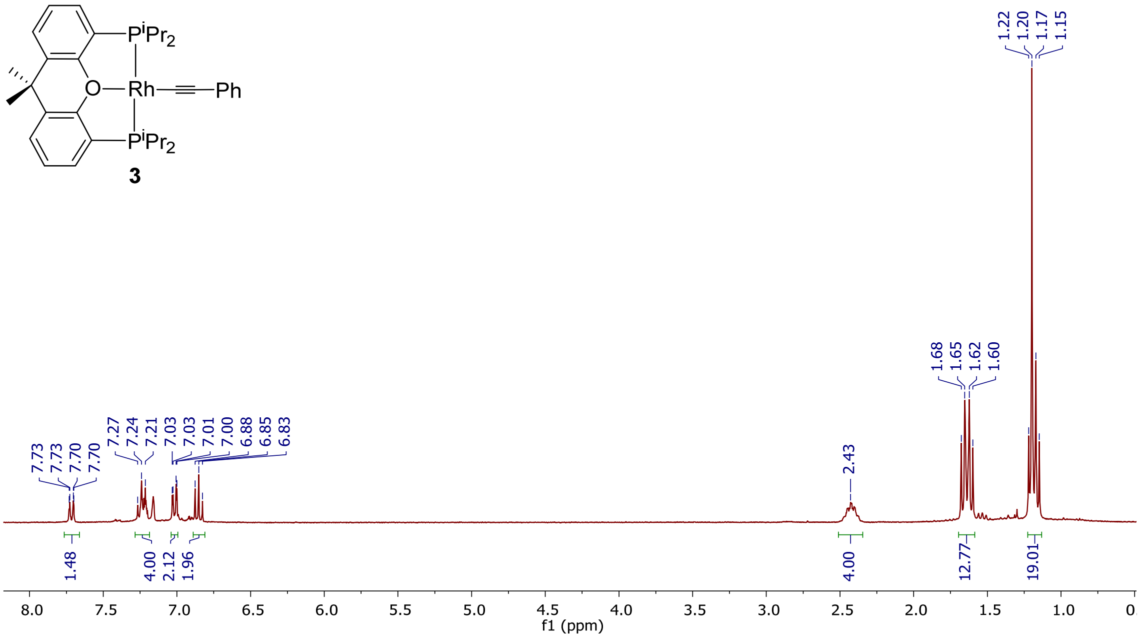

Figure S3. ^{1}H NMR spectrum (300.13 MHz, benzene-*d*$_6$, 298 K) of Rh(C≡CPh){κ^3-*P,O,P*-[xant(P^{i}Pr$_2$)$_2$]} (**3**).

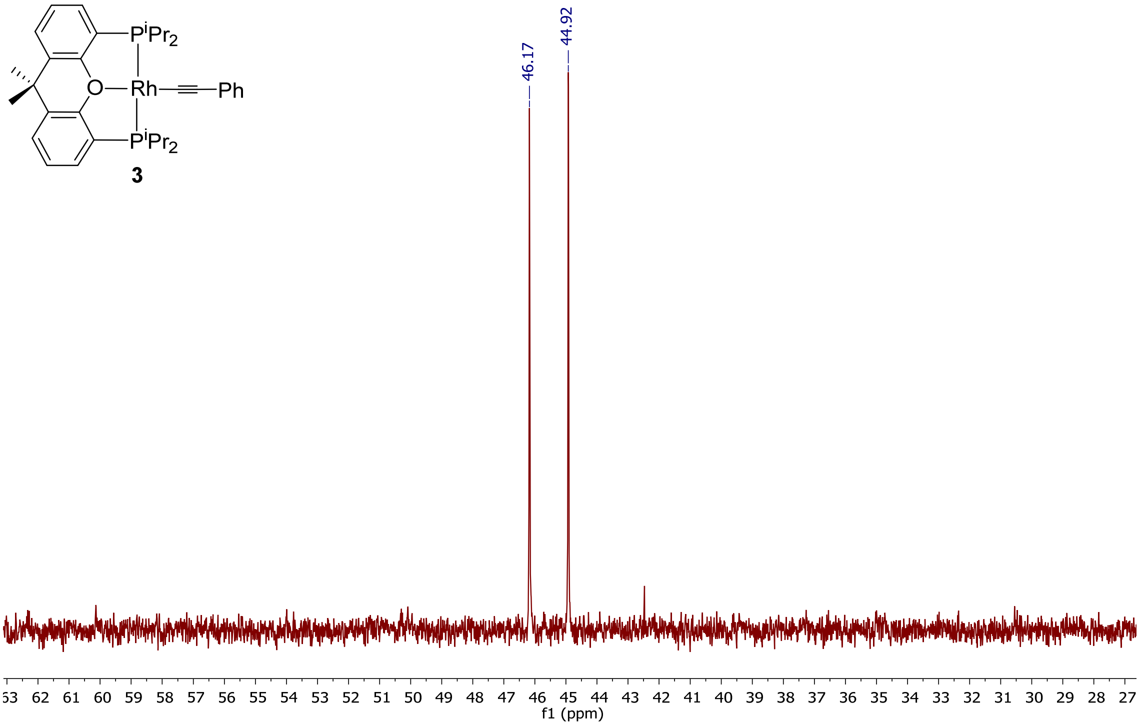

Figure S4. ^{31}P{^{1}H} NMR spectrum (121.49 MHz, benzene-*d*$_6$, 298 K) of Rh(C≡CPh){κ^3-*P,O,P*-[xant(P^{i}Pr$_2$)$_2$]} (**3**).

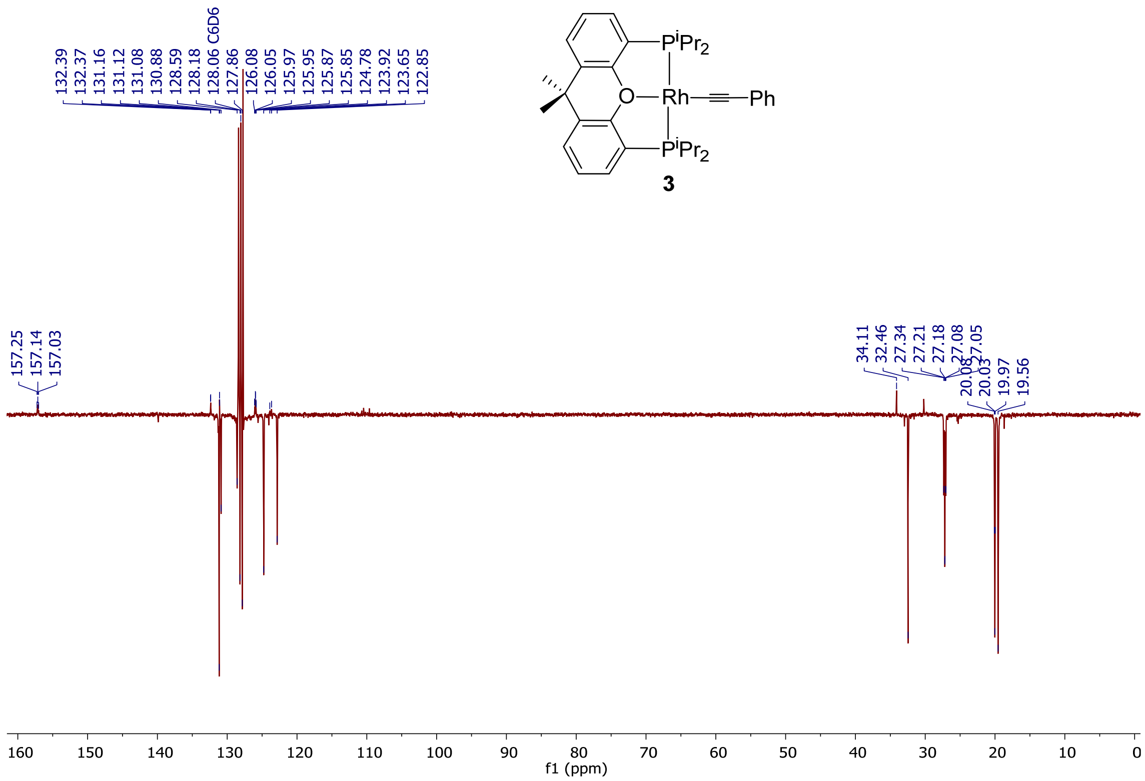

Figure S5. ^{13}C{^{1}H}-apt NMR spectrum (75.48 MHz, benzene-d_6, 298 K) of Rh(C≡CPh){κ^3-*P,O,P*-[xant(P^{i}Pr$_2$)$_2$]} (**3**).

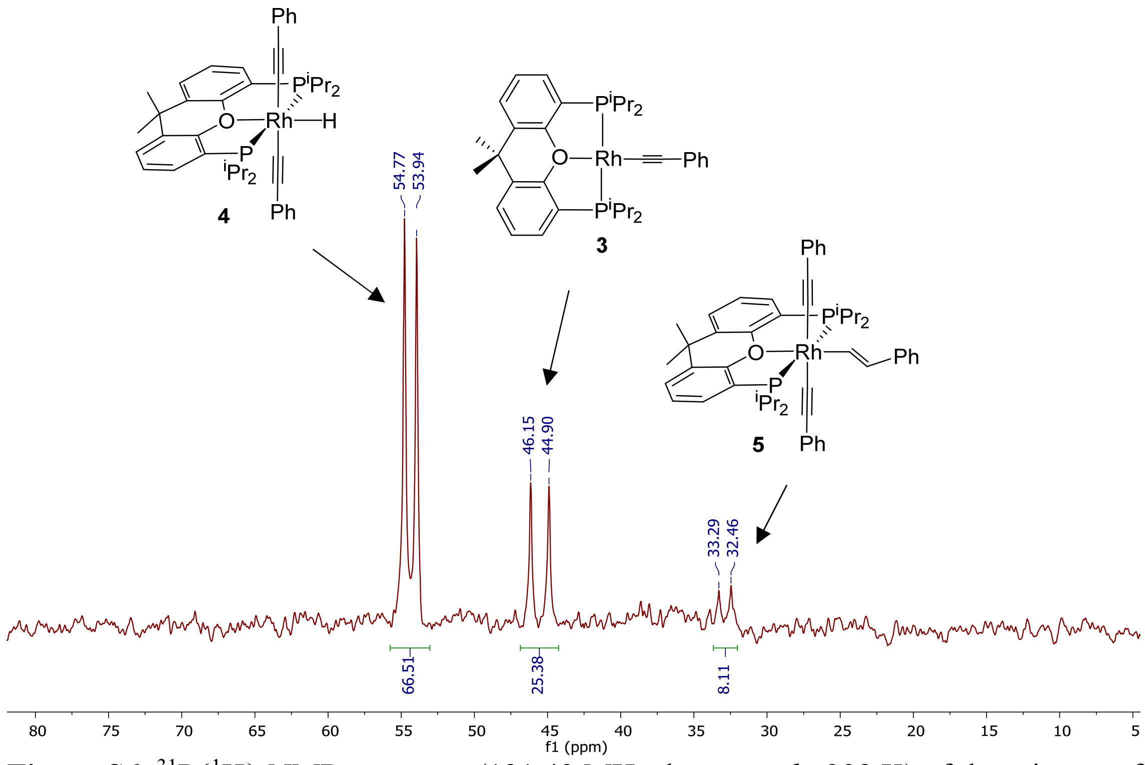

Figure S6. ^{31}P{^{1}H} NMR spectrum (121.49 MHz, benzene-d_6, 298 K) of the mixture of complexes **3**, **4**, and **5** obtained upon addition of phenylacetylene (11 eq) to a solution of complex **3** in benzene-d_6 and registered after 10-20 min of reaction.

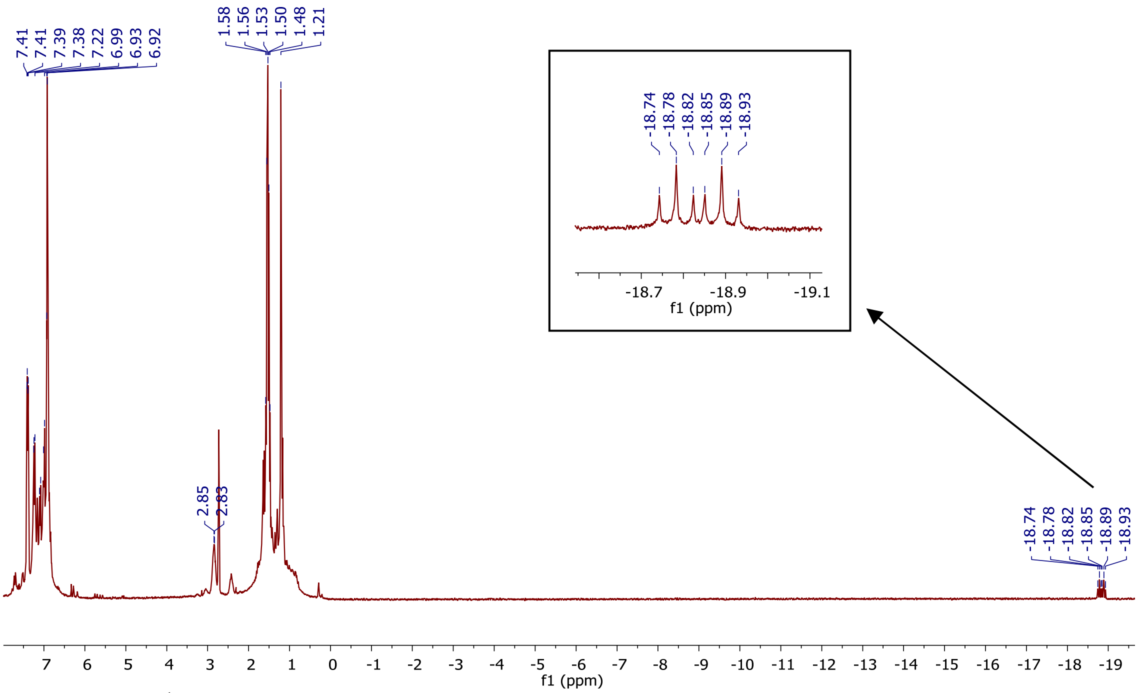

Figure S7. ^{1}H NMR spectrum (300.13 MHz, benzene-*d*$_6$, 298 K) of the mixture of complexes **3**, **4**, and **5** obtained upon addition of phenylacetylene (11 eq) to a solution of complex **3** in benzene-*d*$_6$ registered after 10-20 min of reaction. The inset shows the resonance of the hydride ligand of complex **4**.

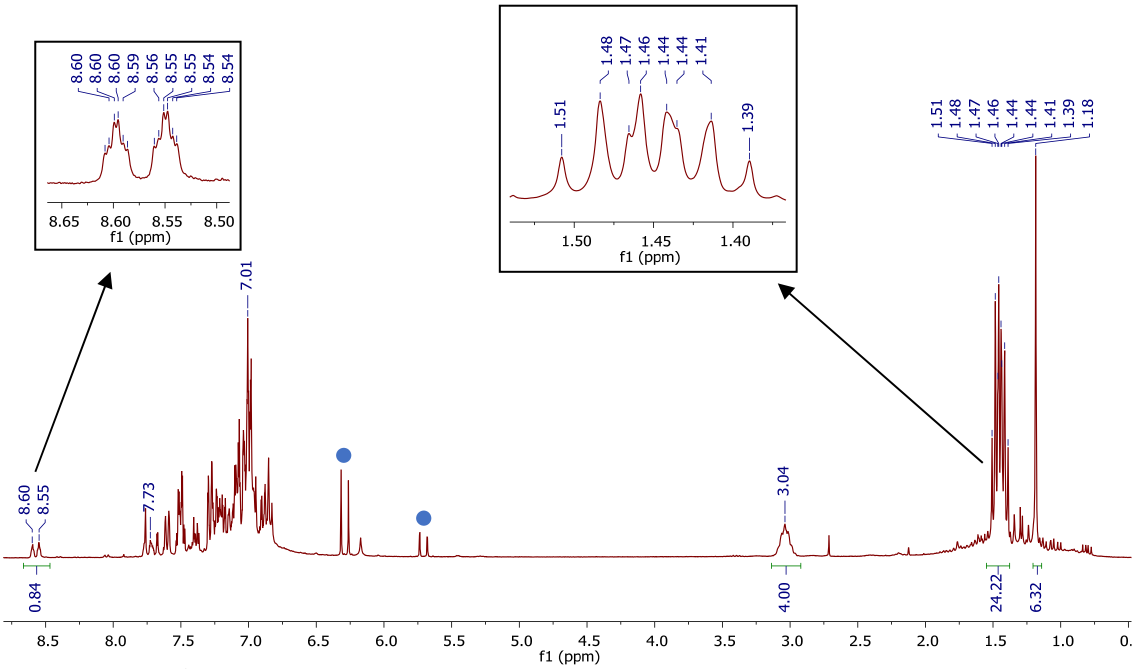

Figure S8. ^{1}H NMR spectrum (300.13 MHz, benzene-d$_6$, 298 K) of Rh{(*E*)-CH=CHPh}(C≡CPh)$_2${κ^3-*P,O,P*-[xant(P^{i}Pr$_2$)$_2$]} (**5**). Organic impurities are denoted with a blue spot. The insets show the resonances assigned to one of the olefinic protons of the complex and those of the methyl groups of the P^{i}Pr$_2$ groups.

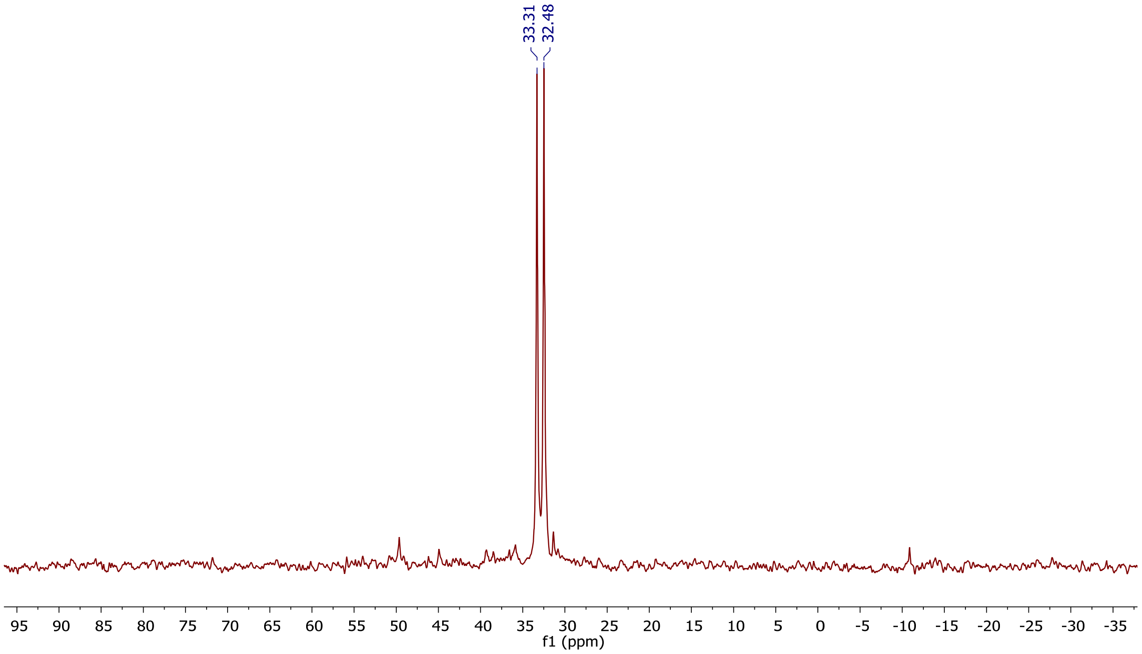

Figure S9. $^{31}P\{^{1}H\}$ NMR spectrum (121.49 MHz, benzene-*d*$_6$, 298 K) of Rh{(*E*)-CH=CHPh}(C≡CPh)$_2${κ^3-*P,O,P*-[xant(P^{i}Pr$_2$)$_2$]} (**5**).

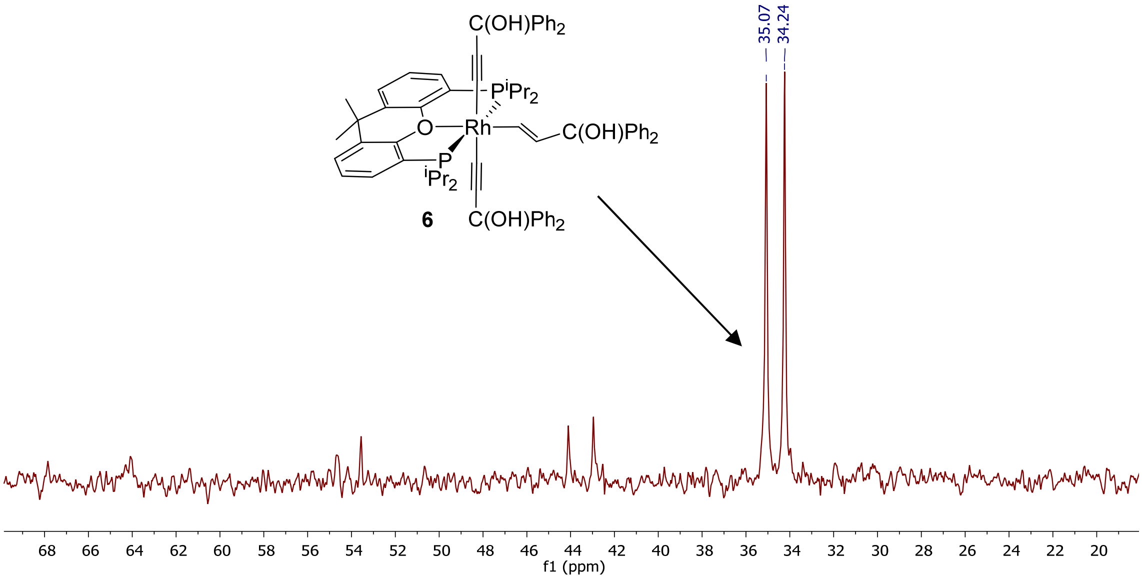

Figure S10. $^{31}P\{^{1}H\}$ NMR spectrum (121.4 MHz, benzene-d$_6$, 298 K) recorded during the catalytic homo-coupling of 1,1-diphenyl-2-propyn-1-ol (spectrum registered after 7 h of reaction).

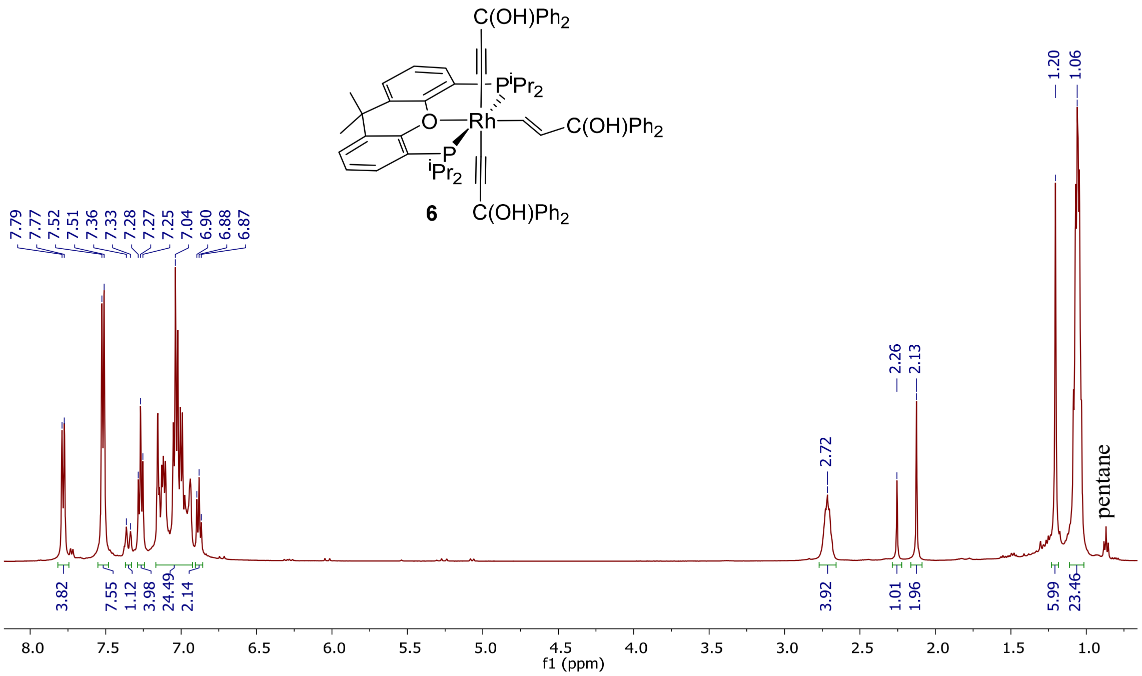

Figure S11. ^{1}H NMR spectrum (500.13 MHz, benzene-d_6, 298 K) of Rh{(*E*)-CH=CHC(OH)Ph$_2$}{C≡CC(OH)Ph$_2$}$_2${κ^3-*P,O,P*-[xant(P^{i}Pr$_2$)$_2$]} (**6**).

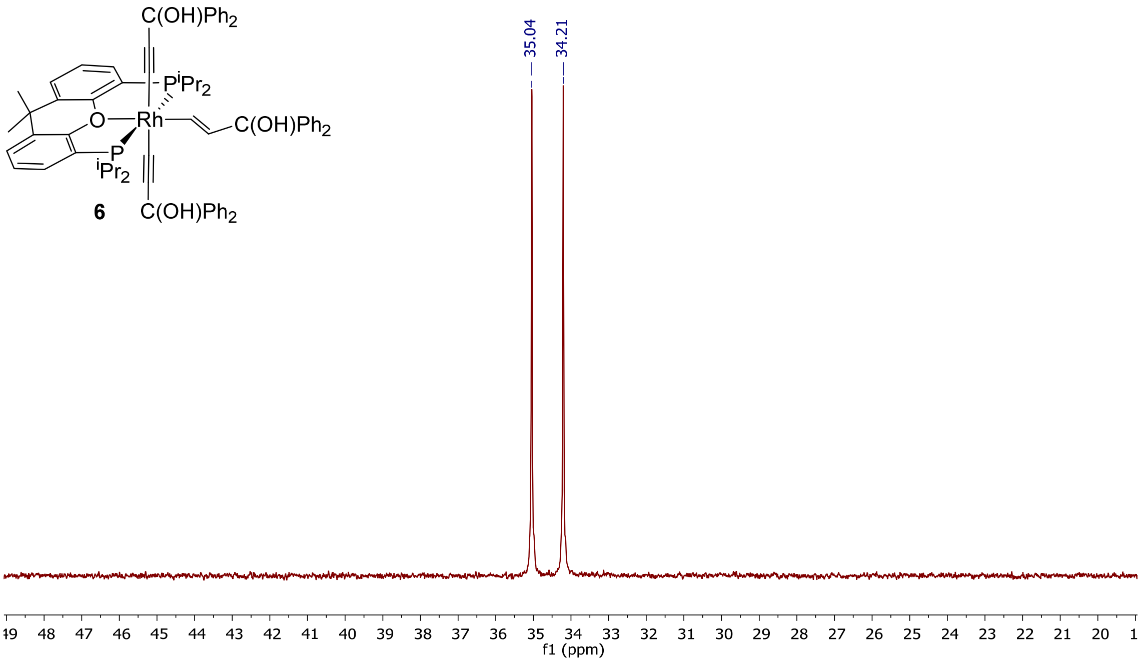

Figure S12. ^{31}P{^{1}H} NMR spectrum (202.46 MHz, benzene-d_6, 298 K) of Rh{(*E*)-CH=CHC(OH)Ph$_2$}{C≡CC(OH)Ph$_2$}$_2${κ^3-*P,O,P*-[xant(P^{i}Pr$_2$)$_2$]} (**6**).

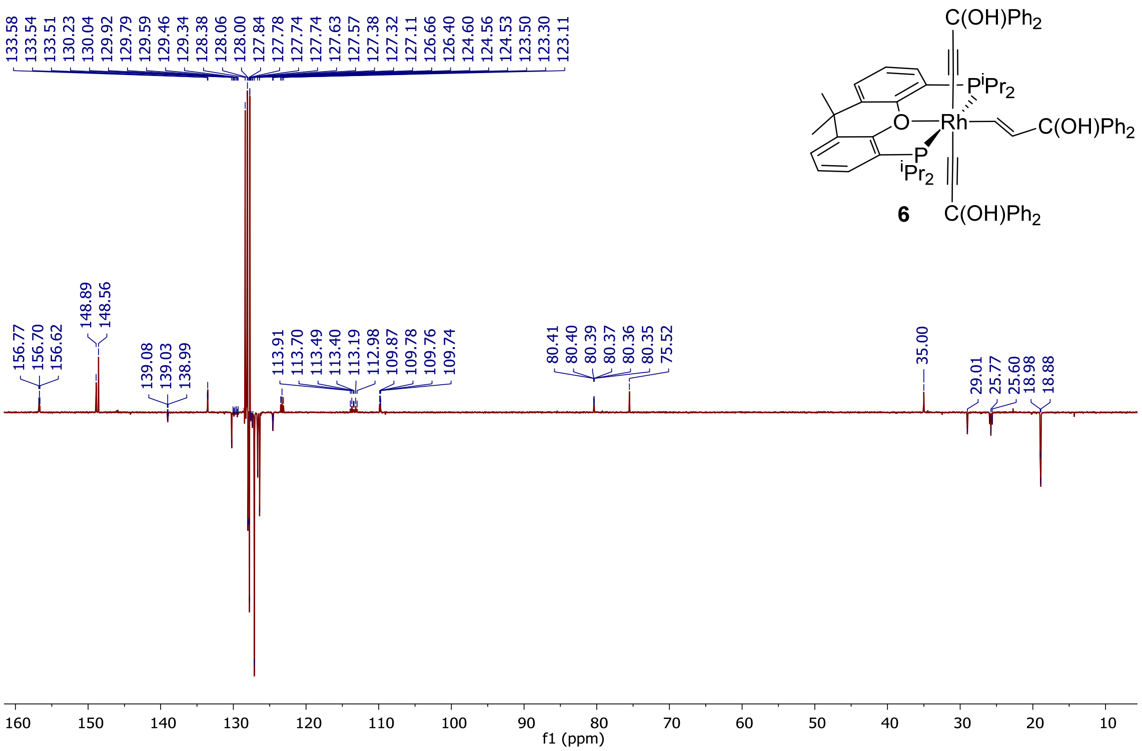

Figure S13. ${}^{13}C\{{}^{1}H\}$-apt NMR spectrum (75.48 MHz, benzene-d6, 298 K) of Rh{(*E*)-CH=CHC(OH)Ph2}{C≡CC(OH)Ph2}2{κ^3-*P,O,P*-[xant(P^{i}Pr2)2]} (**6**).

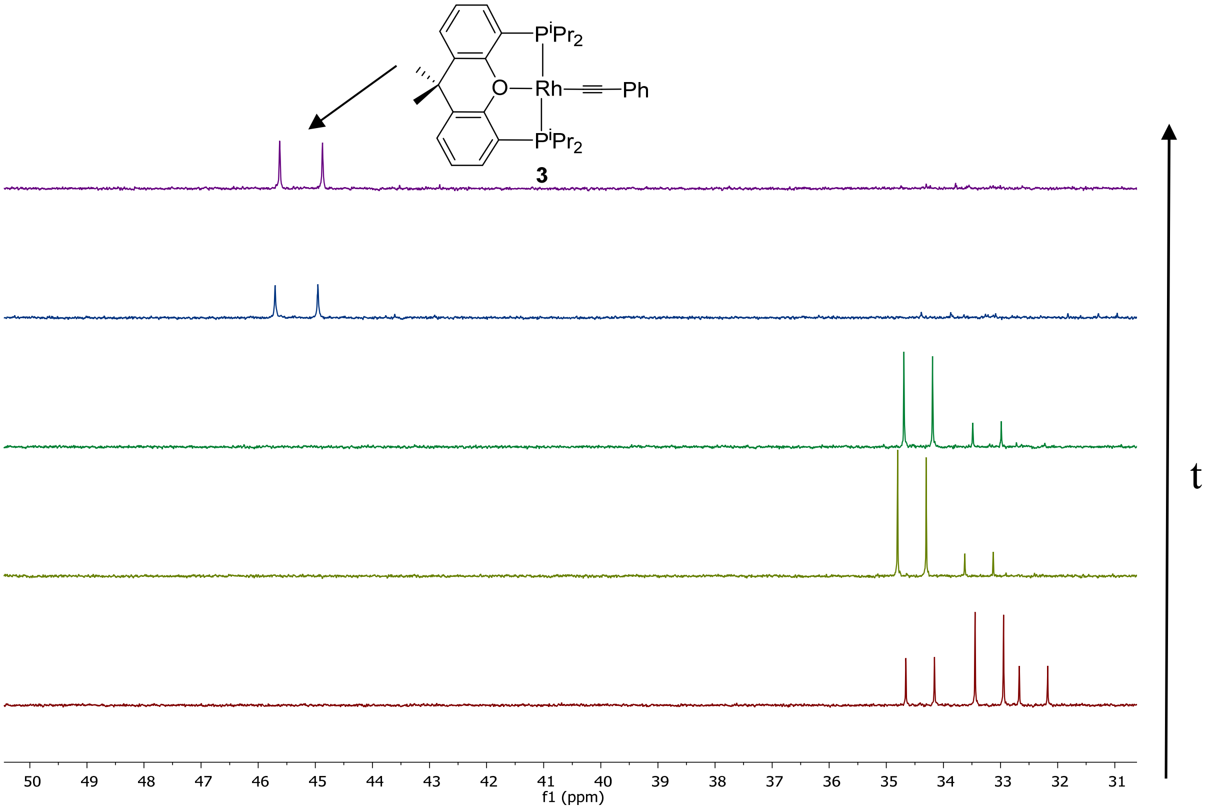

Figure S14. Stacked ${}^{31}P\{{}^{1}H\}$ NMR spectra (202.46 MHz, benzene-*d*6, 298 K) recorded during a catalytic run of cross-coupling between phenylacetylene and 1,1-diphenyl-2-propyn-1-ol.

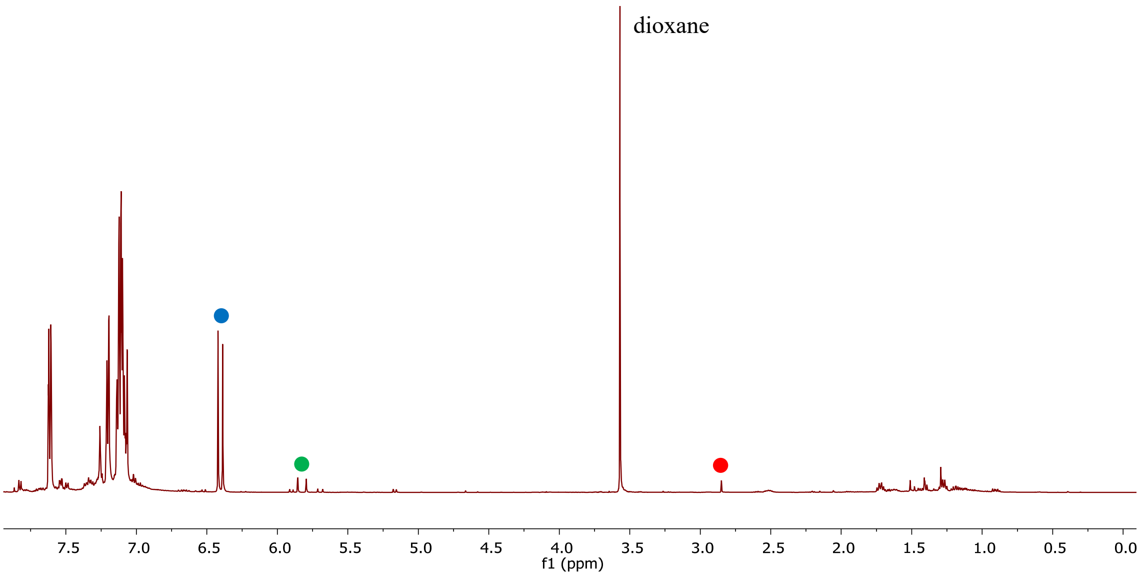

Figure S15. ^{1}H NMR spectrum (500.13 MHz, benzene-d_6, 298 K) of the crude reaction mixture of the dimerization of phenylacetylene. Characteristic resonances used for the calculation of the reaction conversion are marked as follows: blue spot, one olefinic proton of **a$_4$**; green spot, two olefinic protons of the head-to-tail dimer; red spot: HC≡ proton of phenylacetylene.

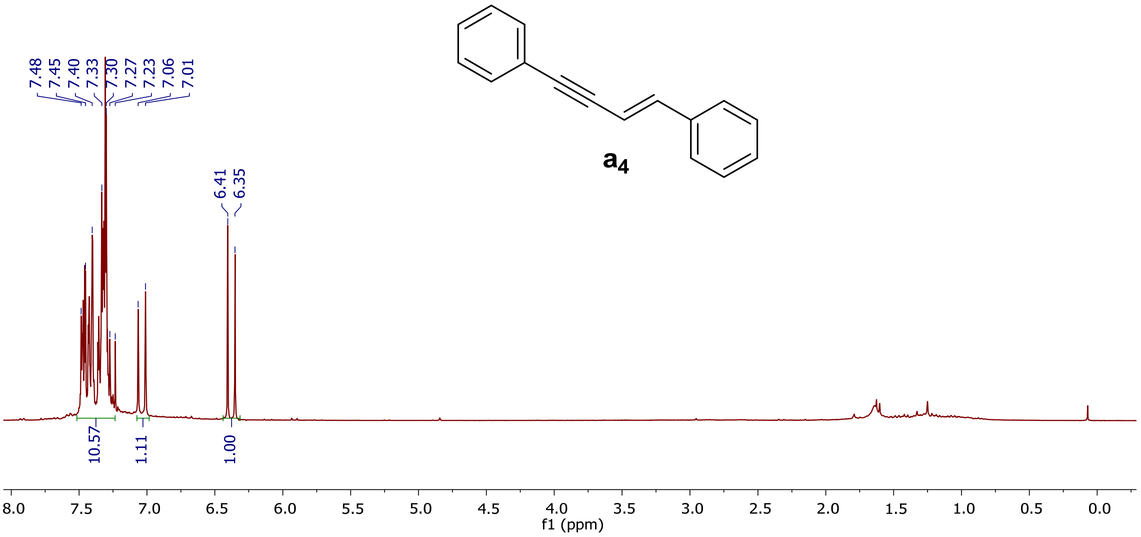

Figure S16. ^{1}H NMR spectrum (300.13 MHz, chloroform-d, 298 K) of **a$_4$**.

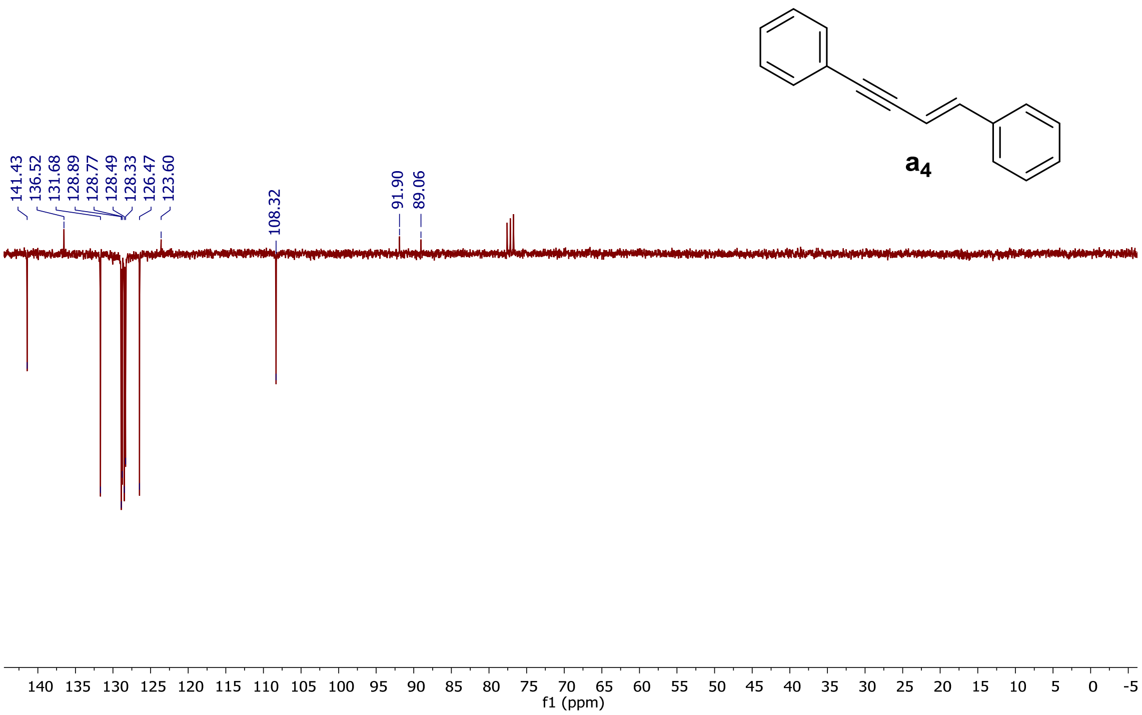

Figure S17. $^{13}C\{^1H\}$-apt NMR spectrum (75.48 MHz, chloroform-*d*, 298 K) of $\mathbf{a_4}$.

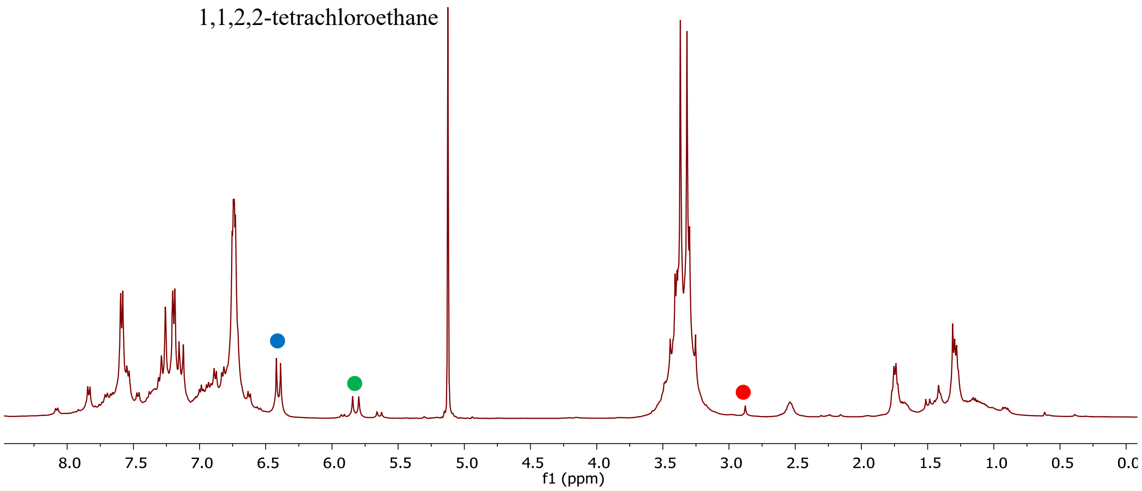

Figure S18. ^1H NMR spectrum (500.13 MHz, benzene-d_6, 298 K) of the crude reaction mixture of the dimerization of 4-methoxyphenylacetylene. Characteristic resonances used for the calculation of the reaction conversion are marked as follows: blue spot, one olefinic proton of $\mathbf{b_4}$; green spot, two olefinic protons of the head-to-tail dimer; red spot: HC≡ proton of 4-methoxyphenylacetylene.

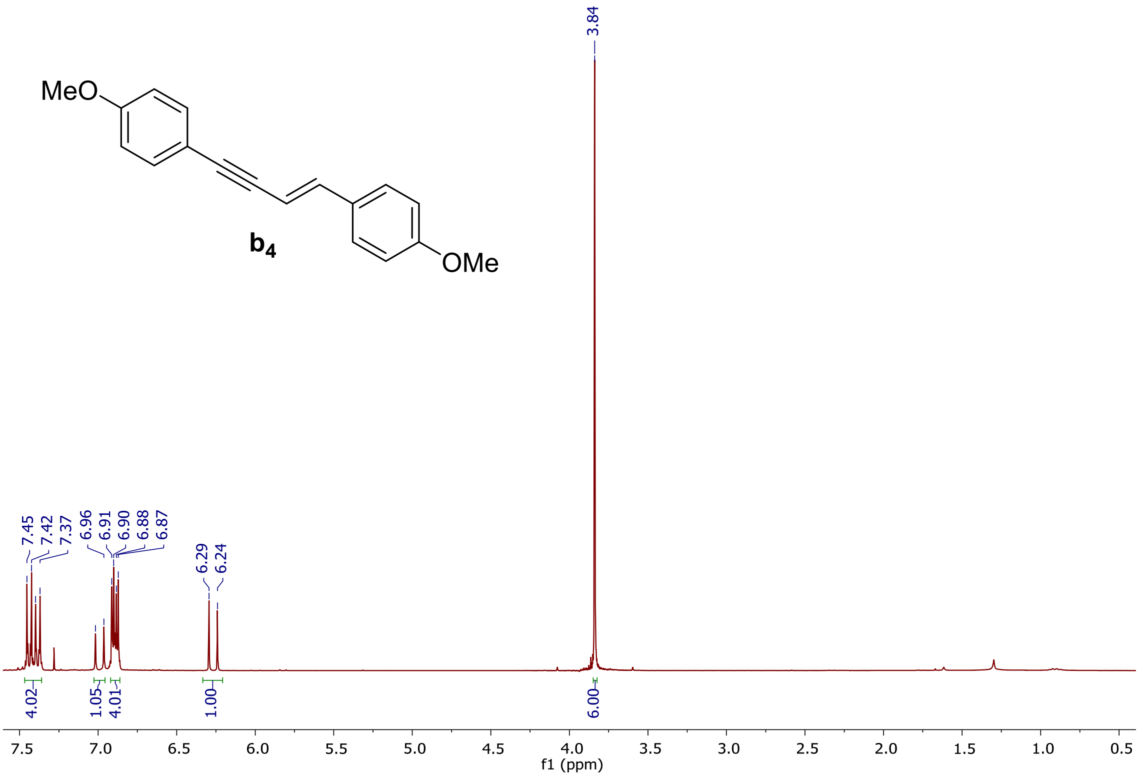

Figure S19. ^{1}H NMR spectrum (300.13 MHz, chloroform-*d*, 298 K) of **b4**.

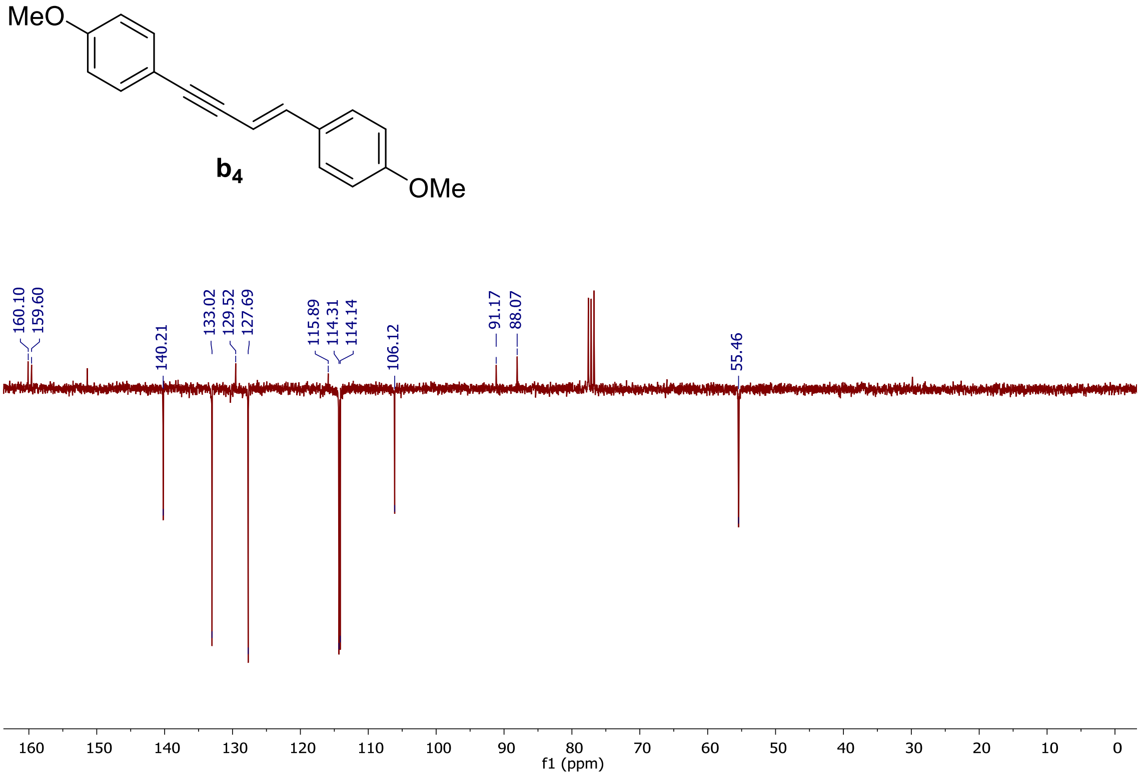

Figure S20. $^{13}C\{^{1}H\}$-apt NMR spectrum (75.48 MHz, chloroform-*d*, 298 K) of **b4**.

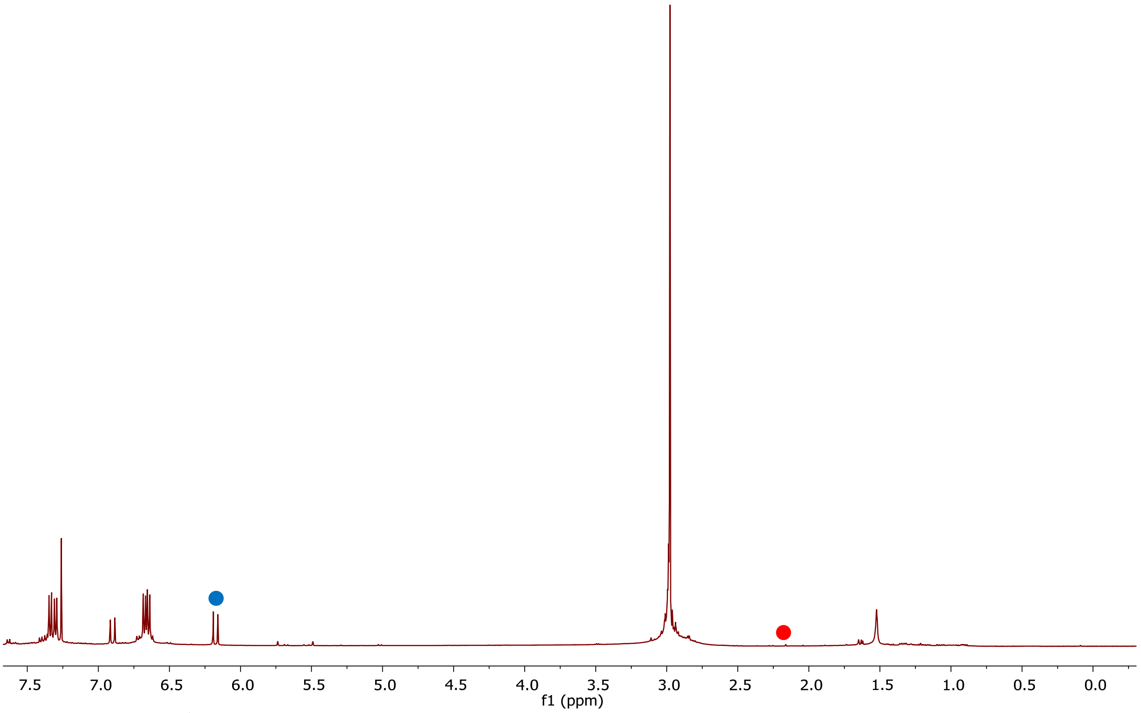

Figure S21. ^{1}H NMR spectrum (500.13 MHz, chloroform-*d*, 298 K) of the crude reaction mixture of the dimerization of 4-dimethylaminophenylacetylene to **c**$_4$. Characteristic resonances used for the calculation of the reaction conversion are marked as follows: blue spot, one olefinic proton of **c**$_4$; red spot: HC≡ proton of 4-dimethylaminophenylacetylene.

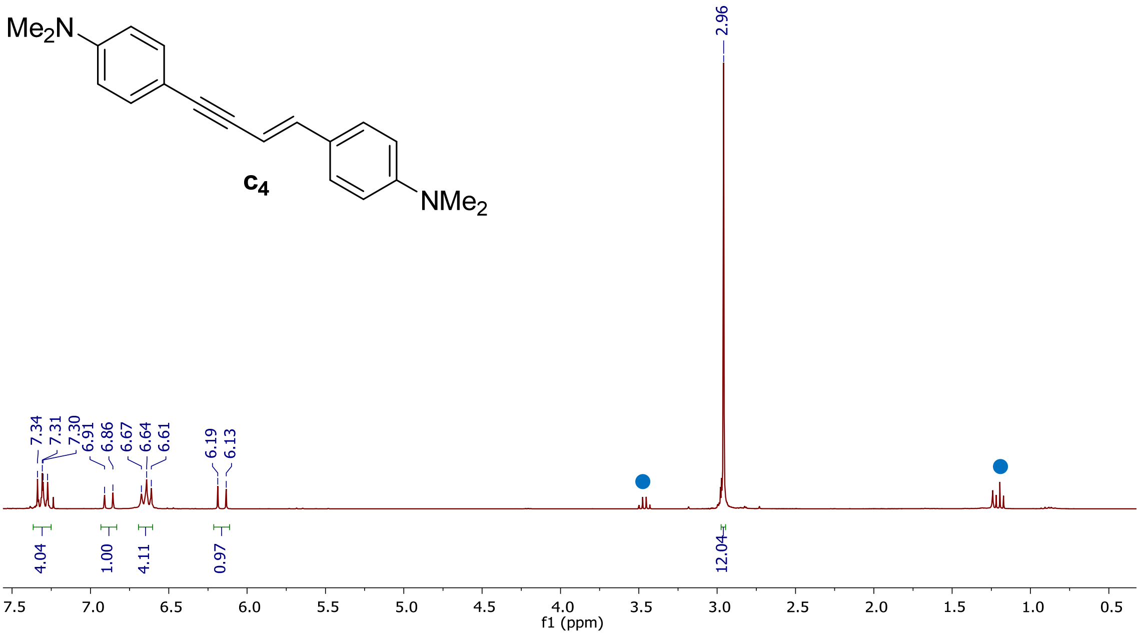

Figure S22. ^{1}H NMR spectrum (300.13 MHz, chloroform-*d*, 298 K) of **c**$_4$. Blue spots: Diethyl ether.

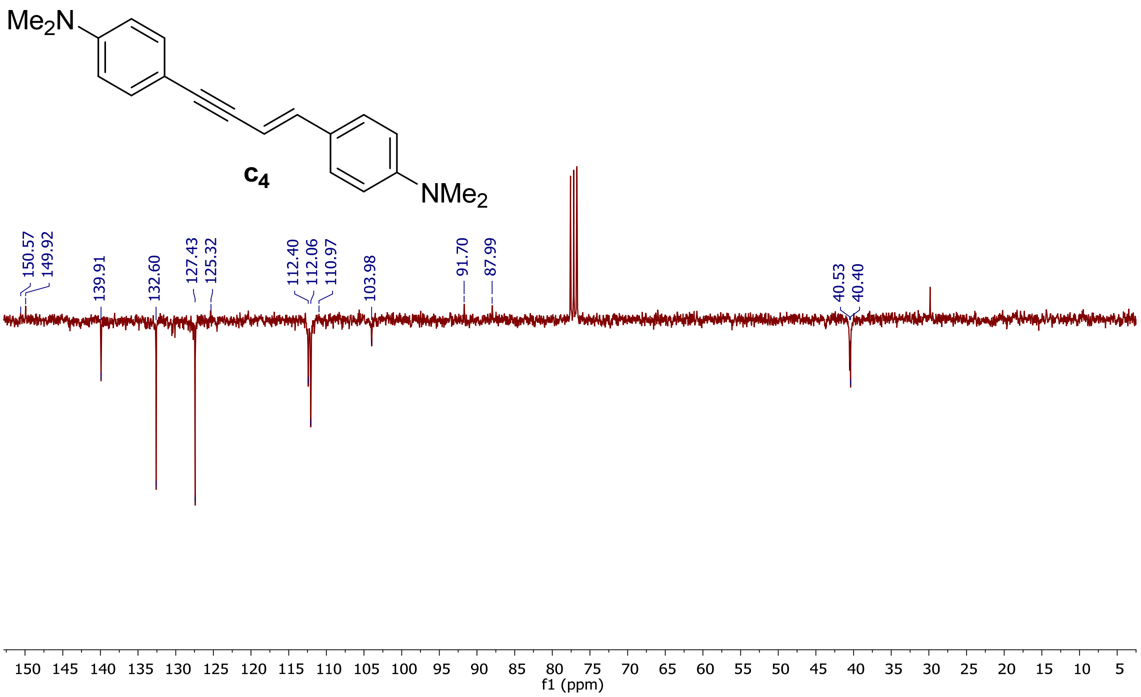

Figure S23. $^{13}C\{^{1}H\}$-apt NMR spectrum (75.48 MHz, chloroform-*d*, 298 K) of **c**$_4$.

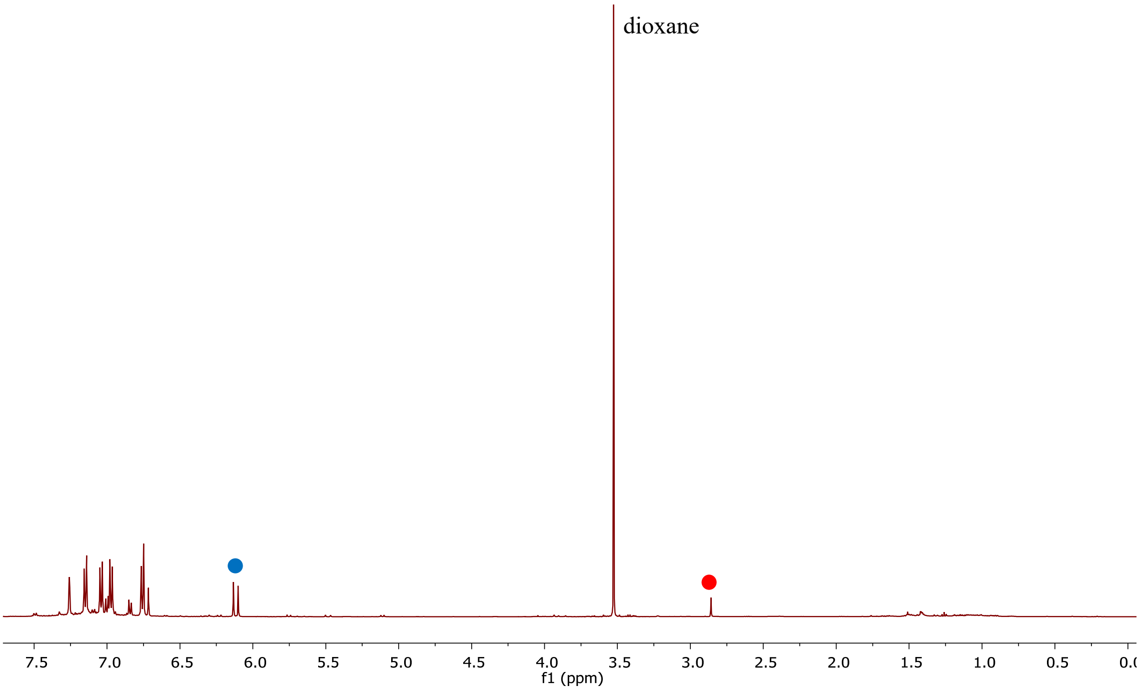

Figure S24. ^{1}H NMR spectrum (500.13 MHz, benzene-*d*$_6$, 298 K) of the crude reaction mixture of the dimerization of 4-ethynylbenzonitrile to **d**$_4$. Characteristic resonances used for the calculation of the reaction conversion are marked as follows: blue spot, one olefinic proton of **d**$_4$; red spot: HC≡ proton of 4-ethynylbenzonitrile.

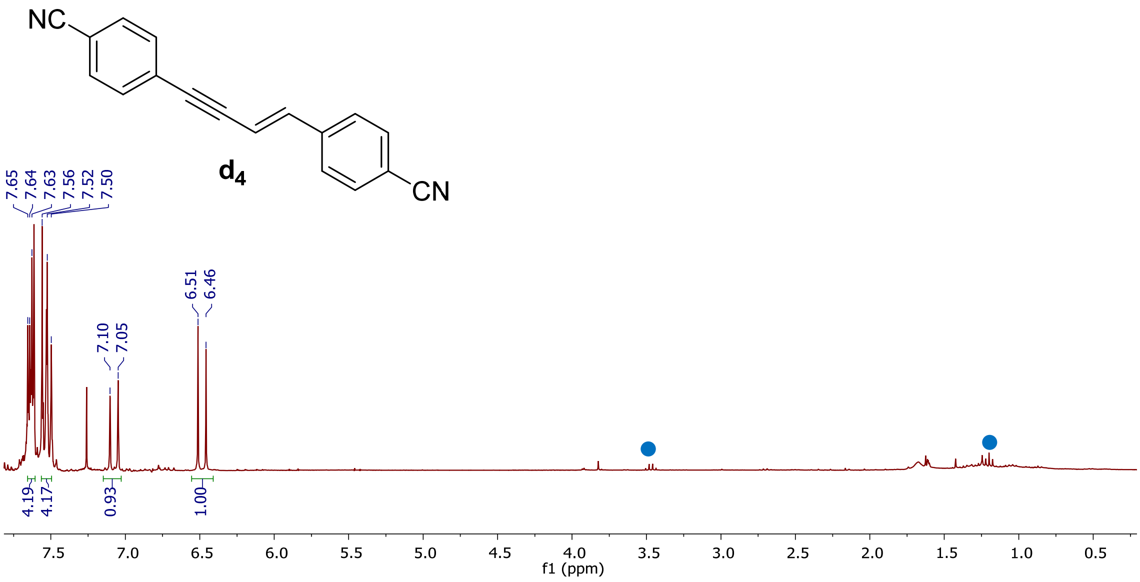

Figure S25. ^{1}H NMR spectrum (300.13 MHz, chloroform-*d*, 298 K) of **d4**. Blue spots: diethyl ether.

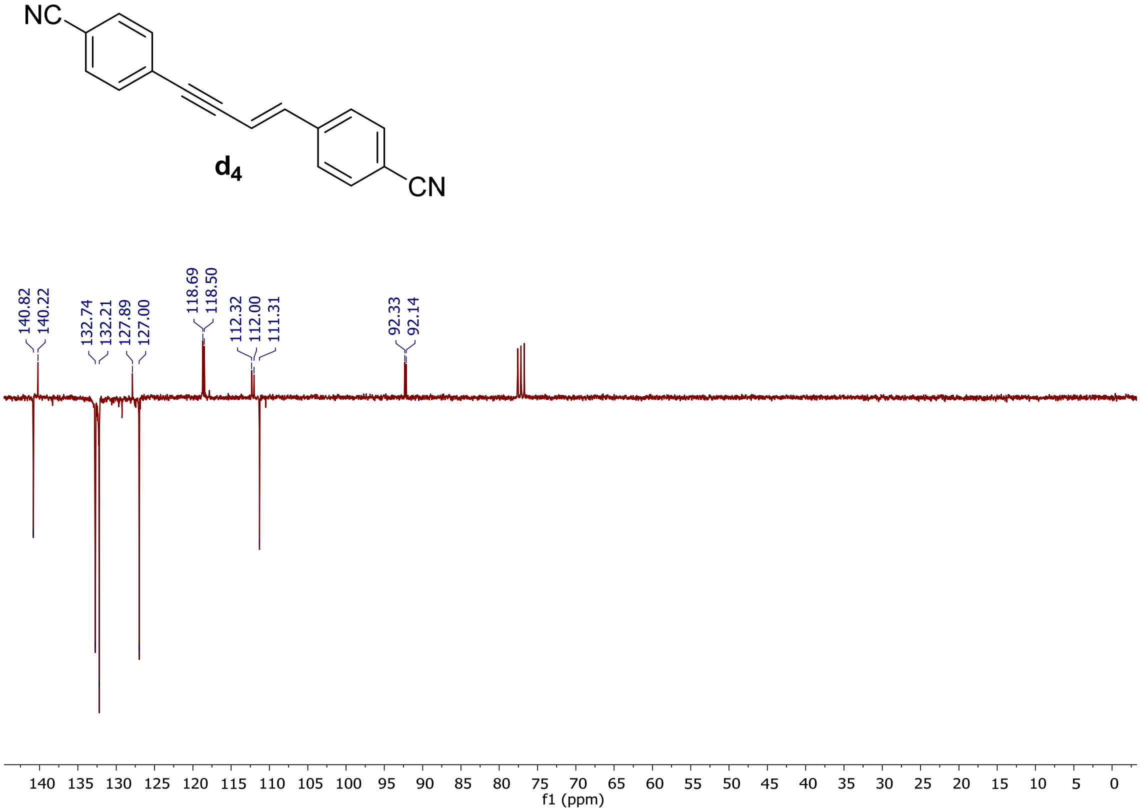

Figure S26. $^{13}C\{^{1}H\}$-apt NMR spectrum (75.48 MHz, chloroform-*d*, 298 K) of **d4**.

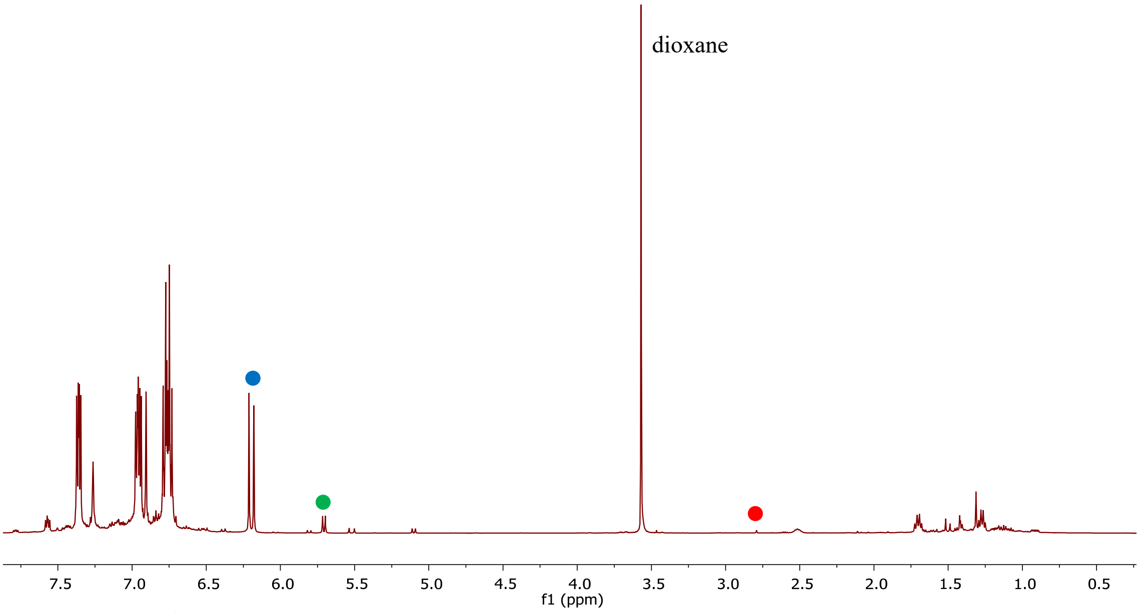

Figure S27. ^{1}H NMR spectrum (500.13 MHz, benzene-d_6, 298 K) of the crude reaction mixture of the dimerization of 1-ethynyl-4-fluorobenzene to **e**$_4$. Characteristic resonances used for the calculation of the reaction conversion are marked as follows: blue spot, one olefinic proton of **e**$_4$; green spot, two olefinic protons of the head-to-tail dimer; red spot: HC≡ proton of 1-ethynyl-4-fluorobenzene.

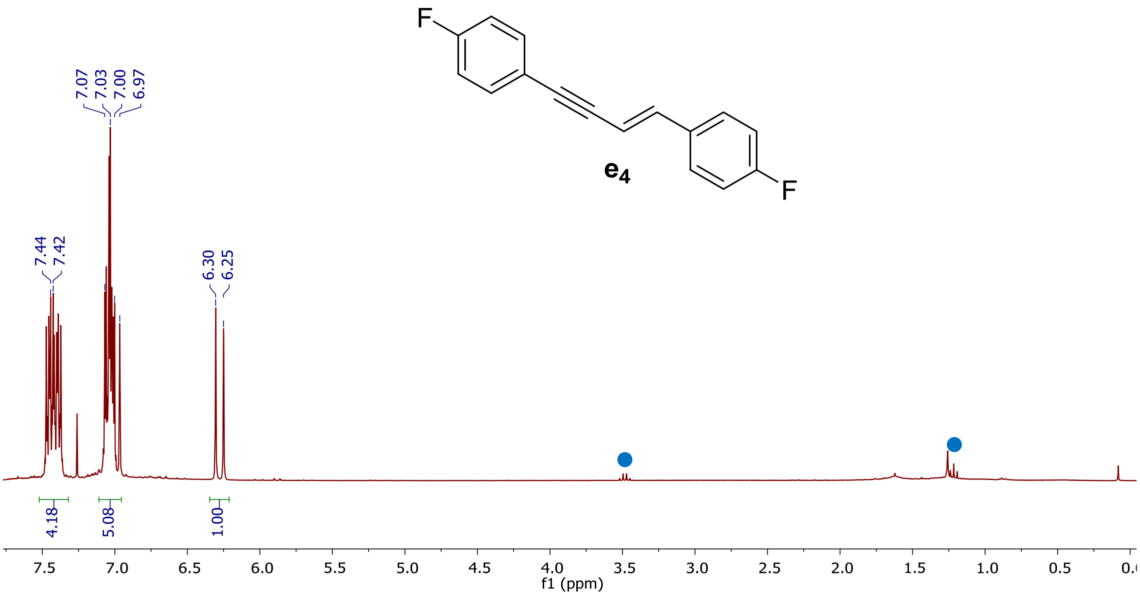

Figure S28. ^{1}H NMR spectrum (300.13 MHz, chloroform-d, 298 K) of **e**$_4$. Blue spots: diethyl ether.

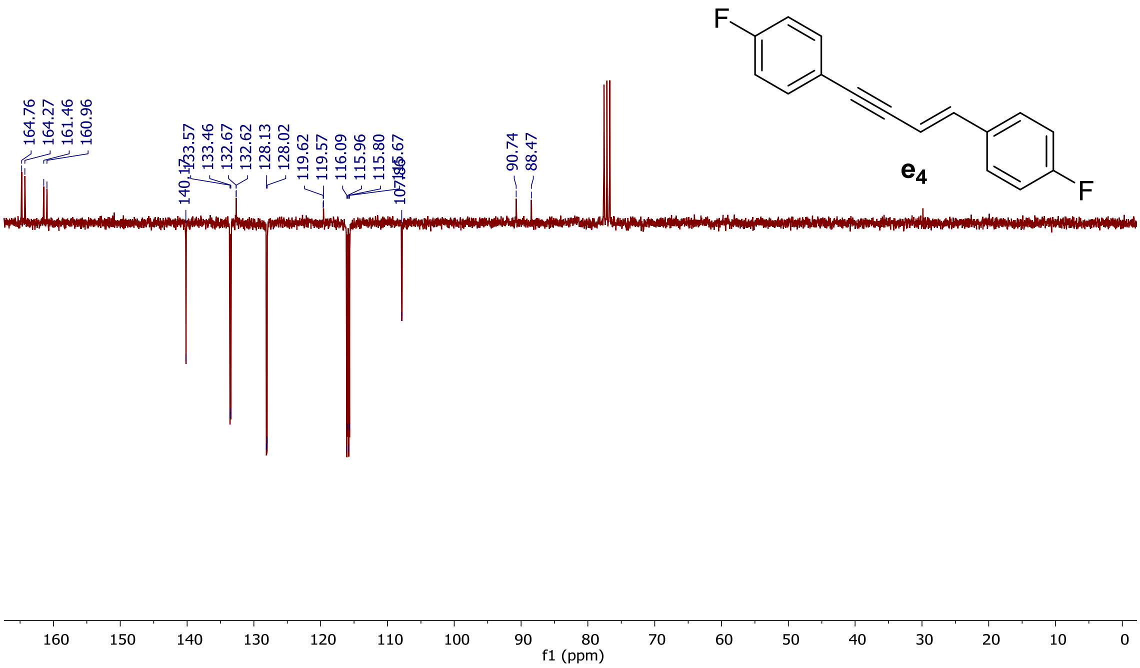

Figure S29. $^{13}C\{^{1}H\}$-apt NMR spectrum (75.48 MHz, chloroform-*d*, 298 K) of $\mathbf{e_4}$.

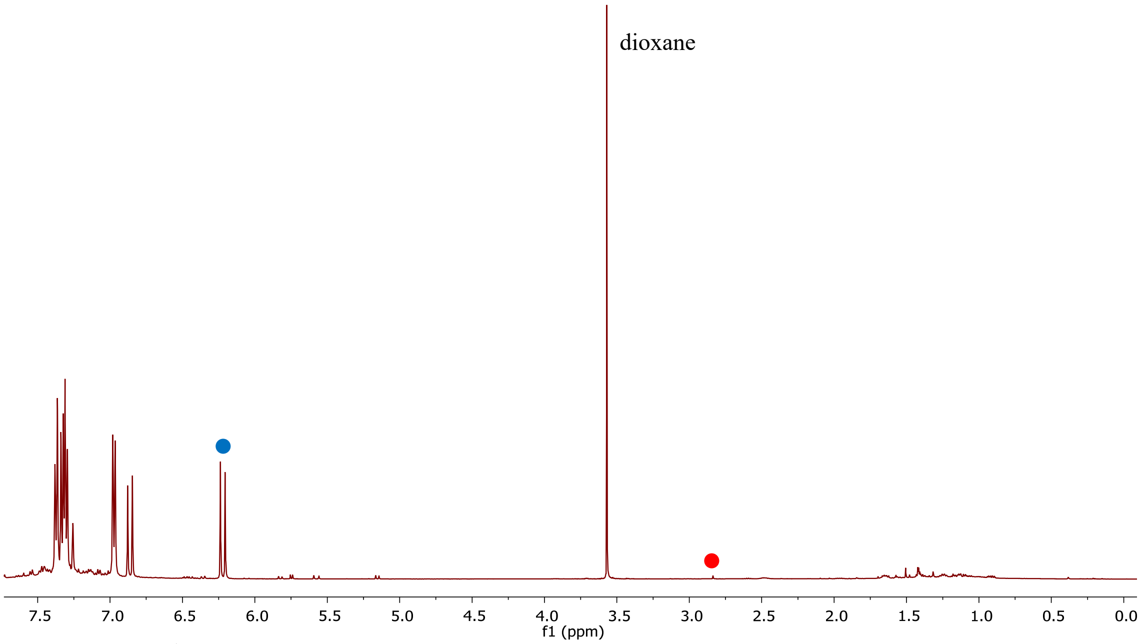

Figure S30. ^{1}H NMR spectrum (500.13 MHz, benzene-d_6, 298 K) of the crude reaction mixture of the dimerization of 4-trifluoromethylphenylacetylene to $\mathbf{f_4}$. Characteristic resonances used for the calculation of the reaction conversion are marked as follows: blue spot, one olefinic proton of $\mathbf{f_4}$; red spot: HC≡ proton of 4-trifluoromethylphenylacetylene.

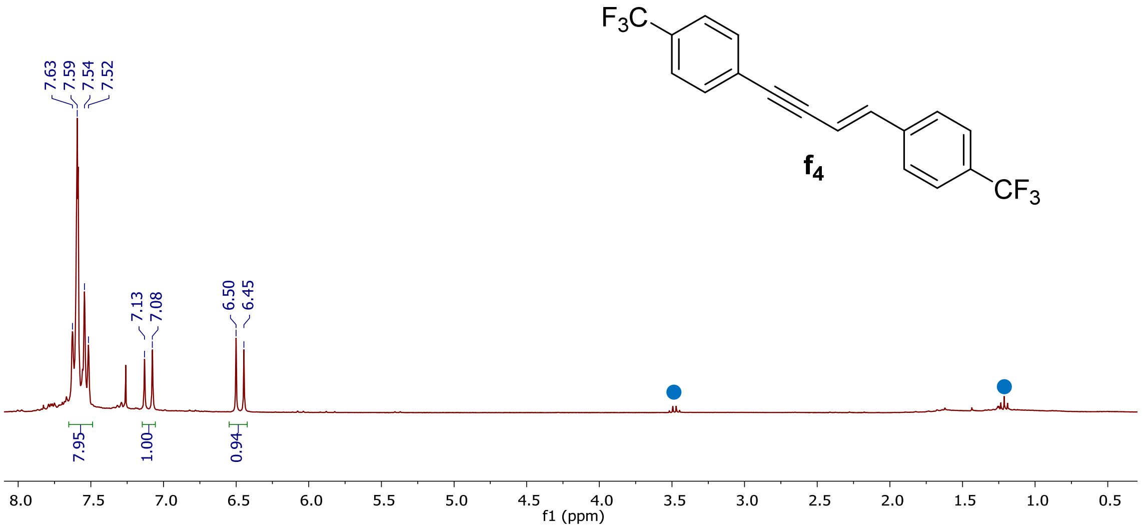

Figure S31. ^{1}H NMR spectrum (300.13 MHz, chloroform-*d*, 298 K) of **f4**. Blue spots: diethyl ether.

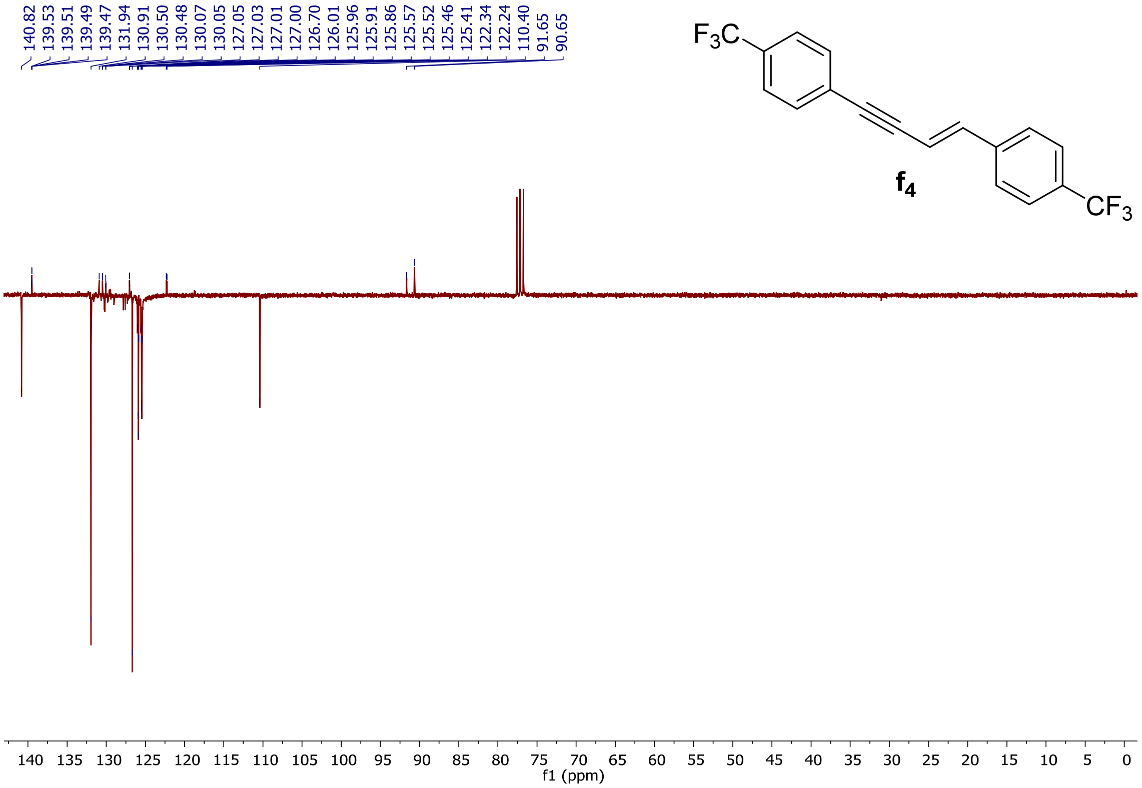

Figure S32. $^{13}C\{^{1}H\}$-apt NMR spectrum (75.48 MHz, chloroform-*d*, 298 K) of **f4**.

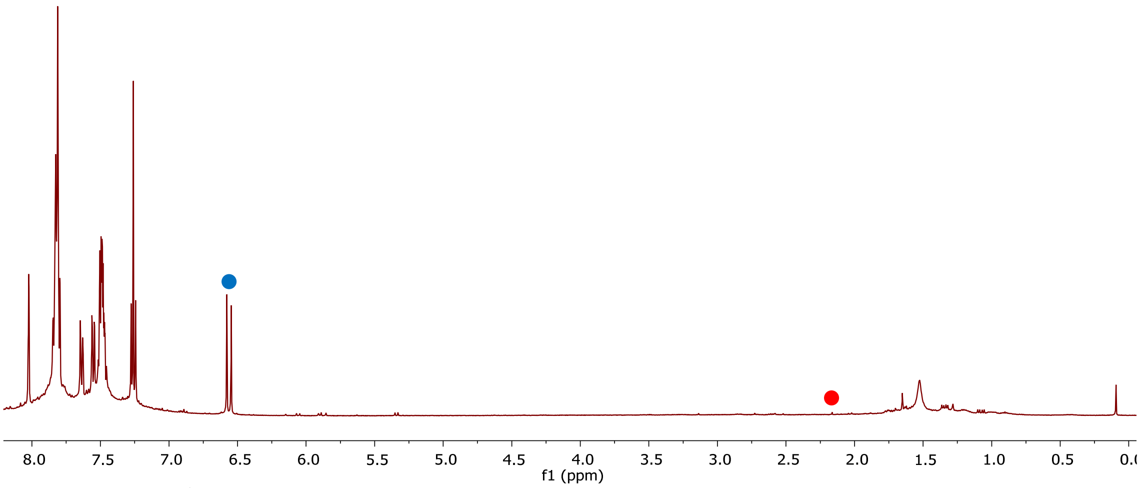

Figure S33. ^{1}H NMR spectrum (500.13 MHz, chloroform-*d*, 298 K) of the crude reaction mixture of the dimerization of 2-ethynylnaphthalene to **g4**. Characteristic resonances used for the calculation of the reaction conversion are marked as follows: blue spot, one olefinic proton of **g4**; red spot: HC≡ proton of 2-ethynylnaphthalene.

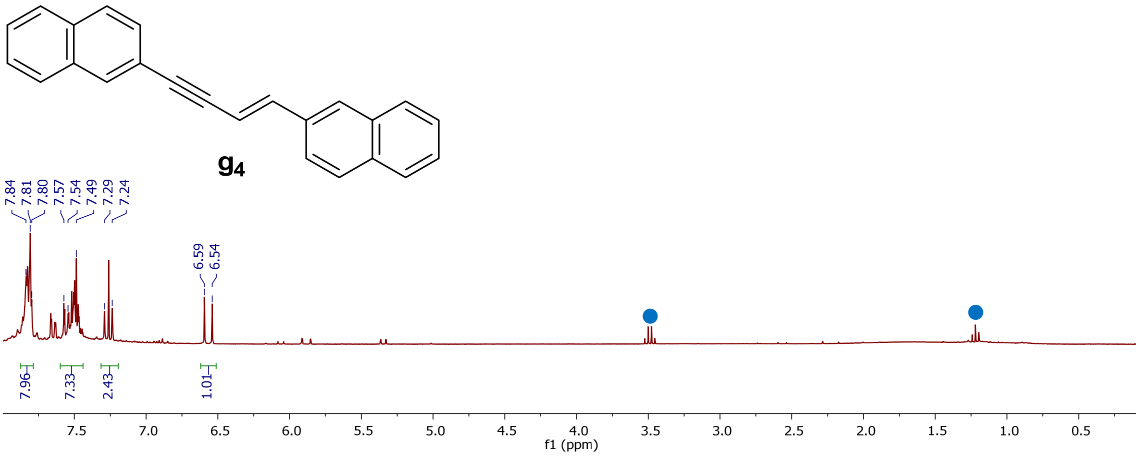

Figure S34. ^{1}H NMR spectrum (300.13 MHz, chloroform-*d*, 298 K) of **g4**. Blue spots: diethyl ether.

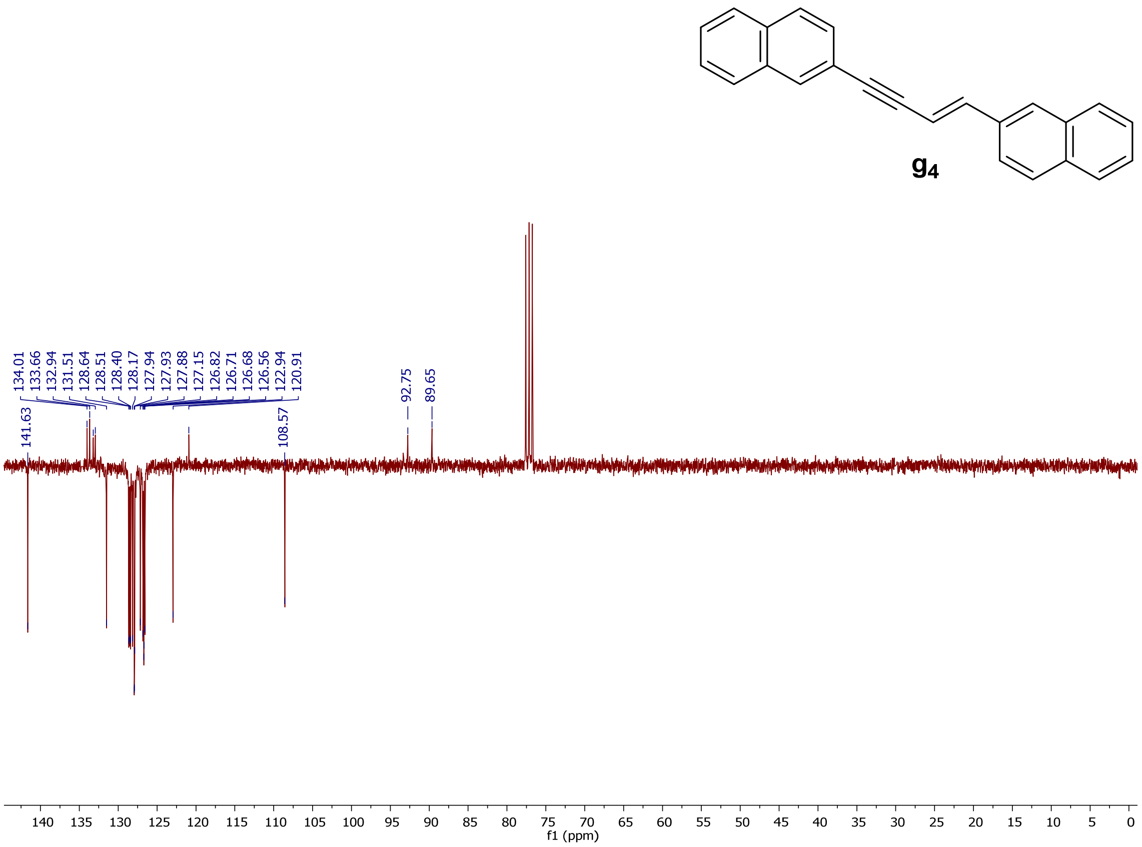

Figure S35. $^{13}C\{^{1}H\}$-apt NMR spectrum (75.48 MHz, chloroform-*d*, 298 K) of **g**$_4$.

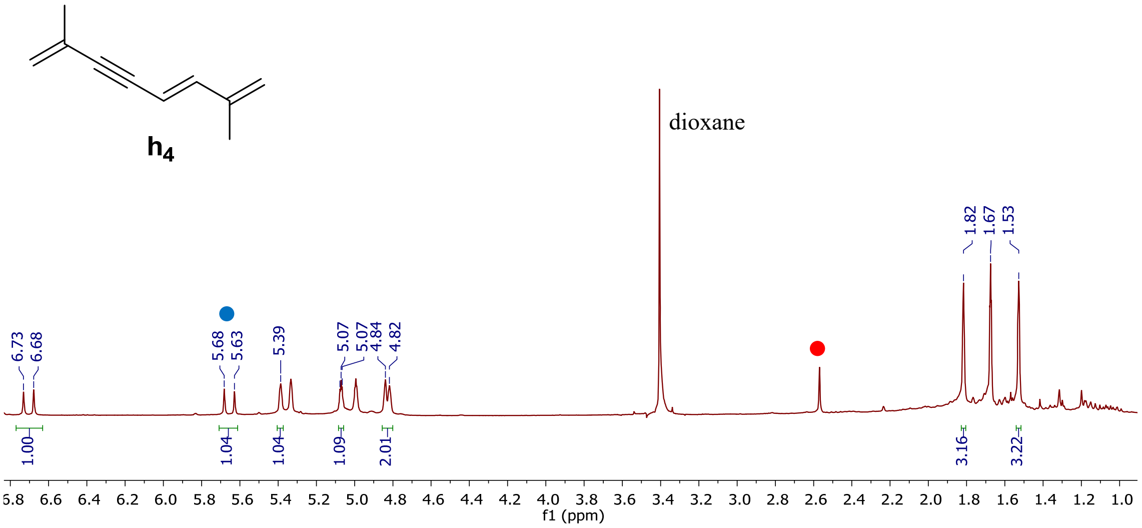

Figure S36. ^{1}H NMR spectrum (500.13 MHz, benzene-*d*$_6$, 298 K) of the crude reaction mixture of the dimerization of 2-methyl-1-buten-3-yne to **h**$_4$. Characteristic resonances used for the calculation of the reaction conversion are marked as follows: blue spot, one of the trans disposed olefinic protons of **h**$_4$; red spot: HC≡ proton of 2-methyl-1-buten-3-yne.

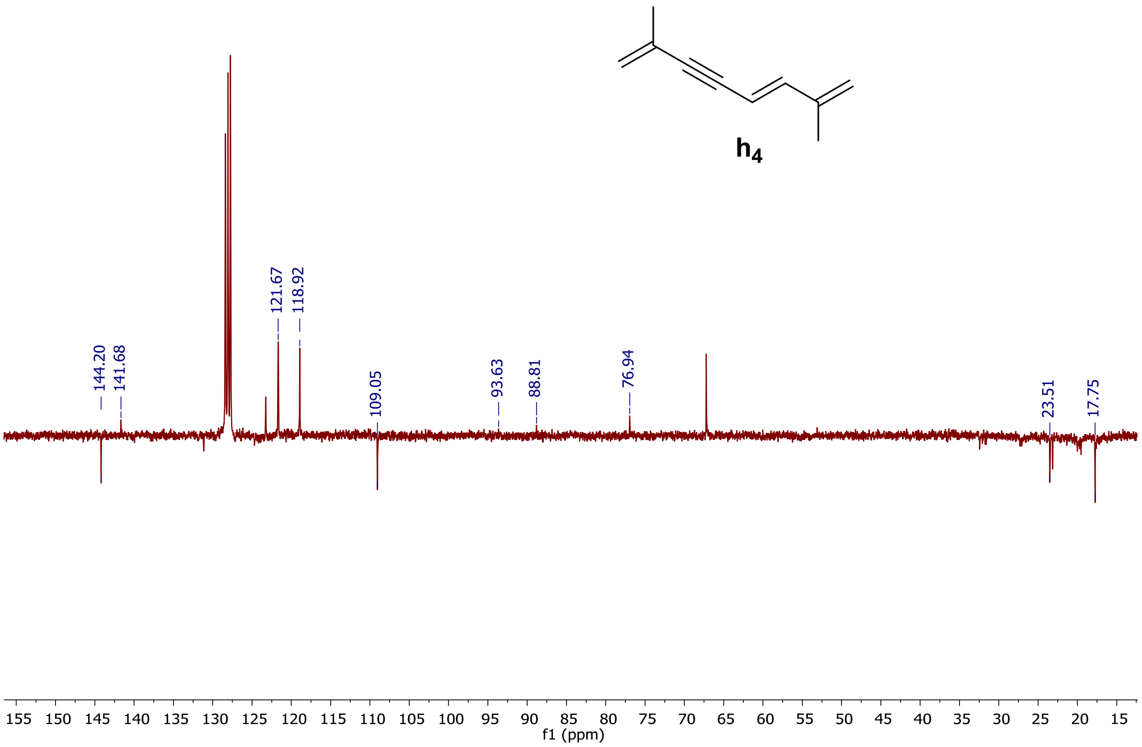

Figure S37. $^{13}C\{^{1}H\}$-apt NMR spectrum (75.48 MHz, benzene-d_6, 298 K) of the crude reaction mixture of the dimerization of 2-methyl-1-buten-3-yne to $\mathbf{h_4}$.

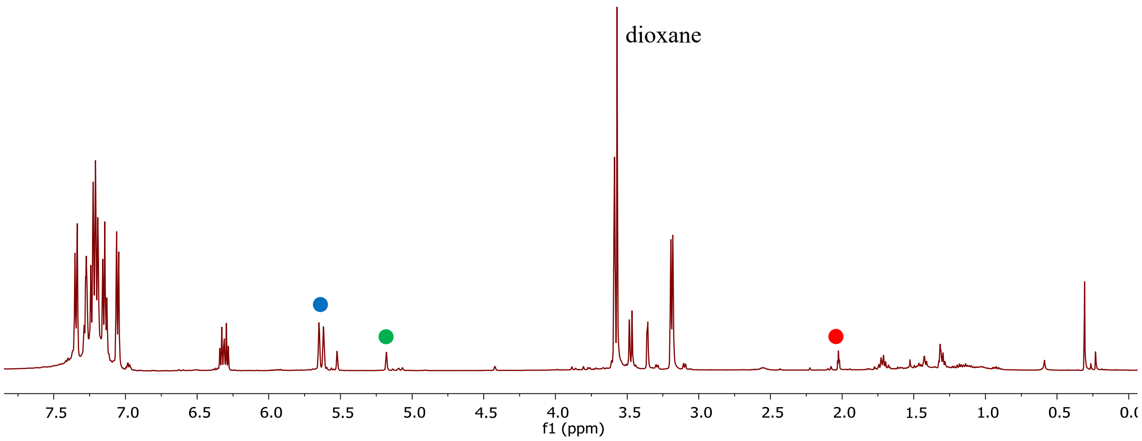

Figure S38. ^{1}H NMR spectrum (500.13 MHz, benzene-d_6, 298 K) of the crude reaction mixture of the dimerization of 3-phenyl-1-propyne to $\mathbf{i_4}$. Characteristic resonances used for the calculation of the reaction conversion are marked as follows: blue spot, one olefinic proton of $\mathbf{i_4}$; green spot, one olefinic proton of the head-to-tail dimer; red spot: HC≡ proton of 3-phenyl-1-propyne.

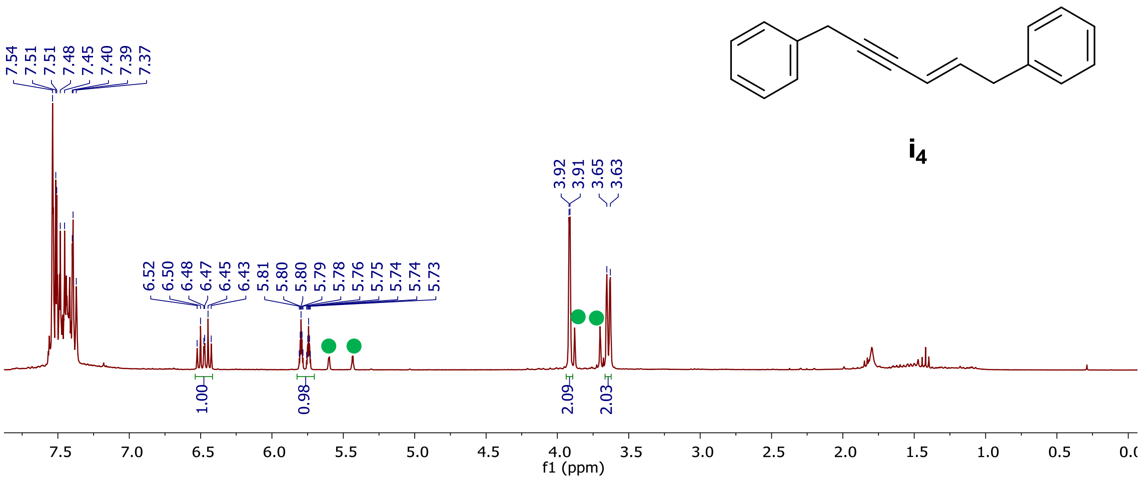

Figure S39. ^{1}H NMR spectrum (300.13 MHz, chloroform-*d*, 298 K) of **i$_4$**. Green spots: olefinic and benzylic protons of the head-to-tail dimer.

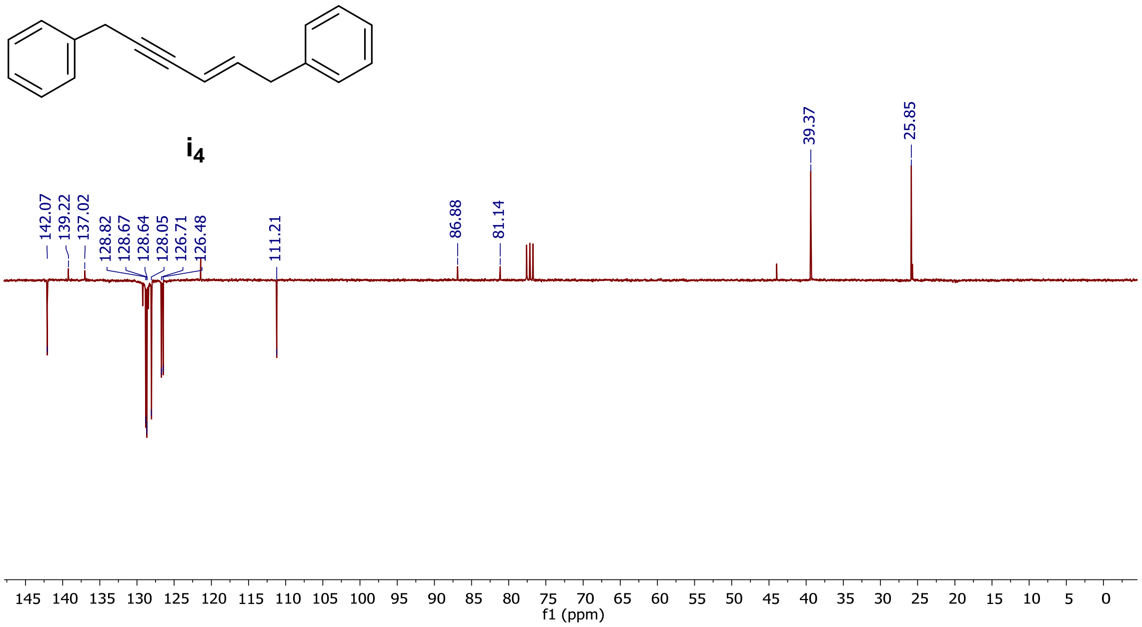

Figure S40. ^{13}C{^{1}H}-apt NMR spectrum (75.48 MHz, chloroform-*d*, 298 K) of **i$_4$**.

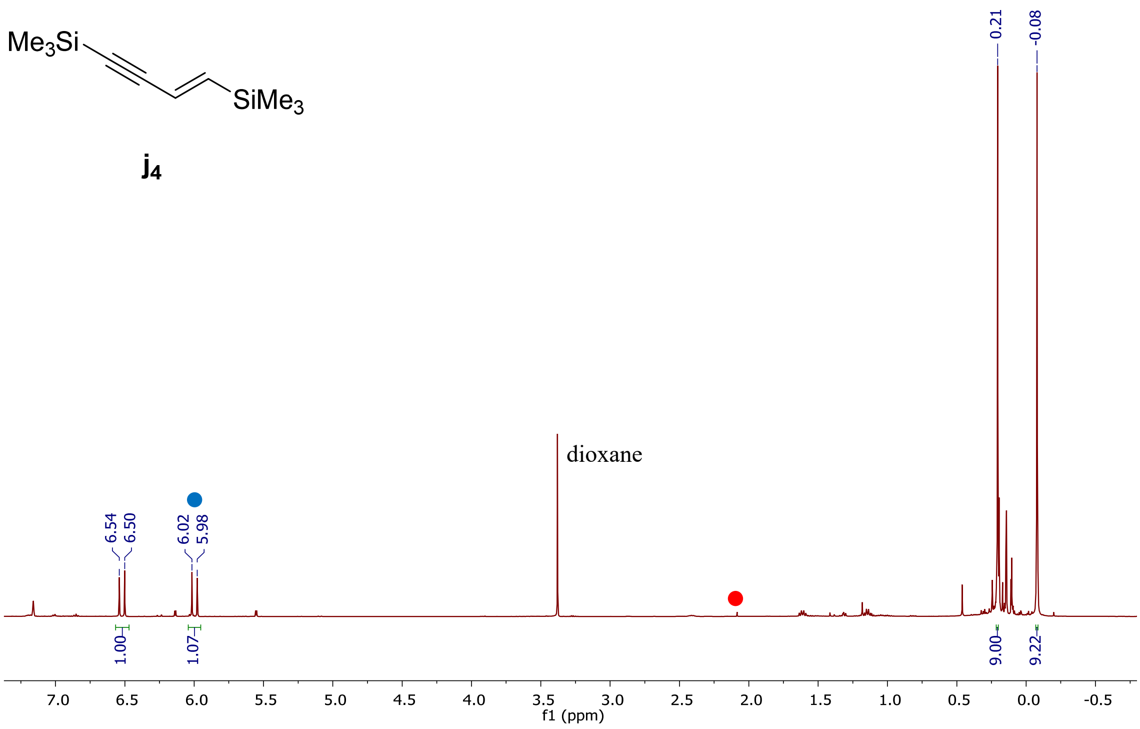

Figure S41. ^{1}H NMR spectrum (500.13 MHz, benzene-d_6, 298 K) of the crude reaction mixture of the dimerization of trimethylsilylacetylene to **j$_4$**. Characteristic resonances used for the calculation of the reaction conversion are marked as follows: blue spot, one olefinic proton of **j$_4$**; red spot: HC≡ proton of trimethylsilylacetylene.

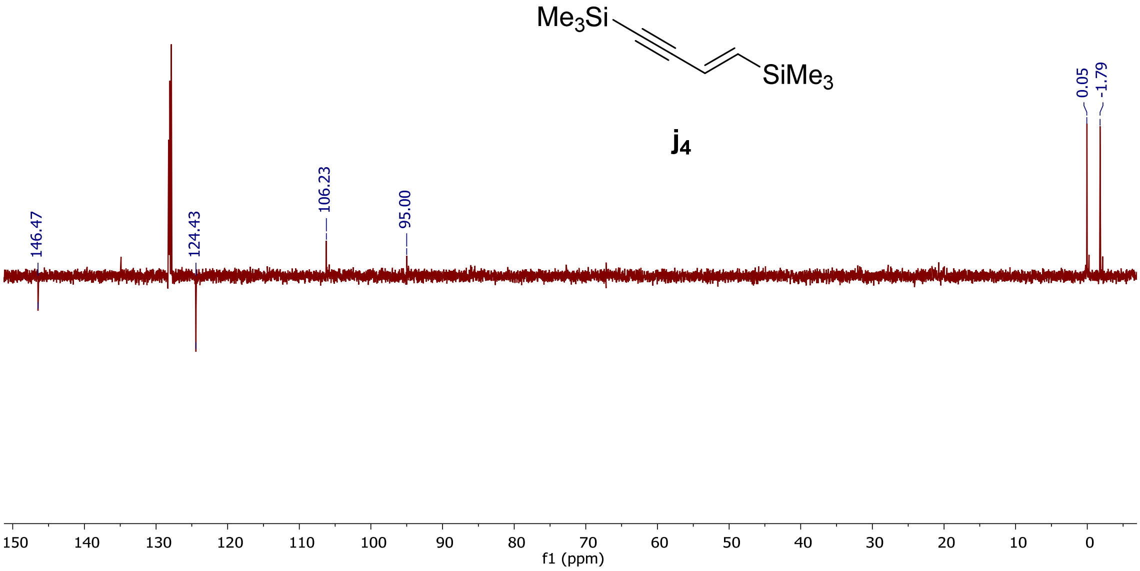

Figure S42. ^{13}C{^{1}H}-apt NMR spectrum (125.77 MHz, benzene-d_6, 298 K) of the crude reaction mixture of the dimerization of **j$_4$**.

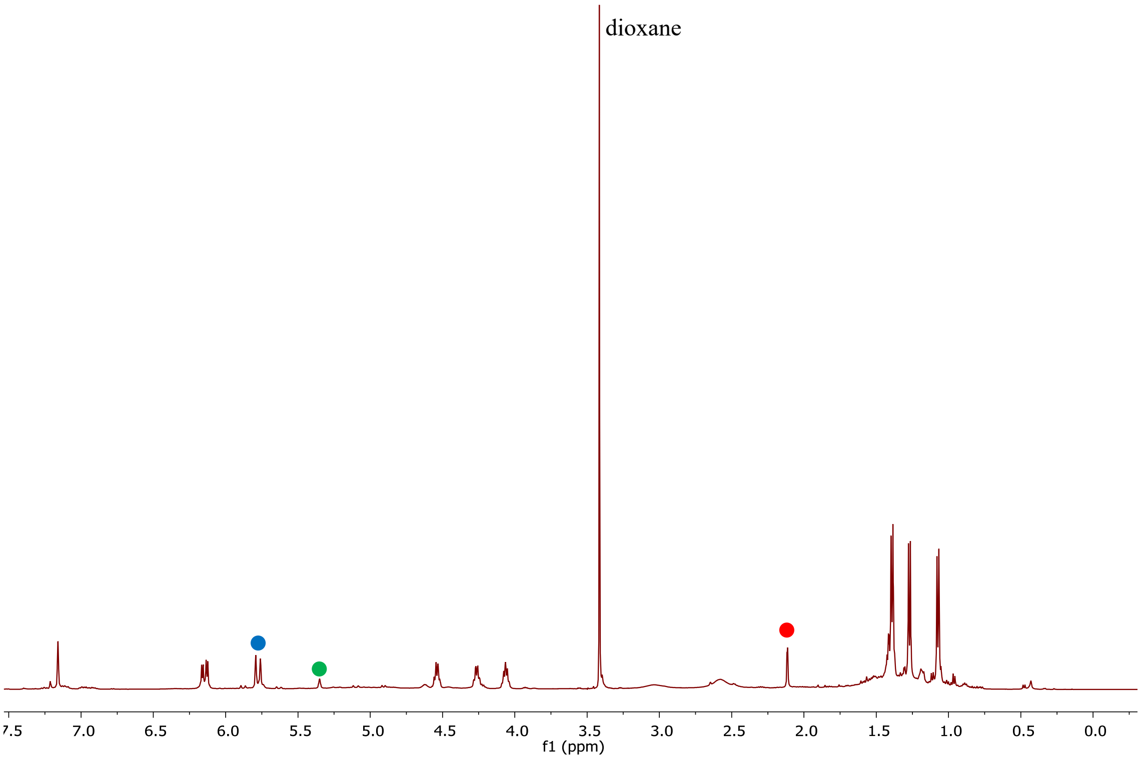

Figure S43. ^{1}H NMR spectrum (500 MHz, benzene-d_6, 298 K) of the crude reaction mixture of the dimerization of 3-butyn-2-ol to $\mathbf{a_5}$. Characteristic resonances used for the calculation of the reaction conversion are marked as follows: blue spot, one olefinic proton of $\mathbf{a_5}$; green spot, one olefinic proton of the head-to-tail dimer; red spot: HC≡ proton of 3-butyn-2-ol.

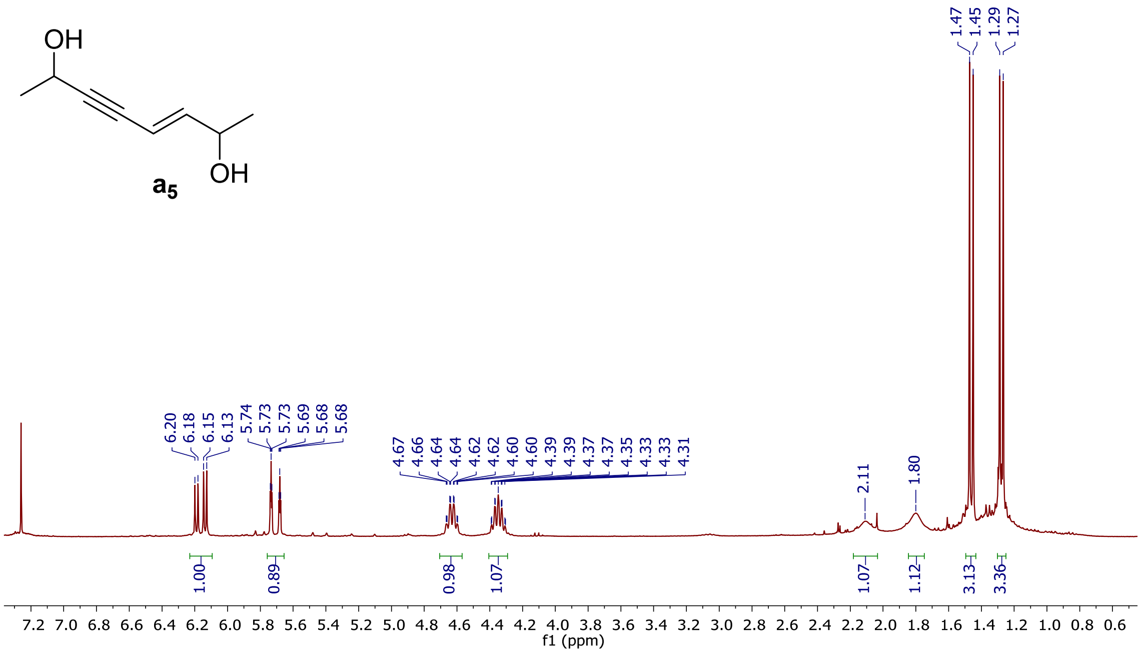

Figure S44. ^{1}H NMR spectrum (300.13 MHz, chloroform-d, 298 K) of $\mathbf{a_5}$.

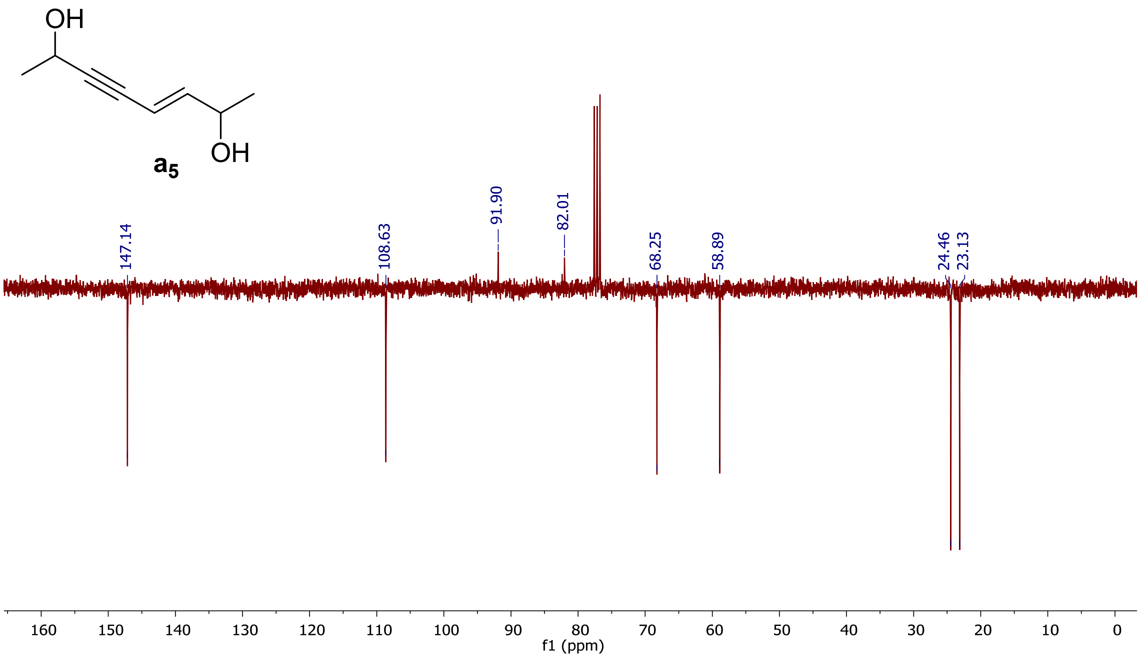

Figure S45. $^{13}C\{^{1}H\}$-apt NMR spectrum (75.48 MHz, chloroform-*d*, 298 K) of $\mathbf{a_5}$.

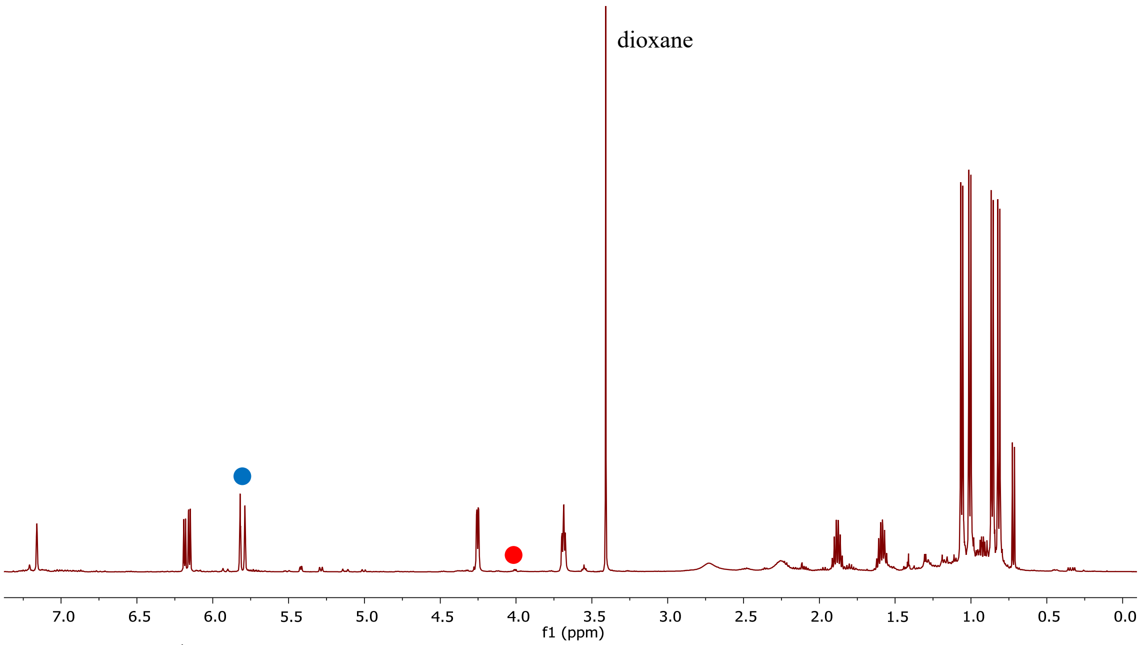

Figure S46. ^{1}H NMR spectrum (500 MHz, benzene-d$_6$, 298 K) of the crude reaction mixture of the dimerization of 4-methyl-1-pentyn-3-ol to $\mathbf{b_5}$. Characteristic resonances used for the calculation of the reaction conversion are marked as follows: blue spot, one olefinic proton of $\mathbf{b_5}$; red spot: C*H*-OH proton of 4-methyl-1-pentyn-3-ol.

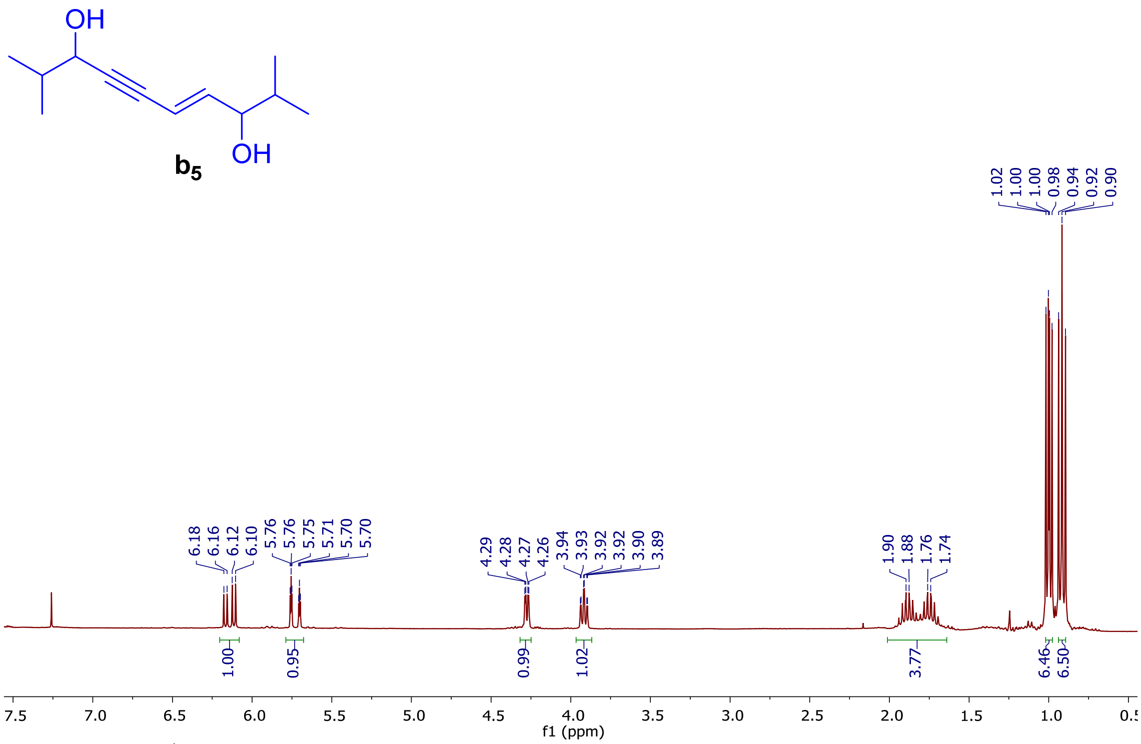

Figure S47. ^{1}H NMR spectrum (300.13 MHz, chloroform-*d*, 298 K) of **b$_5$**.

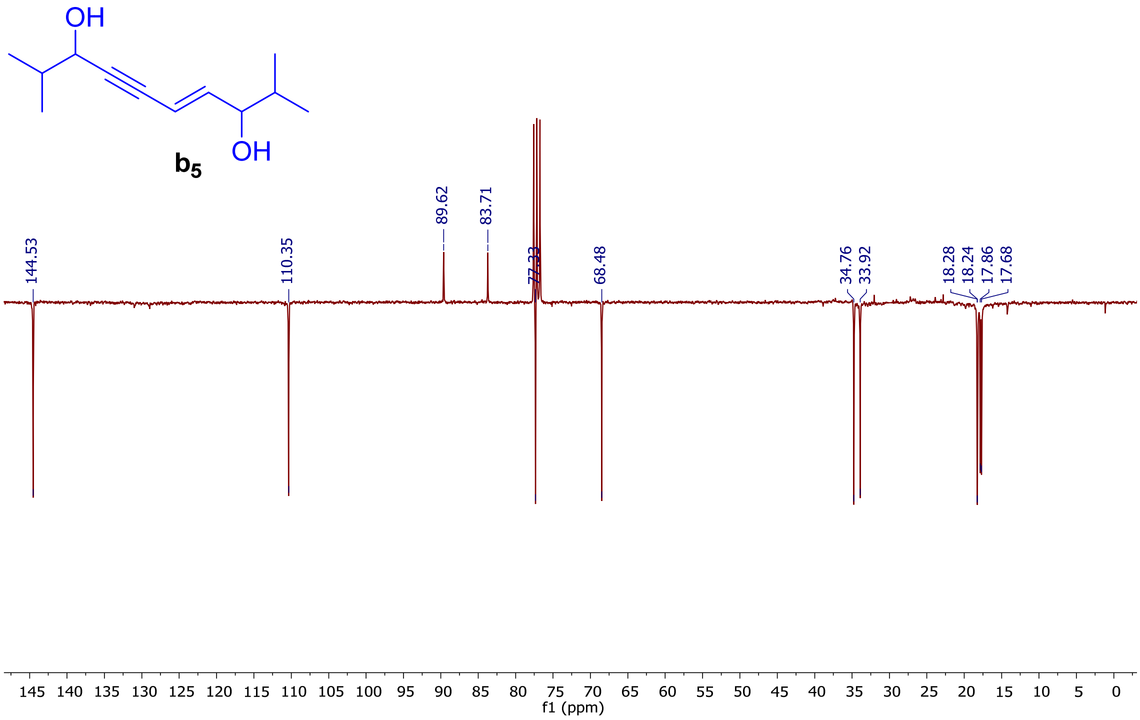

Figure S48. ^{13}C{^{1}H}-apt NMR spectrum (75.48 MHz, chloroform-*d*, 298 K) of **b$_5$.**

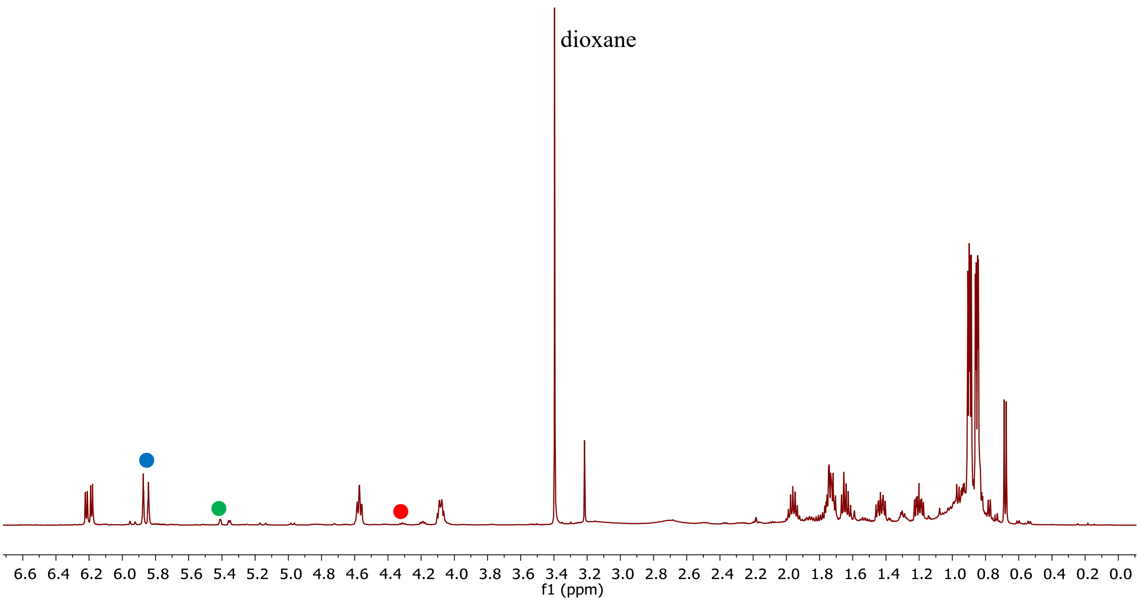

Figure S49. ^{1}H NMR spectrum (500 MHz, benzene-d$_6$, 298 K) of the crude reaction mixture of the dimerization of 5-methyl-1-hexyn-3-ol to **c$_5$**. Characteristic resonances used for the calculation of the reaction conversion are marked as follows: blue spot, one olefinic proton of **c$_5$**; green spot, one olefinic proton of the head-to-tail dimer; red spot: C*H*-OH proton of 5-methyl-1-hexyn-3-ol.

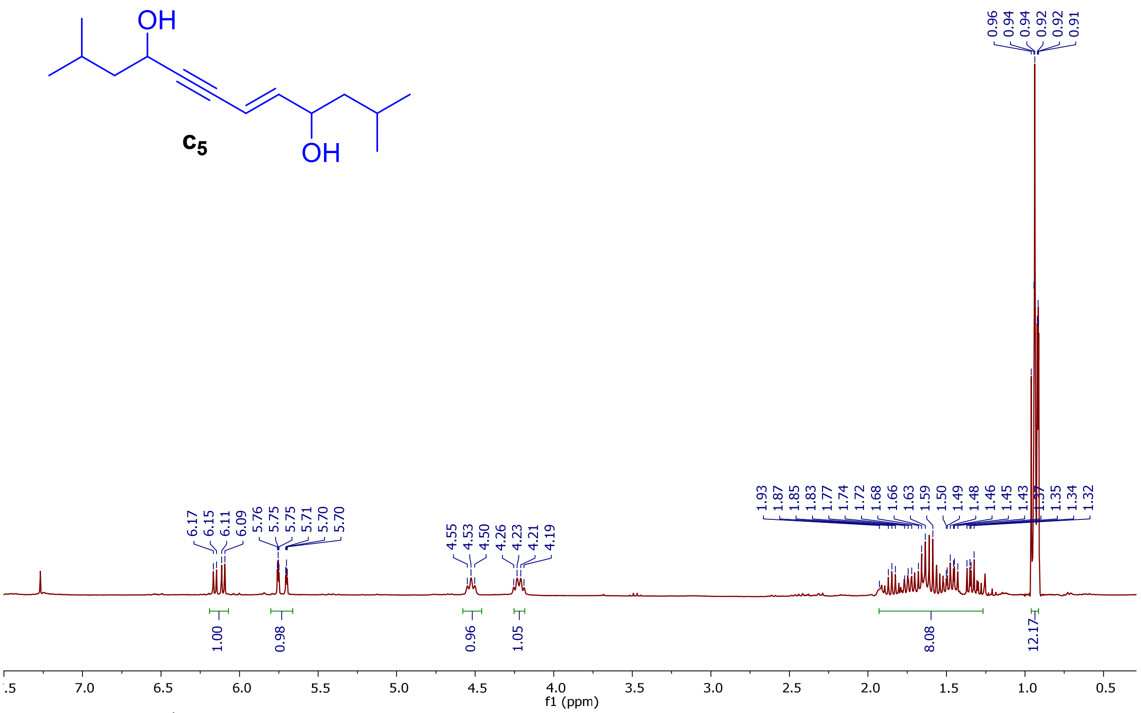

Figure S50. ^{1}H NMR spectrum (300.13 MHz, chloroform-*d*, 298 K) of **c$_5$**.

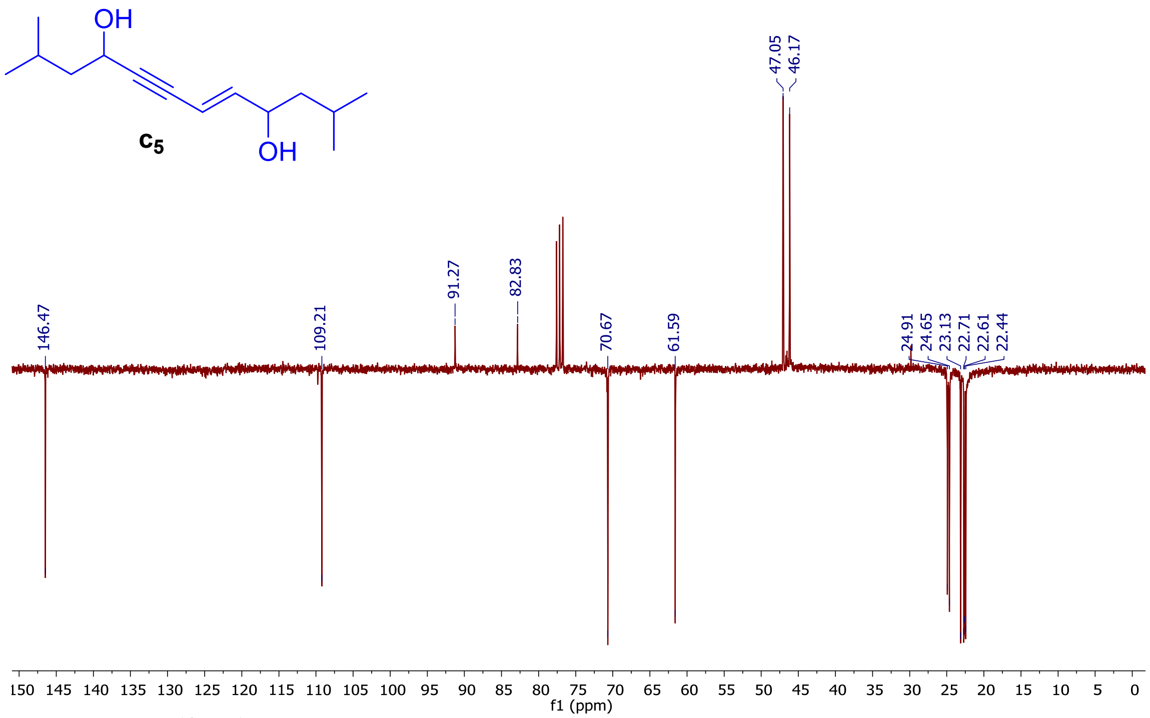

Figure S51. $^{13}C\{^{1}H\}$-apt NMR spectrum (75.48 MHz, chloroform-*d*, 298 K) of $\mathbf{c_5}$.

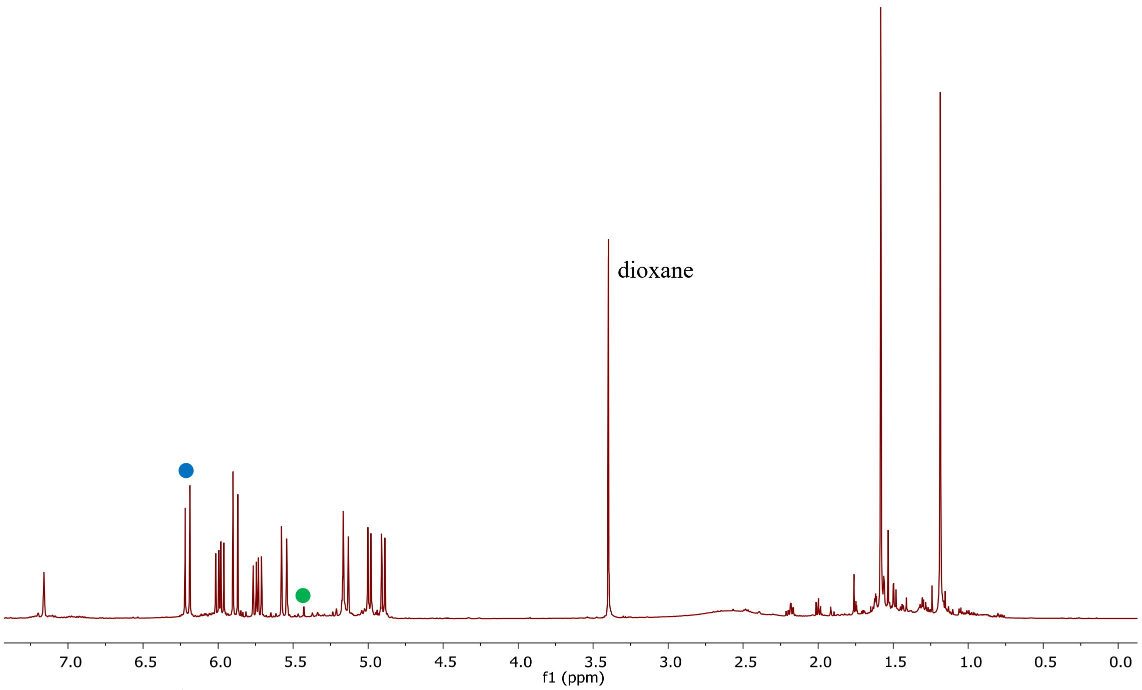

Figure S52. ^{1}H NMR spectrum (500 MHz, benzene-d_6, 298 K) of the crude reaction mixture of the dimerization of 3-methyl-1-penten-4-yn-3-ol to $\mathbf{d_5}$. Characteristic resonances used for the calculation of the reaction conversion are marked as follows: blue spot, one olefinic proton of $\mathbf{d_5}$; green spot, one olefinic proton of the head-to-tail dimer.

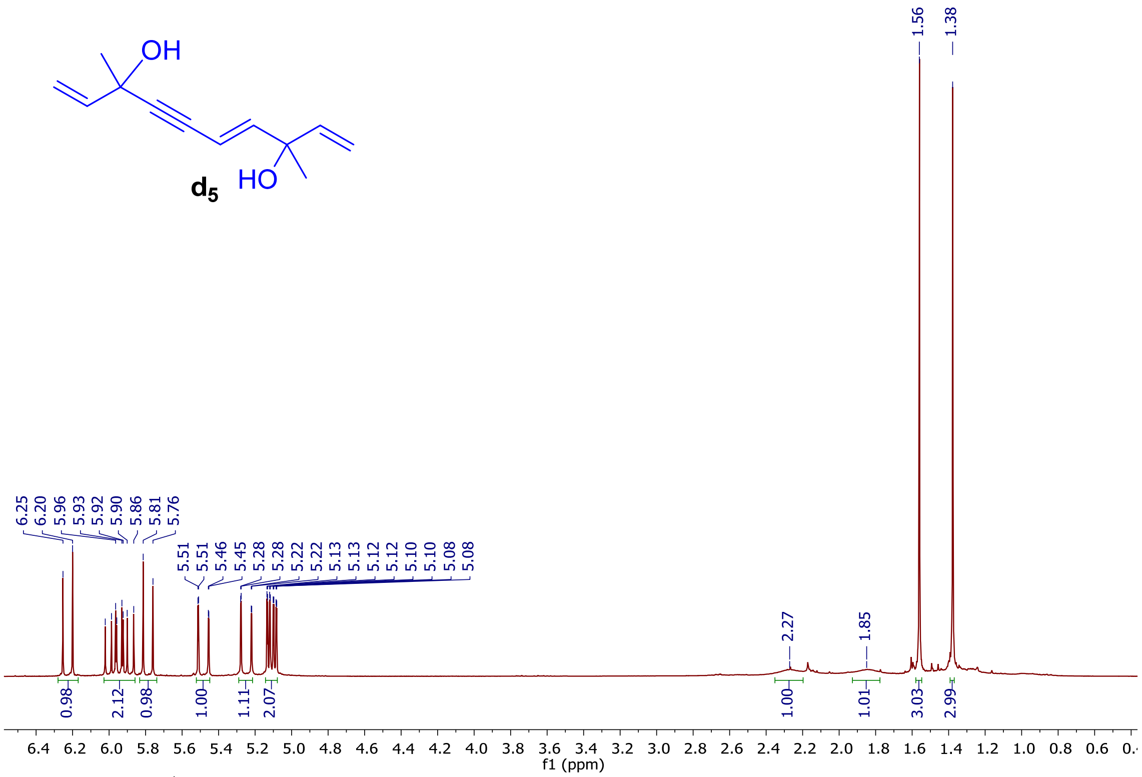

Figure S53. ^{1}H NMR spectrum (300.13 MHz, chloroform-*d*, 298 K) of $\mathbf{d_5}$.

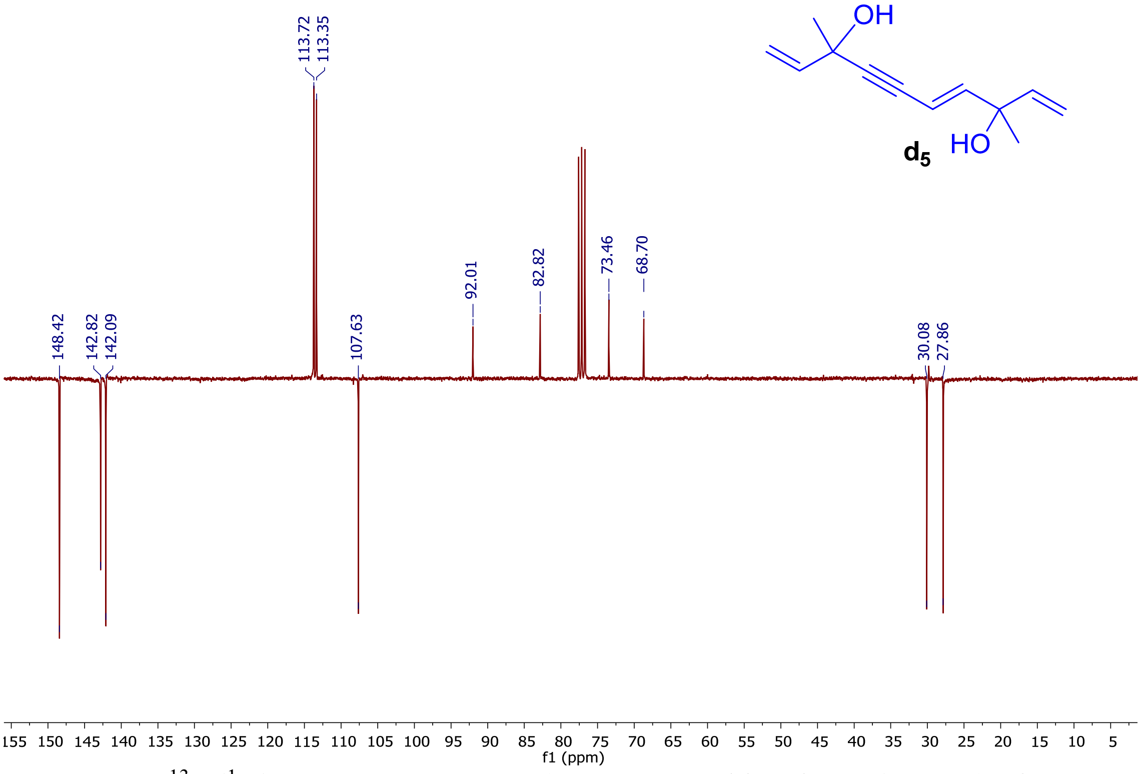

Figure S54. $^{13}C\{^{1}H\}$-apt NMR spectrum (75.48 MHz, chloroform-*d*, 298 K) of $\mathbf{d_5}$.

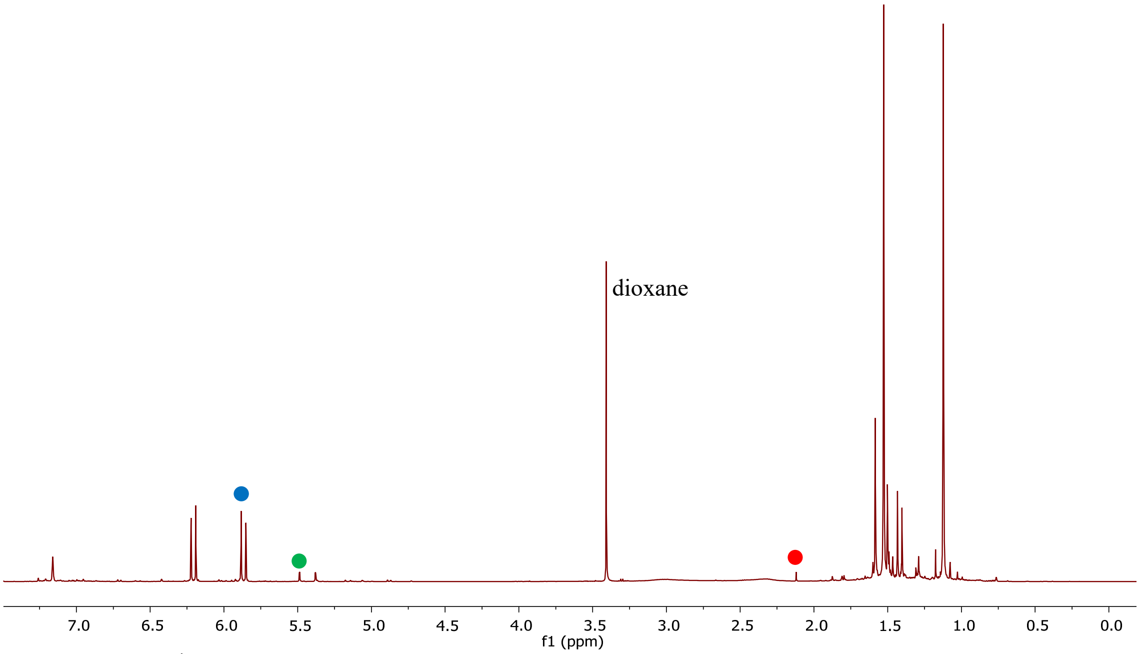

Figure S55. ^1H NMR spectrum (500 MHz, benzene-d_6, 298 K) of the crude reaction mixture of the dimerization of 2-methyl-3-butyn-2-ol to $\mathbf{e_5}$. Characteristic resonances used for the calculation of the reaction conversion are marked as follows: blue spot, one olefinic proton of $\mathbf{e_5}$; green spot, one olefinic proton of the head-to-tail dimer; red spot: HC≡ proton of 2-methyl-3-butyn-2-ol.

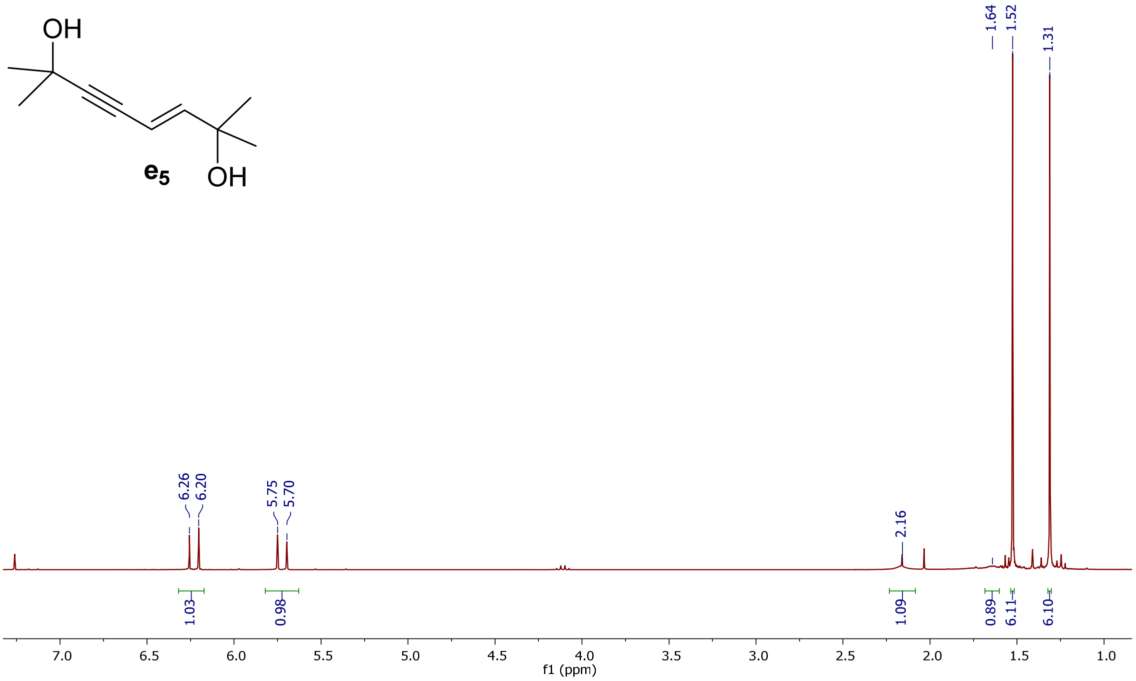

Figure S56. ^1H NMR spectrum (300.13 MHz, chloroform-d, 298 K) of $\mathbf{e_5}$.

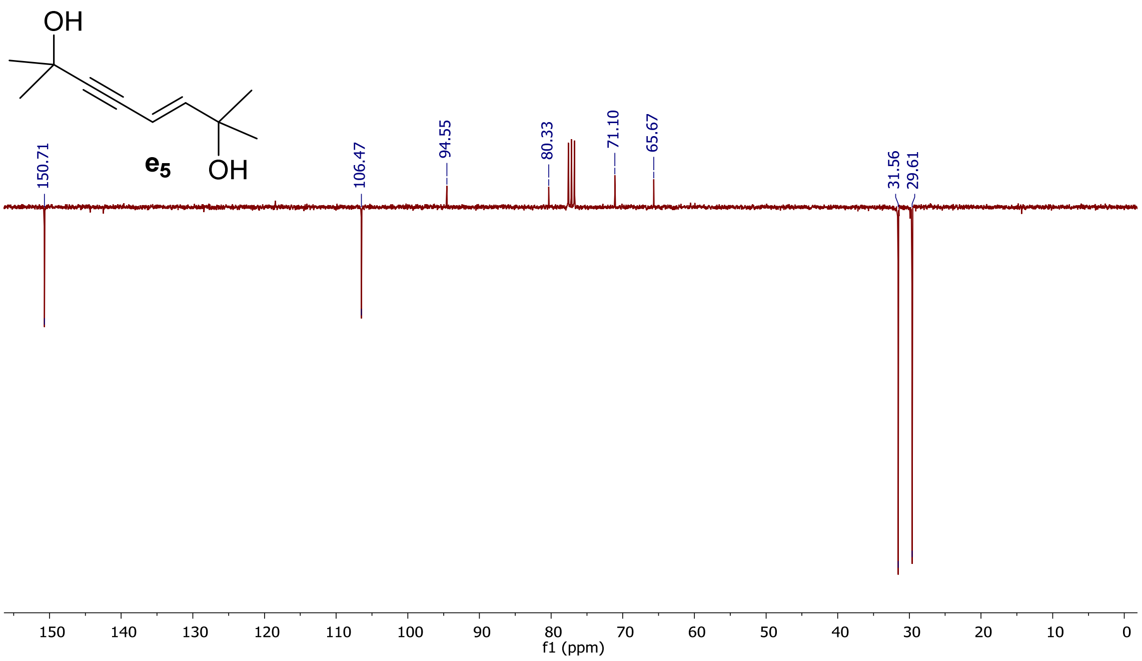

Figure S57. $^{13}C\{^{1}H\}$-apt NMR spectrum (75.48 MHz, chloroform-*d*, 298 K) of **e**$_5$.

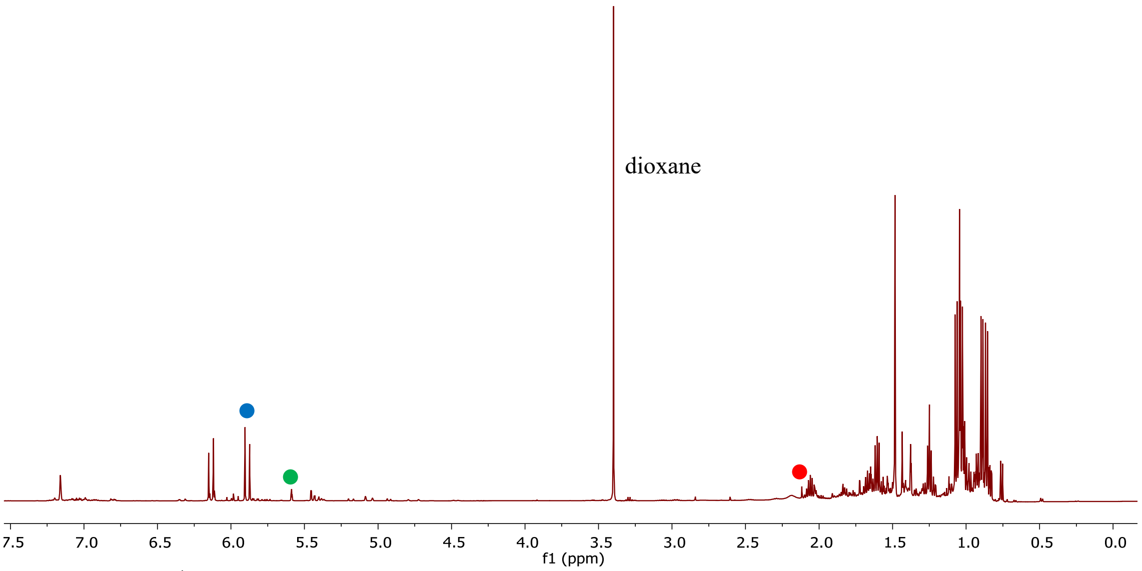

Figure S58. ^{1}H NMR spectrum (500 MHz, benzene-*d*$_6$, 298 K) of the crude reaction mixture of the dimerization of 3,5-dimethyl-1-hexyn-3-ol to **f**$_5$. Characteristic resonances used for the calculation of the reaction conversion are marked as follows: blue spot, one olefinic proton of **f**$_5$; green spot, one olefinic proton of the head-to-tail dimer; red spot: HC≡ proton of 3,5-dimethyl-1-hexyn-3-ol.

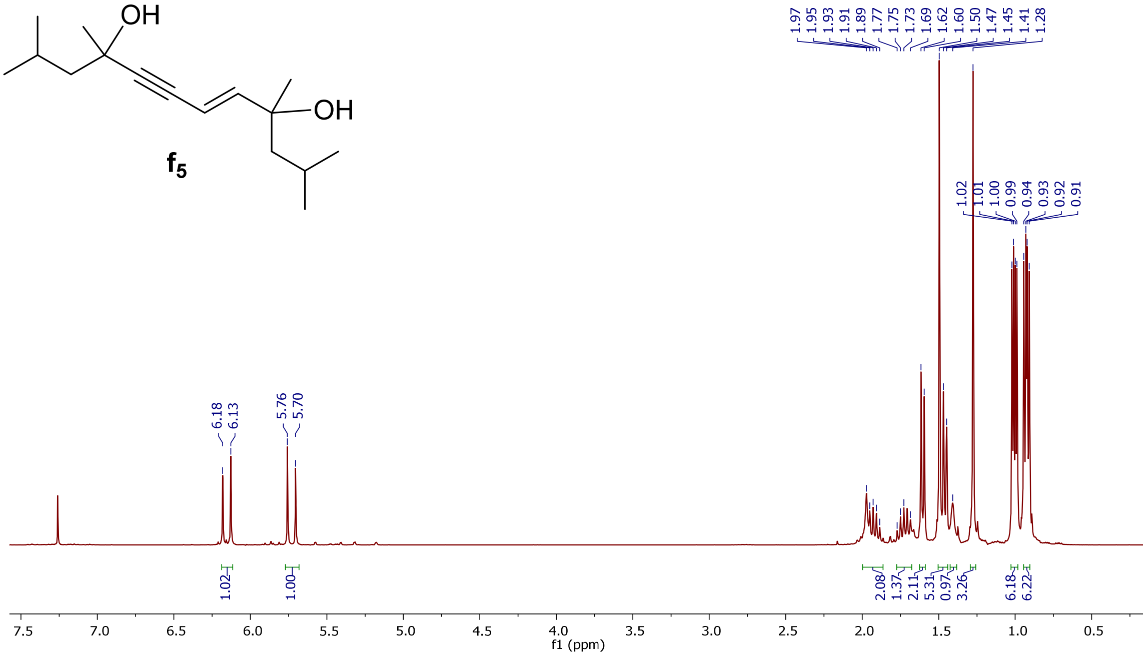

Figure S59. ^{1}H NMR spectrum (300.13 MHz, chloroform-*d*, 298 K) of **f$_5$**.

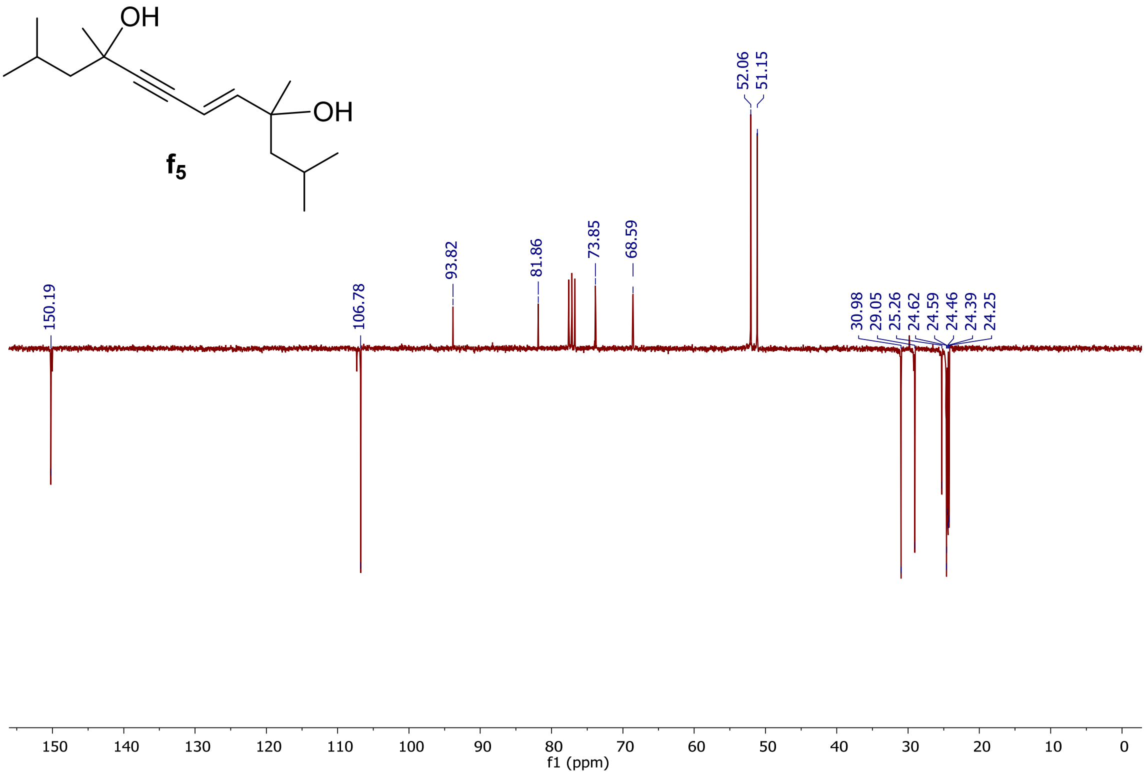

Figure S60. $^{13}C\{^{1}H\}$-apt NMR spectrum (75.48 MHz, chloroform-*d*, 298 K) of **f$_5$**.

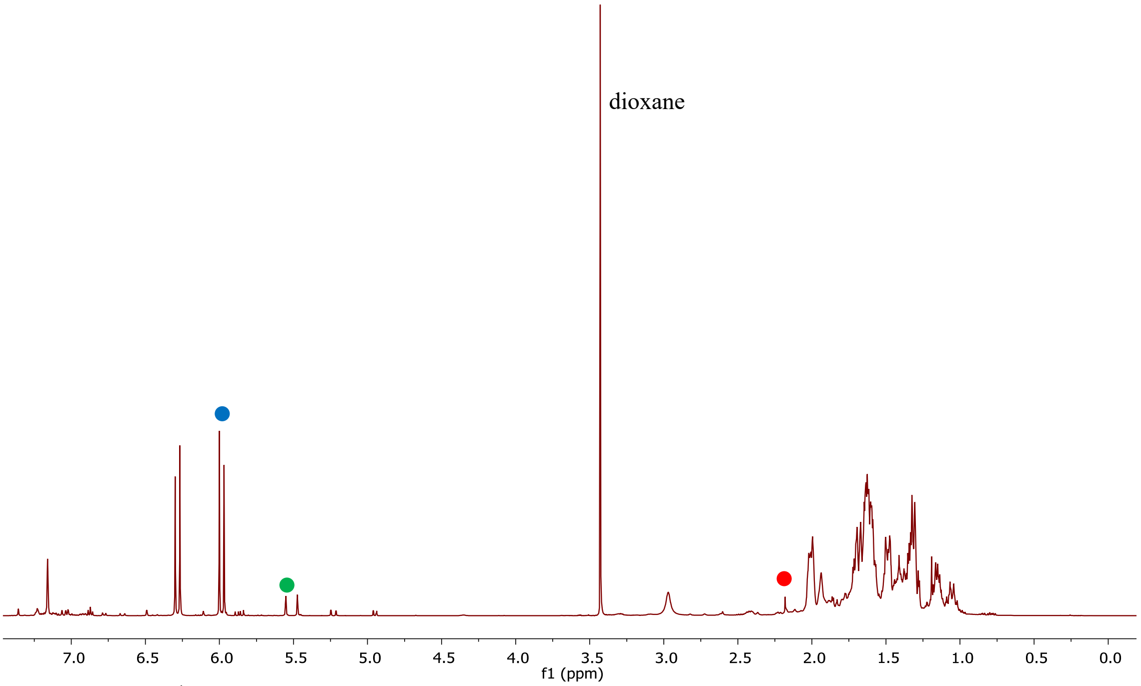

Figure S61. ^{1}H NMR spectrum (500 MHz, benzene-*d*6, 298 K) of the crude reaction mixture of the dimerization of 1-ethynyl-1-cyclohexanol to **g5**. Characteristic resonances used for the calculation of the reaction conversion are marked as follows: blue spot, one olefinic proton of **g5**; green spot, one olefinic proton of the head-to-tail dimer; red spot: HC≡ proton of 1-ethynyl-1-cyclohexanol.

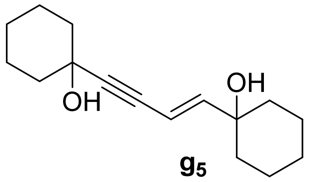

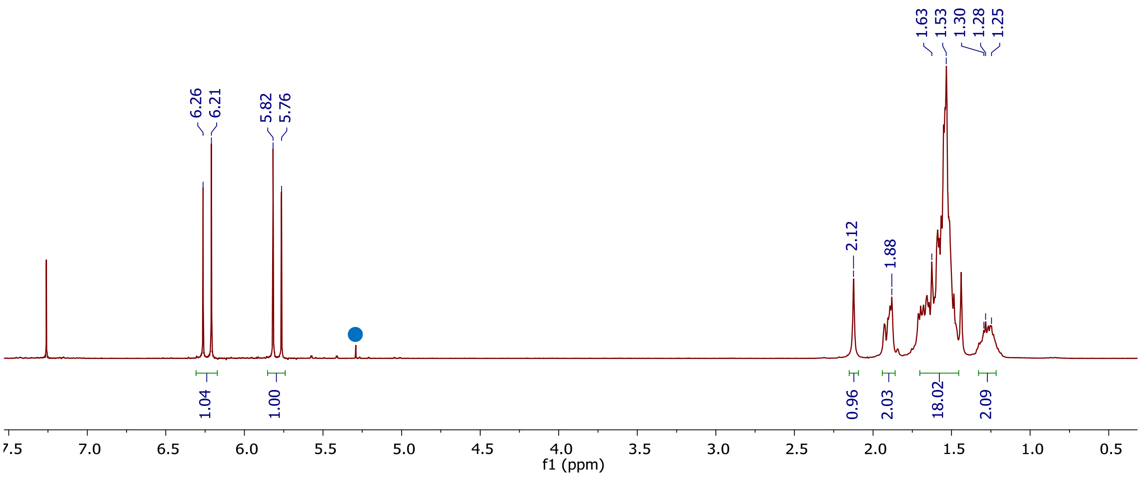

Figure S62. ^{1}H NMR spectrum (300.13 MHz, chloroform-*d*, 298 K) of **g5**. Blue spot: dichloromethane

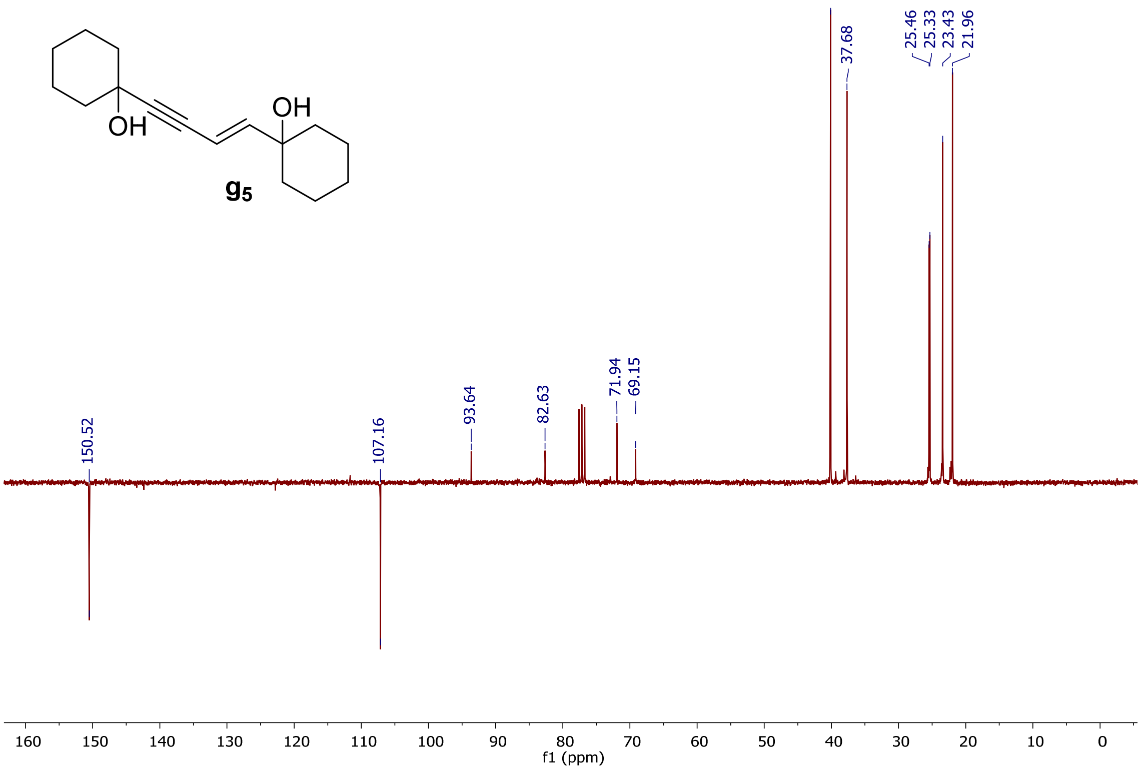

Figure S63. $^{13}C\{^{1}H\}$-apt NMR spectrum (75.48 MHz, chloroform-*d*, 298 K) of $\mathbf{g_5}$.

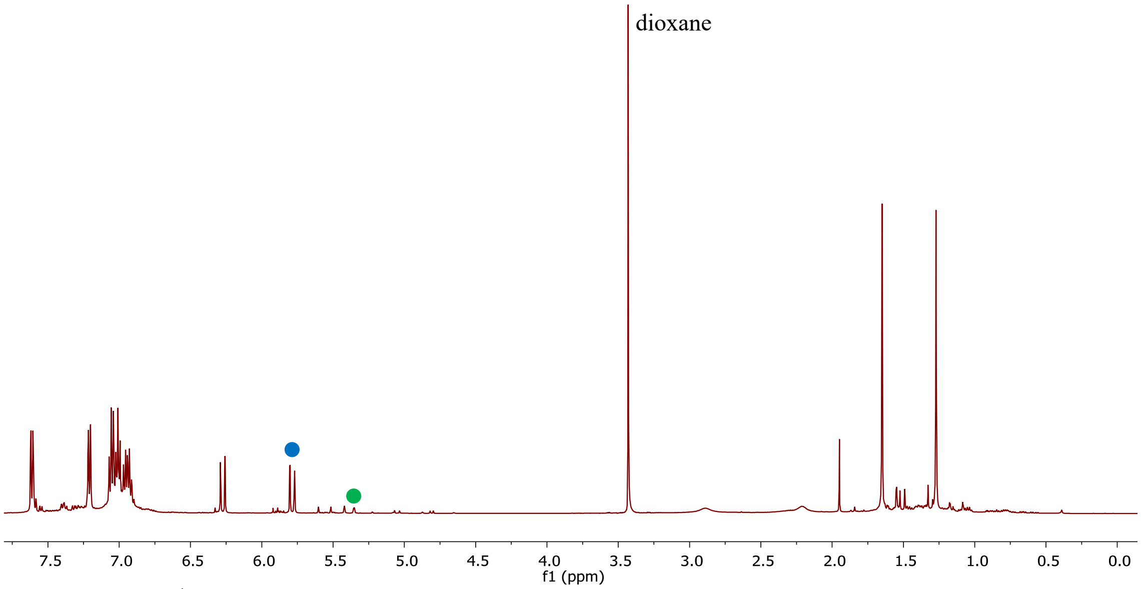

Figure S64. ^{1}H NMR spectrum (500 MHz, benzene-d_6, 298 K) of the crude reaction mixture of the dimerization of 2-phenyl-3-butyn-2-ol to $\mathbf{h_5}$. Characteristic resonances used for the calculation of the reaction conversion are marked as follows: blue spot, one olefinic proton of $\mathbf{h_5}$; green spot, one olefinic proton of the head-to-tail dimer.

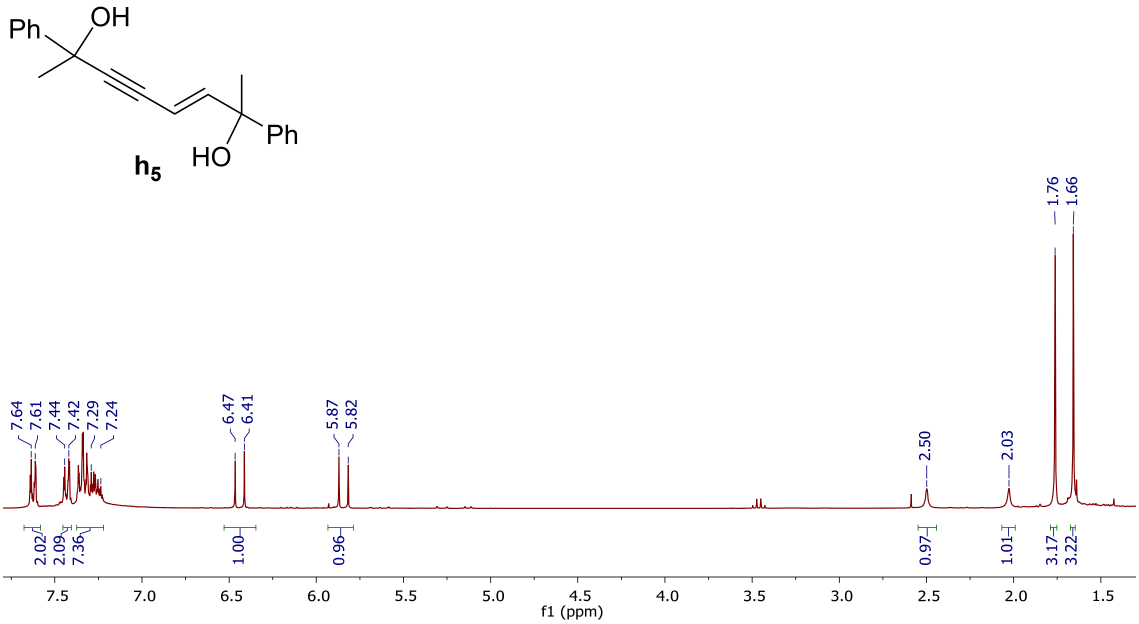

Figure S65. ^{1}H NMR spectrum (300.13 MHz, chloroform-*d*, 298 K) of $\mathbf{h_5}$.

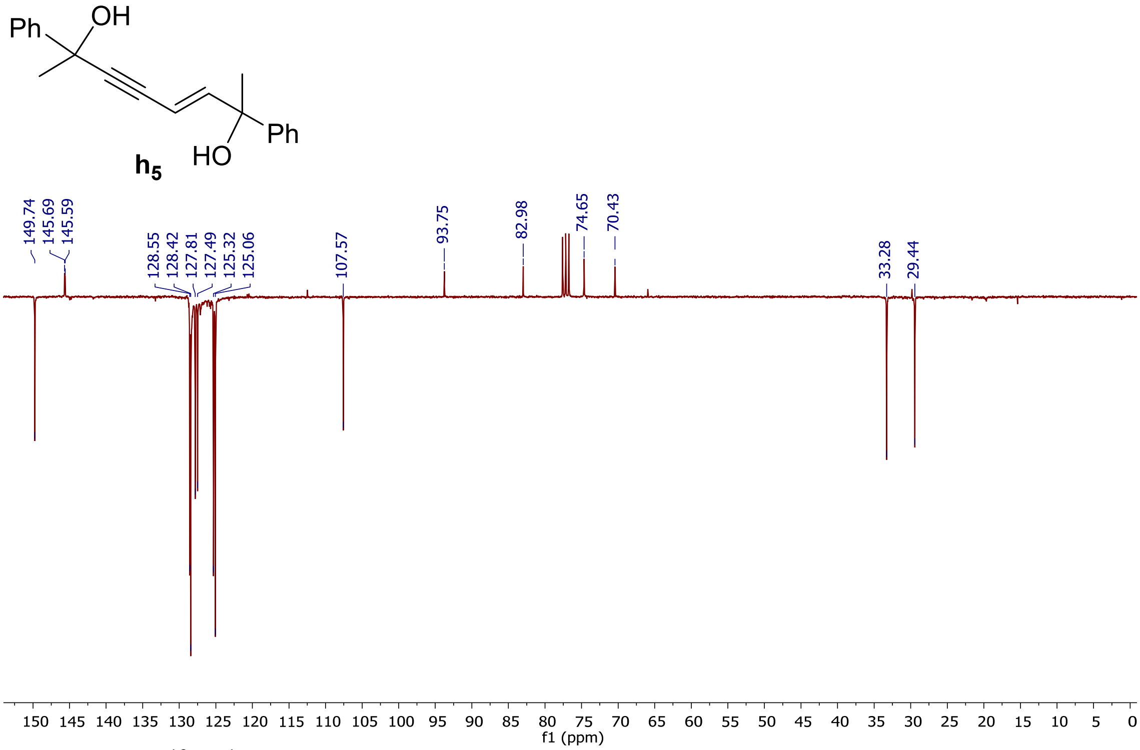

Figure S66. $^{13}C\{^{1}H\}$-apt NMR spectrum (75.48 MHz, chloroform-*d*, 298 K) of $\mathbf{h_5}$.

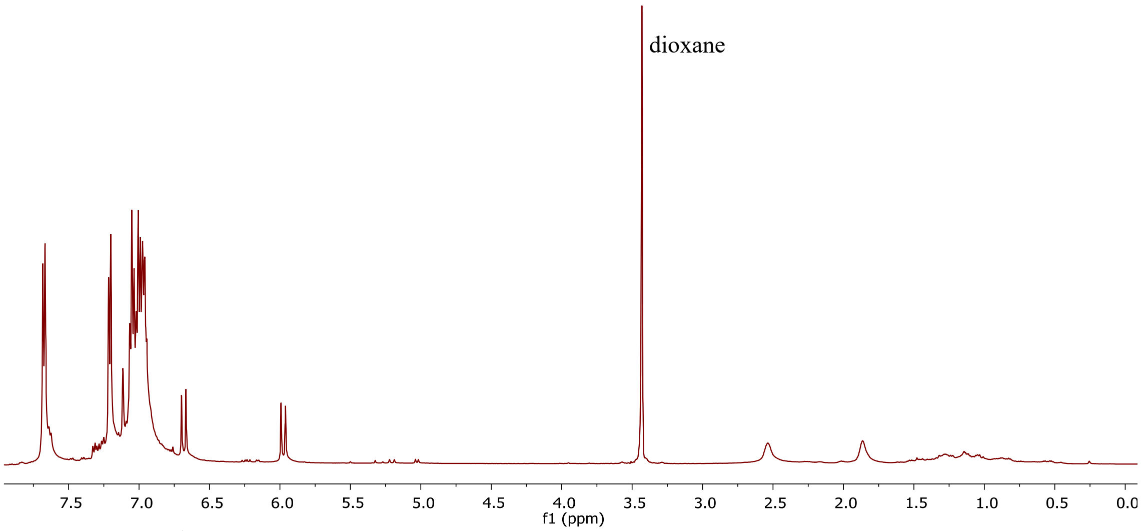

Figure S67. ^{1}H NMR spectrum (500 MHz, benzene-*d*6, 298 K) of the crude reaction mixture of the dimerization of 1,1-diphenyl-2-propyn-1-ol to **i5**.

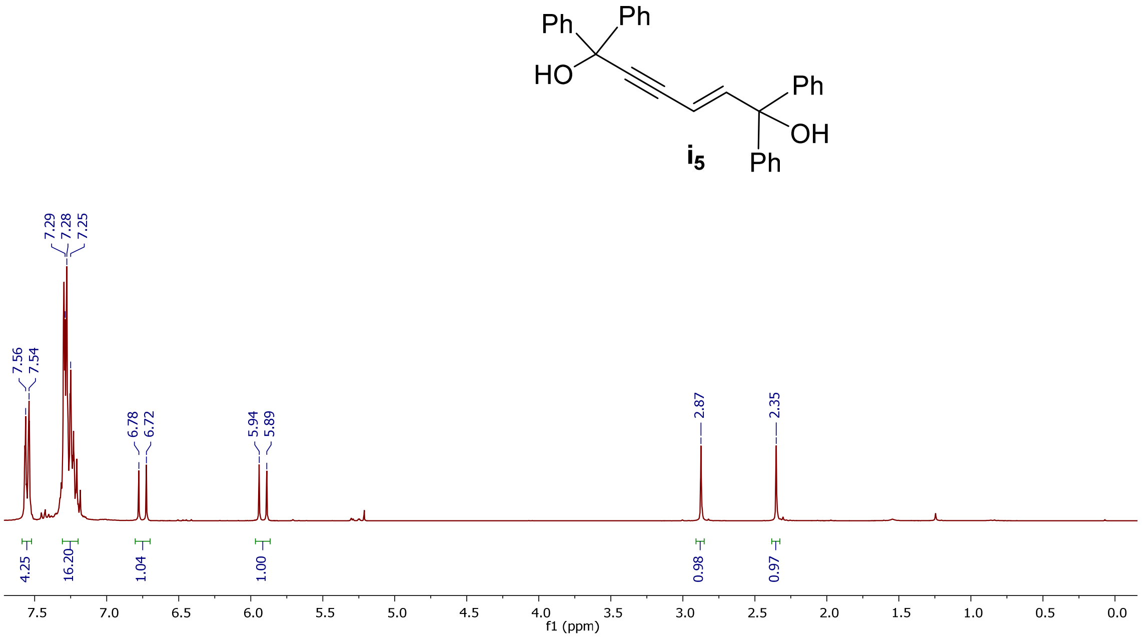

Figure S68. ^{1}H NMR spectrum (300.13 MHz, chloroform-*d*, 298 K) of **i5**.

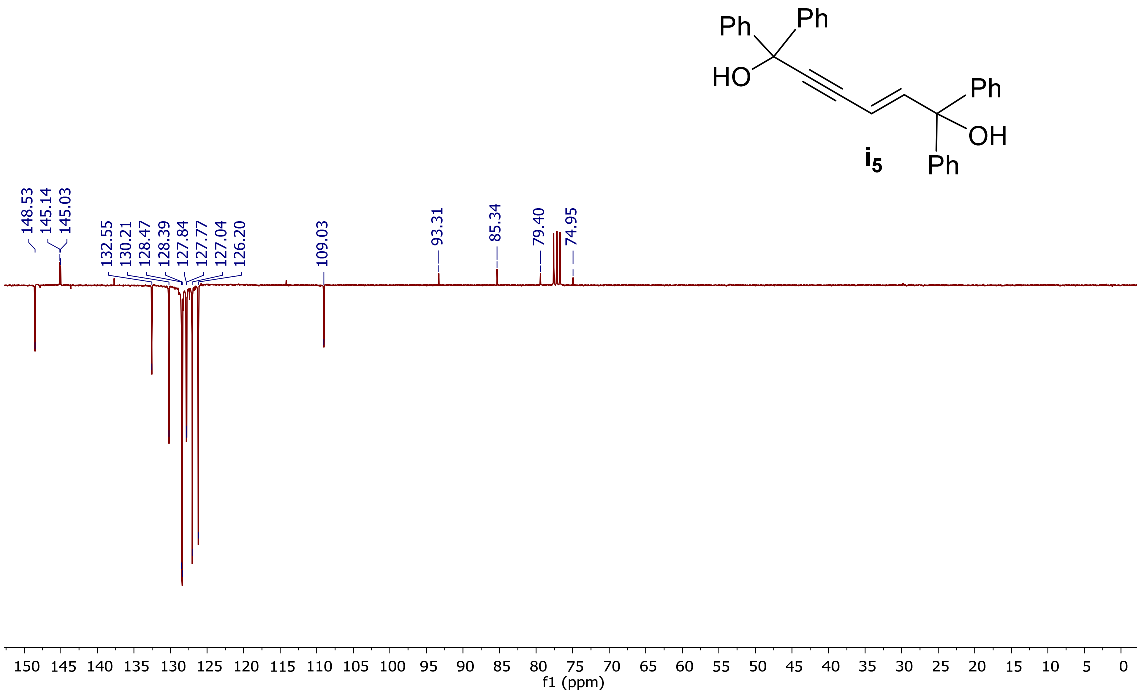

Figure S69. $^{13}C\{^{1}H\}$-apt NMR spectrum (75.48 MHz, chloroform-*d*, 298 K) of $\mathbf{i_5}$.

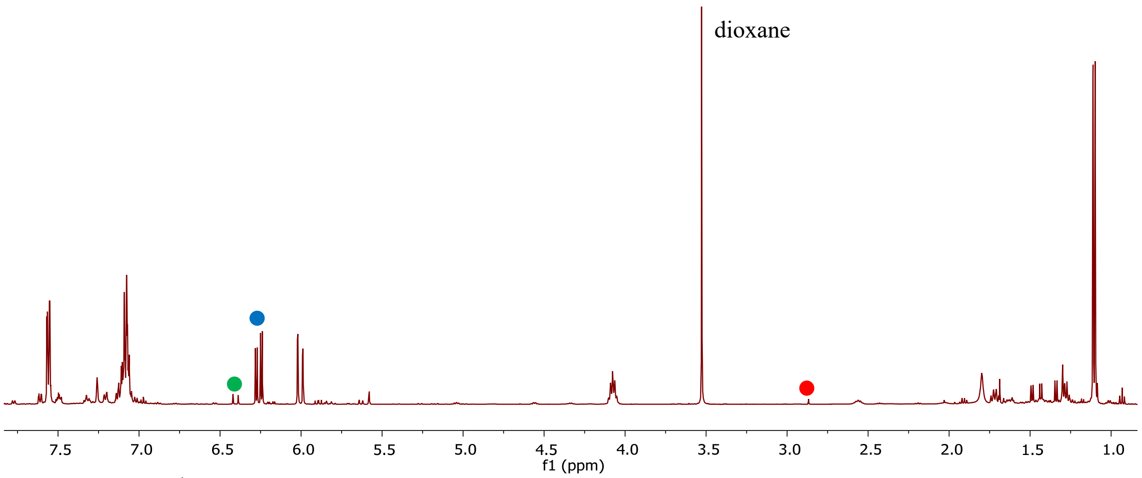

Figure S70. ^{1}H NMR spectrum (500 MHz, benzene-d_6, 298 K) of the crude reaction mixture of the cross-coupling of phenylacetylene and 3-butyn-2-ol to give $\mathbf{a_6}$. Characteristic resonances used for the calculation of the reaction conversion are marked as follows: blue spot, one olefinic proton of $\mathbf{a_6}$; green spot, one olefinic proton of $\mathbf{a_4}$; red spot: HC≡ proton of phenylacetylene.

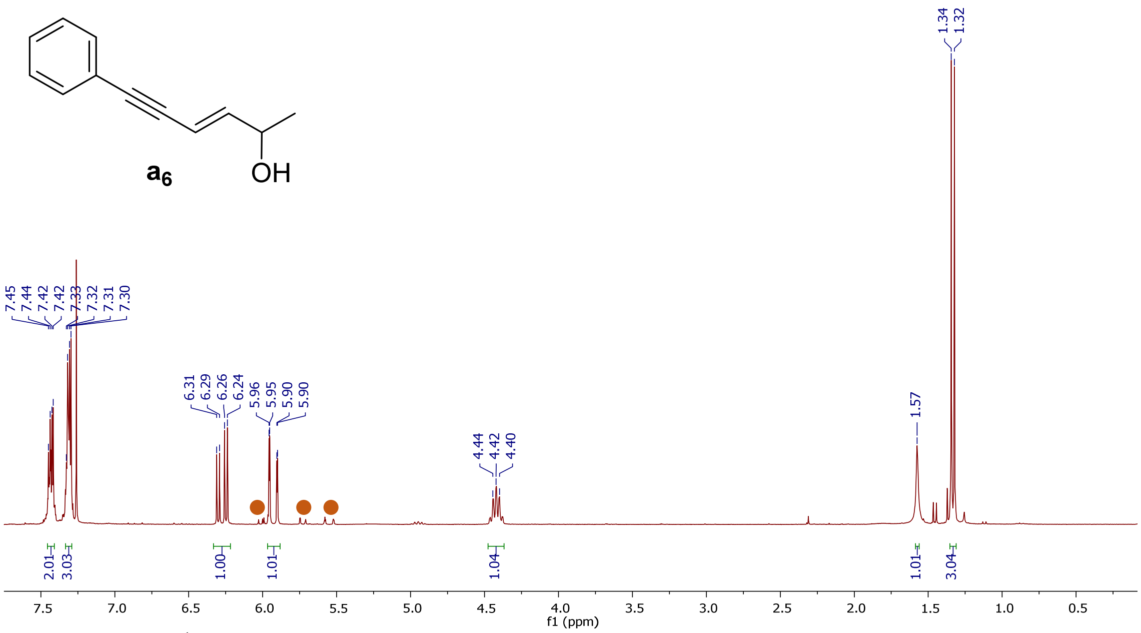

Figure S71. ^{1}H NMR spectrum (300.13 MHz, chloroform-*d*, 298 K) of $\mathbf{a_6}$. Orange spots: olefinic protons of but-3-en-2-ol.

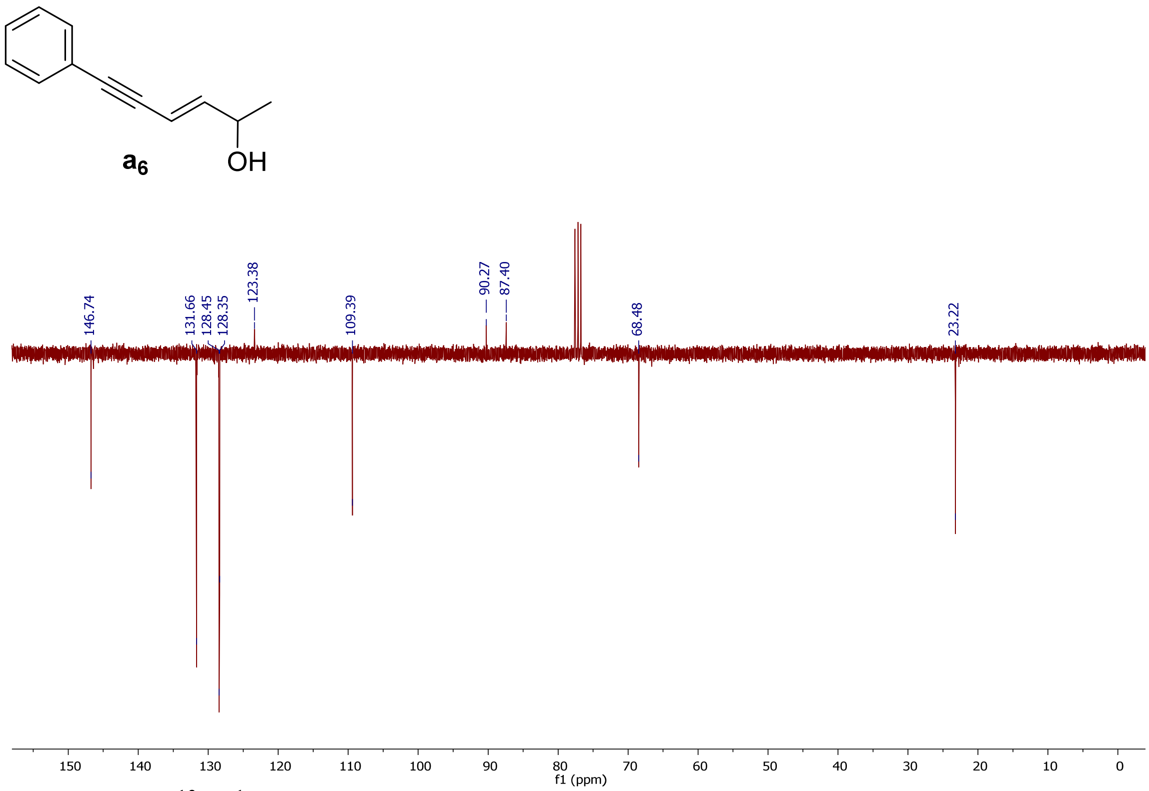

Figure S72. $^{13}C\{^{1}H\}$-apt NMR spectrum (75.48 MHz, chloroform-*d*, 298 K) of $\mathbf{a_6}$.

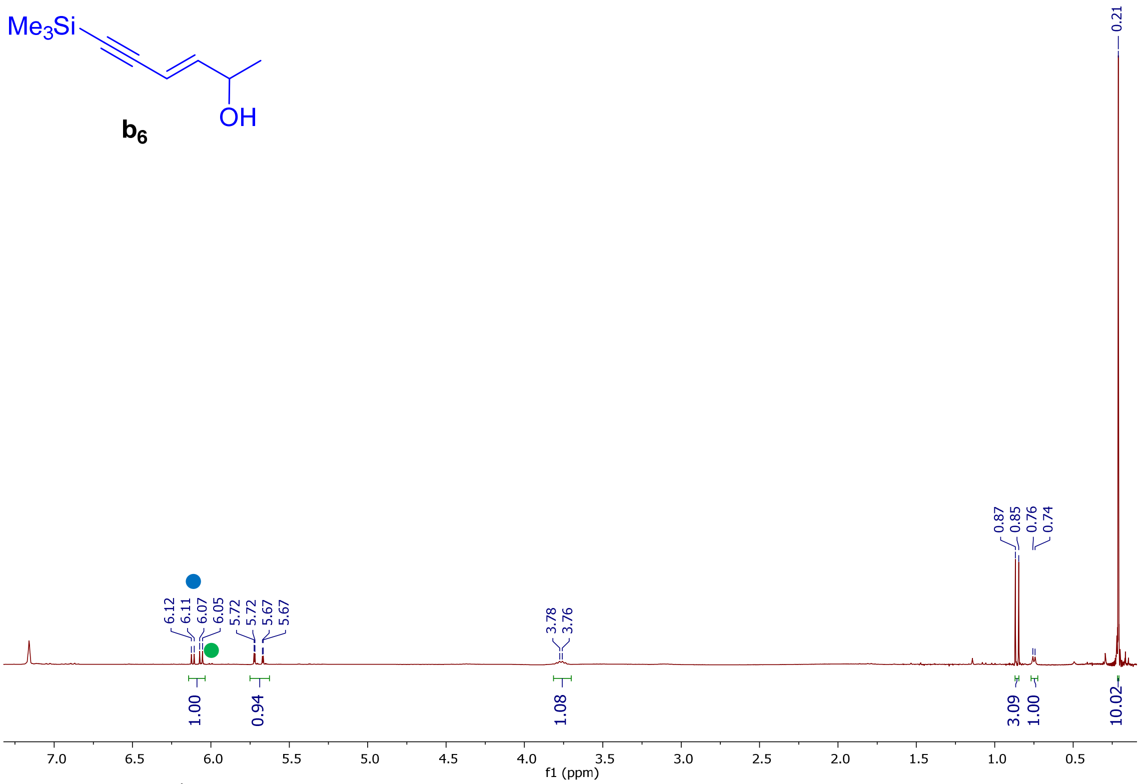

Figure S73. ^{1}H NMR spectrum (300 MHz, benzene-d_6, 298 K) of the crude reaction mixture of the cross-coupling of trimethylsilylacetylene and 3-butyn-2-ol to give $\mathbf{b_6}$. Characteristic resonances used for the calculation of the reaction conversion are marked as follows: blue spot, one olefinic proton of $\mathbf{b_6}$; green spot: one olefinic proton of $\mathbf{a_5}$.

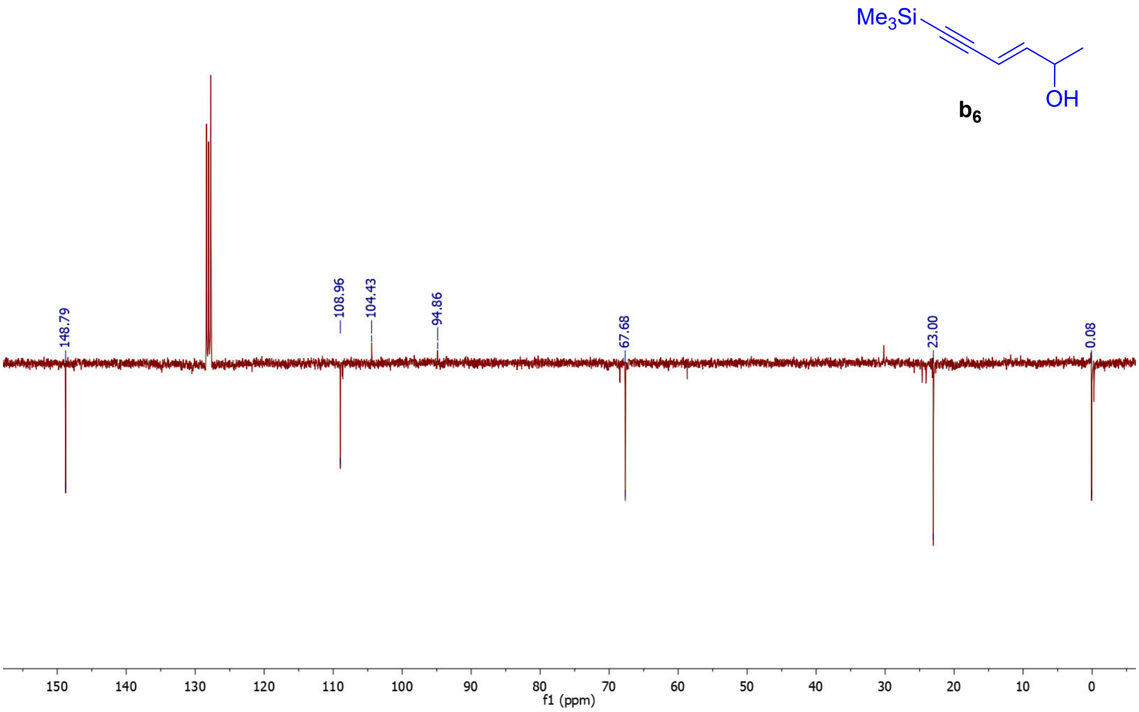

Figure S74. $^{13}C\{^{1}H\}$-apt NMR spectrum (75.48 MHz, benzene-d_6, 298 K) of crude reaction mixture of the cross-coupling of $\mathbf{b_6}$.

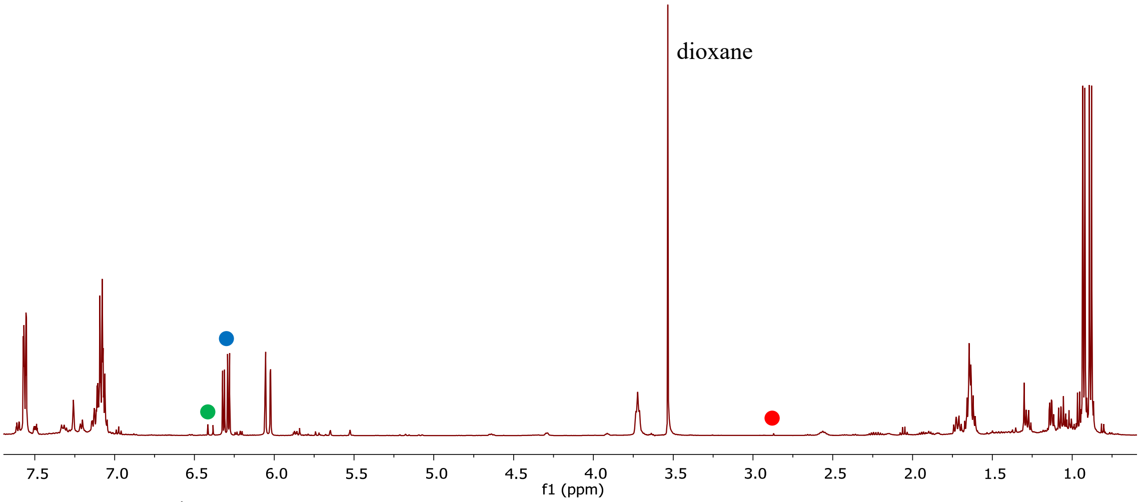

Figure S75. ^{1}H NMR spectrum (500 MHz, benzene-d_6, 298 K) of the crude reaction mixture of the cross-coupling of phenylacetylene and 4-methyl-1-pentyn-3-ol to give **c_6**. Characteristic resonances used for the calculation of the reaction conversion are marked as follows: blue spot, one olefinic proton of **c_6**; green spot, one olefinic proton of **a_4**; red spot: HC≡ proton of phenylacetylene.

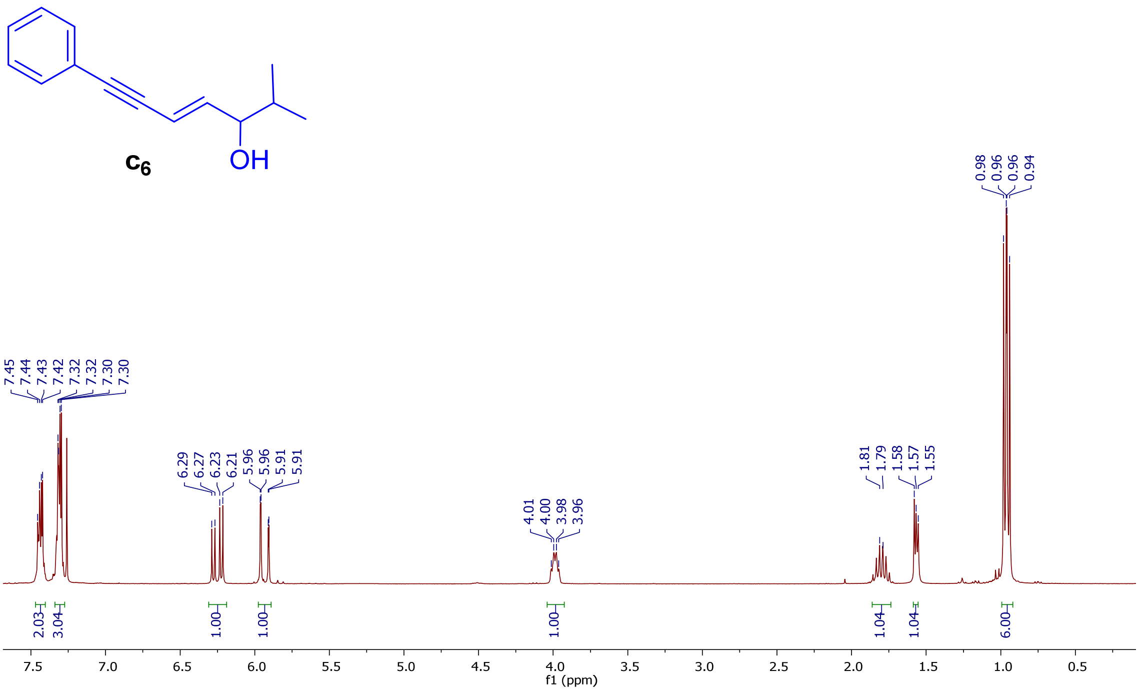

Figure S76. ^{1}H NMR spectrum (300.13 MHz, chloroform-d, 298 K) of **c_6**.

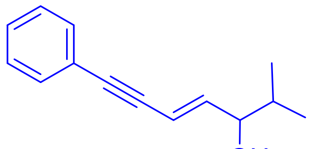

c$_6$

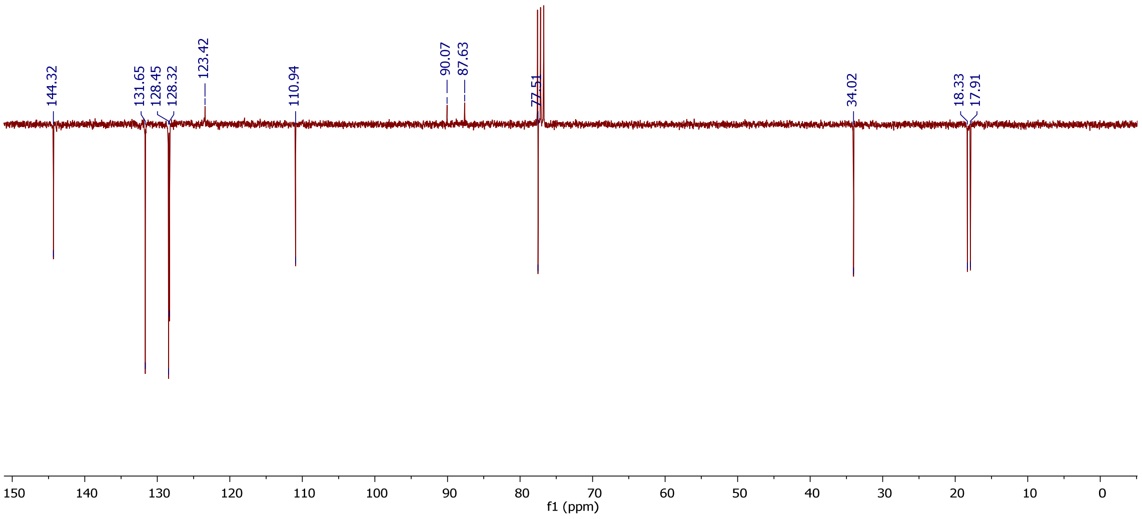

Figure S77. $^{13}C\{^{1}H\}$-apt NMR spectrum (75.48 MHz, chloroform-*d*, 298 K) of **c$_6$**.

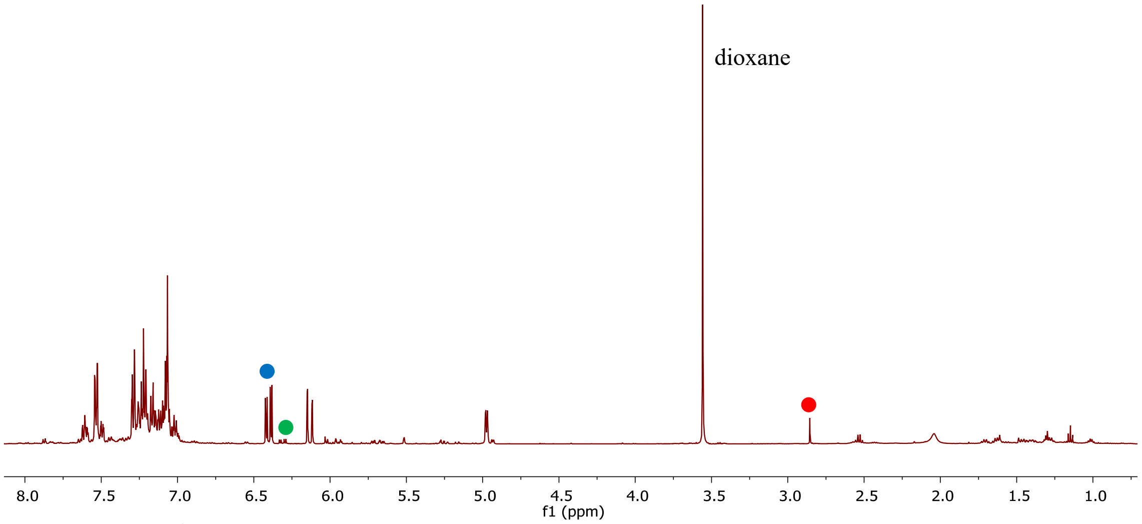

Figure S78. ^{1}H NMR spectrum (500 MHz, benzene-*d*$_6$, 298 K) of the crude reaction mixture of the cross-coupling of phenylacetylene and 1-phenyl-2-propyn-1-ol to give **d$_6$**. Characteristic resonances used for the calculation of the reaction conversion are marked as follows: blue spot, one olefinic proton of **d$_6$**; green spot, one olefinic proton of homo-coupling of 1-phenyl-2-propyn-1-ol; red spot: HC≡ proton of phenylacetylene.

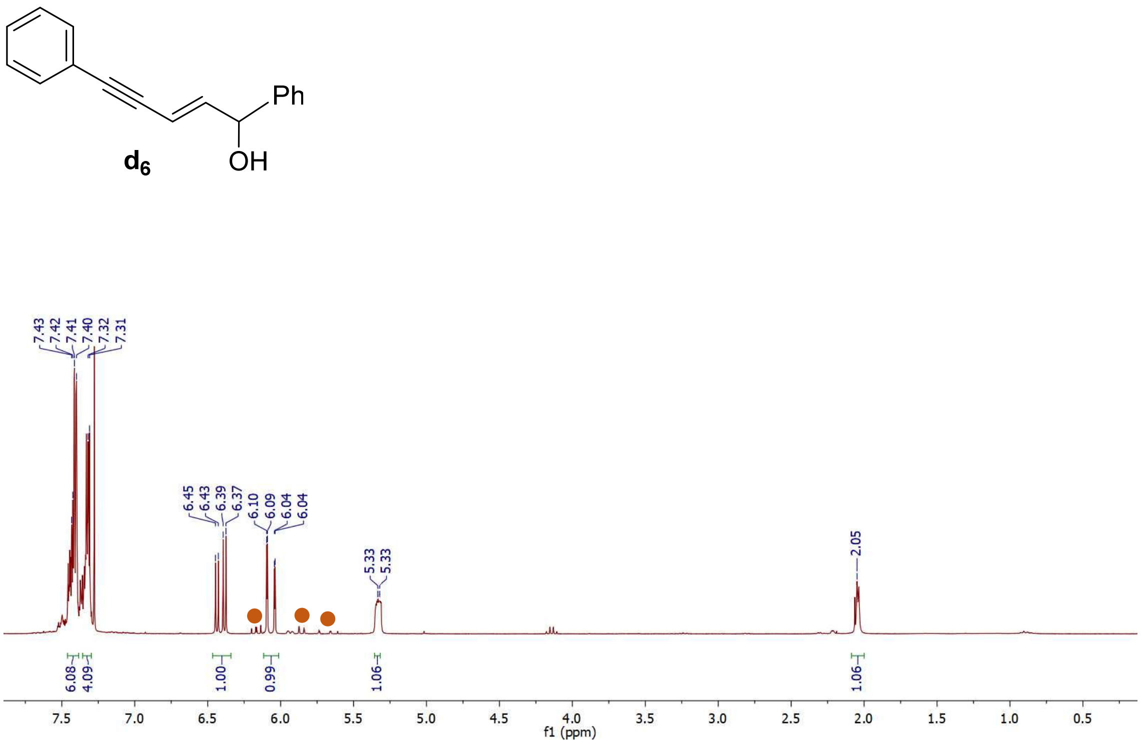

Figure S79. ^{1}H NMR spectrum (300.13 MHz, chloroform-*d*, 298 K) of $\mathbf{d_6}$. Orange spots: olefinic protons of 1-phenyl-2-propen-1-ol.

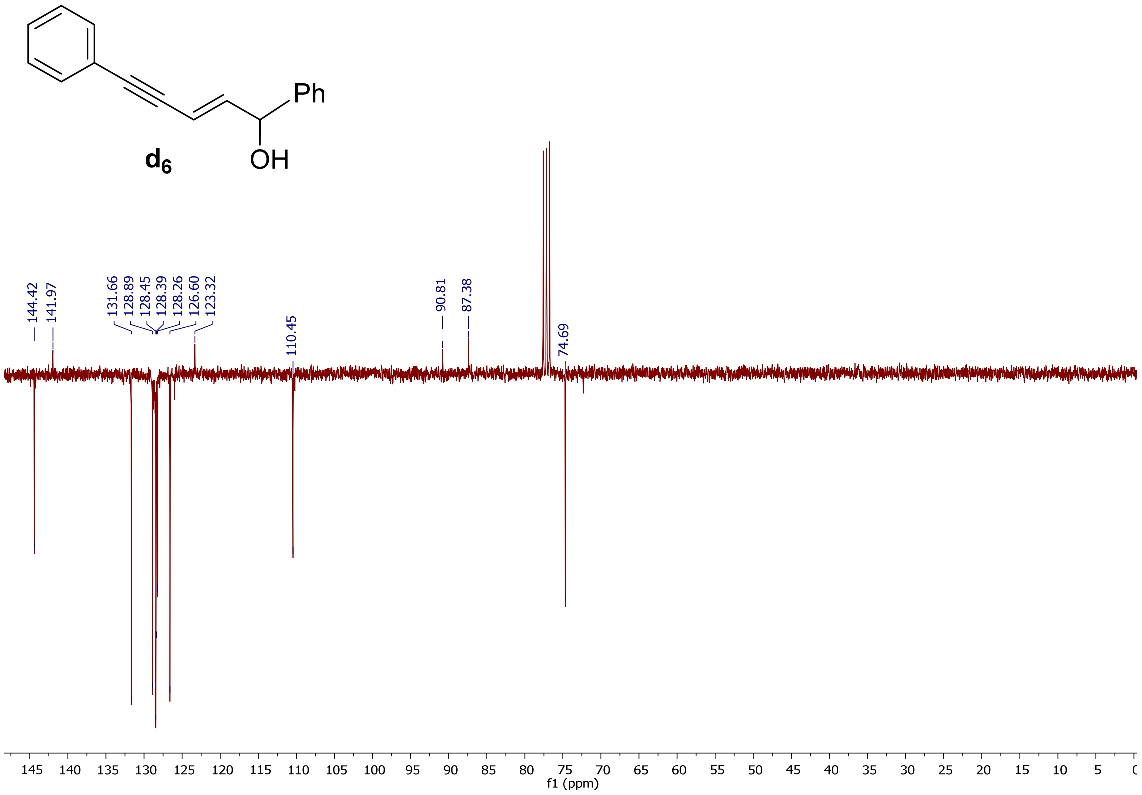

Figure S80. $^{13}C\{^{1}H\}$-apt NMR spectrum (75.48 MHz, chloroform-*d*, 298 K) of $\mathbf{d_6}$.

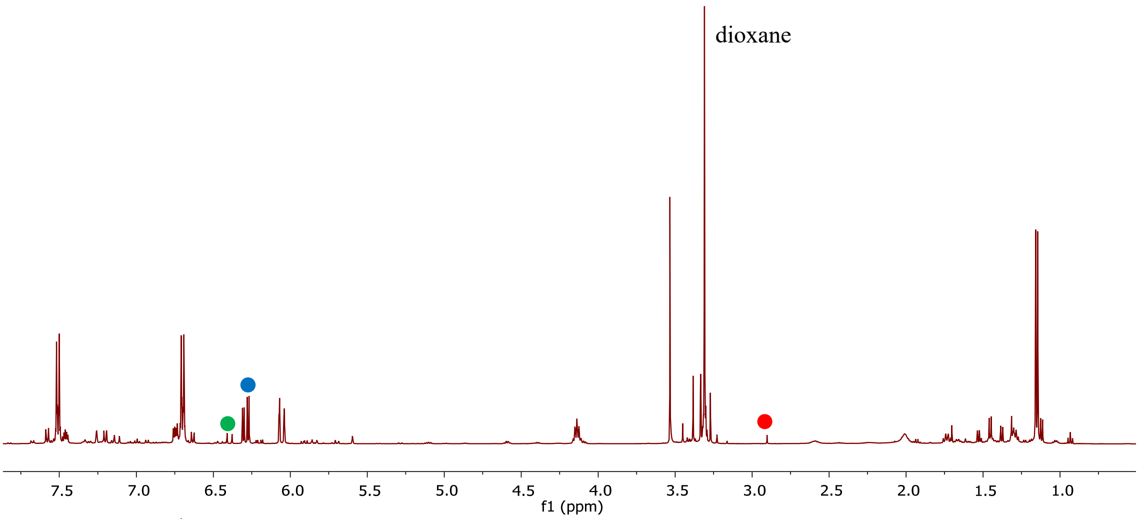

Figure S81. ^{1}H NMR spectrum (500 MHz, benzene-d_6, 298 K) of the crude reaction mixture of the cross-coupling of 4-methoxyphenylacetylene and 3-butyn-2-ol to give $\mathbf{e_6}$. Characteristic resonances used for the calculation of the reaction conversion are marked as follows: blue spot, one olefinic proton of $\mathbf{e_6}$; green spot, one olefinic proton of $\mathbf{b_4}$; red spot: HC≡ proton of 4-methoxyphenylacetylene.

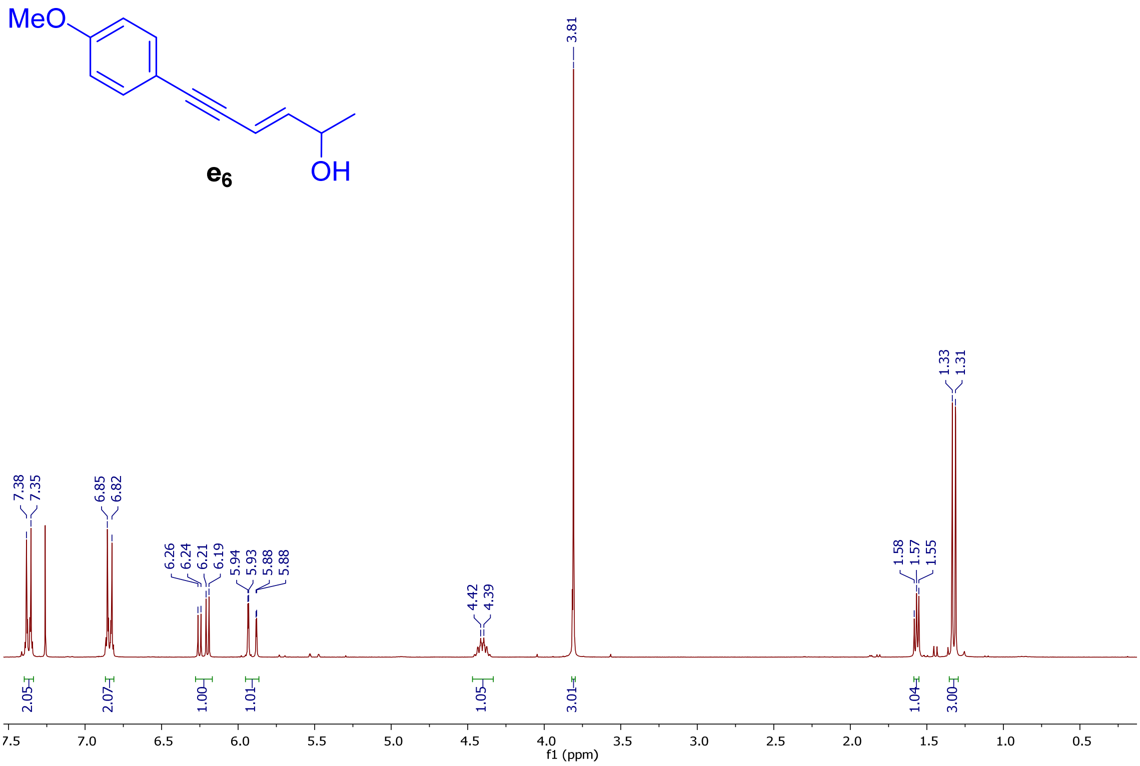

Figure S82. ^{1}H NMR spectrum (300.13 MHz, chloroform-d, 298 K) of $\mathbf{e_6}$.

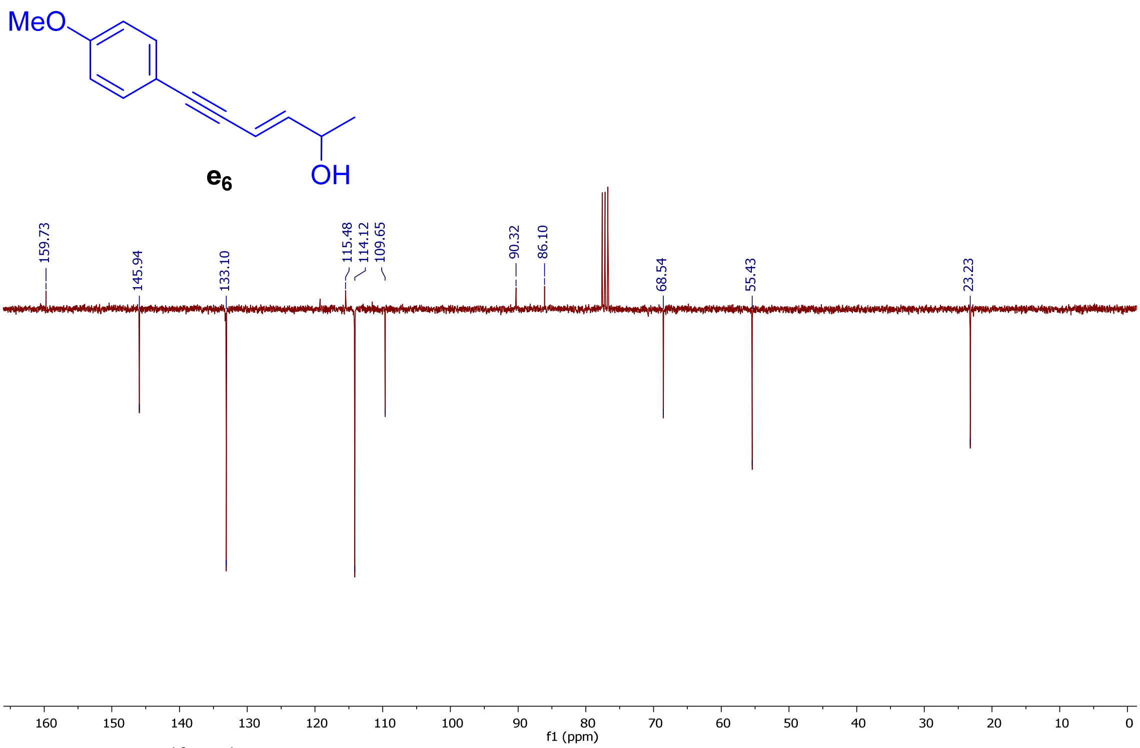

Figure S83. $^{13}C\{^{1}H\}$-apt NMR spectrum (75.48 MHz, chloroform-*d*, 298 K) of $\mathbf{e_6}$.

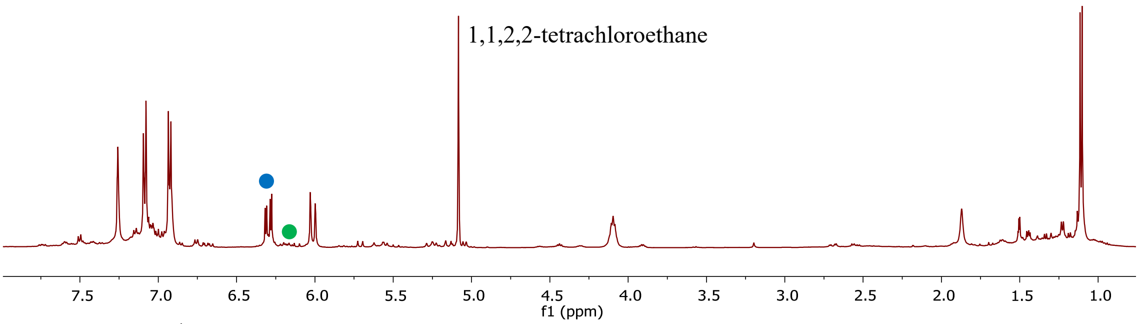

Figure S84. ^{1}H NMR spectrum (500 MHz, benzene-d_6, 298 K) of the crude reaction mixture of the cross-coupling of 4-cyanophenylacetylene and 3-butyn-2-ol to give $\mathbf{f_6}$. Characteristic resonances used for the calculation of the reaction conversion are marked as follows: blue spot, one olefinic proton of $\mathbf{f_6}$; green spot, one olefinic proton of $\mathbf{a_5}$.

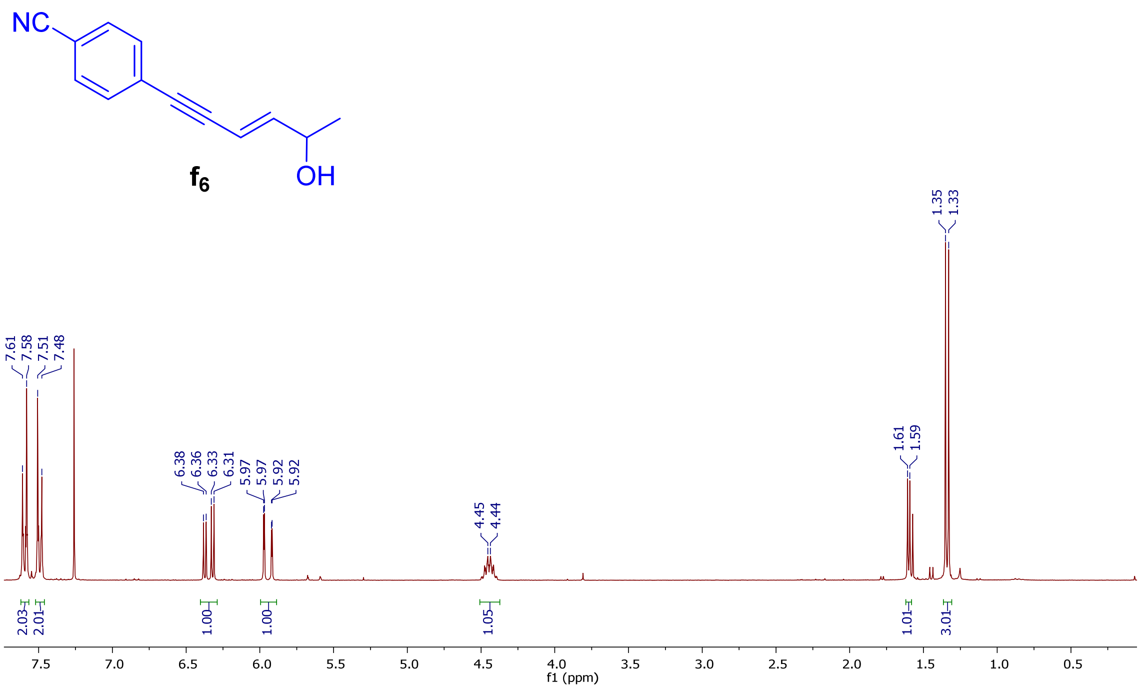

Figure S85. ^{1}H NMR spectrum (300.13 MHz, chloroform-*d*, 298 K) of $\mathbf{f_6}$.

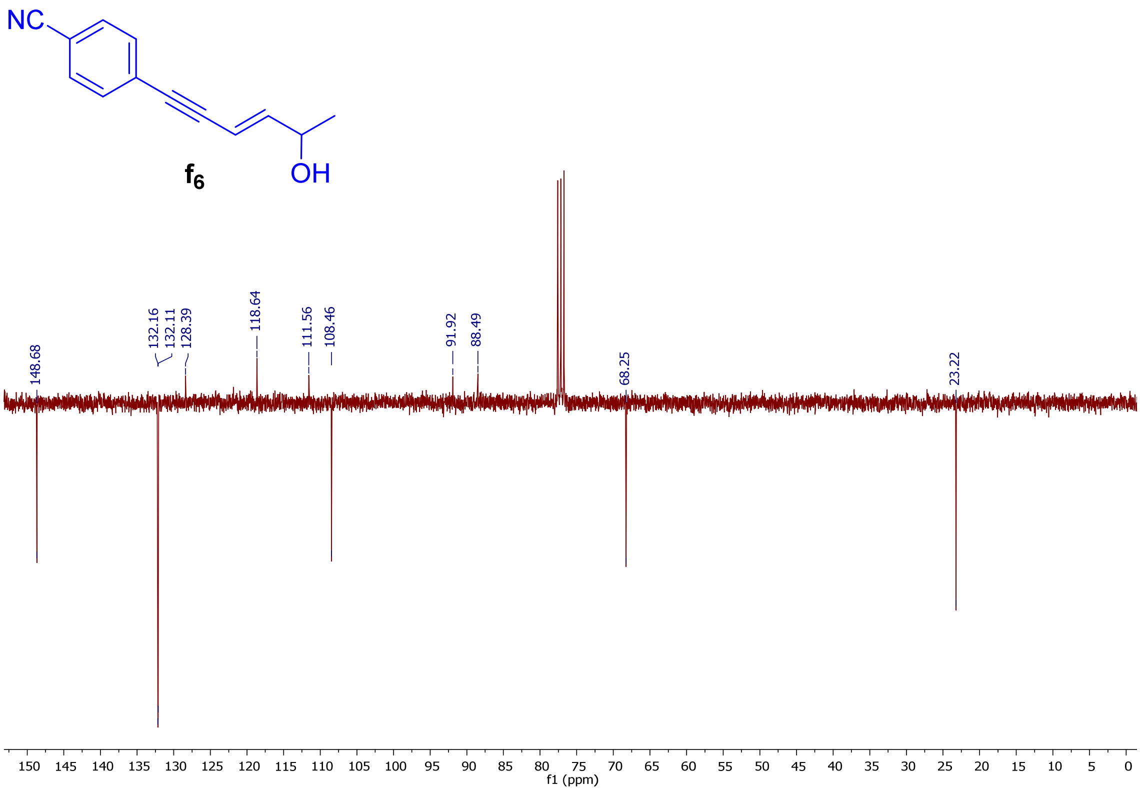

Figure S86. ^{13}C{^{1}H}-apt NMR spectrum (75.48 MHz, chloroform-*d*, 298 K) of $\mathbf{f_6}$.

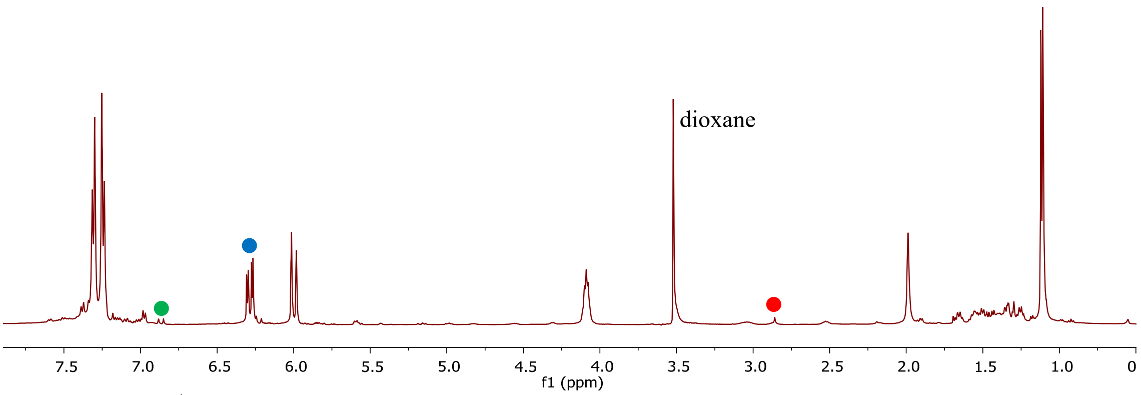

Figure S87. ^{1}H NMR spectrum (500 MHz, benzene-*d*6, 298 K) of the crude reaction mixture of the cross-coupling of 4-(trifluoromethyl)phenylacetylene and 3-butyn-2-ol to give **g$_6$**. Characteristic resonances used for the calculation of the reaction conversion are marked as follows: blue spot, one olefinic proton of **g$_6$**; green spot, one olefinic proton of **f$_4$**; red spot: HC≡ proton of 4-(trifluoromethyl)phenylacetylene.

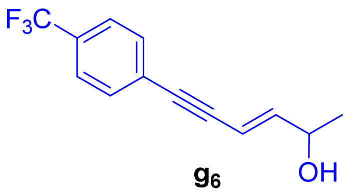

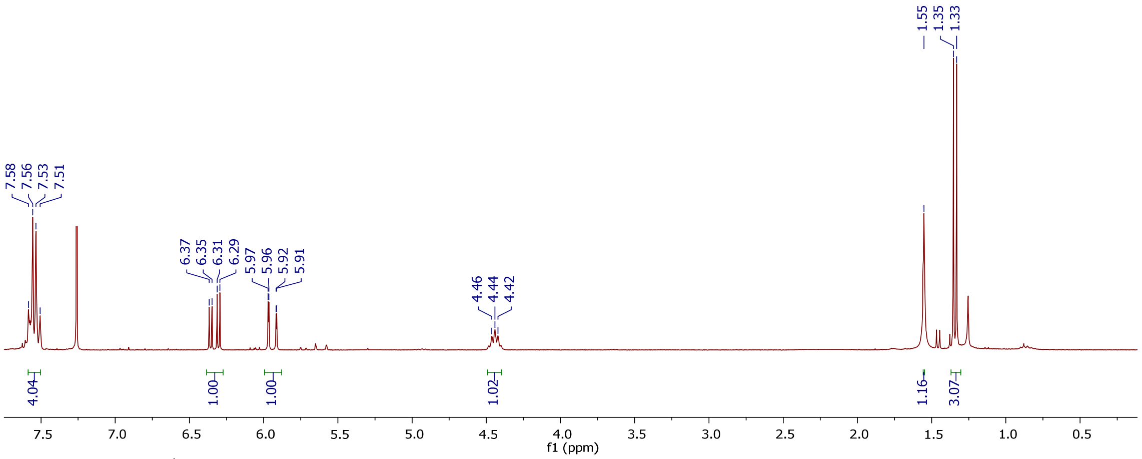

Figure S88. ^{1}H NMR spectrum (300.13 MHz, chloroform-*d*, 298 K) of **g$_6$**.

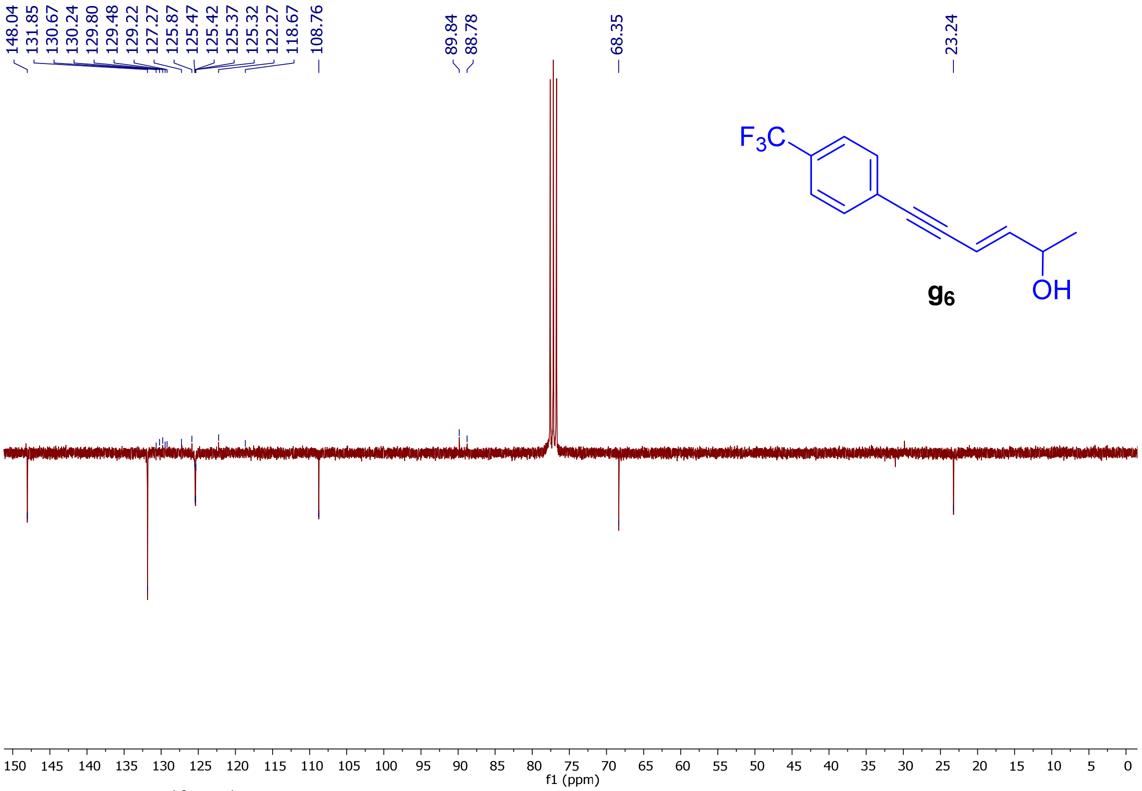

Figure S89. $^{13}C\{^{1}H\}$-apt NMR spectrum (75.48 MHz, chloroform-d, 298 K) of $\mathbf{g_6}$.

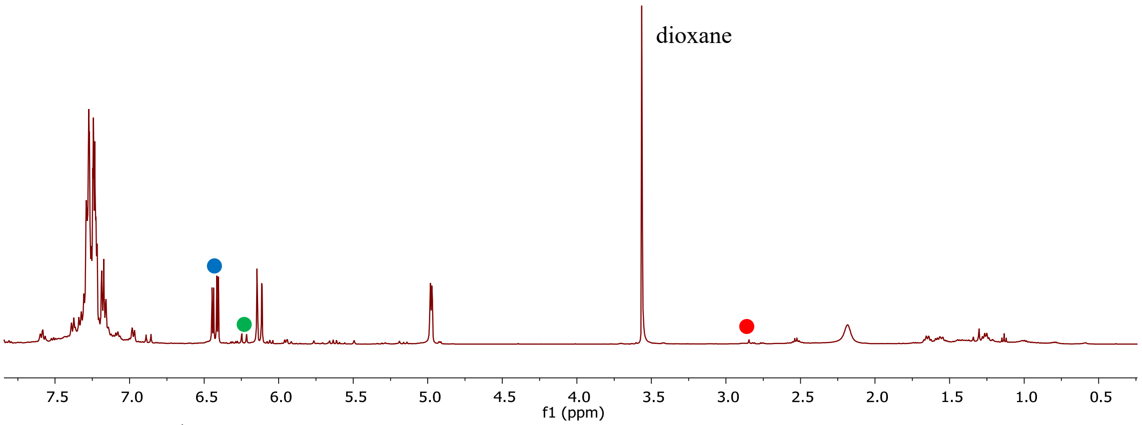

Figure S90. ^{1}H NMR spectrum (500 MHz, benzene-d_6, 298 K) of the crude reaction mixture of the cross-coupling of 4-(trifluoromethyl)phenylacetylene and 1-phenyl-2-propyn-1-ol to give $\mathbf{h_6}$. Characteristic resonances used for the calculation of the reaction conversion are marked as follows: blue spot, one olefinic proton of $\mathbf{h_6}$; green spot, one olefinic proton of $\mathbf{f_4}$; red spot: HC≡ proton of 4-(trifluoromethyl)phenylacetylene.

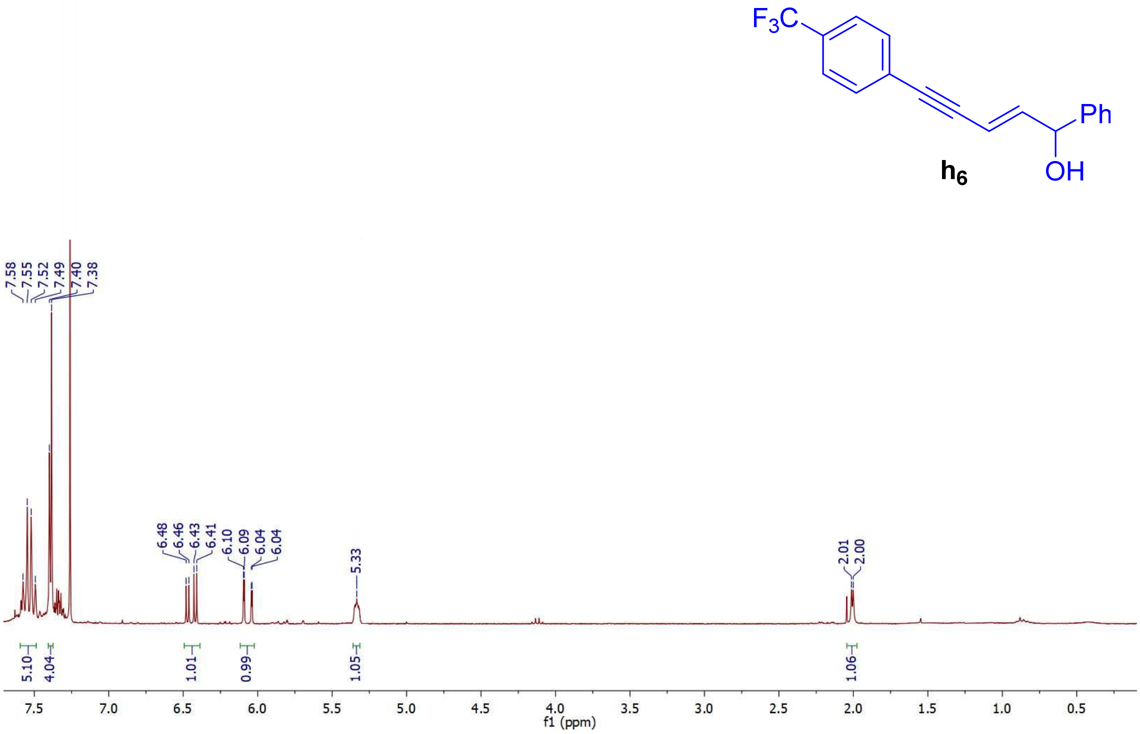

Figure S91. ^{1}H NMR spectrum (300.13 MHz, chloroform-*d*, 298 K) of $\mathbf{h_6}$.

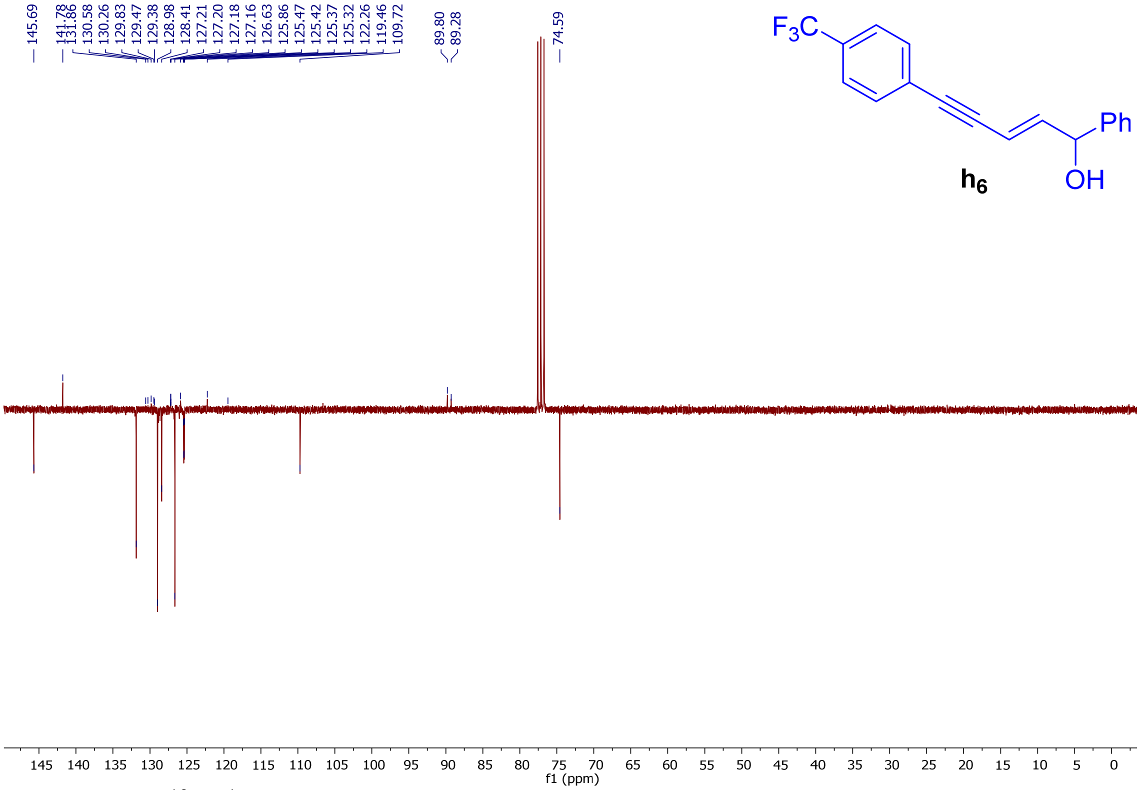

Figure S92. $^{13}C\{^{1}H\}$-apt NMR spectrum (75.48 MHz, chloroform-*d*, 298 K) of $\mathbf{h_6}$.

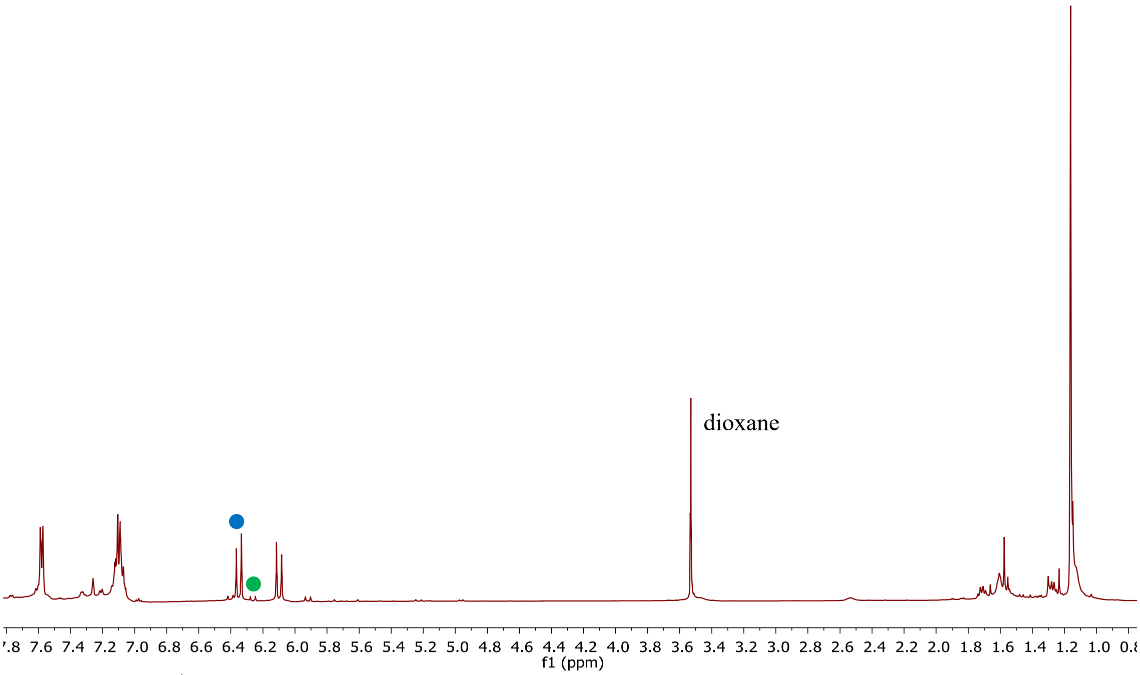

Figure S93. ^{1}H NMR spectrum (500 MHz, benzene-d_6, 298 K) of the crude reaction mixture of the cross-coupling of phenylacetylene and 2-methyl-3-butyn-2-ol to give **i**$_6$. Characteristic resonances used for the calculation of the reaction conversion are marked as follows: blue spot, one olefinic proton of **i**$_6$; green spot, one olefinic proton of **e**$_5$.

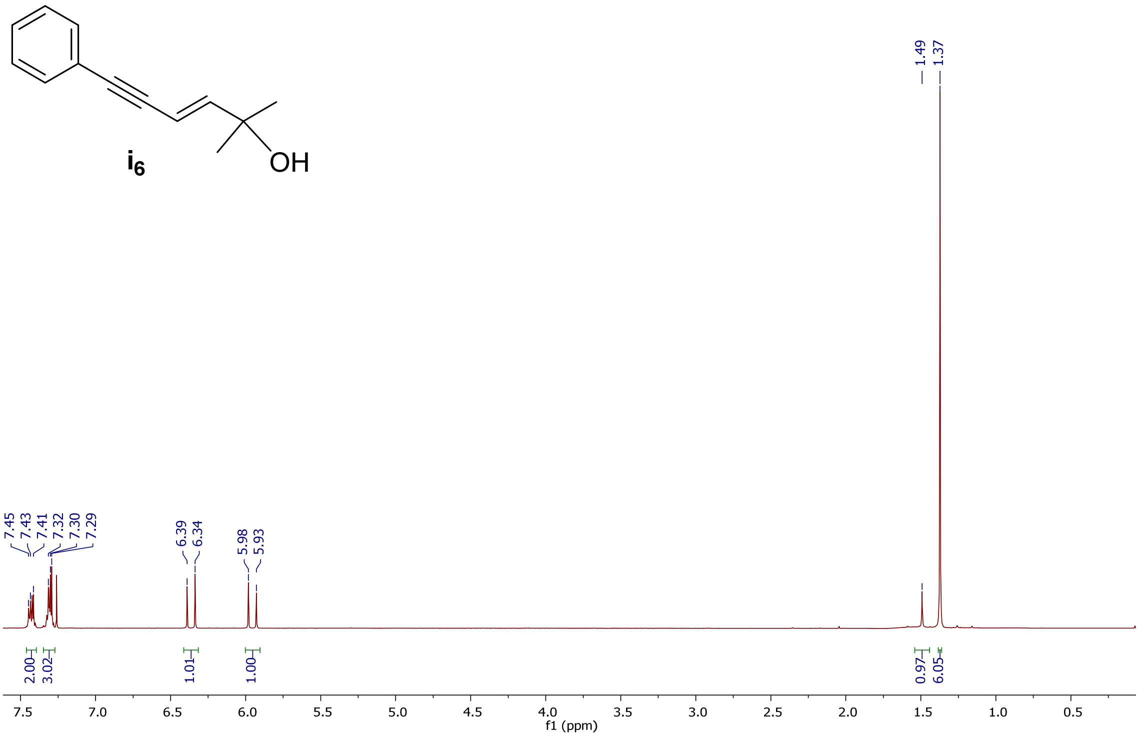

Figure S94. ^{1}H NMR spectrum (300.13 MHz, chloroform-d, 298 K) of **i**$_6$.

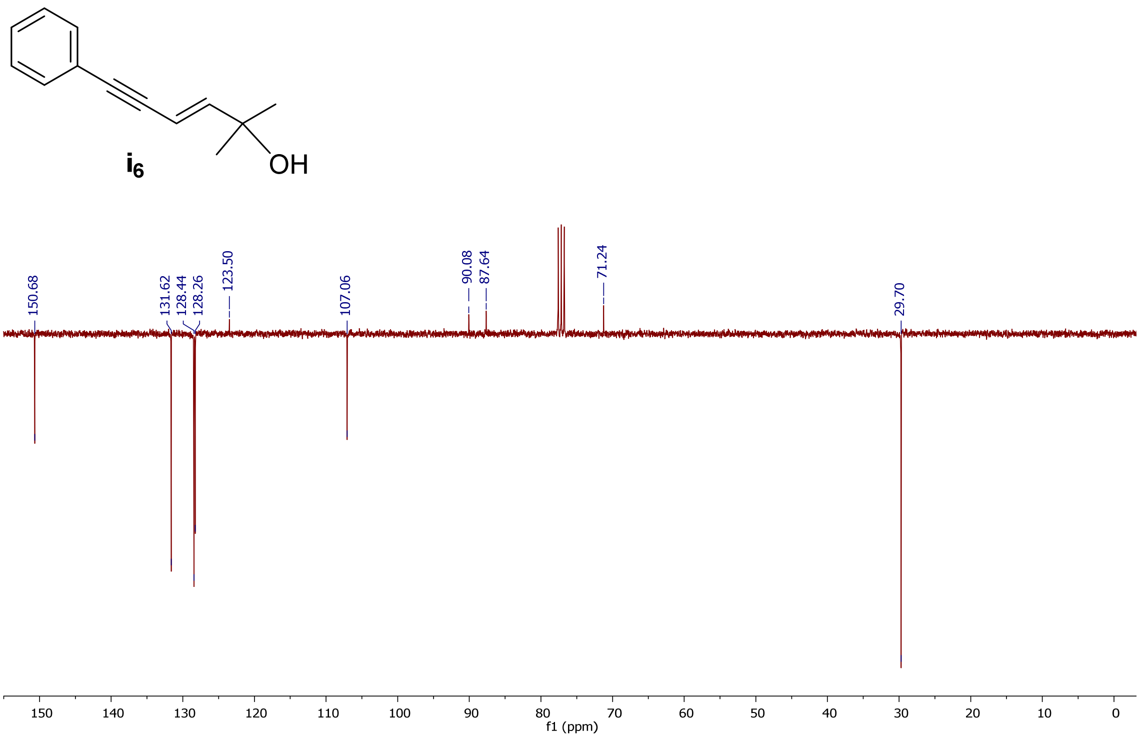

Figure S95. $^{13}C\{^{1}H\}$-apt NMR spectrum (75.48 MHz, chloroform-*d*, 298 K) of $\mathbf{i_6}$.

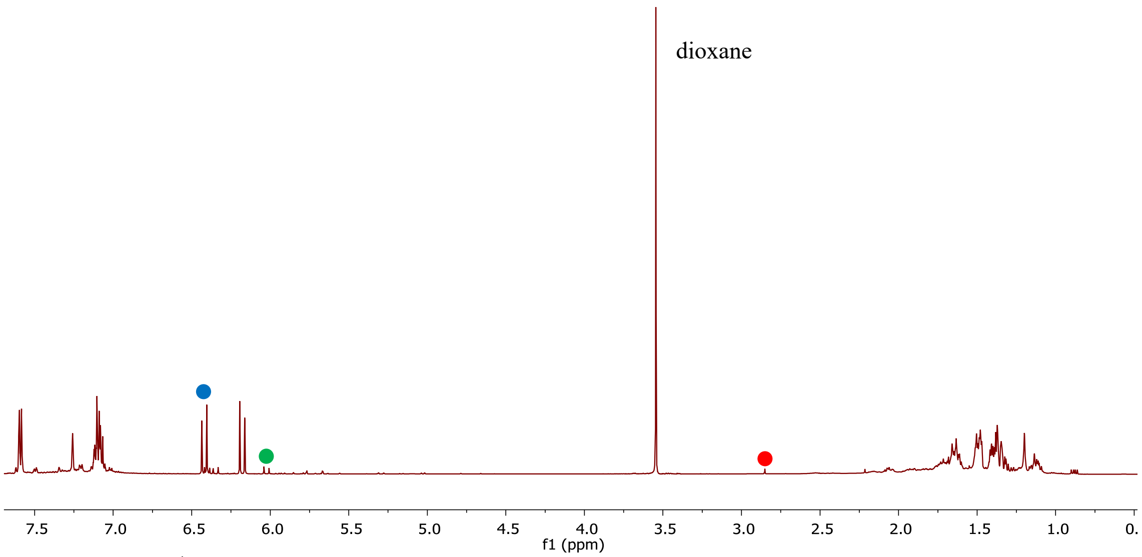

Figure S96. ^{1}H NMR spectrum (500 MHz, benzene-d_6, 298 K) of the crude reaction mixture of the cross-coupling of phenylacetylene and 1-ethynyl-1-cyclohexanol to give $\mathbf{j_6}$. Characteristic resonances used for the calculation of the reaction conversion are marked as follows: blue spot, one olefinic proton of $\mathbf{j_6}$; green spot, one olefinic proton of $\mathbf{g_5}$; red spot; HC≡ proton of phenylacetylene.

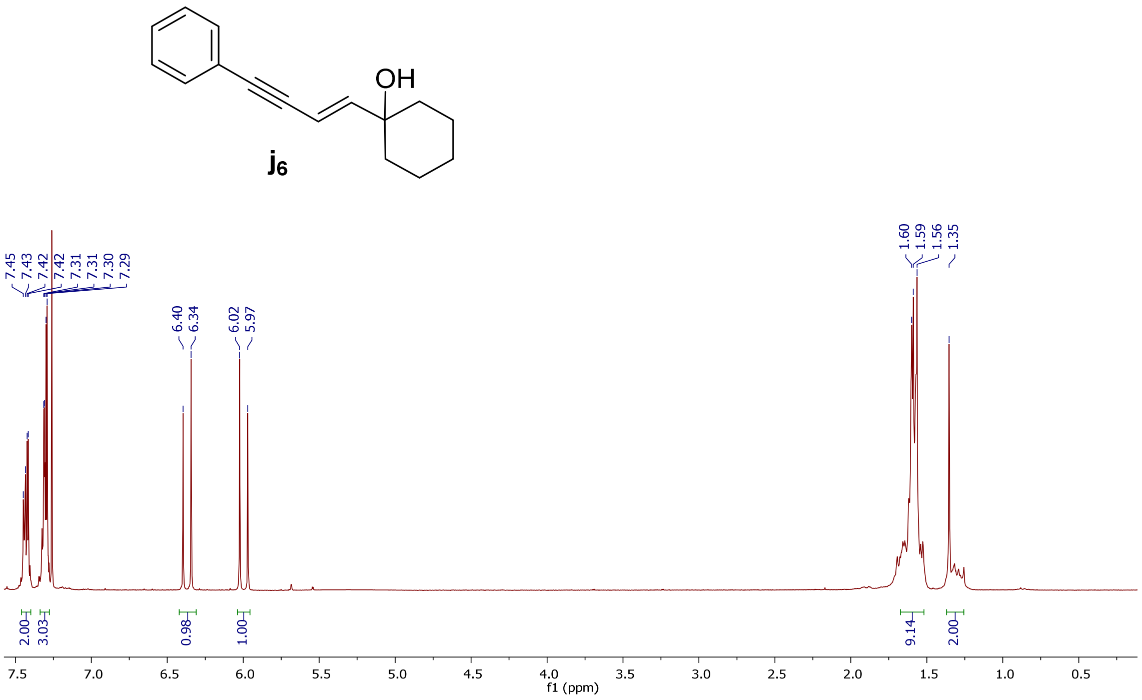

Figure S97. ^{1}H NMR spectrum (300.13 MHz, chloroform-*d*, 298 K) of **j₆**.

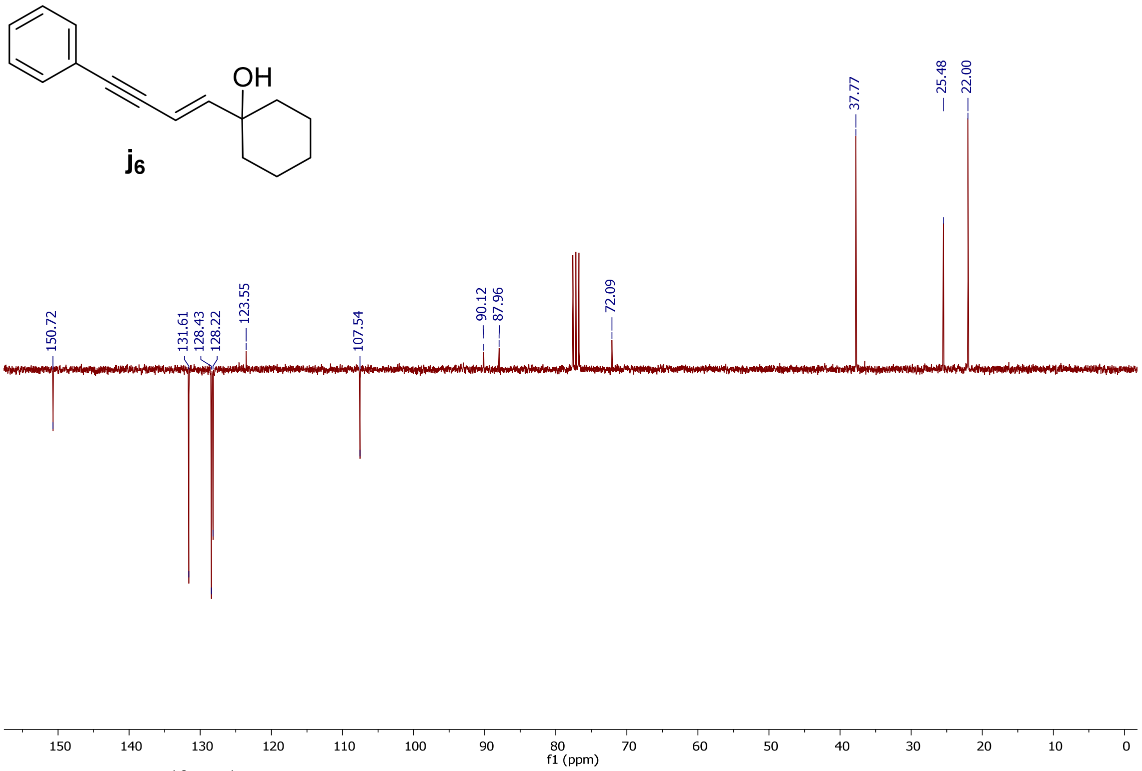

Figure S98. $^{13}C\{^{1}H\}$-apt NMR spectrum (75.48 MHz, chloroform-*d*, 298 K) of **j₆**.

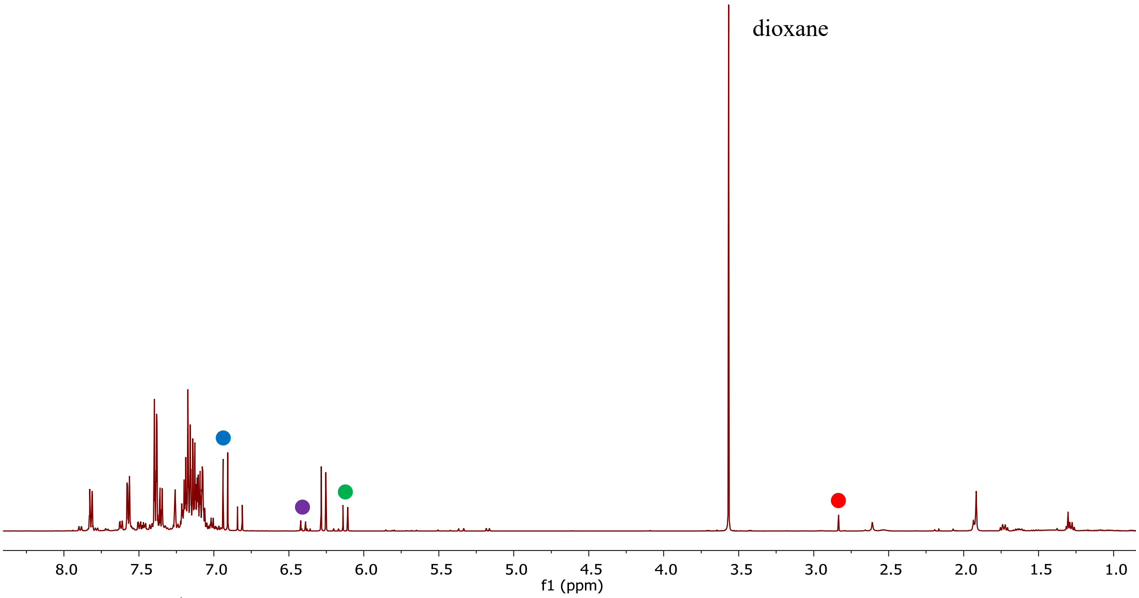

Figure S99. ^{1}H NMR spectrum (500 MHz, benzene-d_6, 298 K) of the crude reaction mixture of the cross-coupling of phenylacetylene and 1,1-diphenyl-2-propyl-1-ol to give **k**$_6$. Characteristic resonances used for the calculation of the reaction conversion are marked as follows: blue spot, one olefinic proton of **k**$_6$; green spot, one olefinic proton of **i**$_5$; violet spot, one olefinic proton of **a**$_4$; red spot; HC≡ proton of phenylacetylene.

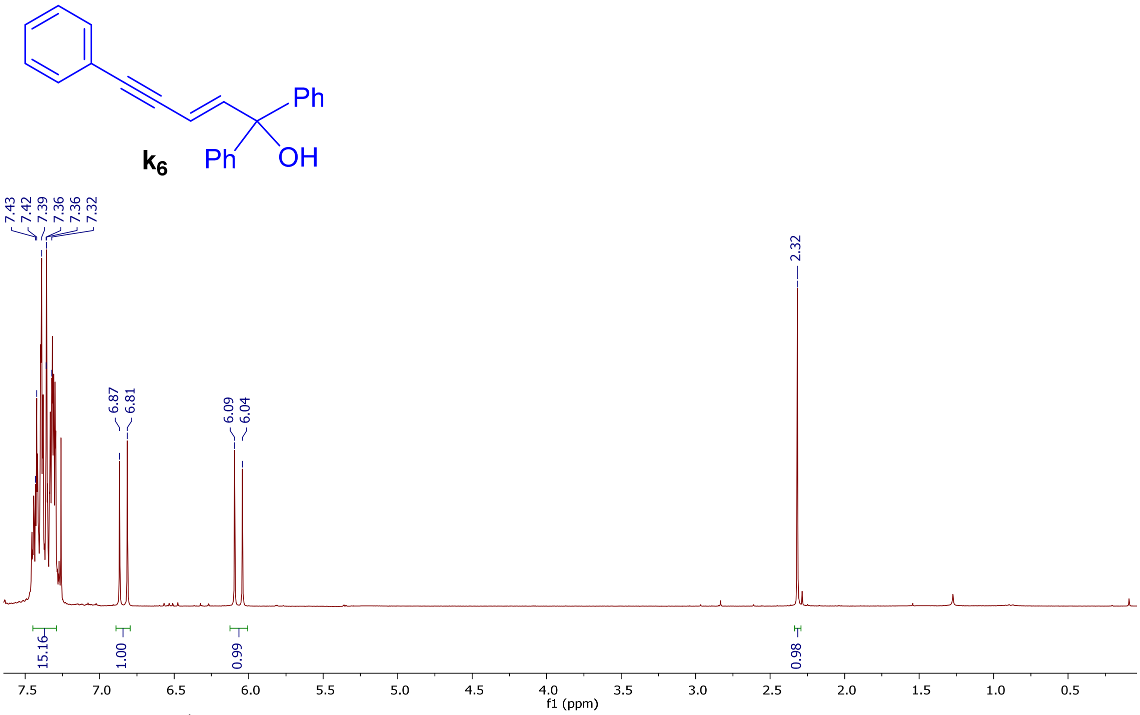

Figure S100. ^{1}H NMR spectrum (300.13 MHz, chloroform-d, 298 K) of **k**$_6$.

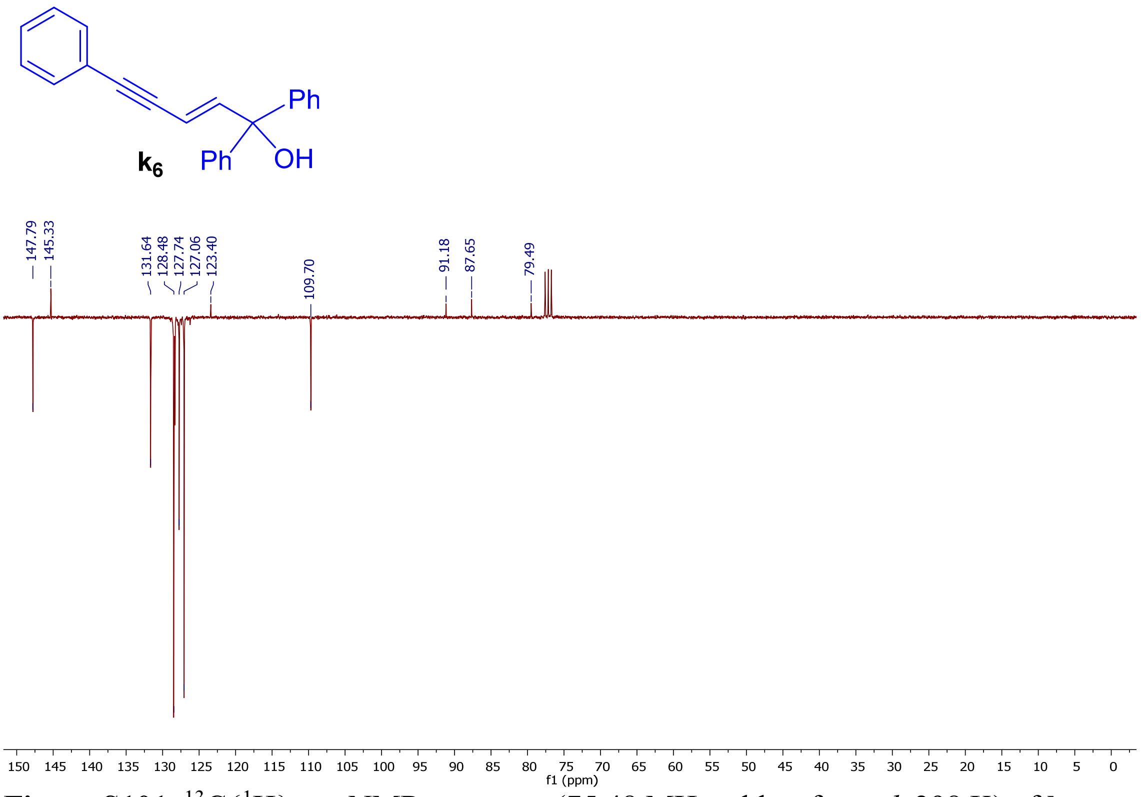

Figure S101. $^{13}C\{^{1}H\}$-apt NMR spectrum (75.48 MHz, chloroform-*d*, 298 K) of **k$_6$**.

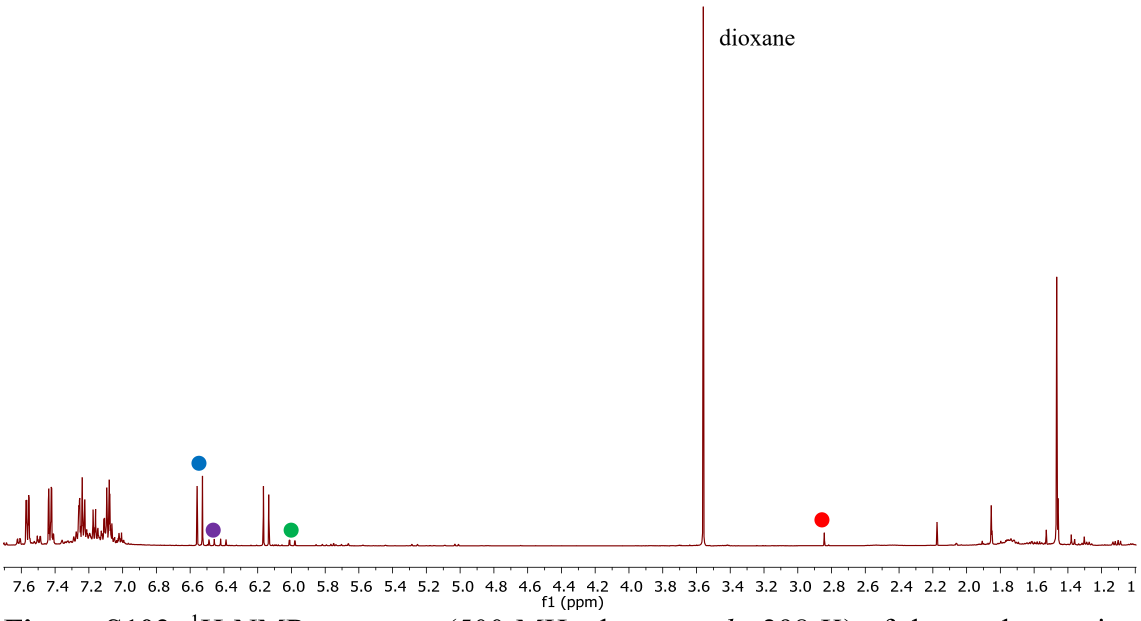

Figure S102. ^{1}H NMR spectrum (500 MHz, benzene-*d*$_6$, 298 K) of the crude reaction mixture of the cross-coupling of phenylacetylene and 2-phenyl-3-butyn-2-ol to give **l$_6$**. Characteristic resonances used for the calculation of the reaction conversion are marked as follows: blue spot, one olefinic proton of **l$_6$**; green spot, one olefinic proton of **h$_5$**; purple spot, one olefinic proton of **a$_4$**; red spot; HC≡ proton of phenylacetylene.

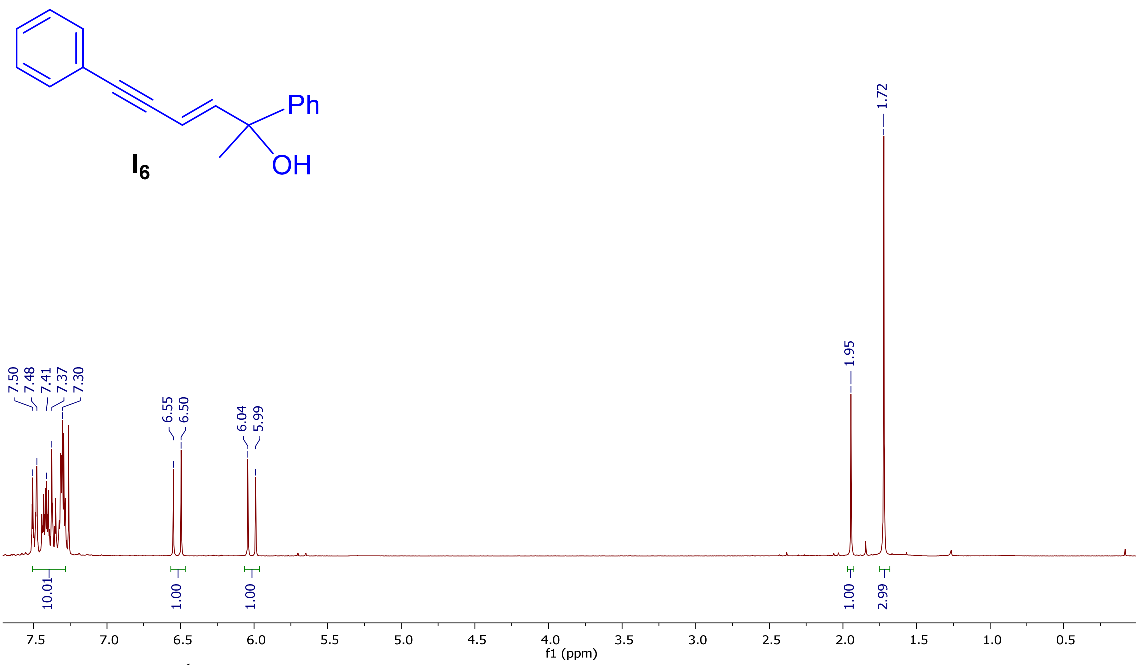

Figure S103. ^{1}H NMR spectrum (300.13 MHz, chloroform-*d*, 298 K) of $\mathbf{I_6}$.

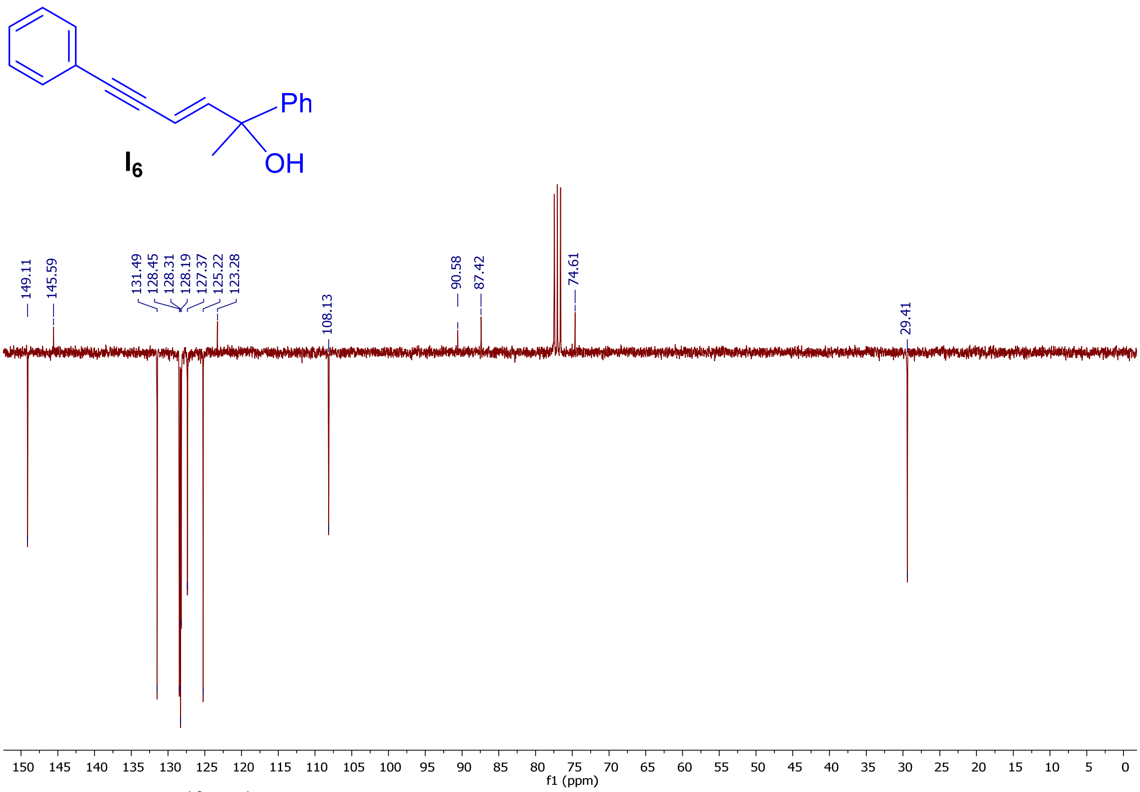

Figure S104. $^{13}C\{^{1}H\}$-apt NMR spectrum (75.48 MHz, chloroform-*d*, 298 K) of $\mathbf{I_6}$.

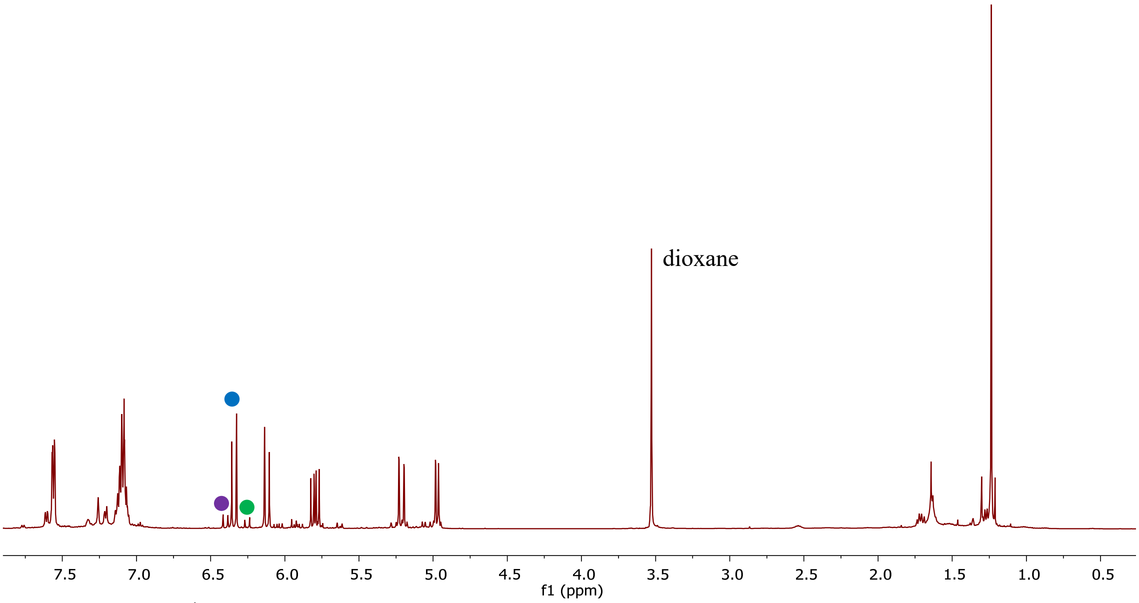

Figure S105. ^{1}H NMR spectrum (500 MHz, benzene-d_6, 298 K) of the crude reaction mixture of the cross-coupling of phenylacetylene and 3-methyl-1-penten-4-yn-3-ol to give $\mathbf{m_6}$. Characteristic resonances used for the calculation of the reaction conversion are marked as follows: blue spot, one olefinic proton of $\mathbf{m_6}$; green spot, one olefinic proton of $\mathbf{d_5}$; purple spot, one olefinic proton of $\mathbf{a_4}$.

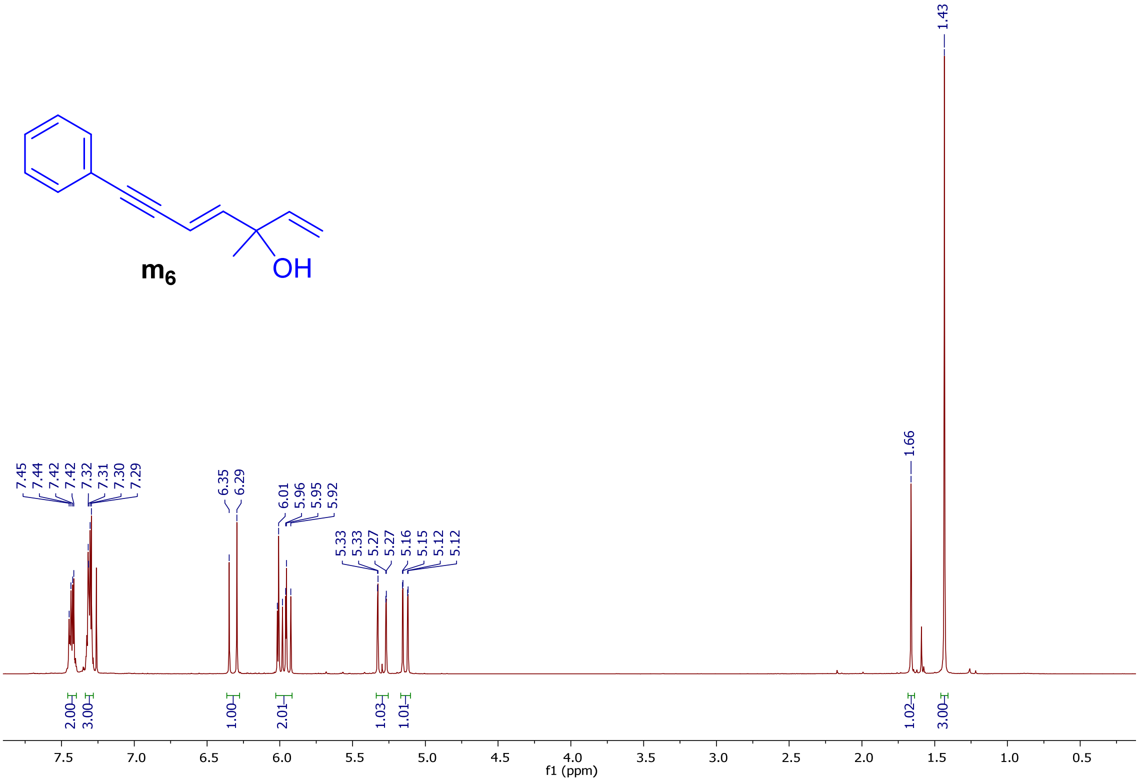

Figure S106. ^{1}H NMR spectrum (300.13 MHz, chloroform-d, 298 K) of $\mathbf{m_6}$.

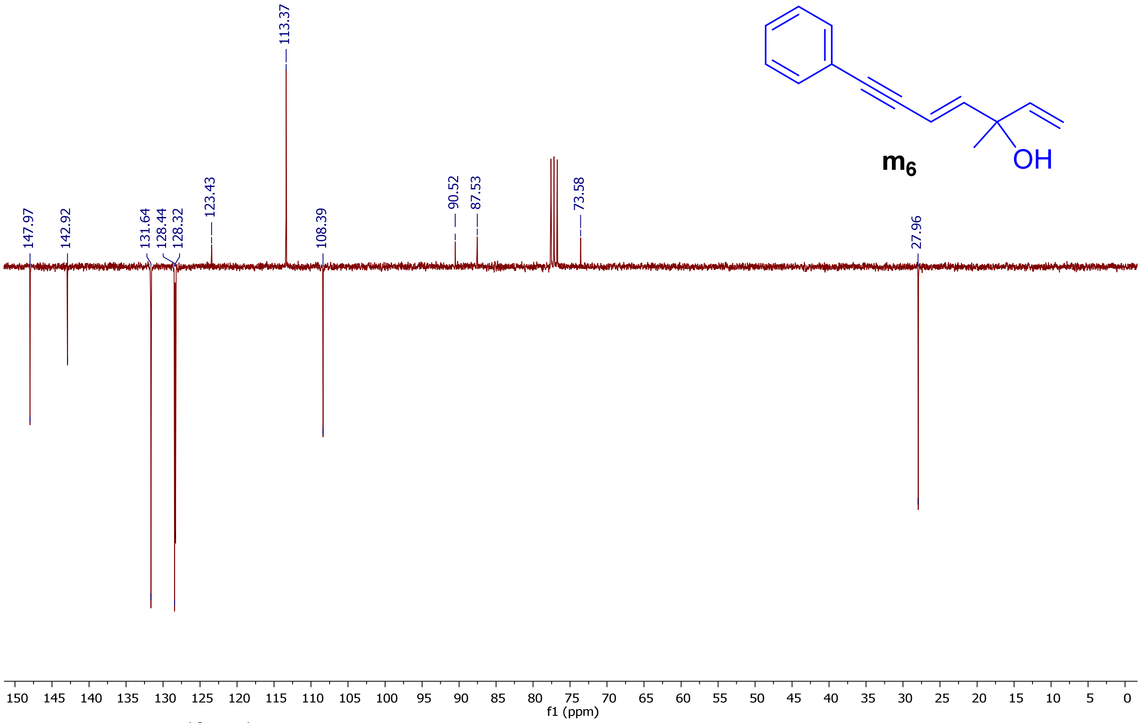

Figure S107. $^{13}C\{^{1}H\}$-apt NMR spectrum (75.48 MHz, chloroform-*d*, 298 K) of $\mathbf{m_6}$.

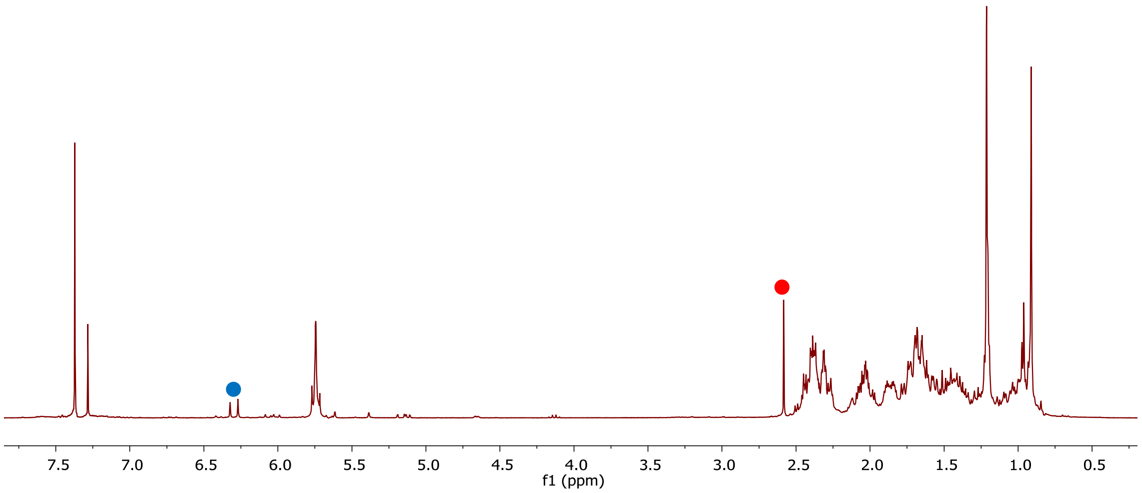

Figure S108. ^{1}H NMR spectrum (300.13 MHz, chloroform-*d*, 298 K) of the crude reaction mixture of the homo-coupling of ethisterone to give $\mathbf{a_8}$. Characteristic resonances used for the calculation of the reaction conversion are marked as follows: blue spot, one olefinic proton of $\mathbf{a_8}$; red spot; HC≡ proton of ethisterone.

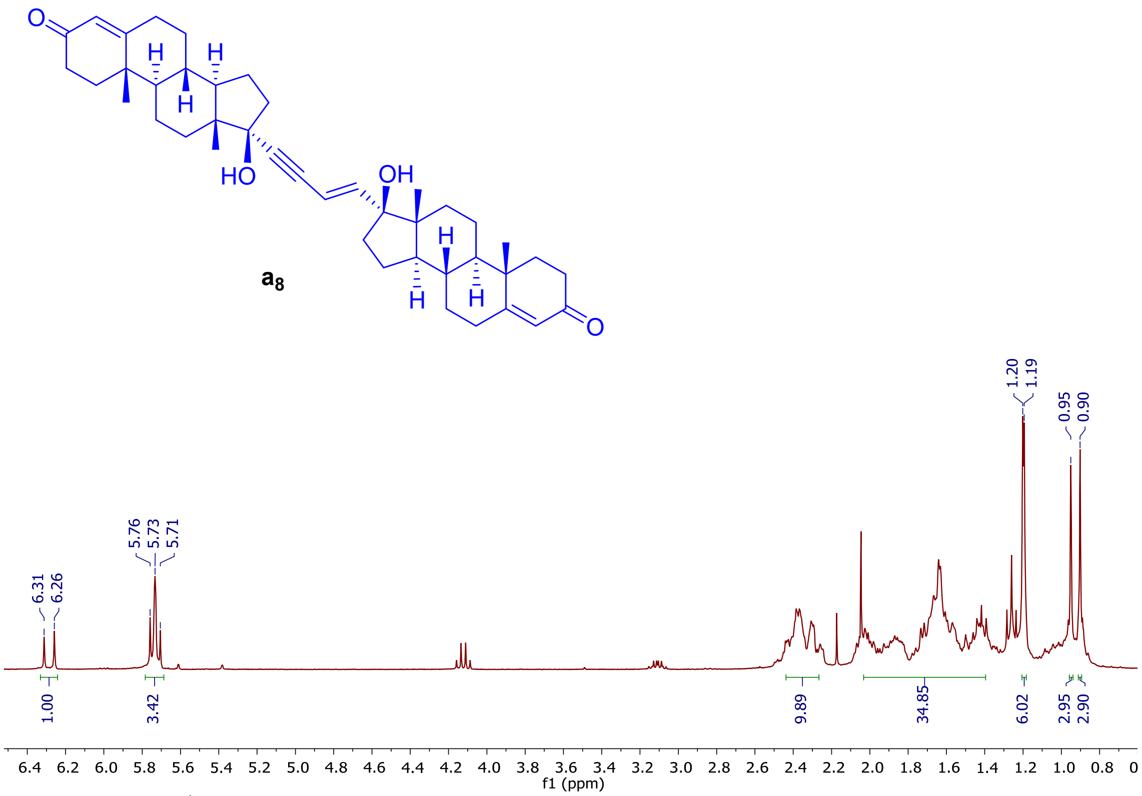

Figure S109. ^{1}H NMR spectrum (300.13 MHz, chloroform-d, 298 K) of $\mathbf{a_8}$.

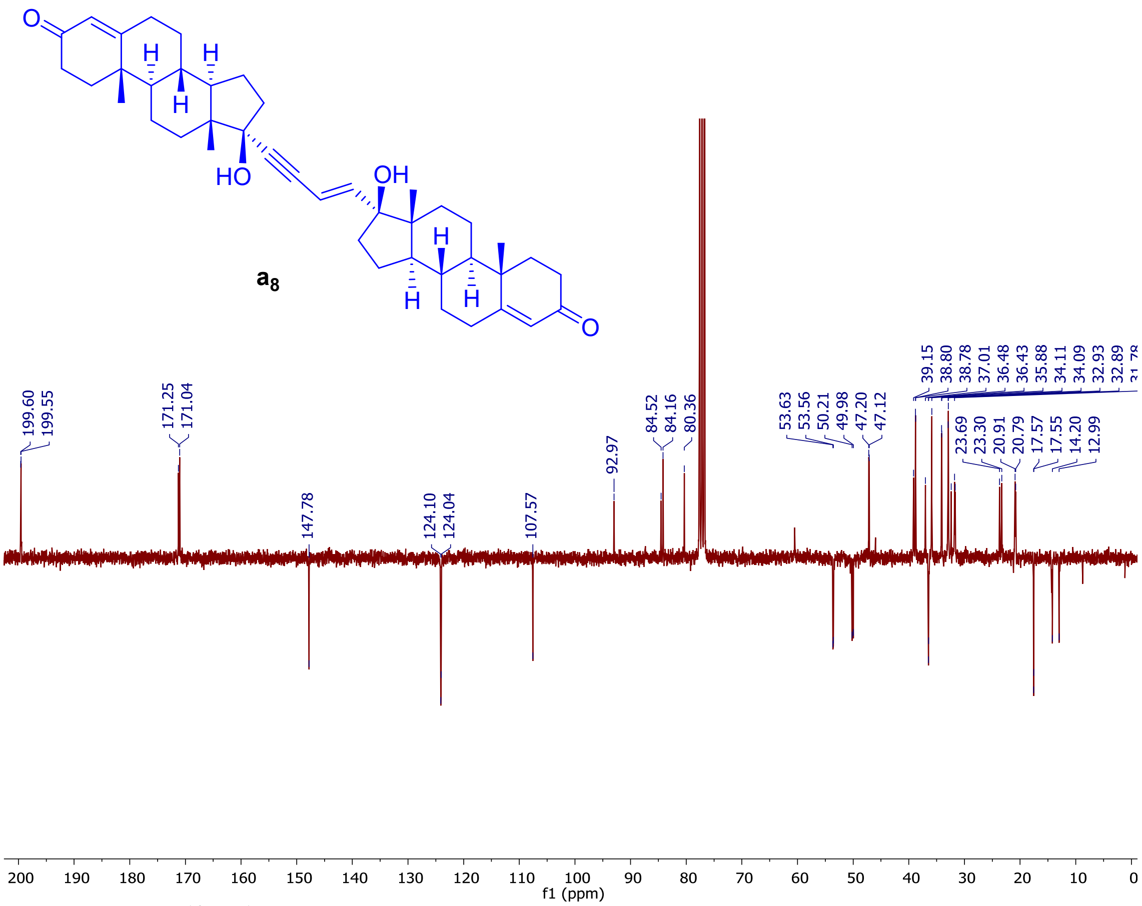

Figure S110. ^{13}C{^{1}H}-apt NMR spectrum (75.48 MHz, chloroform-d, 298 K) of $\mathbf{a_8}$.

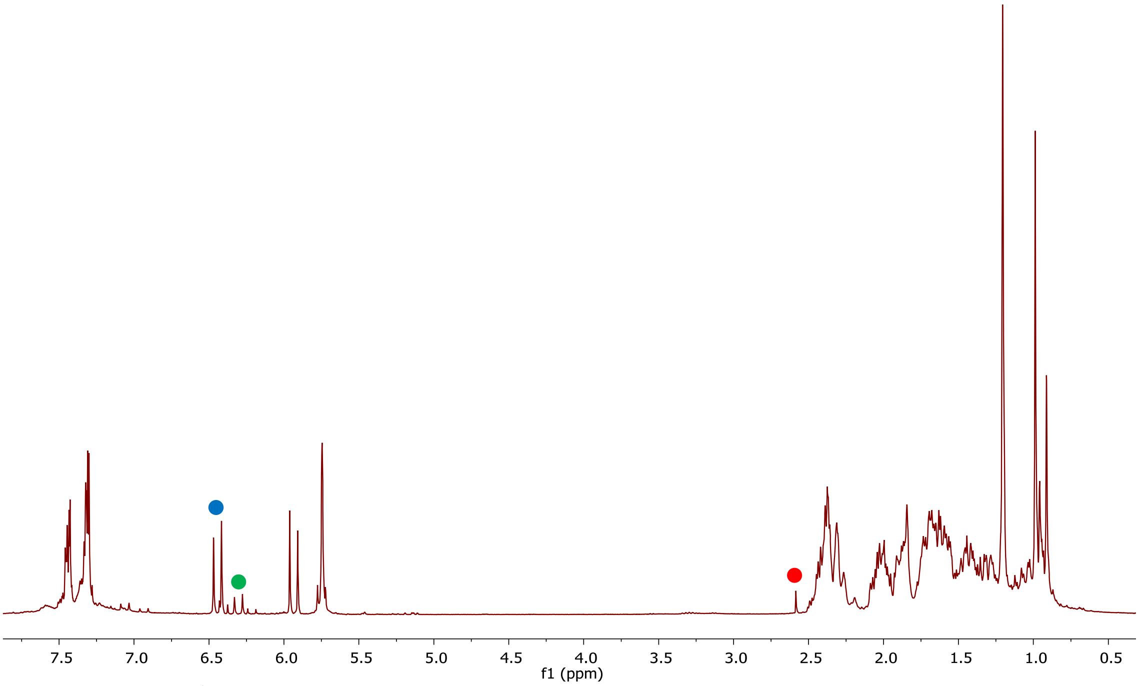

Figure S111. ^{1}H NMR spectrum (300.13 MHz, chloroform-*d*, 298 K) of the crude reaction mixture of the cross-coupling of phenylacetylene and ethisterone to give **b$_8$**. Characteristic resonances used for the calculation of the reaction conversion are marked as follows: blue spot, one olefinic proton of **b$_8$**; green spot, one olefinic proton of **a$_8$**; red spot; HC≡ proton of ethisterone.

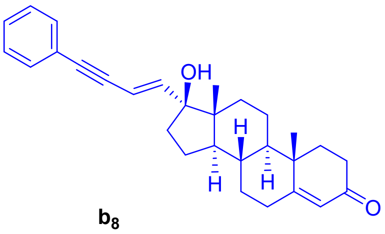

b$_8$

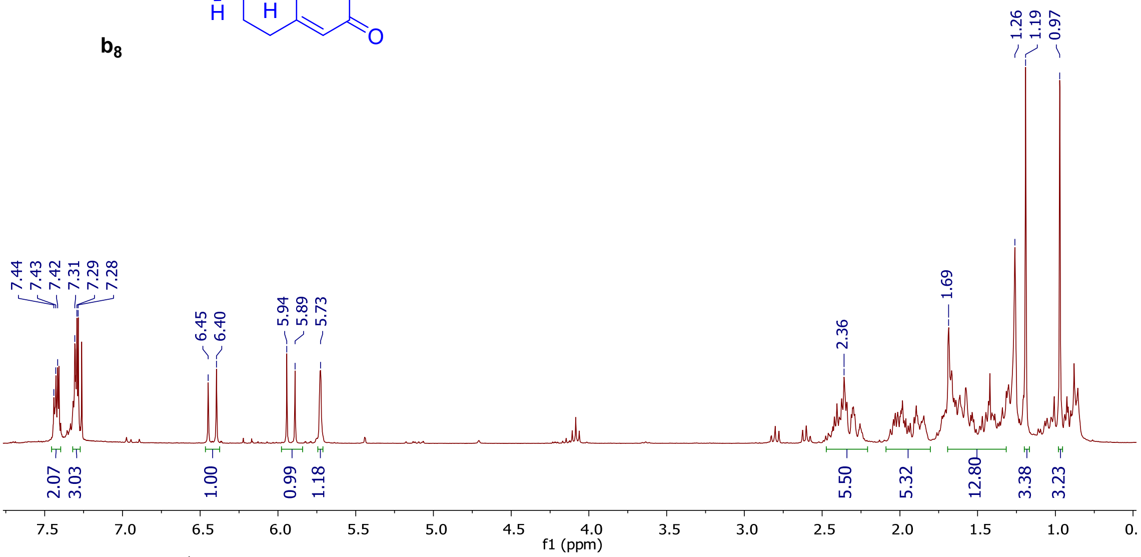

Figure S112. ^{1}H NMR spectrum (300.13 MHz, chloroform-*d*, 298 K) of **b$_8$**.

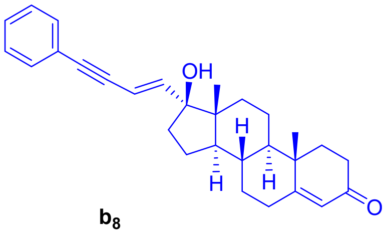

b8

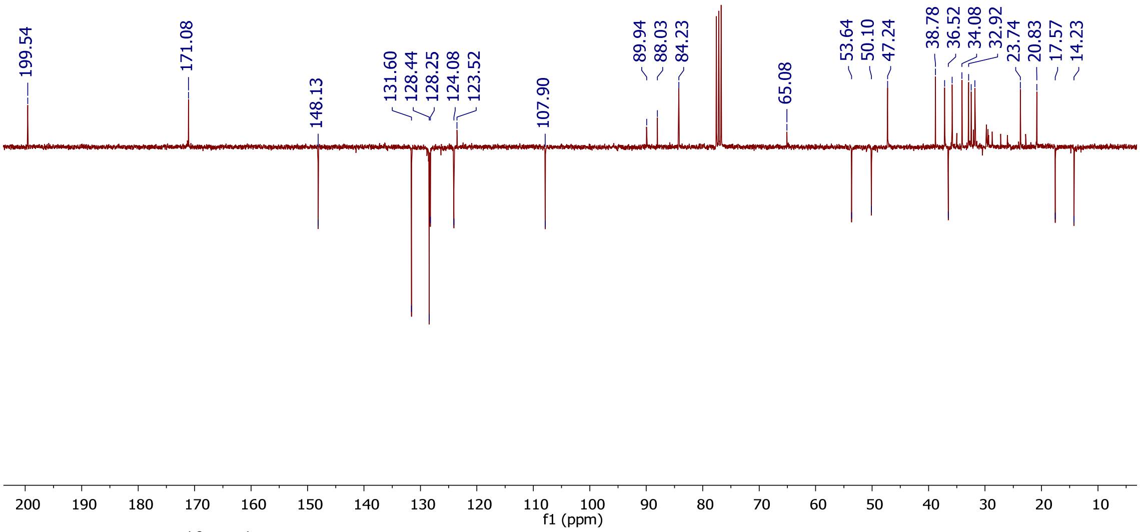

Figure S113. $^{13}C\{^{1}H\}$-apt NMR spectrum (75.48 MHz, chloroform-*d*, 298 K) of **b8**.

• Structural Analysis of Complex 6.

X-ray data were collected on a D8 Venture Bruker diffractometer (Mo radiation, λ = 0.71073 Å). The crystal was mounted under oil in a MiTeGen mount and cooled to 100(2) K with an open-flow nitrogen gas (Oxford Cryosystems). Data were collected using φ and ω narrow scans. Diffracted intensities were integrated and corrected for absorption effects using SAINT[16] and SADABS[17] programs, included in APEX4 package. The structures were solved by direct methods and refined by full-matrix least squares on F2 with SHELXL2019,[18] including isotropic and subsequently anisotropic displacement parameters. The hydrogen atoms were observed in the last Fourier Maps or calculated, and refined freely or using a restricted riding model.

The crystal turns out to be a twin, with the second largest component resulting from a rotation of 179.9 degrees about the reciprocal axis 0.000 0.002 1.000. It was not possible to refine the crystal with two domains (HKLF 5) due to the overlapping of the spots. Although tried, better quality crystals could not be obtained.

Crystal data for **6** (CCDC 2283937): $C_{72}H_{75}O_4P_2Rh$, 0.5(C_5H_{12}), M_W 1204.73, colorless, irregular block, (0.198 x 0.101 x 0.094 mm^3), monoclinic, space group P2/c, *a*: 24.021(3) Å, *b*: 10.9070(15) Å, *c*: 46.819(7) Å, *β*: 91.274(4)°, *V* = 12263(3) Å^3, *Z* = 8, *Z'* = 2, D_{calc}: 1.305 g cm^{-3}, F(000): 5076, T = 100(2) K, μ 0.381 mm^{-1}. 213203 measured reflections (2θ: 3-51°, ω and φ scans 0.5°), 22828 unique (R_{int} = 0.0791); min./max. transm. factors 0.602/0.737. Final agreement factors were R^1 = 0.1444 (21633 observed reflections, I > 2σ(I)) and wR^2 = 0.3326; data/restraints/parameters 22828/112/1472; GoF = 1.239. Largest peak and hole 3.131 and -4.121 e/ Å^3.

• Computational Details

All calculations were performed at the DFT level using the B3LYP functional[19] supplemented with the Grimme's dispersion correction D3[20] as implemented in Gaussian09.[21] Rh atoms were described by means of an effective core potential SDD for the inner electron[22] and its associated double-ζ basis set for the outer ones, complemented with a set of f-polarization functions for rhodium.[23] The 6-31G** basis set was used for all the other atoms.[24] Reactants, intermediates, and products were also characterized by frequency calculations and has positive definite Hessian matrices thus confirming that the computed structure is a minimum on the potential energy surface. Transition states were identified by having one imaginary frequency in the Hessian matrix. It was confirmed that transition states connect with the corresponding intermediates by means of application of an eigenvector corresponding to the imaginary frequency and subsequent optimization of the resulting structures. Gibbs energies were computed at 298.15 K and 1 atmosphere. All values collected in figures correspond to Gibbs energies in toluene in kcal mol^{-1}.

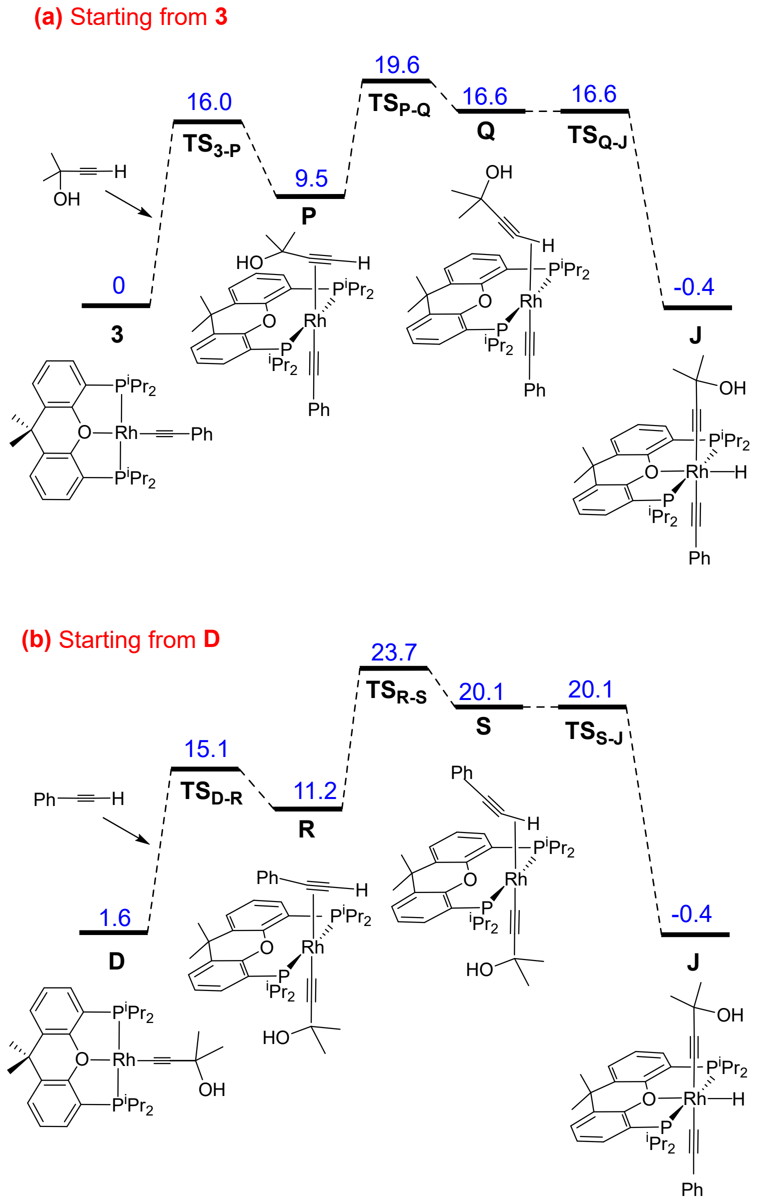

Figure S114. Computed energy profiles (ΔG, in kcal mol^{-1}) for the oxidative additions of both alkynes to **3** and **D** to give **J**.

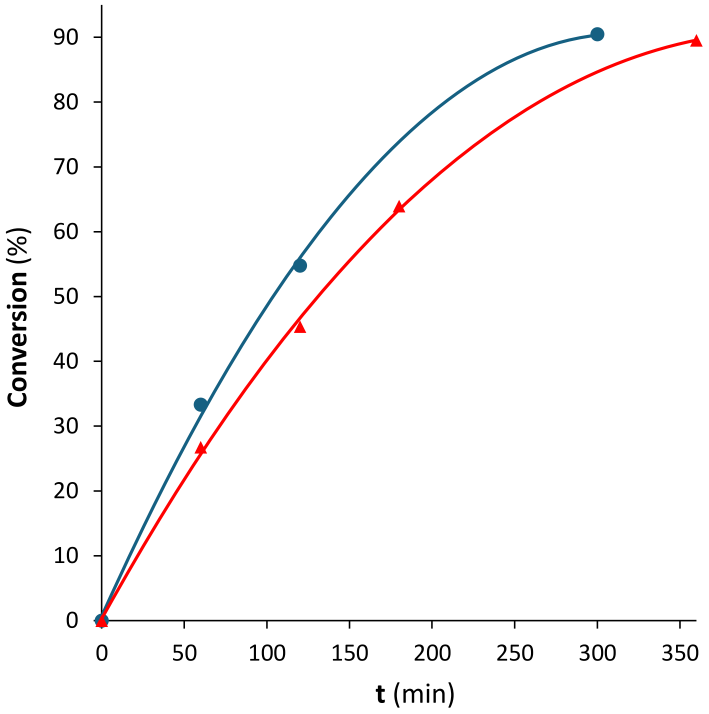

Figure S115. Profiles of the dimerization of phenylacetylene (red) and 2-methyl-3-butyn-2-ol (blue). Conditions: **1** (5 mg, 0.009 mmol), alkyne (0.20 mmol), benzene-d_6 (0.4 mL), 80 ºC.

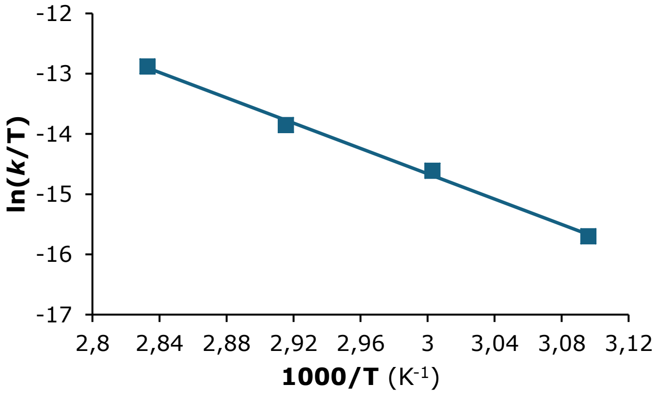

Figure S116. Eyring plot for the reductive elimination of (*E*)-1,1,6,6-tetraphenyl-2-hexen-4-yne-1,6-diol from complex **6**. The following activation parameters are derived from it: $\Delta H^{\neq}$ = 20.9 ± 2.1 kcal mol^{-1}, $\Delta S^{\neq}$ = -13.7 ± 6.1 cal^{-1} K^{-1} mol^{-1}, and $^{298}\Delta G^{\neq}$ = 25.0 ± 3.9 kcal mol^{-1}

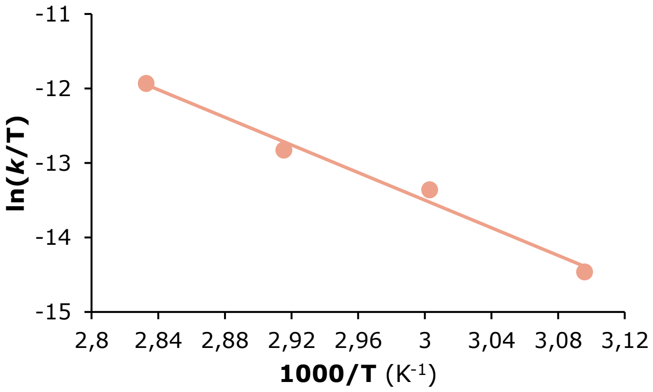

Figure S117. Eyring plot for the catalytic dimerization of 1,1-diphenyl-2-propyn-1-ol promoted by complex **6**. The following activation parameters are derived from it: $\Delta H^{\neq}$ = 18.4 ± 1.9 kcal mol^{-1}, $\Delta S^{\neq}$ = -18.8 ± 5.6 cal^{-1} K^{-1} mol^{-1}, and $^{298}\Delta G^{\neq}$ = 24.0 ± 3.6 kcal mol^{-1}.

• References

(1) Esteruelas, M. A.; Martínez, A.; Oliván, M.; Vélez, A. A General Rhodium Catalyst for the Deuteration of Boranes and Hydrides of the Group 14 Elements. *J. Org. Chem.* **2020**, *85*, 15693-15698.

(2) NMR data agree with those reported previously. See: Buxaderas, E.; Alonso, D. A.; Najera, C. Microwave-assisted palladium-catalyzed highly regio-and stereoselective head to head dimerization of terminal aryl alkynes in water. *RSC Advances*. **2014**, *4*, 46508-46512.

(3) NMR data agree with those reported previously. See: Li, X.; Chen, X. L.; Zhang, Q.; Qu, L. B.; Bi, W. Z.; Sun, K.; Chen J. Y.; Chem X.; Zhao, Y. F. $CuSO_4$-H-phosphonate catalyzed highly stereo-and regioselective dimerization of terminal alkynes. *RSC Adv.* **2015**, *5*, 5004-5009.

(4) NMR data agree with those reported previously. See: Rubina, M.; Gevorgyan, V. Can agostic interaction affect regiochemistry of carbopalladation? Reverse regioselectivity in the palladium-catalyzed dimerization of aryl acetylenes. *J. Am. Chem. Soc.* **2001**, *123*, 11107-11108.

(5) NMR data agree with those reported previously. Weber, S. M.; Queder, J.; Hilt, G. Ligand-Controlled Diastereoselective Cobalt-Catalysed Hydroalkynylation of Terminal Alkynes to *E*-or Z-1, 3-Enynes. *Chem. Eur. J.* **2020**, *26*, 12129-12133.

(6) NMR data agree with those reported previously. Slugovc, C.; Doberer, D.; Gemel, C.; Schmid, R.; Kirchner, K.; Winkler, B.; Stelzer, F. Ruthenium Catalyzed Homocoupling of Terminal Alkynes. *Monatsh. Chem.* **1998**, *129*, 221-233.

(7) NMR data agree with those reported previously. Rubio-Pérez, L.; Azpíroz, R.; Di Giuseppe, A.; Polo, V.; Castarlenas, R.; Pérez-Torrente, J. J.; Oro, L. A. Pyridine-Enhanced Head-to-Tail Dimerization of Terminal Alkynes by a Rhodium–N-Heterocyclic-Carbene Catalyst. *Chem. Eur. J.* **2013**, *19*, 15304-15314.

(8) NMR data agree with those reported previously. Schmitt, H. J.; Singer, H. Die dimerisierung von endständigen α-hydroxyacetylenen mit rhodiumkomplexkatalysatoren. *J. Organomet. Chem.* **1978**, *153*, 165-179.

(9) NMR data agree with those reported previously. Trostyanskaya, I. G.; Beletskaya, I. P. A copper (I or II)/diethylphosphite catalytic system for base-free additive dimerization of alkynes. *Tetrahedron*. **2017**, *73*, 148-153.

(10) NMR data agree with those reported previously. Maleczka, R. E.; Terrell, L. R.; Clark, D. H.; Whitehead, S. L.; Gallagher, W. P.; Terstiege, I. Application of Fluoride-Catalyzed Silane Reductions of Tin Halides to the in Situ Preparation of Vinylstannanes. *J. Org. Chem*. **1999**, *64*, 5958-5965.

(11) NMR data agree with those reported previously. Zatolochnaya, O. V.; Gordeev, E. G.; Jahier, C.; Ananikov, V. P.; Gevorgyan, V. Carboxylate Switch between Hydro- and Carbopalladation Pathways in Regiodivergent Dimerization of Alkynes. *Chem. Eur. J.* **2014**, *20*, 9578-9588.

(12) NMR data agree with those reported previously. Albert, B. J.; Sivaramakrishnan, A.; Naka, T.; Koide, K. Total synthesis of FR901464, an antitumor agent that regulates the transcription of oncogenes and tumor suppressor genes. *J. Am. Chem. Soc.* **2006**, *128*, 2792-2793.

(13) This compound has been used previously as a reaction intermediate. See: Li, S.-Y.; Zhang, X.; Teng, F.; Li, Y.; Li, J.-H. Rh(III)-Catalyzed [3 + 2]/[4 + 2] annulation of acetophenone oxime ethers with 3-acetoxy-1,4-enynes involving C-H activation. *Org. Chem. Front.* **2021**, *8*, 2955-2962. However, no spectroscopic data were reported.

(14) NMR data agree with those reported previously. Singer, H.; Wilkinson, G. The dimerisation of monosubstituted α-hydroxyacetylenes by use of tris (triphenylphosphine) chlororhodium (I) as catalyst. *J. Chem. Soc. A* **1968**, 849-853.

(15) NMR data agree with those reported previously. Xu, H. D.; Zhang, R. W.; Li, X.; Huang, S.; Tang, W.; Hu, W. H. Rhodium-Catalyzed Chemo-and Regioselective Cross Dimerization of Two Terminal Alkynes. *Org. Lett.* **2013**, *15*, 840-843.

(16) SAINT+, version 6.01: Area-Detector Integration Software, Bruker AXS, Madison,WI, 2001.

(17) Blessing, R. H. *Acta Crystallogr.* **1995**, *A51*, 33. SADABS: Area-detector absorption correction; Bruker- AXS, Madison, WI, 1996.

(18) SHELXL-2019/1. Sheldrick, G. M. *Acta Cryst.* **2008**, *A64*, 112-122.

(19) (a) Lee, C.; Yang, W.; Parr, R. G. Development of the Colle-Salvetti correlation energy formula into a functional of the electron density. *Phys. Rev. B* **1988**, *37*, 785-789. (b) Becke, A. D. Density-functional exchange-energy approximation with correct asymptotic behavior. *J. Chem. Phys.* **1993**, *98*, 5648–5652. (c) Stephens, P. J.; Devlin, F. J.; Chabalowski, C. F.; Frisch, M. J. Ab Initio Calculation of Vibrational Absorption and Circular Dichroism Spectra Using Density Functional Force Fields. *J. Phys. Chem.* **1994**, *98*, 11623-11627.

(20) Grimme, S.; Antony, J.; Ehrlich, S.; Krieg, H. A consistent and accurate ab initio parametrization of density functional dispersion correction (DFT-D) for the 94 elements H-Pu. *J. Chem. Phys.* **2010**, *132*, 154104.

(21) Gaussian 09, Revision D.01, Frisch, M. J.; Trucks, G. W.; Schlegel H. B.; Scuseria, G. E.; Robb, M. A.; Cheeseman, J. R.; Scalmani, G.; Barone, V.; Mennucci, B.; Petersson, G. A.; Nakatsuji, H.; Caricato, M.; Li, X.; Hratchian, H. P.; Izmaylov, A. F.; Bloino, J.; Zheng, G.; Sonnenberg, J. L.; Hada, M.; Ehara, M.; Toyota, K.; Fukuda, R.;

Hasegawa, J.; Ishida, M.; Nakajima, T.; Honda, Y.; Kitao, O.; Nakai, H.; Vreven, T.; Montgomery, J. A.; Peralta, Jr., J. E.; Ogliaro, F.; Bearpark, M.; Heyd, J. J.; Brothers, E.; Kudin, K. N.; Staroverov, V. N.; Keith, T.; Kobayashi, R.; Normand, J.; Raghavachari, K.; Rendell, A.; Burant, J. C.; Iyengar, S. S.; Tomasi, J.; Cossi, M.; Rega, N.; S43 Millam, J. M.; Klene, M.; Knox, J. E.; Cross, J. B.; Bakken, V.; Adamo, C.; Jaramillo, J.; Gomperts, R.; Stratmann, R. E.; Yazyev, O.; Austin, A. J.; Cammi, R.; Pomelli, C.; Ochterski, J. W.; Martin, R. L.; Morokuma, K.; Zakrzewski, V. G.; Voth, G. A.; Salvador, P.; Dannenberg, J. J.; Dapprich, S.; Daniels, A. D.; Farkas, O.; Foresman, J. B.; Ortiz, J. V.; Cioslowski, J.; Fox, D. J. Gaussian, Inc., Wallingford CT, **2013**.

(22) Andrea, D.; Häußermann, U. M.; Dolg, M.; Stoll, H.; Preuss, H. Energy adjusted ab initio pseudopotentials for the second and third row transition elements. *Theor. Chim. Acta* **1990**, *77*, 123-141.

(23) Ehlers, A. W.; Bohme, M.; Dapprich, S.; Gobbi, A.; Hollwarth, A.; Jonas, V.; Kohler, K. F.; Stegmann, R.; Veldkamp, A.; Frenking, G. A set of f-polarization functions for pseudo-potential basis sets of the transition metals SC-Cu, Y-Ag and La-Au. *Chem. Phys. Lett.* **1993**, *208*, 111-114.

(24) (a) Hehre, W. J.; Ditchfield, R.; Pople, J. A. Self-Consistent Molecular Orbital Methods. XII. Further Extensions of Gaussian-Type Basis Sets for Use in Molecular Orbital Studies of Organic Molecules. *J. Chem. Phys.* **1972**, *56*, 2257-2261. (b) Francl, M. M.; Pietro, W. J.; Hehre, W. J.; Binkley, J. S.; Gordon, M. S.; DeFrees, D. J.; Pople, J. A. Self-consistent molecular orbital methods. XXIII. A polarization-type basis set for second-row elements. *J. Chem. Phys.* **1982**, *77*, 3654-3665.
